# Selective oxymetalation of terminal alkynes *via* 6-*endo* cyclization: mechanistic investigation and application to the efficient synthesis of 4-substituted isocoumarins†
†Electronic supplementary information (ESI) available: Additional experimental data, characterization, calculation data and experimental details. CCDC 1576342–1576344 and 1579824. For ESI and crystallographic data in CIF or other electronic format see DOI: 10.1039/c8sc01537f


**DOI:** 10.1039/c8sc01537f

**Published:** 2018-06-15

**Authors:** Yuji Kita, Tetsuji Yata, Yoshihiro Nishimoto, Kouji Chiba, Makoto Yasuda

**Affiliations:** a Department of Applied Chemistry , Graduate School of Engineering , Osaka University , 2-1 Yamadaoka, Suita , Osaka 565-0871 , Japan . Email: yasuda@chem.eng.osaka-u.ac.jp; b Frontier Research Base for Global Young Researchers Center for Open Innovation Research and Education (COiRE) , Graduate School of Engineering , Osaka University , 2-1 Yamadaoka, Suita , Osaka 565-0871 , Japan . Email: nishimoto@chem.eng.osaka-u.ac.jp; c Material Science Division , MOLSIS Inc. , 1-28-38 Shinkawa, Chuo-ku , Tokyo 104-0033 , Japan

## Abstract

The cyclization of heteroatom-containing alkynes with π acidic metal salts is an attractive method to prepare heterocycles because the starting materials are readily available and the organometallic compounds are useful synthetic intermediates. A new organometallic species in the heterocyclization provides an opportunity to synthesize heterocycles that are difficult to obtain. Herein, we describe a novel cyclic oxymetalation of 2-alkynylbenzoate with indium or gallium salts that proceeds with an unusual regioselectivity to give isocoumarins bearing a carbon–metal bond at the 4-position. This new type of metalated isocoumarin provided 3-unsubstituted isocoumarins that have seldom been investigated despite their important pharmacological properties. Indium and gallium salts showed high performance in the selective 6-*endo* cyclization of terminal alkynes while boron or other metals such as Al, Au, and Ag caused 5-*exo* cyclization or decomposition of terminal alkynes, respectively. The metalated isocoumarin and its reaction intermediate were unambiguously identified by X-ray crystallographic analysis. The theoretical calculation of potential energy profiles showed that oxyindation could proceed *via* 6-*endo* cyclization under thermodynamic control while previously reported oxyboration would give a 5-membered ring under kinetic control. The investigation of electrostatic potential maps suggested that the differences in the atomic characters of indium, boron and their ligands would contribute to such a regioselective switch. The metalated isocoumarins were applied to organic synthetic reactions. The halogenation of metalated isocoumarins proceeded to afford 4-halogenated isocoumarins bearing various functional groups. The palladium-catalyzed cross coupling of organometallic species with organic halides gave various 4-substituted isocoumarins. A formal total synthesis of oosponol, which exhibits strong antifungal activity, was accomplished.

## Abstract 2

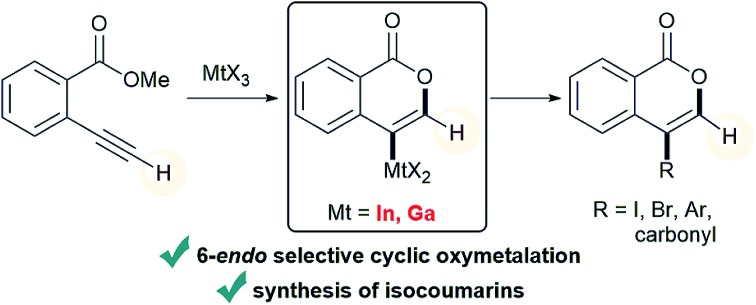
The oxymetalation of terminal alkynes proceeded in a 6-*endo* cyclization manner with the use of indium or gallium trihalide.

## Introduction

**Scheme 1:**
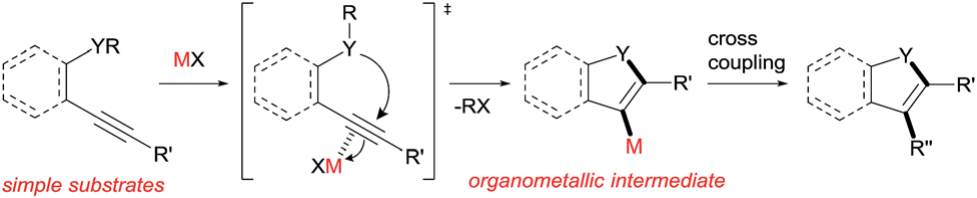
Metal salt-mediated heterocyclization of ω-heteroatom-substituted alkynes.

Heterocyclic compounds have attracted much attention in pharmaceutical chemistry as well as in photochemistry and also play a pivotal role as building blocks in organic synthetic transformation.^1^ Therefore, a novel efficient synthetic method for heterocyclic frameworks is highly desired in various fields of chemistry. Many well-established methods are available in the literature.^2^ The heterocyclization of ω-heteroatom-substituted alkynes using π acidic metal salts is undoubtedly a powerful strategy for the preparation of heterocycles (Scheme 1).^3^ This addition reaction uses readily available alkynes as a starting material. Furthermore, metal salt-mediated cyclization spontaneously forms a carbon–metal bond and a heterocyclic framework and produces organometallic intermediates leading to target heterocycles *via* appropriate synthetic reactions such as cross coupling. These features allowed us to directly access various substituted heterocycles from simple organic substrates.

**Scheme 2:**
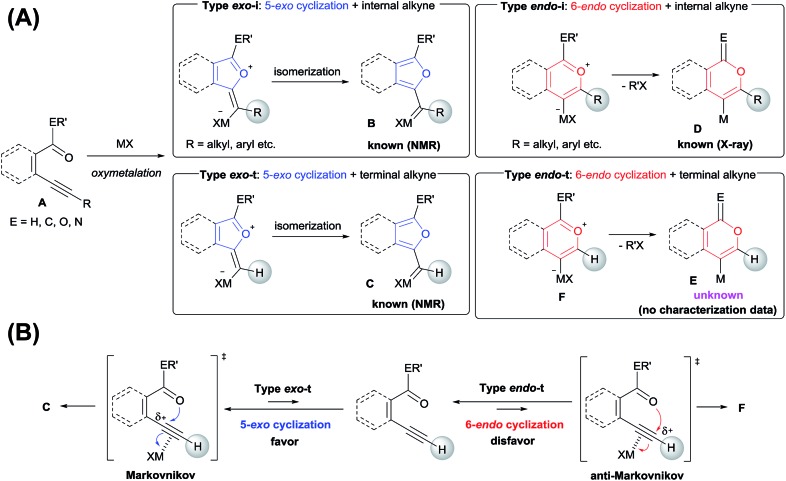
(A) Four types of metalated heterocycles from the oxymetalation of alkyne **A** including a carbonyl moiety. (B) Comparison of the transition states of Type *exo*-t with Type *endo*-t.

Various heteroatom-containing alkynes have been investigated for use in the synthesis of heterocyclic compounds using π acidic metal salts. Alkyne **A** includes a carbonyl moiety and is a feasible and beneficial substrate to obtain 5- or 6-membered oxacyclic alkenylmetals (Scheme 2A). When **A** is treated with a metal salt (MX), oxymetalation proceeds to afford heterocyclic compounds through either 5-*exo* or 6-*endo* cyclization (Type *exo* or *endo*). Considering that the structure of **A** bears either an internal (R = alkyl, aryl, *etc.*) or a terminal alkyne (R = H), oxymetalation can be distinguished by four types of reaction courses: Type *exo*-i, Type *exo*-t, Type *endo*-i, and Type *endo*-t. In Types *exo*-i and *exo*-t, the furan frameworks **B** and **C** have a metal carbenoid moiety and are obtained *via* the isomerization of zwitterion intermediates.^4^,^5^ On the other hand, Types *endo*-i and *endo*-t lead to 1*H*-isochromen derivatives **D** and **E***via* the elimination of R′X from zwitterion intermediates.^6^,^7^ Among these four types, only Type *endo*-t is kinetically unfavorable due to an unstable cationic transition state *via* an anti-Markovnikov addition manner (Scheme 2B). Furthermore, recent theoretical research about the regioselectivity of cyclization revealed that the nucleophilic cyclization of alkynes displays *exo* selectivity intrinsically.^8^ On the other hand, Lewis acidic metals can promote *endo* cyclization by decrease of the stereoelectronic penalty, but the *exo* cyclization was not disturbed, and, thus, the cyclization showed low selectivity.^7^,^9^ For the above reasons, there is no report of a preparation method for species **E***via* Type *endo*-t in contrast to the cases of Types *exo*-i, *exo*-t, and *endo*-i, for which target organometallic compounds (**B–D**) are well established.4b,5a,6c,6e If the reaction course of oxymetalation is realized from **A** to **E** in Type *endo*-t,^10^ various 6-membered heterocycles based on **E**, which have been difficult to obtain and remain unknown, should be synthesized. Therefore, the establishment of a strategy for Type *endo*-t is an important challenge in heterocyclic chemistry.

**Scheme 3:**
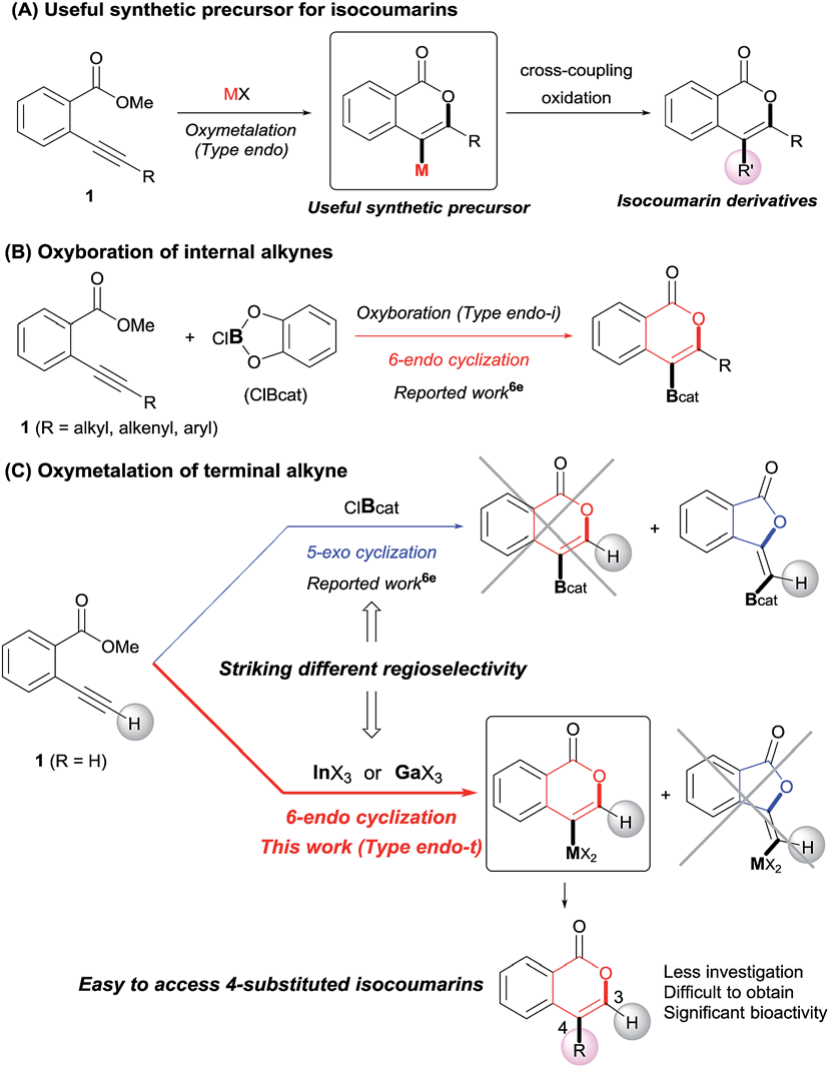
(A) Oxymetalation of 2-alkynylbenzoate **1** followed by transformation for the construction of isocoumarins. (B) Previously reported oxyboration of internal alkynes to generate 4-borylated isocoumarins. (C) Oxymetalation of terminal alkynes, reported oxyboration for 5-membered compounds (blue path), and our developed oxymetalation for 6-membered compounds (red path).

Isocoumarins are an important class of oxygen-containing heterocycles that exhibit a wide range of pharmacological properties.^11^ Thus, the development of their general synthetic method has attracted much attention. The reaction of Type *endo* would be a powerful tool for the synthesis of isocoumarins (Scheme 3A). In fact, reports have described the oxymetalation of 2-alkynylbenzoate **1** (R = alkyl, alkenyl, aryl) for Type *endo*-i and application to the synthesis of isocoumarins.6a,6b,6e Recently, Blum reported an excellent method for the construction of 4-borylated isocoumarins by the oxyboration of the internal alkynes **1** in the Type *endo*-i reaction course (Scheme 3B).6*e* However, terminal alkyne **1** (R = H) gave only a 5-*exo* cyclization product according to the Markovnikov rule (blue path in Scheme 3C).6*e* This result prompted us to explore the oxymetalation of the terminal alkynes **1** for Type *endo*-t. The oxymetalation of Type *endo*-t provides 3-unsubstituted and 4-substituted isocoumarins that are seldom investigated due to the lack of synthetic methods,^12^ and limited substituents have been introduced at the 4-position despite the well-known beneficial significant bioactivity characteristics such as antitumor,^13^ antiangiogenic,^14^ antifungal,^15^ and antibiotic.^16^

**Scheme 4:**
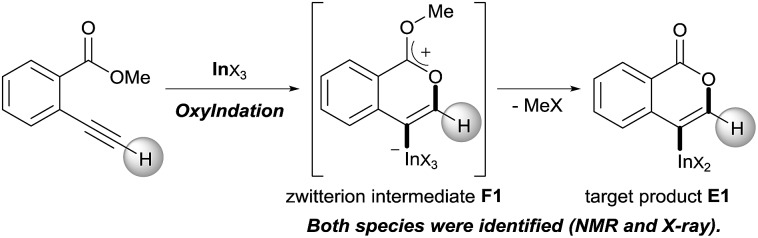
Oxyindation of alkynes for the synthesis of an isocoumarin framework *via* a zwitterion intermediate.

Our group developed the indium or gallium salt-mediated carbometalation of simple terminal alkynes with silyl ketene acetals by utilizing their high π electron affinity and moderate Lewis acidity.^17^ In this context, we investigated the Type *endo*-t reaction of 2-ethynylbenzoate using indium or gallium trihalides for the synthesis of corresponding metalated isocoumarins. In this report, we successfully achieved a 6-*endo* selective oxymetalation of terminal alkynes and fully characterized the target organometallic species **E1***via* an NMR study and X-ray crystallographic analysis. Furthermore, the intermediate **F1** was isolated, which revealed that oxymetalation proceeds *via* the zwitterion intermediate **F1**, and the elimination of the alkyl halide gives the target product **E1** (Scheme 4). While benzopyrylium species such as **F** are known as highly reactive intermediates in the proposed catalytic oxymetalation cycle,10a,^18^ the isolation of species **F** is a challenging issue.10e,10f,^19^ To the best of our knowledge, **F1** is the first example of a fully characterized benzopyrylium intermediate **F**. In addition, we fully disclosed the mechanism by combining experimental data and theoretical calculation. These mechanistic investigations were consistent with the achievement of isolation of the zwitterion intermediate and demonstrated that its stability is a crucial point in this remarkable cyclization regioselectivity.

## Results and discussion

### Optimization of reaction conditions

First, we examined the effect of Lewis acids on oxymetalation using methyl 2-ethynylbenzoate **1a** (Table 1). The reaction of **1a** with metal halides was carried out in toluene at 50 °C, and the reaction mixture was quenched with acetic acid. The reaction using InCl_3_ afforded the target isocoumarin **2***via* 6-*endo* cyclization, albeit in a low yield (entry 1). Gratifyingly, InBr_3_ and InI_3_ mediated oxymetalation smoothly proceeded in a 6-*endo* cyclization fashion to give **2** in high yields (entries 2 and 3). In these cases, the reaction mixture was quenched with deuterated acetic acid to afford **2** bearing deuterium at the 4-position. We did not observe an isocoumarin bearing deuterium at the 3-position which could be produced through the generation of indium acetylide^20^ followed by Lewis acid mediated cycloaddition. The reaction using InI_3_ showed a higher ratio of D/H than the case of InBr_3_. This result suggested the more efficient generation of the alkenylmetal intermediate **X** in the case of InI_3_. Gallium salts were also suitable for the 6-*endo* cyclization of **1a**, and GaI_3_ gave a high yield (entries 4 and 5). On the other hand, typical Lewis acids such as AlCl_3_, AlI_3_, BBr_3_ and TiCl_4_ were ineffective (entries 6–9). Transition metal salts such as PdCl_2_, CuBr_2_ and FeBr_3_ provided no target product and resulted in a decomposition of **1a** (entries 10–12). Alkynophilic π-acids such as gold and silver salts were subjected to the present cyclization. It was found that AuCl_3_, AuCl, AgOTf and AuCl/AgOTf resulted in low yields (entries 13–16). A decrease in yield was observed at lower temperature (entry 17). The solvent effect was examined on oxyindation using InI_3_. Dichloroethane as a solvent provided a good yield while chlorobenzene and hexane afforded only moderate yields (entries 18–20). The yields were appreciably decreased in CH_3_CN and THF (entries 21 and 22) probably because the coordination of these solvents to InI_3_ decreased the Lewis acidity. Finally, InI_3_ was the most effective Lewis acid, and, therefore, we chose entry 3 to represent the optimal conditions.

**Table 1:** Effect of Lewis acids on the oxymetalation of 2-ethynylbenzoate **1a**^*a*^

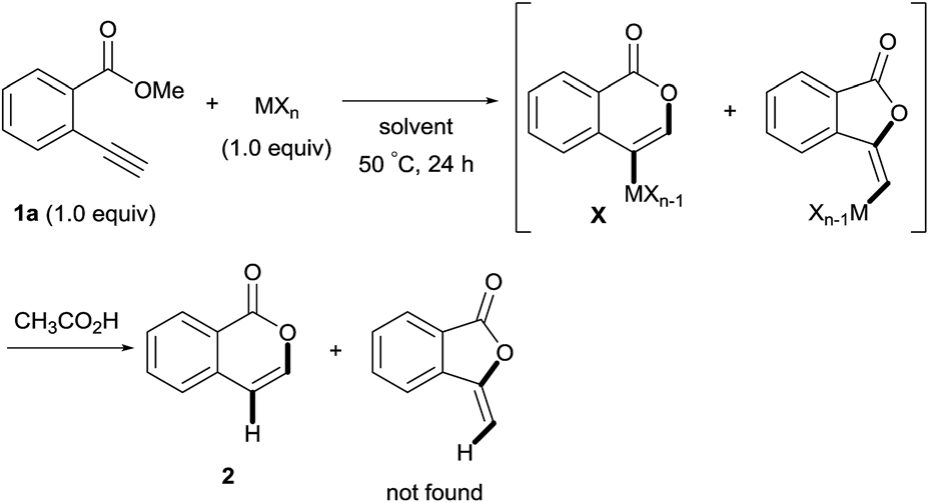			
Entry	MX_*n*_	Solvent	Yield^*b*^ of **2** (%)
1	InCl_3_	Toluene	13
2^*c*^	InBr_3_	Toluene	82 (77% D)
3^*c*^	InI_3_	Toluene	79 (91% D)
4	GaBr_3_	Toluene	30
5	GaI_3_	Toluene	77
6	AlCl_3_	Toluene	0
7	AlI_3_	Toluene	0
8	BBr_3_	Toluene	0
9	TiCl_4_	Toluene	0
10	PdCl_2_	Toluene	0
11	CuBr_2_	Toluene	0
12	FeBr_3_	Toluene	0
13	AuCl_3_	Toluene	7
14	AuCl	Toluene	5
15	AgOTf	Toluene	31
16	AuCl/AgOTf	Toluene	18
17^*d*^	InI_3_	Toluene	61
18	InI_3_	ClCH_2_CH_2_Cl	78
19	InI_3_	ClC_6_H_5_	57
20	InI_3_	Hexane	57
21	InI_3_	CH_3_CN	17
22	InI_3_	THF	0

^*a*^Reaction conditions: **1a** (0.5 mmol), Lewis acid MX_*n*_ (0.5 mmol), solvent (1 mL), 50 °C, 24 h.

^*b*^The yield of **2** was determined by ^1^H NMR.

^*c*^The reaction mixture was quenched with CH_3_CO_2_D (30 equiv., 5 min) and a subsequent addition of H_2_O (10 mL).

^*d*^35 °C.

### Mechanistic investigation

**Fig. 1:**
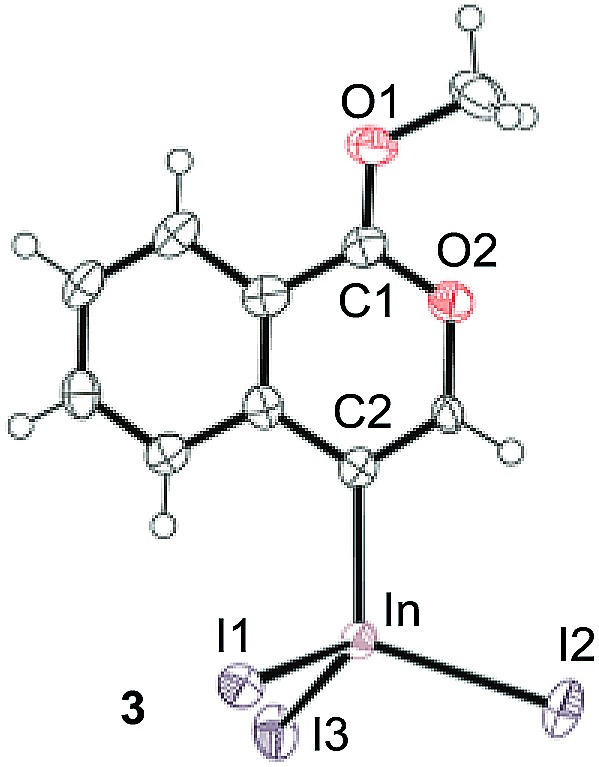
The X-ray crystallographic structure of zwitterion intermediate **3** with the thermal ellipsoids shown at 50% probability (CCDC ; 1579824). Selected bond lengths (Å): C1–O1 = 1.267(9), C1–O2 = 1.298(11), C2–In = 2.171(7), In–I1 = 2.7392(8), In–I2 = 2.7219(8), and In–I3 = 2.6915(7).

**Fig. 2:**
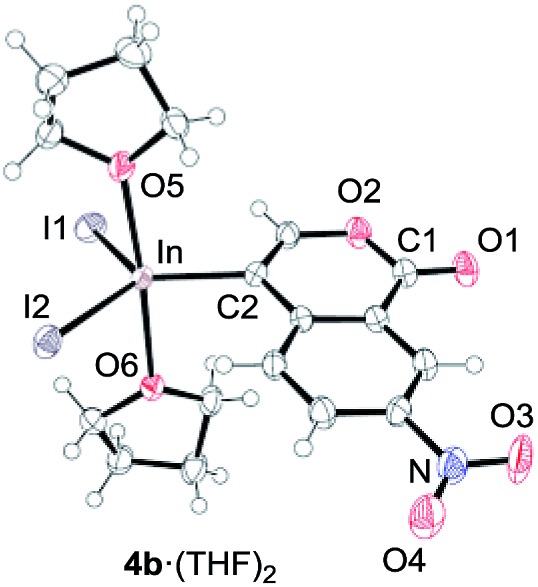
The X-ray crystallographic structure of an isocoumarin including a carbon–indium bond at the 4-position, **4b**·(THF)_2_, with the thermal ellipsoids shown at 50% probability (CCDC ; 1576342). Selected bond lengths (Å) and angles (deg): C1–O1 = 1.211(4), C1–O2 = 1.367(5), C2–In = 2.162(3), In–I1 = 2.7148(4), In–I2 = 2.7005(4), In–O5 = 2.318(3), In–O6 = 2.371(3), O5–In–O6 = 175.44(10), I1–In–C2 = 116.70(11), C2–In–I2 = 125.51(11), and I2–In–I1 = 117.621(12).

To gain insight into the reaction mechanism, we used ^1^H NMR spectroscopy to monitor the oxyindation. When 2-alkynylbenzoate **1a** was mixed with InI_3_ in CDCl_3_ at –30 °C, no reaction occurred. At –5 °C, some amount of a new product was observed (see Fig. S1 and S2† in the ESI). At room temperature, a large amount of white precipitation was formed. This white solid was also obtained in the reaction of **1a** with InI_3_ in toluene at room temperature (eqn (1)). X-ray crystallographic analysis revealed that the white solid was a 6-membered oxacyclic zwitterion **3** bearing a carbon–indium bond (Fig. 1). The bond lengths of the two carbon–oxygen bonds (C1–O1 = 1.267 Å and C1–O2 = 1.298 Å) in the zwitterion **3** existed between a C–O double bond (1.203 Å) and the single bond (1.377 Å) of a typical isocoumarin derivative,^21^ and, thus, the positive charge was delocalized in an ester moiety. The indium atom was coordinated to three iodines and showed a distorted tetrahedral structure with a formal negative charge. The formed zwitterionic alkenyl indium **3** was heated at 50 °C in toluene to give a neutral alkenylindium product **4a**, quantitatively by the elimination of MeI (eqn (2)). Although a suitable single crystal of **4a** for X-ray analysis was not obtained, we successfully conducted X-ray diffraction analysis of nitro-substituted alkenylindium **4b** produced from 2-ethynyl-5-nitrobenzoate **1b** (eqn (3) and Fig. 2). The bond lengths of C1–O1 (1.211 Å) and C1–O2 (1.367 Å) were similar to those of a reported isocoumarin framework.^21^ The indium complex **4b** displayed trigonal bipyramidal coordination with two THF ligands in axial positions. These results indicated a two-step pathway including a fast cyclization and a slow elimination of MeI during the 6-*endo* oxyindation process from **1** to **4**.1
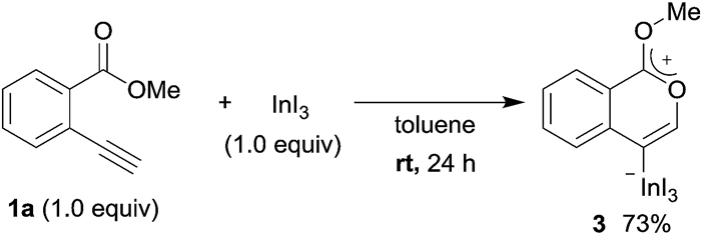

2
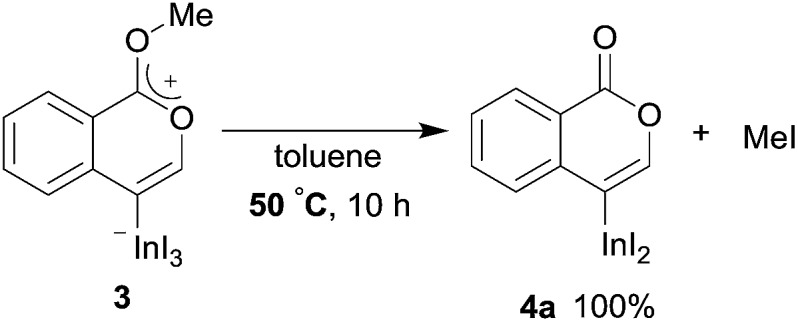

3
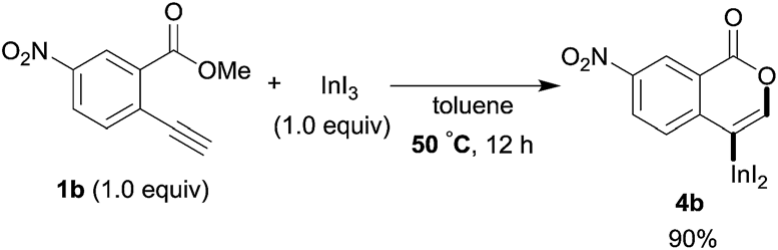



### Theoretical calculation for oxyindation

**Scheme 5:**
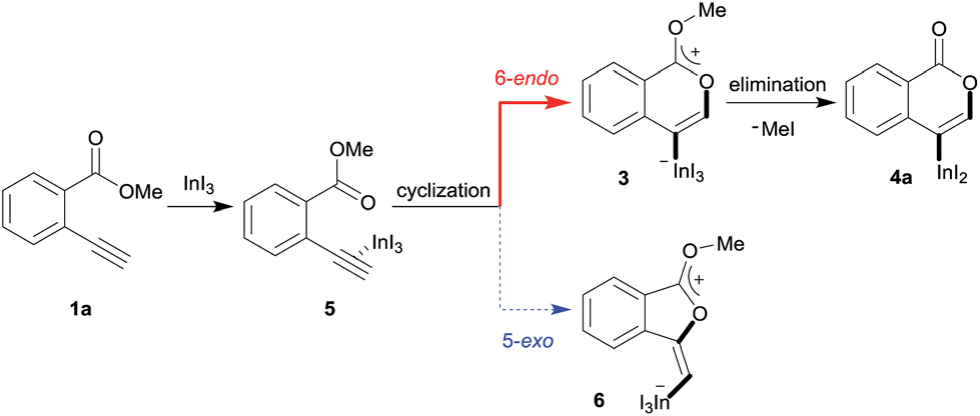
A proposed mechanism for the formation of the isocoumarin **4a**.

A mechanism for the formation of the target isocoumarin **4a** using InI_3_ is proposed in Scheme 5, wherein InI_3_ is coordinated to the alkyne moiety in **5**, oxyindation proceeds *via* 6-*endo* cyclization to give the zwitterion intermediate **3**, and, finally, the elimination of MeI affords **4a**.

**Fig. 3:**
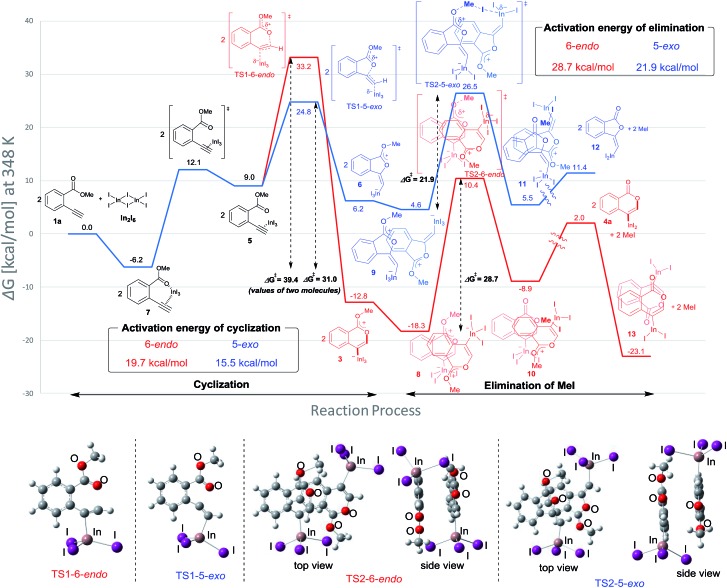
The energy profiles of 6-*endo* and 5-*exo* oxyindations and 3D molecular structures of transition states. DFT calculation was performed using wB97XD/6-31+G (d,p) for C, H, and O and using DGDZVP for In and I. Solvation effect was introduced using the IEFPCM model, and toluene was used as a solvent.

Density functional theory (DFT) calculations were performed to more thoroughly consider the reaction mechanism. The calculation of the potential energy profile for 6-*endo* cyclization (red) is shown in Fig. 3. We selected **1a** and In_2_I_6_ as the starting materials because InI_3_ exists in a dimer fashion.^22^ The coordination of two **1a** to In_2_I_6_ dissociates the aggregation of InI_3_ to give complex **7**, in which InI_3_ is chelated with the alkyne moiety and carbonyl group of **1a** (Fig. S3† in the ESI shows the detailed mechanism of generating the complex **7** from **1a** and In_2_I_6_). The dissociation of the carbonyl oxygen atom generates complex **5**, in which InI_3_ directly activates the alkyne moiety. In this pathway, the anti-addition of InI_3_ and the ester moiety to the alkyne moiety proceeds in a concerted mechanism to provide a stable 6-membered zwitterion intermediate **3**. The elimination of MeI proceeds in an intermolecular fashion, because the intramolecular elimination of MeI requires a very unstable intermediate (Fig. S4† in the ESI shows the potential energy profile for the intramolecular elimination of MeI). Two zwitterions aggregate in a head-to-tail fashion to give complex **8**, and then the elimination step starts from **8**. The intermolecular nucleophilic substitution of the methyl group by I^–^ proceeds in an S_N_2-mechanism to give complex **10** and MeI, and then a subsequent elimination of MeI affords the target product **4a**.^23^ A carbonyl group of **4a** coordinates to the indium atom of another **4a** to give the stable dimeric product **13**. The activation energy of the elimination step (**8** to TS2-6-*endo*, 28.7 kcal mol^–1^) is much higher than that of the cyclization step (**7** to TS1-6-*endo*, 19.7 kcal mol^–1^).^24^ Therefore, the elimination of MeI is a rate-determining step. We also calculated the 5-*exo* cyclization pathway (blue) to investigate the regioselectivity. This process proceeds *via* concerted cyclization, wherein the 5-membered zwitterion **6** is much more unstable than the 6-membered version **3**. The intermolecular elimination of MeI takes place in an S_N_2-manner (**9** to **11**). The transition state (TS2-5-*exo*) shows the highest energy level, and it is even higher than the energy profile of the 6-*endo* cyclization process (red) due to the instability of the 5-membered zwitterion **6**.

In order to clarify the unique 6-*endo* cyclization selectivity of oxyindation, the energy profiles of the two cyclization manners were compared. The activation energy of 5-*exo* cyclization is lower (**7** to TS1-5-*exo*, 15.5 kcal mol^–1^) than that of 6-*endo* cyclization (**7** to TS1-6-*endo*, 19.7 kcal mol^–1^). However, 5-*exo* cyclization is reversible because the activation energy for the elimination of MeI (**9** to TS2-5-*exo*, 21.9 kcal mol^–1^) is much higher than that of retro-cyclization (**6** to TS1-5-*exo*, 9.3 kcal mol^–1^) due to the instability of the zwitterion **6**. On the other hand, during 6-*endo* cyclization, both activation energies of elimination (**8** to TS2-6-*endo*, 28.7 kcal mol^–1^) and retro-cyclization (**3** to TS1-6-*endo*, 23.0 kcal mol^–1^) are high because the 6-membered zwitterion intermediate **3** is thermodynamically stable. This result indicates that 6-*endo* cyclization is irreversible and the most thermodynamically stable form of intermediate **8** is exclusively generated to provide the target product **4a**, which is consistent with the successful isolation of the zwitterion intermediate **3** (Fig. 1). Therefore, oxyindation proceeds under thermodynamic control to afford the stable 6-membered product **4a**. We also calculated an energy profile of InCl_3_-mediated oxyindation and found the same pathway with the case of InI_3_ (see Fig. S5† in the ESI). The activation energy of the elimination step in the case of InCl_3_ is higher than that of InI_3_ because of the low nucleophilicity of Cl^–^, and it caused much less reactivity of InCl_3_ (entry 1, Table 1).

**Fig. 4:**
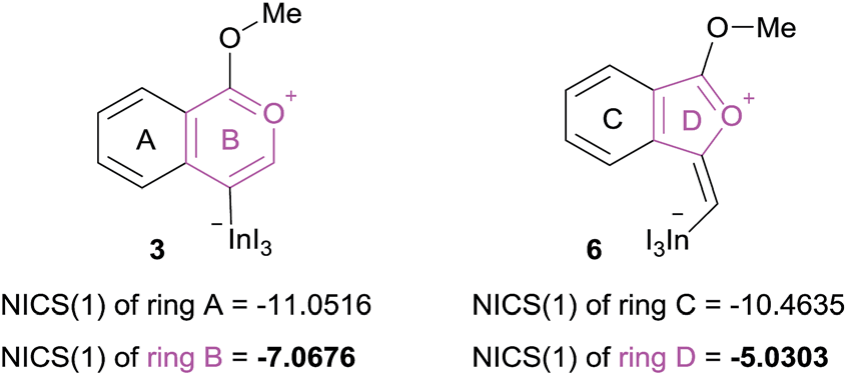
NICS(1) values of 6-membered zwitterion **3** and 5-membered zwitterion **6**. The aromaticity was calculated using B3LYP/6-31G (d,p) for C, H, and O and using DGDZVP for In and I for their optimized structures.

The remarkable regioselectivity of oxyindation is ascribed to the differences in stability between the 6-membered zwitterion **3** and the 5-membered **6**. Zwitterion **3** is much more stable than **6**, and this difference in stability originates from the aromaticity of these compounds, although ring strain is also a consideration. To verify this possibility, the aromaticity of zwitterions was evaluated *via* NICS(1)^25^ (Fig. 4), and the 6-membered compound **3** showed a higher level of aromaticity than **6**.

### Theoretical calculation for oxyboration

**Scheme 6:**
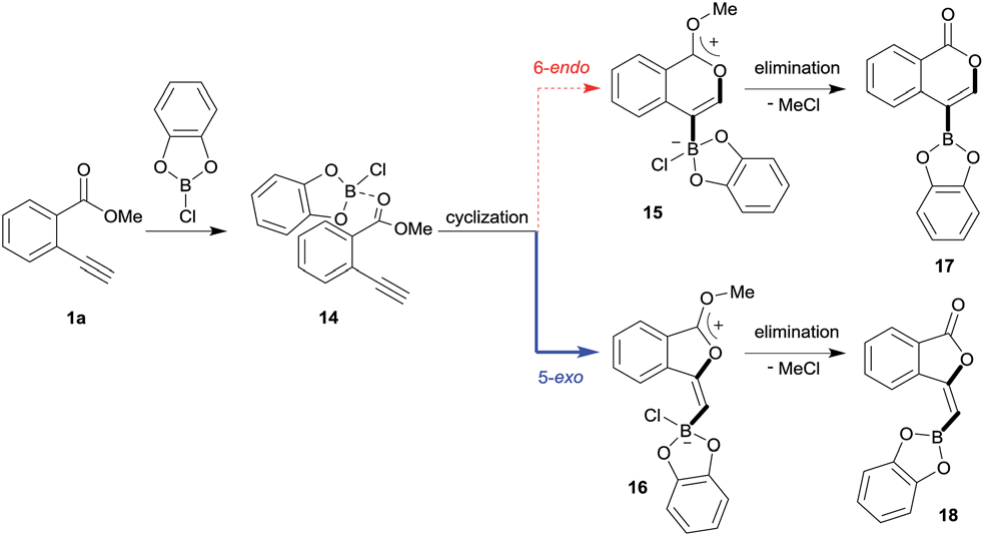
A proposed mechanism for oxyboration.

**Fig. 5:**
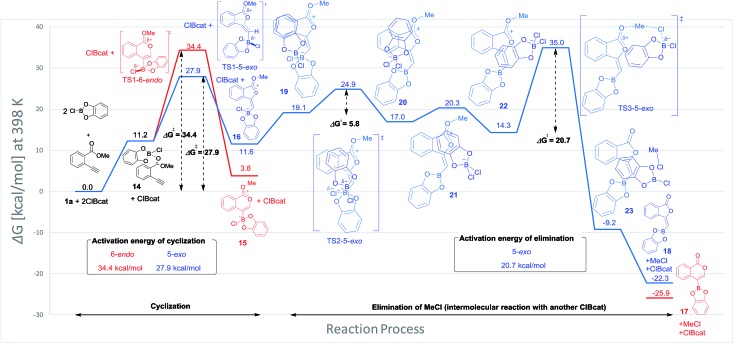
The energy profiles of 5-*exo* and 6-*endo* oxyborations. DFT calculation was performed with wB97XD/6-31+G (d,p) for C, H, O, B, and Cl. Solvation effect was introduced using the IEFPCM model, and toluene was used as a solvent.

**Fig. 6:**
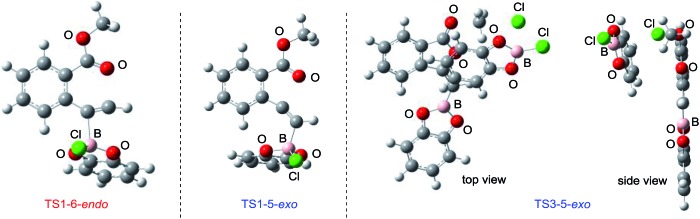
3D molecular structures of transition states in oxyborations.

Blum and co-workers reported that the oxyboration of **1a** using B-chlorocatecholborane (ClBcat) gave a 5-membered product6*e* rather than the 6-membered version (Scheme 6). ClBcat is coordinated to the carbonyl moiety of **1a**. Then, oxyboration proceeds *via* 5-*exo* cyclization to give the zwitterion intermediate **16**, and the elimination of MeCl gives the target product **18**. We also performed DFT calculation of oxyboration to investigate the striking change in the regioselectivity between oxyboration and oxyindation. First, the calculation of oxyboration was performed for a similar oxyindation mechanism *via* concerted cyclization and S_N_2-type elimination of MeCl from aggregated zwitterion intermediates (see Fig. S6† in the ESI). We considered another possibility for the elimination step, because the recent theoretical investigation of ClBcat-mediated heterocyclization has shown other mechanisms,^26^ whereby the Me group is attacked either by dissociated chloride26*a* or by [Cl_2_Bcat]^–^.26*b* Thus, we considered these additional two plausible elimination steps assisted by either free Cl^–^ or [Cl_2_Bcat]^–^ (see Fig. S7† in the ESI and Fig. 5 and 6). The result of comparison between these three pathways showed that the most probable path was the use of [Cl_2_Bcat]^–^ (details of the comparison are shown in the ESI†).

The total reaction profile of oxyboration is described in Fig. 5 and 6. In that profile, 5-*exo* cyclization from **1a** and 2ClBcat to **16** has an activation energy (27.9 kcal mol^–1^) that is lower than that of 6-*endo* cyclization (**1a** and 2ClBcat to **15**, 34.4 kcal mol^–1^). The chloride moiety of zwitterion **16** coordinates to another ClBcat to provide complex **19**. The chloride transfer process (**19** to **20**) has a low energy barrier (5.8 kcal mol^–1^), and [Cl_2_Bcat]^–^ is generated rapidly. Cl in [Cl_2_Bcat]^–^ approaches the methyl group in the ester moiety (**20** → **21** → **22**), and an elimination of MeCl (**22** to **23**) in the S_N_2-mechanism occurs to give 5-membered product **18**. The activation energy of the elimination of MeCl (**22** to TS3-5-*exo*) is 20.7 kcal mol^–1^, which allows the elimination of MeCl to proceed smoothly to give the final product **18**. The fast elimination step allows oxyboration to proceed under kinetic control to accomplish the 5-*exo* selective cyclization.

### Comparing the transition state of the cyclization step in oxyindation with that in oxyboration based on an electrostatic potential map.

**Fig. 7:**
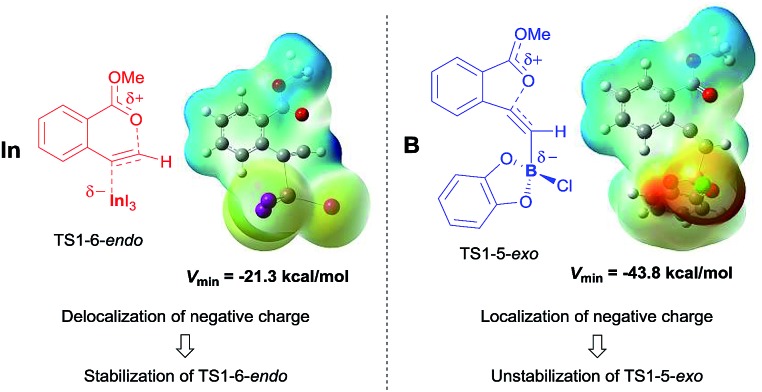
Electrostatic potential maps were calculated on the 0.001 au isosurface of electron density for optimized structures of the transition states of oxyindation (left) and oxyboration (right). The potential is depicted by a color gradient from the most negative (red) to the most positive (blue) value (kcal mol^–1^). *V*_min_ represents the most negative surface electrostatic potential.

The significant difference between oxyindation and oxyboration was investigated because each showed a characteristic energy profile, particularly for the cyclization step. The energy barrier of cyclization in oxyindation (6-*endo*: 19.7 kcal mol^–1^, 5-*exo*: 15.5 kcal mol^–1^) is much lower than that of oxyboration (6-*endo*: 34.4 kcal mol^–1^, 5-*exo*: 27.9 kcal mol^–1^). Therefore, the electrostatic potential maps for the transition states of cyclization (TS1-6-*endo* and TS1-5-*exo*) were calculated (Fig. 7). The value of *V*_min_, which represents the most negative surface electrostatic potential, was investigated to evaluate the degree of localization for a negative charge.^27^ The *V*_min_ of the organoindium species (left, in Fig. 7) was less negative than that of boron (right, in Fig. 7), which showed that the negative charge was delocalized in the transition state of oxyindation compared with oxyboration. The value of *V*_max_, which is the most positive surface electrostatic potential, was also calculated and was less affected by the differences in the metals (see Table S2† in the ESI). The polarizability of the indium, boron and heteroatoms binding to a metal explained these results. Indium and iodine atoms have large polarizability (*α*_In_ = 69 a.u. and *α*_I_ = 35.1 a.u.),^28^ and the increasing negative charge in the TS1 of oxyindation was efficiently delocalized to stabilize the zwitterionic TS1-6-*endo*.^29^ On the other hand, boron, chlorine and oxygen atoms have smaller polarizability (*α*_B_ = 20.5 a.u., *α*_Cl_ = 14.7 a.u., and *α*_O_ = 6.04 a.u.)^28^ than indium and iodine atoms, so that TS1-5-*exo* becomes unstable due to the localization of a negative charge. The difference in the fundamental features between indium and boron atoms imparts a significant amount of influence on the regioselectivity of oxymetalation.

### Summary of DFT calculation

**Fig. 8:**
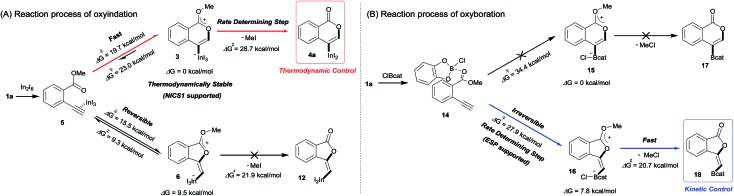
The summarized results of DFT calculation.

In oxyindation (Fig. 8A), the activation energy of 5-*exo* cyclization is much lower than that required for the elimination of MeI to lead to reversible 5-*exo* cyclization. Therefore, the thermodynamically stable 6-membered zwitterion **3** was selectively produced to accomplish the remarkable 6-*endo* selectivity. The elimination step from **3** is a rate-determining step that provides the target metalated isocoumarin **4a**. On the other hand, the energy barrier for cyclization in oxyboration (Fig. 8B) is higher than that for the elimination of MeCl and the cyclization step is a rate-determining step, which leads to irreversible 5-*exo* cyclization to afford the 5-membered product **18** under kinetic control. Therefore, the activation energies of cyclization as well as elimination are important factors to determine the regioselectivity in cyclization.

### Application to the synthesis of isocoumarin derivatives

**Scheme 7:**
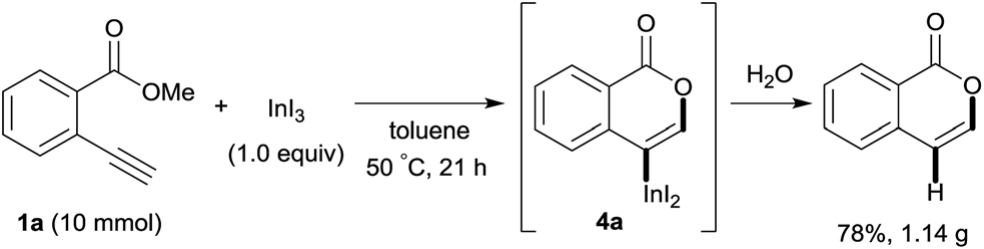
Gram-scale synthesis of isocoumarin including a carbon–indium bond.

Our developed oxyindation was applied to the synthesis of isocoumarin derivatives. First, the gram-scale synthesis of an organoindium species was carried out. Methyl ester **1a** (10 mmol) reacted with InI_3_ to give organoindium **4a**, and 1.14 g of isocoumarin was isolated by the addition of H_2_O (Scheme 7).

Next, the oxidation of the produced alkenylindium compounds was performed (Table 2). An oxyindation of **1a** using InI_3_ was carried out, and the organoindium **4a** was oxidized with PhI(OAc)_2_ in a one-pot procedure to give 4-iodoisocoumarin **24a** (entry 1). Subjecting InBr_3_ to the oxidation reaction provided 4-bromoisocoumarin **25a** in a high yield (entry 2). Therefore, various types of 2-alkynylbenzoates were surveyed in the sequential oxyindation/halogenation process to give 4-halogenated isocoumarins. Substrates with electron withdrawing groups such as nitro and carbonyl groups gave the target products **24b** and **24c** in high yields (entries 3 and 4). The structure of **24b** was characterized by X-ray crystallographic analysis (see Fig. S11† in the ESI). Substrates with methyl or aryl groups efficiently afforded the target isocoumarins **24d** and **24e** (entries 5 and 6). Also, 2-alkynylbenzoates, including halogen moieties (Br, Cl and F), were suitable for this reaction system to give the isocoumarins **25f–24h** in moderate yields (entries 7–9). The synthesis of isocoumarins from internal alkynes was also investigated. The optimization of the reaction conditions showed that gallium salts were more suitable than indium salts for the oxymetalation of an internal alkyne (see Table S3† in the ESI). Therefore, gallium salts were employed in the reactions of internal alkynes **1i–1k** to provide the 3,4-disubstituted isocoumarins **24i**, **24j** and **25k** (entries 10–12).

**Table 2:** Sequential oxymetalation/halogenation of various types of 2-alkynylbenzoate **1**^*a*^

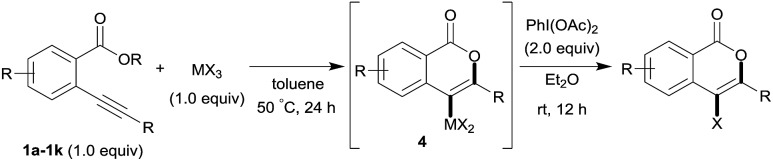				
Entry	**1**	MX_3_	Target	Yield^*b*^ (%)
1	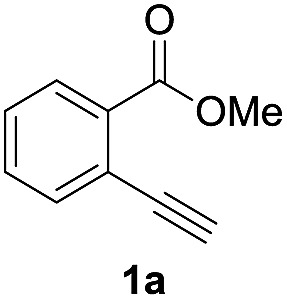	InI_3_	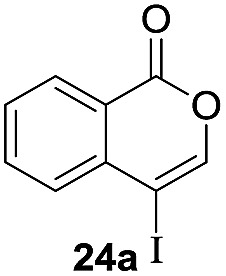	64
2	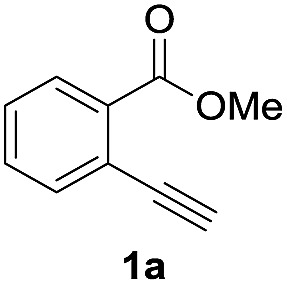	InBr_3_	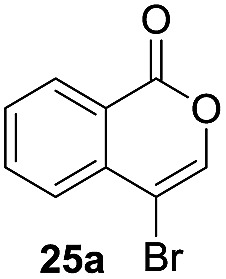	73
3	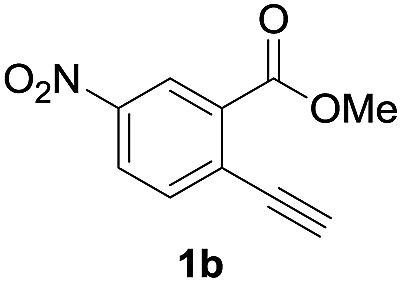	InI_3_	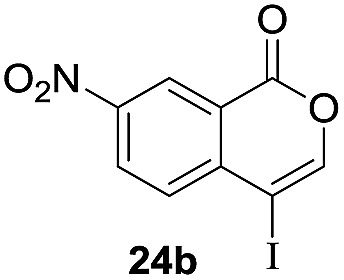	61
4	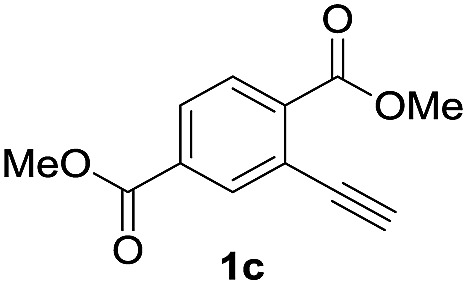	InI_3_	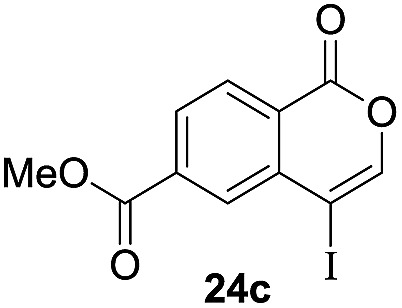	70
5	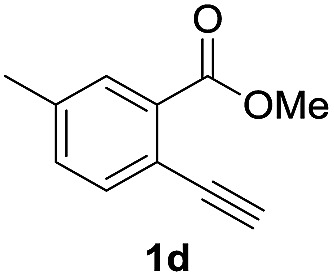	InI_3_	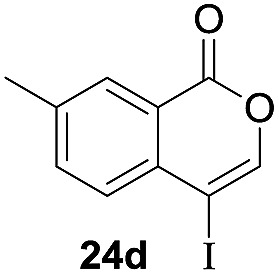	54
6	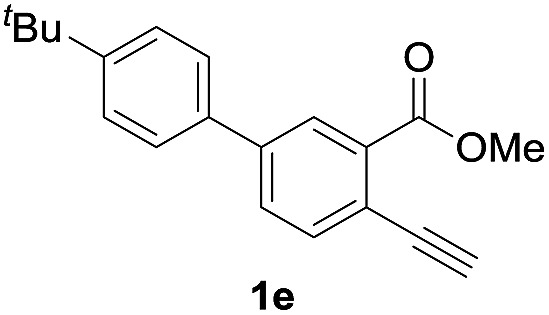	InI_3_	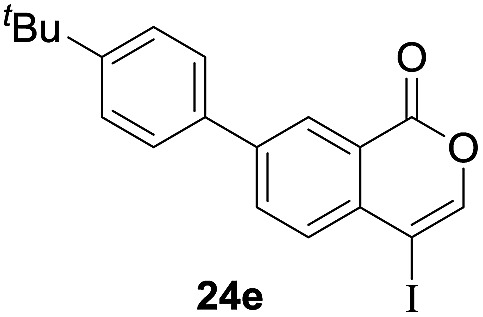	54
7	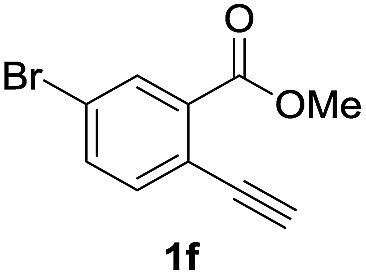	InBr_3_	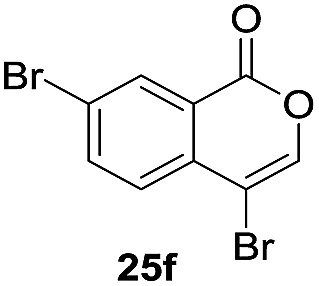	47
8	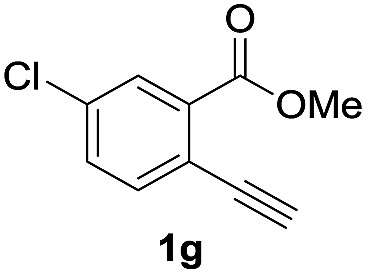	InI_3_	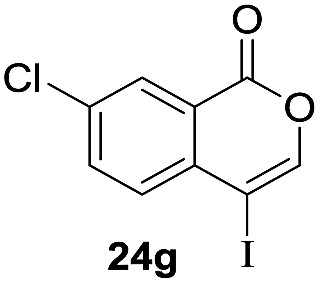	67
9	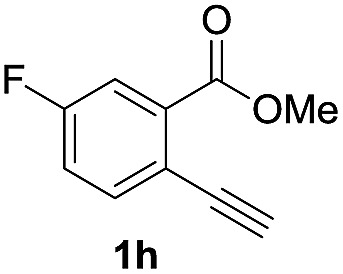	InI_3_	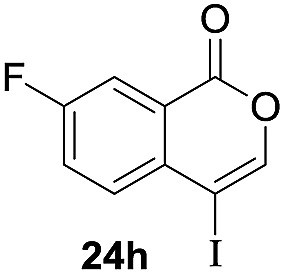	61
10	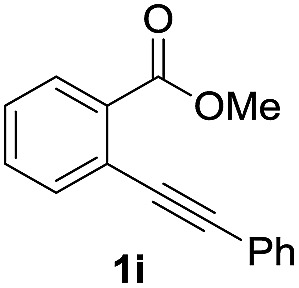	GaI_3_	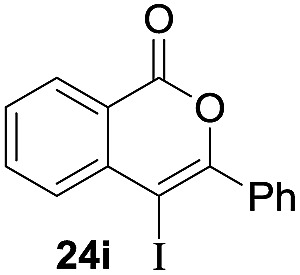	60
11	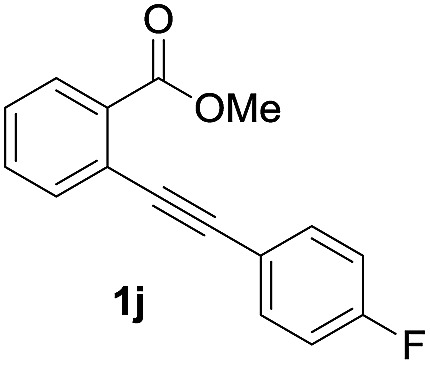	GaI_3_	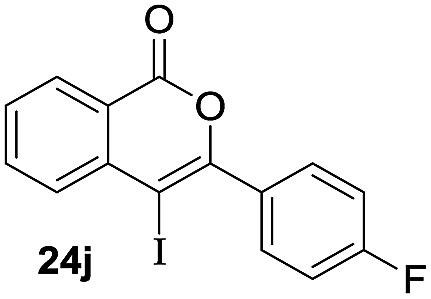	56
12	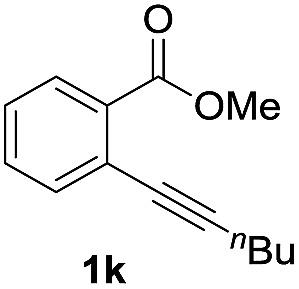	GaBr_3_	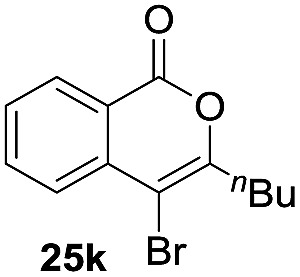	60

^*a*^First step: **1** (0.5 mmol), MX_3_ (0.5 mmol), toluene (1 mL), 50 °C, 24 h. Second step: PhI(OAc)_2_ (1.0 mmol), Et_2_O (1 mL), rt, 12 h.

^*b*^Isolated yields.

One-pot syntheses of 4-substituted isocoumarins were performed *via* oxyindation followed by a palladium-catalyzed cross-coupling reaction (Table 3).^30^ After the oxymetalation of **1a** using InBr_3_, the addition of a palladium catalyst, lithium chloride, organic halides **27**, and an additional solvent to the resultant toluene solution afforded the coupling product **28**. Iodobenzene **27a** and aryl iodides bearing an electron donating group **27b** or an electron withdrawing group **27c** were applicable to give the 4-arylisocoumarins **28aa–28ac** in high yields (entry 1). Palladium-catalyzed cross coupling with acid chlorides also proceeded efficiently. Reactions using the benzoyl chloride derivatives **27d** and **27e**, as well as the alkanoyl chloride **27f**, afforded the isocoumarins **28ad–28af** with ketone moieties in good yields (entries 2 and 3). The structure of **28ae** was characterized by X-ray crystallographic analysis (see Fig. S12† in the ESI). In this reaction system, alkyl halides such as benzyl bromide **27g** and allyl bromide **27h** were also suitable to give 4-alkylisocoumarins **28ag** and **28ah**, respectively (entries 4 and 5). Various types of 4-substituted isocoumarins were obtained from an isocoumarin that included a carbon–indium bond by utilizing palladium-catalyzed cross coupling.

**Table 3:** One-pot formation of 4-substituted isocoumarins by palladium-catalyzed cross coupling of organoindium species **26** with organic halides **27**^*a*^

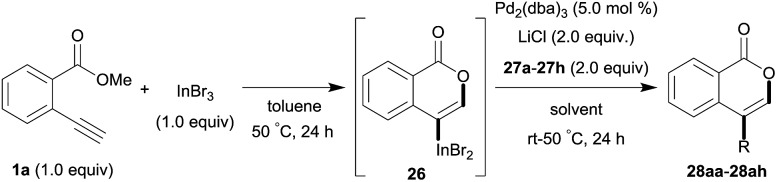			
Entry	**27**	Target	Yield^*b*^ (%)
1	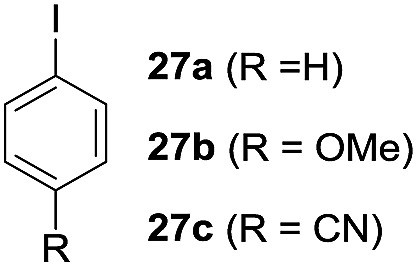	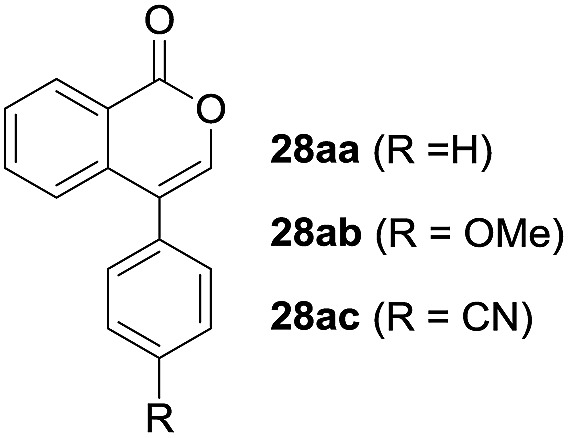	**28aa**: 81**28ab**: 71**28ac**: 72
2	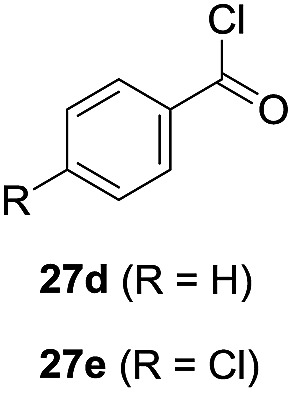	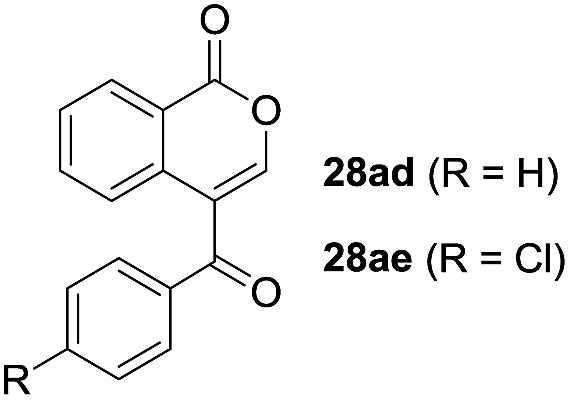	**28ad**: 82**28ae**: 64
3^*c*^	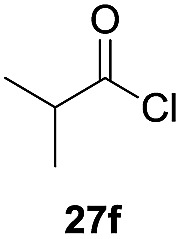	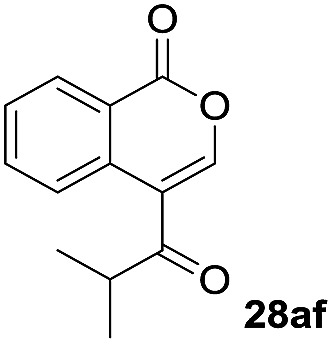	61
4^*d*^	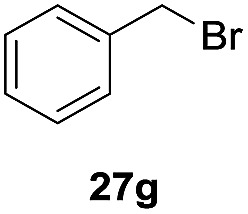	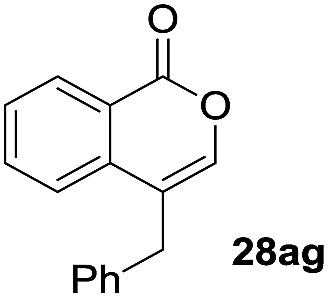	51
5^*d*^	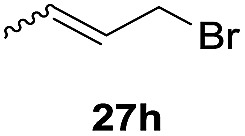	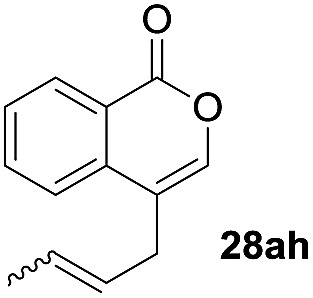	45^*e*^

^*a*^Basic reaction conditions of the first step: **1a** (0.5 mmol), InBr_3_ (0.5 mmol), toluene (1 mL), 50 °C, 24 h. Second step: Pd_2_dba_3_ (0.025 mmol), LiCl (1.0 mmol), **27** (1.0 mmol), NMP (2.5 mL), 50 °C, 24 h.

^*b*^Isolated yields.

^*c*^HMPA (2.5 mL), rt, 24 h.

^*d*^HMPA (2.5 mL), 50 °C, 24 h.

^*e*^ *E*/*Z* = 90 : 10.

### Formal total synthesis of oosponol

**Scheme 8:**
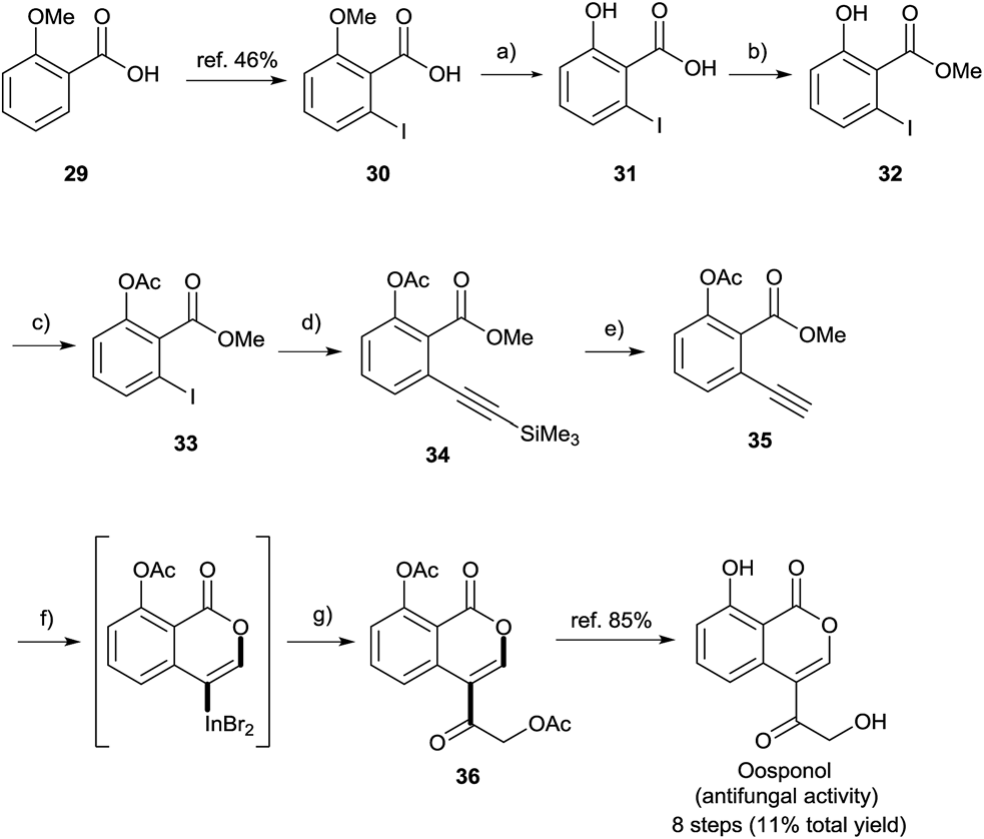
Formal total synthesis of oosponol. Reagents and reaction conditions: (a) BBr_3_ (1 M in CH_2_Cl_2_, 2.0 equiv.), CH_2_Cl_2_, rt, 20 h, 100%. (b) H_2_SO_4_ (20 mol%), MeOH, reflux, 20 h, 87%. (c) AcCl (1.04 equiv.), pyridine (1.04 equiv.), acetone, rt, 14 h, 97%. (d) ethynyltrimethylsilane (1.1 equiv), PdCl_2_(PPh_3_)_2_ (2.0 mol%), CuI (20 mol%), NEt_3_, RT, 17 h, 100%. (e) 1 M KF aq. (1.65 equiv.), DMF, RT, 0.5 h, 76%. (f) InBr_3_ (1.0 equiv.), Toluene, 50 °C, 24 h. (g) Pd_2_dba_3_ (5.0 mol%), LiCl (2.0 equiv.), 2-(acetyloxy)acetyl chloride **27i** (2.0 equiv.), HMPA, rt, 9 h, 44%.

Finally, a formal total synthesis of oosponol, which exhibits strong antifungal activity,^15^ was conducted (Scheme 8). Firstly, the iodination of commercially available compound **29** proceeded *via* a method found in the literature.^31^ During the initial investigation, **30** was transformed into methyl 2-ethynyl-6-methoxybenzoate, and then we attempted the synthesis of the precursor of oosponol *via* oxyindation and cross-coupling, but the reaction returned a complicated mixture (see Scheme S1† in the ESI). Therefore, in another synthetic route, the OMe group of **29** was converted to an OAc group with less ability to donate electrons. The OMe moiety of **30** was completely deprotected with BBr_3_. The acid-catalyzed esterification and acetylation of the phenol moiety gave methyl 6-iodoacetylsalicylate **33** in a high yield. Sonogashira coupling followed by the removal of a silyl moiety afforded the desired 2-alkynylbenzoate derivative **35**. The oxymetalation of **35** using InBr_3_ and sequential palladium-catalyzed cross coupling with acid chloride **27i** produced the key intermediate **36**, and the hydrolyzation of **36** yielded oosponol.16*b* Our method used a readily available starting material and gave a higher yield than previous studies.16b,^32^

## Conclusions

We achieved the synthesis of isocoumarins bearing a metal–carbon bond at the 4-position *via* 6-*endo* selective oxymetalation of 2-alkynylbenzoate **1** (Type *endo*-t). Indium and gallium salts showed high activity for the oxymetalation of 2-ethynylbenzoate **1a**. Both the metalated isocoumarin **4b** and the zwitterion intermediate **3** were identified by X-ray crystallographic analysis. This is the first example of the isolation of the product **E** and the benzopyrylium intermediate **F** proposed in the mechanism of oxymetalation (Scheme 2A). The elimination of MeI from zwitterion **3** occurred under heating conditions to give the target product **4a**, which means that the rate-determining step was the elimination step. DFT calculation suggested that thermodynamic control led to 6-*endo* selective oxyindation, while kinetic control led to 5-*exo* selective oxyboration. The 6-membered product proved much more stable than the 5-membered product due to a difference in the degree of aromatic stability. The investigation of the electrostatic potential of the transition state in the cyclization pathway suggested that a delocalization of negative charge by the large atomic radii of In and I atoms stabilizes the zwitterionic transition state. In contrast, the small atomic radii of B, Cl, and O atoms cause a localization of negative charge to destabilize the corresponding transition state. The difference in stability between the 6- and 5-membered zwitterions and the elemental character of InI_3_ both played important roles in the unique regioselectivity of oxymetalation and in the facile preparation of the **E** species.

These isocoumarins bearing a carbon–metal bond at the 4-position were applied to organic synthesis. Oxymetalation provided isocoumarins on a gram scale. The oxidation of organoindium or gallium species yielded various types of 4-halogenated isocoumarins. Palladium-catalyzed cross coupling with aryl iodide, acid chloride, and alkyl bromide gave a wide range of 4-substituted isocoumarins in a one-pot reaction. Therefore, the unprecedented regioselectivity of the present oxymetalation contributed to the synthesis of new types of isocoumarins. We accomplished a formal total synthesis of oosponol to demonstrate the utility of our reaction system.

## Conflicts of interest

There are no conflicts to declare.

## Supplementary Material

Supplementary information

Crystal structure data
