# Supplementary material for: Selective oxymetalation of terminal alkynes via 6-endo cyclization: mechanistic investigation and application to the efficient synthesis of 4-substituted isocoumarins
Source: Chem Sci. 2018 Jun 15;9(28):6041–52. doi: 10.1039/c8sc01537f (PMC6053900; doi:10.1039/c8sc01537f)

Electronic Supplementary Material (ESI) for Chemical Science.
This journal is © The Royal Society of Chemistry 2018

Electronic Supplementary Information

Selective Oxymetalation of Terminal Alkynes via 6-*Endo* Cyclization: Mechanistic Investigation and Application to the Efficient Synthesis of 4-Substituted Isocoumarins

Yuji Kita,[a] Tetsuji Yata,[a] Yoshihiro Nishimoto,*[b] Kouji Chiba[c] and Makoto Yasuda*[a]
[a]Department of Applied Chemistry, Graduate School of Engineering, Osaka University, 2-1 Yamadaoka, Suita, Osaka 565-0871, Japan
[b]Frontier Research Base for Global Young Researchers Center for Open Innovation Research and Education (COiRE), Graduate School of Engineering, Osaka University, 2-1 Yamadaoka, Suita, Osaka 565-0871, Japan
[c]Material Science Div., MOLSIS Inc., Tokyo 104-0033, Japan

Table of Contents

General 2
Material 2
Typical Procedures 10
Monitoring of Oxyindation by ^{1}H NMR Spectroscopy 11
The Effect of *O*-alkyl Groups on the Elimination of Alkyl Halide 12
Formal Total Synthesis of Oosponol via Methyl 2-Ethynyl-6-Methoxybenzoate 12
Product 14
Isolation of Organoindium Compounds 36
Computational Details 42
X-ray Crystallographic Analysis of Isocoumarin Derivatives 151
Optimization of Reaction Conditions for Oxymetalation of Internal Alkyne 152
References 153
NMR Spectra 154

General

New compounds were characterized by ^{1}H, ^{13}C, ^{13}C off-resonance techniques, COSY, HMQC, HMBC, IR, MS, HRMS. ^{1}H (400 MHz) and ^{13}C NMR (100 MHz) spectra were obtained with TMS as internal standard. IR spectra were recorded as thin films or as solids in KBr pellets. High-resolution mass spectra were obtained by magnetic sector type mass spectrometer. Column chromatography was performed on silica gel (MERK C60 or Fuji Silysia FL100DX). All reactions were carried out under nitrogen. Yields were determined by ^{1}H NMR using internal standards (bromoform).

Material

Dehydrated toluene was purchased and used without further purification. 2-Alkynylbenzoate derivatives **1a**, **1c**, **1f**, **1g**, **1i, 1k, 1l, 1m** were synthesized by literature procedures and spectral data of these compounds are shown below. Methyl 2-alkynylbenzoate derivatives **1b**, **1d**, **1e**, **1h**, **1j** are new compounds, and synthetic method and spectral data of these compounds are shown below. All metal salt, organohalides **27a-i** and the starting material of oosponol **29** are commercially available.

(**1a**) Methyl 2-ethynylbenzoate

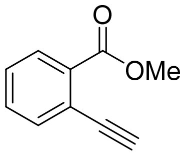

This compound was synthesized by a reported method[1] and the NMR data was agreement with the literature[1]. The spectral data of this compound is shown below.

(**1b**) methyl 2-ethynyl-5-nitrobenzoate

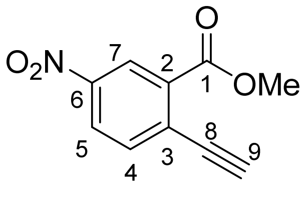

To a three necked flask, methyl 5-nitro-2-((trimethylsilyl)ethynyl)benzoate (2.83 g, 10.2 mmol), MeOH (20 mL) and K_2CO_3 (0.198 g, 1.43 mmol) were added. After stirring for 3 h

at rt, the solvent was evaporated and the residue was diluted by CH_2Cl_2 (20 mL). The solution was washed by water (20 mL) and sat. NaCl aq. (20 mL). The organic layer was dried ($MgSO_4$) and the solvent was evaporated. The residue was purified by column chromatography (hexane/ethyl acetate = 80:20, column length 11 cm, diameter 26 mm, spherical silica gel) to give the product (1.56 g, 74%). IR: (KBr) 1701 (C=O) cm^{-1}, 1523 (NO_2) cm^{-1}; ^{1}H NMR: (400 MHz, $CDCl_3$) 8.80 (d, J = 2.3 Hz, 1H, 7-H), 8.33 (dd, J = 8.6, 2.3 Hz, 1H, 5-H), 7.80 (d, J = 8.6 Hz, 1H, 4-H), 4.00 (s, 3H, OMe), 3.73 (s, 1H, 9-H); ^{13}C NMR: (100 MHz, $CDCl_3$) 164.2 (s, C-1), 146.9 (s, C-6), 136.0 (d, C-4), 133.7 (s, C-2), 128.9 (s, C-3), 126.0 (d, C-5), 125.5 (d, C-7), 87.9 (d, C-9), 80.3 (s, C-8), 52.8 (q, OMe); MS: (EI, 70 eV) m/z 205 (M, 100), 174 (81), 147 (51), 128 (72), 100 (49), 74 (41); HRMS: (CI, 70 eV) Calculated ($C_{10}H_8NO_4$) 206.0453 $[M + H]^+$ Found: 206.0451.

(**1c**) dimethyl 2-ethynylterephthalate

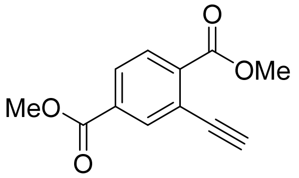

This compound was synthesized by a reported method[1] and the NMR data was agreement with the literature[2]. The spectral data of this compound is shown below.

(**1d**) methyl 2-ethynyl-5-methylbenzoate

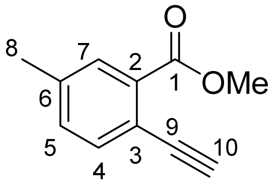

To a three necked flask, methyl 5-methyl-2-((trimethylsilyl)ethynyl)benzoate (7.96 g, 32.3 mmol), MeOH (30 mL) and K_2CO_3 (0.41 g, 3.0 mmol) were added. After stirring for 15 h at rt, the solvent was evaporated and the residue was diluted by CH_2Cl_2 (20 mL). The solution was washed by water (20 mL) and sat. NaCl aq. (20 mL). The organic layer was dried ($MgSO_4$) and the solvent was evaporated. The residue was purified by column chromatography (hexane/ethyl acetate = 80:20, column length 11 cm, diameter 26 mm, spherical silica gel) to give the product (2.33 g, 40%). IR: (KBr) 1720 (C=O) cm^{-1}; ^{1}H NMR: (400 MHz, $CDCl_3$) 7.74 (d, J = 1.4 Hz, 1H, 7-H), 7.50 (d, J = 7.7 Hz, 1H, 4-H), 7.26 (dd, J = 7.7, 1.4 Hz, 1H, 5-H), 3.91 (s, 3H, OMe), 3.36 (s, 1H, 10-H), 2.37 (s, 3H, 8-H_3); ^{13}C NMR: (100 MHz, $CDCl_3$) 166.4 (s, C-1), 138.6 (s, C-6), 134.6 (d, C-4), 132.4 (d, C-5), 132.1 (s, C-2), 130.7 (d, C-7), 119.5 (s, C-3), 82.0 (s, C-9), 81.2 (d, C-10), 51.9 (q, OMe), 21.1 (q, C-8); MS: (EI, 70 eV) m/z 174 (M, 94), 143 (92), 115 (100); HRMS: (CI, 70 eV) Calculated ($C_{11}H_{11}O_2$) 175.0759 $[M+H]^+$ Found: 175.0762.

(Precursor of **1e**) 4'-(*tert*-butyl)-4-((trimethylsilyl)ethynyl)-[1,1'-biphenyl]-3-carboxylate

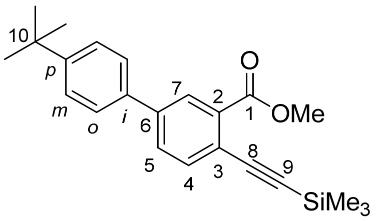

To a three necked flask, methyl 4'-(*tert*-butyl)-4-iodo-[1,1'-biphenyl]-3-carboxylate (1.20 g, 3.04 mmol), THF (4.5 mL), CuI (0.0128 g, 0.127 mmol), PPh_3 (0.0202 g, 0.0770 mmol), $PdCl_2(PPh_3)_2$ (0.101 g, 0.144 mmol), NEt_3 (0.475 g, 4.69 mmol) and trimethylsilylacetylene (0.45 g. 4.58 mmol) was added. After stirring for 57 h at rt, the reaction mixture was filtered

through a celite pad. The filtrate was evaporated and the residue was purified by column chromatography (hexane, column length 11 cm, diameter 26 mm, spherical silica gel) to give the product (0.984 g, 90%). IR: (KBr) 1736 (C=O) cm^{-1}; ^{1}H NMR: (400 MHz, $CDCl_3$) 8.16 (d, *J* = 1.9 Hz, 1H, 7-H), 7.66 (dd, *J* = 8.1, 1.9 Hz, 1H, 5-H), 7.62 (d, *J* = 8.1 Hz, 1H, 4-H), 7.55 (d, *J* = 8.7 Hz, 2H, *o*), 7.46 (d, *J* = 8.7 Hz, 2H, *m*), 3.94 (s, 3H, OMe). 1.35 (s, 9H, 10-Me_3), 0.289 (s, 9H, $SiMe_3$); ^{13}C NMR: (100 MHz, $CDCl_3$) 166.9 (s, C-1), 151.2 (s, *p*), 140.8 (s, C-6), 136.2 (s, *i*), 134.9 (d, C-4), 132.9 (s, C-2), 129.6 (d, C-5), 128.6 (d, C-7), 126.6 (d, *o*), 125.9 (d, *m*), 121.6 (s, C-3), 103.4 (s, C-8), 100.0 (s, C-9), 52.0 (q, OMe), 34.6 (s, C-10), 31.3 (q, 10-Me_3), -0.091 (q, $SiMe_3$); MS: (EI, 70 eV) m/z 364 (M, 89), 349 (93), 319 (100); HRMS: (EI, 70 eV) Calculated ($C_{23}H_{28}O_2Si$) 364.1859 $[M]^+$ Found: 364.1855.

(**1e**) methyl 4'-(*tert*-butyl)-4-ethynyl-[1,1'-biphenyl]-3-carboxylate

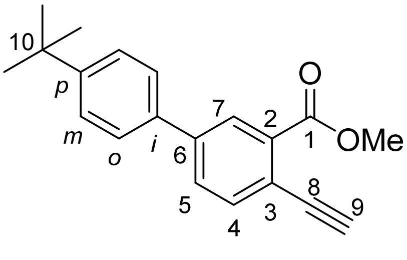

To a three necked flask, methyl 4'-(*tert*-butyl)-4-((trimethylsilyl)ethynyl)-[1,1'-biphenyl]-3-carboxylate (0.73 g, 2.00 mmol), MeOH (2.0 mL) and K_2CO_3 (0.028 g, 0.203 mmol) were added. After stirring for 18 h at rt, the solvent was evaporated and the residue was diluted by CH_2Cl_2 (5 mL). The solution was washed by water (5 mL) and sat. NaCl aq. (5 mL). The organic layer was dried ($MgSO_4$) and the solvent was evaporated. The residue was purified by column chromatography (hexane/ethyl acetate = 90:10, column length 11 cm, diameter 26 mm, spherical silica gel) to give the product (0.385 g, 65%). IR: (KBr) 1724 (C=O) cm^{-1};

^{1}H NMR: (400 MHz, $CDCl_3$) 8.18 (d, *J* = 1.9 Hz, 1H, 7-H), 7.70 (dd, *J* = 8.2, 1.9 Hz, 1H, 5-H), 7.67 (d, *J* = 8.2 Hz, 1H, 4-H), 7.56 (d, *J* = 8.7 Hz, 2H, *o*), 7.49 (d, *J* = 8.7 Hz, 2H, *m*), 3.96 (s, 3H, OMe). 3.43 (s, 1H, 9-H), 1.36 (s, 9H, 10-Me_3); ^{13}C NMR: (100 MHz, $CDCl_3$) 166.5 (s, C-1), 151.4 (s, *p*), 141.2 (s, C-6), 136.2 (s, *i*), 135.4 (d, C-4), 132.9 (s, C-2), 129.9 (d, C-5), 128.7 (d, C-7), 126.7 (d, *o*), 125.9 (d, *m*), 121.0 (s, C-3), 82.5 (d, C-9), 82.1 (s, C-8), 52.2 (q, OMe), 34.6 (s, C-10), 31.3 (q, 10-Me_3); MS: (EI, 70 eV) m/z 292 (M, 61), 277 (100); HRMS: (EI, 70 eV) Calculated ($C_{20}H_{20}O_2$) 292.1463 $[M]^+$ Found: 292.1463.

(**1f**) methyl 5-bromo-2-ethynylbenzoate

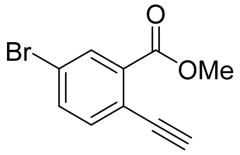

This compound was synthesized by a reported method[1] and the NMR data was agreement with the literature[3]. The spectral data of this compound is shown below.

(**1g**) methyl 5-chloro-2-ethynylbenzoate

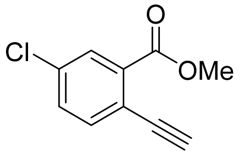

This compound was synthesized by a reported method[1] and the NMR data was agreement with the literature[4]. The spectral data of this compound is shown below.

(**1h**) methyl 2-ethynyl-5-fluorobenzoate

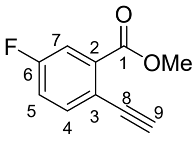

To a three necked flask, methyl 5-fluoro-2-((trimethylsilyl)ethynyl)benzoate (1.05 g, 4.20 mmol), MeOH (4.2 mL) and K_2CO_3 (0.063 g, 0.457 mmol) were added. After stirring for 5 h at rt, the solvent was evaporated and the residue was diluted by CH_2Cl_2 (10 mL). The solution was washed by water (10 mL) and sat. NaCl aq. (10 mL). The organic layer was dried ($MgSO_4$) and the solvent was evaporated. The residue was purified by column chromatography (hexane/ethyl acetate = 80:20, column length 11 cm, diameter 26 mm, spherical silica gel) to give the product (0.502 g, 67%). IR: (KBr) 1724 (C=O) cm^{-1}; ^1H NMR: (400 MHz, $CDCl_3$) 7.67-7.56 (m, 2H, 4-H and 7-H), 7.19 (td, J^3_{HH} = 8.2 Hz, J^3_{HF} = 8.2 Hz, J^4_{HH} = 2.9 Hz 1H, 5-H), 3.93 (s, 3H, OMe), 3.40 (s, 1H, 9-H); ^{13}C NMR: (100 MHz, $CDCl_3$) 165.0 (s, C-1), 161.7 (d, J = 250.7 Hz, C-6), 136.7 (d, J = 8.2 Hz, C-4), 134.3 (d, J = 8.2 Hz, C-2), 119.1 (d, J = 22.1, C-5), 118.6 (d, J = 4.1 Hz, C-3), 117.2 (d, J = 23.8 Hz, C-7), 82.0 (d, C-9), 80.8 (s, C-8), 52.3 (q, OMe); MS: (EI, 70 eV) m/z 178 (M, 84), 147 (100), 119 (95); HRMS: (CI, 70 eV) Calculated ($C_{10}H_8O_2F$) 179.0508 $[M + H]^+$ Found: 179.0511.

(**1i**) methyl 2-(phenylethynyl)benzoate

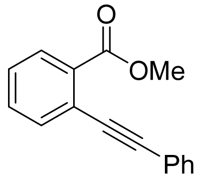

This compound was synthesized by a reported method[1] and the NMR data was agreement with the literature[5]. The spectral data of this compound is shown below.

(**1j**) methyl 2-((4-fluorophenyl)ethynyl)benzoate

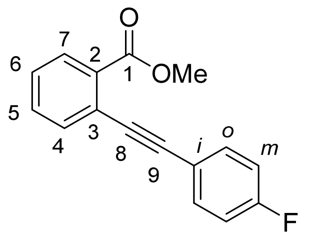

To a three necked flask, methyl 2-iodebenzoate (1.30 g, 4.96 mmol), NEt_3 (5 mL), $PdCl_2(PPh_3)_2$ (0.04 g, 0.057 mmol), CuI (0.050 g, 0.50 mmol) was added and 1-ethynyl-4-fluorobenzene (0.70 g. 5.83 mmol) was dropwised at 0 °C. After stirring for 24 h at rt, the reaction mixture was filtered through a celite pad. The filtrate was evaporated and the residue was purified by column chromatography (hexane/ethyl acetate = 90:10, column length 11 cm, diameter 26 mm, spherical silica gel) to give the product (0.70 g, 55%). IR: (neat) 1732 (C=O) cm^{-1}; ^1H NMR: (400 MHz, $CDCl_3$) 7.97 (dd, J = 8.2, 1.4 Hz, 1H, 7-H), 7.62 (dd, J = 7.7, 1.4 Hz, 1H, 4-H), 7.56 (dd, J = 9.1, 5.4 Hz, 2H, *o*), 7.48 (td, J = 7.7, 1.4 Hz, 1H, 5-H), 7.37 (td, J = 7.7, 1.4 Hz, 1H, 6-H), 7.05 (dd, J = 9.1, 8.6 Hz, 2H, *m*). 3.95 (s, 3H, OMe); ^{13}C NMR: (100 MHz, $CDCl_3$) 166.5 (s, C-1), 162.6 (s, d, $^1J_{CF}$ = 250 Hz, *p*), 133.8 (d), 133.6 (d), 133.5 (d), 131.7 (d, C-5), 131.6 (s, C-2), 130.4 (d, C-7), 127.9 (d, C-6), 123.5 (s, C-3), 119.3 (s, d, $^4J_{CF}$ = 2.9 Hz, *i*), 115.6 (d, d, $^2J_{CF}$ = 21.9 Hz, *m*), 93.2 (s, C-8), 87.9 (s, C-9), 52.1 (q, OMe); MS: (EI, 70 eV) m/z 254 (M, 100), 239 (87), 211 (42), 183 (40); HRMS: (EI, 70 eV) Calculated ($C_{20}H_{20}O_2$) 254.0743 $[M]^+$ Found: 254.0743.

(**1k**) methyl 2-(hex-1-yn-1-yl)benzoate

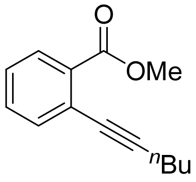

This compound was synthesized by a reported method[1] and the NMR data was agreement with the literature[6]. The spectral data of this compound is shown below.

(**1l**) ethyl 2-ethynylbenzoate

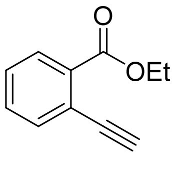

This compound was synthesized by a reported method[2] and the NMR data was agreement with the literature[7]. The spectral data of this compound is shown below.

(**1m**) isopropyl 2-ethynylbenzoate

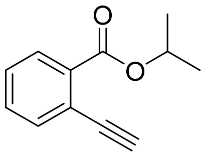

This compound was synthesized by a reported method[1] and the NMR data was agreement with the literature[8]. The spectral data of this compound is shown below.

Typical Procedures

Oxymetalation of methyl 2-alkynylbenzoate followed by protonolysis (Table 1 and Scheme 7)

In glove box filled for nitrogen, to a sealed vial, InI_3 (0.5 mmol), toluene (1 mL) and methyl 2-ethynylbenzoate **1a** (0.5 mmol) were added. The solution was stirred at 50 °C and the reaction mixture was quenched by deuterated acetic acid (1 mL) or water. After addition of water (10 mL), the solution was extracted with CH_2Cl_2 (5 mL x 3). The collected organic layer was dried ($MgSO_4$). In the investigation shown in Table 1, the solvent was evaporated and the yield of deuterated isocoumarin **2** was determined by ^{1}H NMR (400 MHz, in $CDCl_3$). In Scheme 7, the solvent was evaporated and the residue was purified by column chromatography.

Oxymetalation of 2-alkynylbenzoate and quantitation of alkyl halide (Table S1)

In glove box filled for nitrogen, to a sealed vial, InI_3 (0.5 mmol), toluene-d_8 (1 mL) and 2-ethynylbenzoate **1** (0.5 mmol) were added. After stirring at 50 °C for 1 h or 3 h, internal standard ($CHBr_3$) was added to the reaction mixture in glove box filled for nitrogen and then the yield of alkyl halide and the conversion of 2-ethynylbenzoate was determined by ^{1}H-NMR.

Oxymetalation of 2-alkynylbenzoate followed by halogenation (Table 2)

In glove box filled for nitrogen, to a sealed vial, InI_3 (0.5 mmol), toluene (1 mL) and methyl 2-alkynylbenzoate **1** (0.5 mmol) were added. After stirring at 50 °C for 24 h, the slurry was diluted by Et_2O (2 mL) and $PhI(OAc)_2$ (1.0 mmol) was added to the solution in glove box filled for nitrogen. The reaction mixture was stirred at rt for 12 h and then quenched by 1 N HCl aq. (5 mL) and CH_2Cl_2 (2 mL). The solution was extracted with CH_2Cl_2 (2 mL x 3) and the collected organic layer was dried ($MgSO_4$). The solvent was evaporated and the residue was purified by column chromatography.

Oxymetalation of 2-alkynylbenzoate followed by palladium catalyzed cross coupling (Table 3)

In glove box filled for nitrogen, to a sealed vial, $InBr_3$ (0.5 mmol), toluene (1 mL) and methyl 2-ethynylbenzoate **1a** (0.5 mmol) were added. After stirring at 50 °C for 24 h, Pd_2dba_3 (0.025 mmol), LiCl (1.0 mmol), organohalide **27** (1.0 mmol) and solvent (NMP or HMPA, 2.5 mL)

was added to the slurry in glove box filled for nitrogen. The mixture was stirred at rt or 50 °C for 24 h and then quenched by water (10 mL) and Et_2O (5 mL). The solution was extracted with Et_2O (10 mL x 3) and the collected organic layer was dried ($MgSO_4$). The solvent was evaporated and the residue was purified by column chromatography.

Monitoring of Oxyindation by ^{1}H NMR Spectroscopy

(1) at - 30 °C

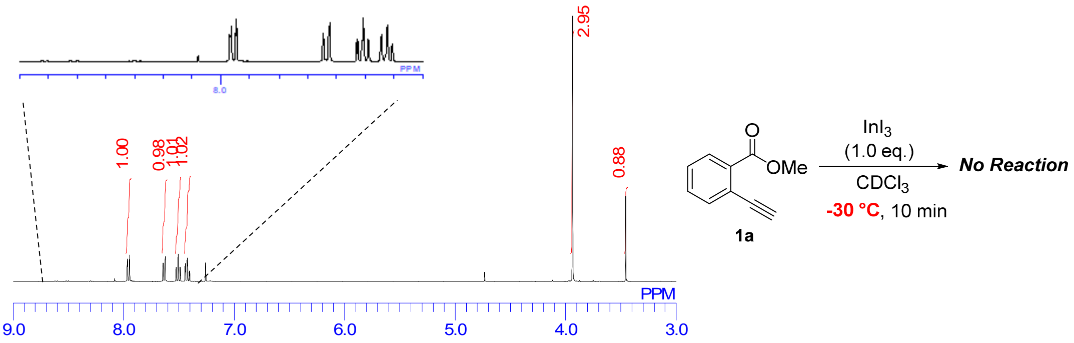

Fig. S1 Monitoring of oxyindation at - 30 °C using ^{1}H NMR (400 MHz, in $CDCl_3$) spectroscopy

When methyl 2-alkynylbenzoate **1a** was mixed with InI_3 in $CDCl_3$ at - 30 °C, no reaction occurred.

(2) at - 5 °C

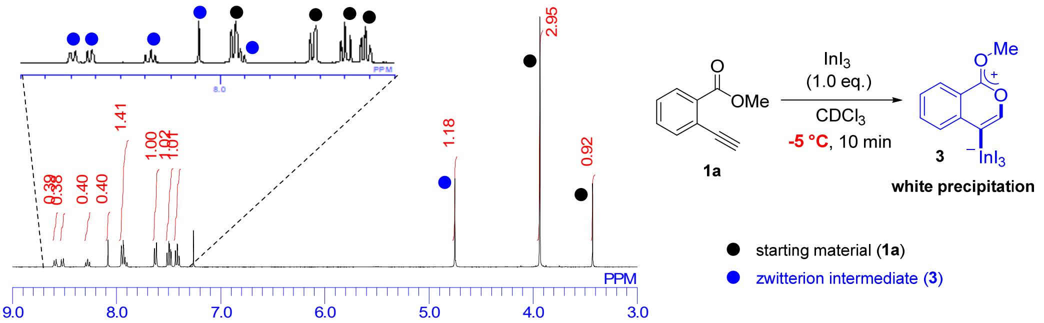

Fig. S2 Monitoring of oxyindation at - 5 °C using ^{1}H NMR (400 MHz, in $CDCl_3$) spectroscopy

A small amount of zwitterion intermediate **3** was observed.

The Effect of *O*-alkyl Groups on the Elimination of Alkyl Halide

As shown in Eq. 2, elimination of alkyl halide would be rate determining step. Accordingly, the reactivity of *O*-alkyl esters toward the elimination step was compared. Oxyindation of three types of alkyl esters was conducted in a short reaction time and the yields of alkyl halides and conversions of 2-alkynylbenzoate **1** were investigated by ^{1}H NMR (Table S1). The yield of zwitterion intermediate cannot be determined due to its insolubility to toluene. In all cases, fast conversion of **1** and formation of zwitterion species were observed. Methyl ester **1a** and ethyl ester **1l** gave MeI and EtI in 40% and 10% yields, respectively (entries 1 and 2). No alkyl halide was obtained in the case of isopropyl ester **1m** in spite of long reaction time (entry 3). These results indicated that the steric hindrance of *O*-alkyl group inhibited the elimination of alkyl halide.

Table S1 The effect of *O*-alkyl groups on the elimination of alkyl halide[a]

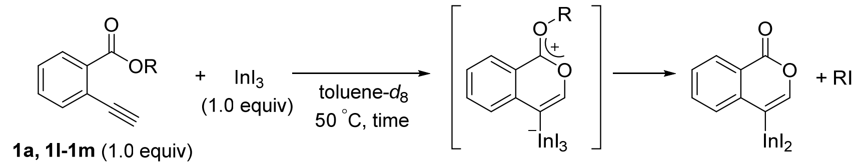

entry	R	time	NMR Yield (%) of RI	Conversion (%) of **1**
1	Me (**1a**)	1 h	40	94
2	Et (**1l**)	1 h	10	83
3	^{i}Pr (**1m**)	3 h	0	100

[a]**1** (0.5 mmol), InI_3 (0.5 mmol), toluene-d_8 (1 mL), 50 °C.

Formal Total Synthesis of Oosponol via Methyl 2-Ethynyl-6-Methoxybenzoate

We transformed **30** into methyl 2-ethynyl-6-methoxybenzoate **A** with traditional methods and tried to synthesize the precursor of oosponol **B** by oxyindation and cross-coupling (Scheme S1). However, the oxyindation gave complicated mixture and no starting material was observed due to some side reactions ascribing to the strong electron donating ability of OMe group presumably.

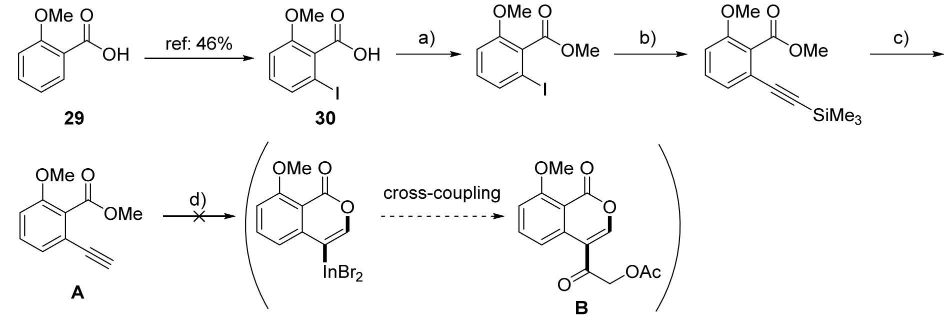

Scheme S1 Formal total synthesis of oosponol. Reagent and reaction conditions: a) (i) oxalyl chloride (2.0 equiv.), DMF (10 drops), CH_2Cl_2, RT, 3 h. (ii) MeOH, 100%. b) Ethynyltrimethylsilane (3.0 equiv.), $PdCl_2(PPh_3)_2$ (5.0 mol %), CuI (2.5 mol %), PPh_3 (2.5 mol %), NEt_3 (1.5 equiv.), THF, 80 °C, 19 h, 38%. c) K_2CO_3 (10 mol %.), MeOH, RT, 2 h, 80%. d) $InBr_3$ (1.0 equiv.), Toluene, 50 °C, 15 h. 0%.

Product

(**2**) Isocoumarin (Scheme 7)

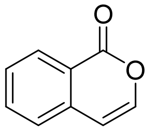

A two necked flask was dried over and filled with nitrogen. To this flask, the InI_3 (4.98 g, 10.0 mmol), toluene (20 mL) and methyl 2-ethynylbenzoate **1a** (1.60 g, 10.0 mmol) were added. The solution was stirred at 50 °C for 21 h and the reaction mixture was quenched by water (30 mL). This solution was extracted with CH_2Cl_2 (30 mL x 3) and the collected organic layer was dried ($MgSO_4$). The solvent was evaporated and the residue was purified by column chromatography (hexane/ethyl acetate = 90:10, column length 11 cm, diameter 26 mm, spherical silica gel) to give the product (1.14 g, 78%). The NMR data was agreement with the literature[9]. The spectral data of this compound is shown below.

(**24a**) 4-iodo-1*H*-isochromen-1-one

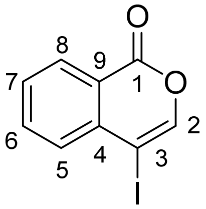

To a solution of InI_3 (0.255 g, 0.515 mmol) in toluene (1 mL), methyl 2-ethynylbenzoate (0.0824 g, 0.514 mmol) was added. The mixture was stirred at 50 °C for 24 h. Et_2O (1 mL) and $PhI(OAc)_2$ (0.32 g, 0.993 mmol) was added to the reaction mixture at rt. After stirring at rt for 12 h, the mixture was quenched by CH_2Cl_2 (2 mL) and 1 N HCl aq. (5 mL), and then extracted with CH_2Cl_2 (2 mL x 3). The collected organic layer was dried ($MgSO_4$). The solvent was evaporated and the residue was purified by column chromatography (hexane/ethyl acetate = 90:10, column length 11 cm, diameter 26 mm, spherical silica gel) to give the product (0.090 g, 64%). IR: (KBr) 1718 (C=O) cm^{-1}; ^1H NMR: (400 MHz, $CDCl_3$) 8.26 (d, J = 7.7 Hz, 1H, 8-H), 7.82 (t, J = 7.6 Hz, 1H, 6-H), 7.59 (m, 3H, 2-H, 5-H, 7-H); ^{13}C NMR: (100 MHz, $CDCl_3$) 161.0 (s, C-1), 147.4 (d, C-2), 136.3 (s, C-4), 135.6 (d, C-8), 130.1 (d), 130.0 (d), 129.7 (d), 121.9 (s, C-9), 75.9 (s, C-3); MS: (EI, 70 eV) *m/z* 272 (M^+, 100), 244 (89), 89 (97), 63 (26); HRMS: (CI, 70 eV) Calculated ($C_9H_6IO_2$) 272.9412 $[M + H]^+$ Found 272.9415.

(**25a**) 4-bromo-1*H*-isochromen-1-one

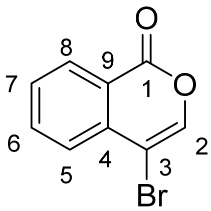

To a solution of InI_3 (0.255 g, 0.515 mmol) in toluene (1 mL), methyl 2-ethynylbenzoate (0.0820 g, 0.512 mmol) was added. The mixture was stirred at 50 °C for 24 h. Et_2O (1 mL) and $PhI(OAc)_2$ (0.335 g, 1.04 mmol) was added to the reaction mixture at rt. After stirring at rt for 12 h, the mixture was quenched by CH_2Cl_2 (2 mL) and 1 N HCl aq. (5 mL), and then extracted with CH_2Cl_2 (2 mL x 3). The collected organic layer was dried ($MgSO_4$). The solvent was evaporated and the residue was purified by column chromatography (hexane/ethyl acetate = 90:10, column length 11 cm, diameter 26 mm, spherical silica gel) to give the product (0.0845 g, 73%). IR : (KBr) 1736 (C=O) cm^{-1}; ^1H NMR: (400 MHz, $CDCl_3$) 8.31 (d, J = 7.5 Hz, 1H, 8-H), 7.85 (t, J = 7.5 Hz, 1H, 6-H), 7.74 (d, J = 7.5 Hz, 1H, 5-H), 7.62 (t, J = 7.5 Hz, 1H, 7-H), 7.51 (s, 1H, 2-H); ^{13}C NMR: (100 MHz, $CDCl_3$) 160.8 (s, C-1), 143.0 (d, C-2), 135.4 (d, C-6), 135.0 (s, C-4), 130.1 (d) 130.0 (d) 125.8 (d, C-5), 121.6 (s, C-9) 103.6 (s, C-3); MS: (EI, 70 eV) *m/z* 224 (M^+, 55), 226 ($M^+ + 2$, 54), 196 (59), 89 (100), 63 (22); HRMS: (CI, 70 eV) Calculated ($C_9H_6BrO_2$) 224.9551 $[M + H]^+$ Found 224.9548.

(**24b**) 4-iodo-7-nitro-1*H*-isochromen-1-one

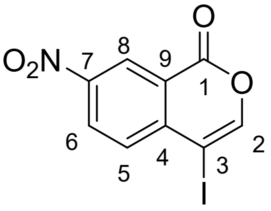

To a solution of InI_3 (0.248 g, 0.500 mmol) in toluene (1 mL), methyl 2-ethynyl-5-nitrobenzoate (0.105 g, 0.512 mmol) was added. The mixture was stirred at 50 °C for 24 h. Et_2O (1 mL) and $PhI(OAc)_2$ (0.350 g, 1.09 mmol) was added to the reaction mixture at rt. After stirring at rt for 12 h, the mixture was quenched by CH_2Cl_2 (2 mL) and 1 N HCl aq. (5 mL), and then extracted with CH_2Cl_2 (2 mL x 3). The collected organic layer was dried

($MgSO_4$). The solvent was evaporated and the residue was purified by column chromatography (hexane/ethyl acetate = 80:20, column length 11 cm, diameter 26 mm, spherical silica gel) to give the product (0.0978 g, 61%). This product was recrystallized from CH_2Cl_2 and hexane to give a single crystal and the structure was determined by X-ray crystallographic analysis (CCDC 1576343). IR: (KBr) 1720 (C=O) cm^{-1}, 1342 (NO_2) cm^{-1}; ^{1}H NMR: (400 MHz, $CDCl_3$) 9.10 (d, J = 2.7 Hz, 1H, 8-H), 8.61 (dd, J = 9.2, 2.7 Hz, 1H, 6-H), 7.84 (d, J = 9.2 Hz, 1H, 5-H), 7.76 (s, 1H, 2-H); ^{13}C NMR: (100 MHz, $CDCl_3$) 159.1 (s, C-1), 150.4 (d, C-2), 147.8 (s, C-7), 141.3 (s, C-4), 132.0 (d, C-5), 129.5 (d, C-6), 125.7 (d, C-8), 122.5 (s, C-9), 73.6 (s, C-3); MS: (EI, 70 eV) m/z 317 (M, 100), 289 (68); HRMS: (CI, 70 eV) Calculated ($C_9H_5NO_4I$) 317.9263 $[M + H]^+$ Found: 317.9263.

(**24c**) methyl 4-iodo-1-oxo-1*H*-isochromene-6-carboxylate

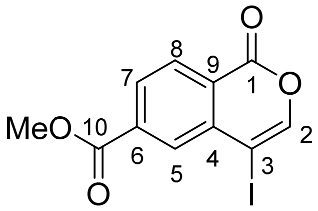

To a solution of InI_3 (0.255 g, 0.515 mmol) in toluene (1 mL), dimethyl 2-ethynylterephthalate (0.11 g, 0.504 mmol) was added. The mixture was stirred at 50 °C for 24 h. Et_2O (1 mL) and $PhI(OAc)_2$ (0.330 g, 1.02 mmol) was added to the reaction mixture at rt. After stirring at rt for 12 h, the mixture was quenched by CH_2Cl_2 (2 mL) and water (5 mL), and then extracted with CH_2Cl_2 (2 mL x 3). The collected organic layer was dried ($MgSO_4$). The solvent was evaporated and the residue was purified by column chromatography (hexane/ethyl acetate = 90:10, column length 11 cm, diameter 26 mm, spherical silica gel) to give the product (0.1171 g, 70%). IR: (KBr) 1736 (C=O) cm^{-1}; ^{1}H NMR: (400 MHz, $CDCl_3$)

8.35 (d, J = 8.0 Hz, 1H, 8-H), 8.27 (d, J = 1.2 Hz, 1H, 5-H), 8.20 (dd, J = 8.0, 1.2 Hz, 1H, 7-H), 7.64 (s, 1H, 2-H), 4.02 (s, 3H, OMe); ^{13}C NMR: (100 MHz, $CDCl_3$) 165.3 (s, C-10), 160.3 (s, C-1), 148.1 (d, C-2), 136.7 (s), 136.4 (s), 131.6 (d, C-5), 130.5 (d, C-8), 130.0 (d, C-7), 125.0 (s), 75.2 (s, C-3), 52.9 (q, OMe); MS: (EI, 70 eV) m/z 330 (M, 100), 302 (71), 147 (82); HRMS: (CI, 70 eV) Calculated ($C_{11}H_8O_4I$) 330.9467 $[M + H]^+$ Found: 330.9469.

(**24d**) 4-iodo-7-methyl-1*H*-isochromen-1-one

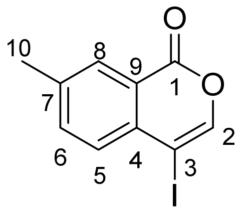

To a solution of InI_3 (0.260 g, 0.525 mmol) in toluene (1 mL), methyl 2-ethynyl-5-methylbenzoate (0.087 g, 0.500 mmol) was added. The mixture was stirred at 50 °C for 24 h. Et_2O (1 mL) and $PhI(OAc)_2$ (0.335 g, 1.04 mmol) was added to the reaction mixture at rt. After stirring at rt for 12 h, the mixture was quenched by CH_2Cl_2 (2 mL) and 1 N HCl aq. (5 mL), and then extracted with CH_2Cl_2 (2 mL x 3). The collected organic layer was dried ($MgSO_4$). The solvent was evaporated and the residue was purified by column chromatography (hexane/ethyl acetate = 90:10, column length 11 cm, diameter 26 mm, spherical silica gel) to give the product (0.0774 g, 54%). IR: (KBr) 1751 (C=O) cm^{-1}; ^{1}H NMR: (400 MHz, $CDCl_3$) 8.07 (s, 1H, 8-H), 7.61 (d, J = 8.2 Hz, 1H, 6-H), 7.54 (s, 1H, 2-H), 7.48 (d, J = 8.2 Hz, 1H, 5-H), 2.50 (s, 3H, 10-H); ^{13}C NMR: (100 MHz, $CDCl_3$) 161.3 (s, C-1), 146.6 (d, C-2), 140.3 (s, C-7), 136.7 (d, C-6), 134.0 (s, C-4), 130.0 (d), 129.8 (d), 121.8

(s, C-9), 75.9 (s, C-3), 21.2 (q, C-10); MS: (EI, 70 eV) m/z 286 (M, 100), 258 (66), 103 (88); HRMS: (CI, 70 eV) Calculated ($C_{10}H_8O_2I$) 286.9569 $[M + H]^+$ Found: 286.9567.

(**24e**) 7-(4-(*tert*-butyl)phenyl)-4-iodo-1*H*-isochromen-1-one

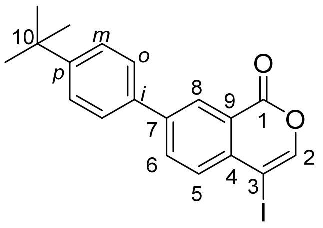

To a solution of InI_3 (0.259 g, 0.523 mmol) in toluene (1 mL), methyl 4'-(*tert*-butyl)-4-ethynyl-[1,1'-biphenyl]-3-carboxylate (0.1453 g, 0.497 mmol) was added. The mixture was stirred at 50 °C for 24 h. Et_2O (1 mL) and $PhI(OAc)_2$ (0.335 g, 1.04 mmol) was added to the reaction mixture at rt. After stirring at rt for 12 h, the mixture was quenched by CH_2Cl_2 (2 mL) and 1 N HCl aq. (5 mL), and then extracted with CH_2Cl_2 (2 mL x 3). The collected organic layer was dried ($MgSO_4$). The solvent was evaporated and the residue was purified by column chromatography (hexane/ethyl acetate = 90:10, column length 11 cm, diameter 26 mm, spherical silica gel) to give the product (0.109 g, 54%). IR: (KBr) 1728 (C=O) cm^{-1}; ^1H NMR: (400 MHz, $CDCl_3$) 8.51 (d, J = 1.9 Hz, 1H, 8-H), 8.05 (dd, J = 8.5, 1.9 Hz, 1H, 6-H), 7.67 (d, J = 8.5 Hz, 1H, 5-H), 7.64 (d, J = 8.7 Hz, 2H, *o*), 7.60 (s, 1H, 2-H), 7.53 (d, J = 8.7 Hz, 2H, *m*); ^{13}C NMR: (100 MHz, $CDCl_3$) 161.3 (s, C-1), 151.7 (s, *p*), 147.1 (d, C-2), 142.5 (s, C-7), 135.6 (s, *i*), 135.0 (s, C-4), 134.0 (d, C-6), 130.7 (d, C-5), 127.7 (d, C-8), 126.8 (d, *o*), 126.1 (d, *m*), 122.3 (s, C-9), 75.7 (s, C-3), 34.6 (s, C-10), 31.3 (q, 10-Me x 3); MS: (EI, 70 eV) m/z 404 (M, 56), 389 (100); HRMS: (CI, 70 eV) Calculated ($C_{19}H_{18}O_2I$) 405.0351 $[M + H]^+$ Found: 405.0352.

(**25f**) 4,7-dibromo-1*H*-isochromen-1-one

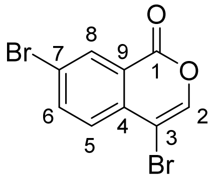

To a solution of $InBr_3$ (0.190 g, 0.536 mmol) in toluene (1 mL), methyl 5-bromo-2-ethynylbenzoate (0.120 g, 0.502 mmol) was added. The mixture was stirred at 50 °C for 24 h. Et_2O (1 mL) and $PhI(OAc)_2$ (0.322 g, 1.00 mmol) was added to the reaction mixture at rt. After stirring at rt for 12 h, the mixture was quenched by CH_2Cl_2 (2 mL) and 1 N HCl aq. (5 mL), and then extracted with CH_2Cl_2 (2 mL x 3). The collected organic layer was dried ($MgSO_4$). The solvent was evaporated and the residue was purified by column chromatography (hexane/ethyl acetate = 90:10, column length 11 cm, diameter 26 mm, spherical silica gel) to give the product (0.071 g, 47%). IR: (KBr) 1732 (C=O) $cm^{-1;}$ ^{1}H NMR: (400 MHz, $CDCl_3$) 8.43 (d, *J* = 1.8 Hz, 1H, 8-H), 7.94 (dd, *J* = 8.6, 1.8 Hz, 1H, 6-H), 7.62 (d, *J* = 8.6 Hz, 1H, 5-H), 7.53 (s, 1H, 2-H); ^{13}C NMR: (100 MHz, $CDCl_3$) 159.5 (s, C-1), 143.3 (d, C-2), 138.5 (d, C-6), 133.9 (s, C-4), 132.6 (d, C-8), 127.6 (d, C-5), 123.5 (s, C-7), 122.8 (s, C-9), 102.8 (s, C-3); MS: (EI, 70 eV) m/z 306 (M + 4, 39), 304 (M + 2, 100), 302 (M, 40), 169 (65), 167 (70); HRMS: (CI, 70 eV) Calculated ($C_9H_5Br_2O_2$) 302.8656 $[M+H]^+$ Found: 302.8654.

(**24g**) 7-chloro-4-iodo-1*H*-isochromen-1-one

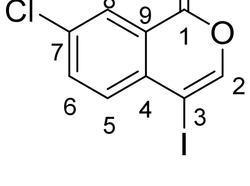

To a solution of InI_3 (0.256 g, 0.516 mmol) in toluene (1 mL), methyl 5-chloro-2-ethynylbenzoate (0.098 g, 0.505 mmol) was added. The mixture was stirred at 50 °C for 24 h. Et_2O (1 mL) and $PhI(OAc)_2$ (0.321 g, 0.997 mmol) was added to the reaction mixture at rt. After stirring at rt for 12 h, the mixture was quenched by CH_2Cl_2 (2 mL) and 1 N HCl aq. (5 mL), and then extracted with CH_2Cl_2 (2 mL x 3). The collected organic layer was dried ($MgSO_4$). The solvent was evaporated and the residue was purified by column chromatography (hexane/ethyl acetate = 90:10, column length 11 cm, diameter 26 mm, spherical silica gel) to give the product (0.1036 g, 67%). IR: (KBr) 1724 (C=O) $cm^{-1;}$ ^1H NMR: (400 MHz, $CDCl_3$) 8.24 (d, J = 2.3 Hz, 1H, 8-H), 7.75 (dd, J = 8.7, 2.3 Hz, 1H, 6-H), 7.59 (s, 1H, 2-H), 7.57 (d, J = 8.7 Hz, 1H, 5-H); ^{13}C NMR: (100 MHz, $CDCl_3$) 159.9 (s, C-1), 147.6 (d, C-2), 135.78 (d, C-6), 135.76 (s, C-7), 134.9 (s, C-4), 131.8 (d, C-5), 129.4 (d, C-8), 122.9 (s, C-9), 74.6 (s, C-3); MS: (EI, 70 eV) m/z 308 (M + 2, 35), 306 (M, 100), 278 (64), 123 (84); HRMS: (CI, 70 eV) Calculated ($C_9H_5ClO_2I$) 306.9023 $[M + H]^+$ Found: 306.9022.

(**24h**) 7-fluoro-4-iodo-1*H*-isochromen-1-one

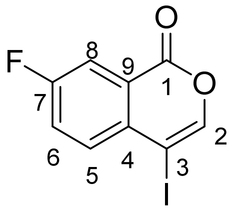

To a solution of InI_3 (0.255 g, 0.515 mmol) in toluene (1 mL), methyl 5-fluoro-2-ethynylbenzoate (0.0913 g, 0.512 mmol) was added. The mixture was stirred at 50 °C for 24 h. Et_2O (1 mL) and $PhI(OAc)_2$ (0.330 g, 1.02 mmol) was added to the reaction mixture at rt.

After stirring at rt for 12 h, the mixture was quenched by CH_2Cl_2 (2 mL) and 1 N HCl aq. (5 mL), and then extracted with CH_2Cl_2 (2 mL x 3). The collected organic layer was dried ($MgSO_4$). The solvent was evaporated and the residue was purified by column chromatography (hexane/ethyl acetate = 90:10, column length 11 cm, diameter 26 mm, spherical silica gel) to give the product (0.091 g, 61%). IR: (KBr) 1728 (C=O) cm^{-1}; ^{1}H NMR: (400 MHz, $CDCl_3$) 7.93 (d, $^3J_{HF}$ = 8.2 Hz, 1H, 8-H), 7.64 (dd, J = 8.9 Hz, $^4J_{HF}$ = 5.1 Hz, 1H, 5-H), 7.56 (s, 1H, 2-H), 7.52 (dd, J = 8.9 Hz, $^3J_{HF}$ = 8.2 Hz, 1H, 6-H); ^{13}C NMR: (100 MHz, $CDCl_3$) 162.6 (s, d, $^1J_{CF}$ = 252 Hz, C-7), 160.1 (s, C-1), 146.6 (d, C-2), 132.9 (s, d, $^4J_{CF}$ = 2.5 Hz, C-4), 132.7 (d, d, $^3J_{CF}$ = 7.4 Hz, C-5), 123.6 (d, d, $^2J_{CF}$ = 22.9 Hz, C-6), 123.3 (s, d, $^3J_{CF}$ = 8.2 Hz, C-9), 115.5 (d, d, $^2J_{CF}$ = 23.8 Hz, C-8), 74.5 (s, C-3); MS: (EI, 70 eV) m/z 290 (M, 96), 262 (55), 107 (100); HRMS: (CI, 70 eV) Calculated ($C_9H_5FO_2I$) 290.9318 $[M + H]^+$ Found: 290.9315.

(**24i**) 4-iodo-3-phenyl-1*H*-isochromen-1-one

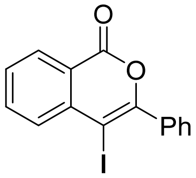

To a solution of GaI_3 (0.230 g, 0.511 mmol) in toluene (1 mL), methyl 2-(phenylethynyl)benzoate (0.116 g, 0.491 mmol) was added. The mixture was stirred at 50 °C for 24 h. Et_2O (2 mL) and $PhI(OAc)_2$ (0.322 g, 1.00 mmol) was added to the reaction mixture at rt. After stirring at rt for 12 h, the mixture was quenched by CH_2Cl_2 (2 mL) and 1 N HCl aq. (5 mL), and then extracted with CH_2Cl_2 (2 mL x 3). The collected organic layer was dried ($MgSO_4$). The solvent was evaporated and the residue was purified by column

chromatography (hexane/ethyl acetate = 90:10, column length 11 cm, diameter 26 mm, spherical silica gel) to give the product (0.103 g, 60%). The NMR data was agreement with the literature[6]. The spectral data of this compound is shown below.

(**24j**) 3-(4-fluorophenyl)-4-iodo-1*H*-isochromen-1-one

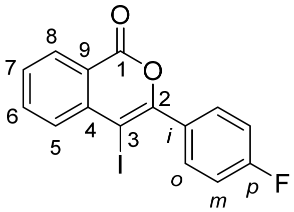

To a solution of GaI_3 (0.231 g, 0.513 mmol) in toluene (1 mL), methyl 2-((4-fluorophenyl)ethynyl)benzoate (0.129 g, 0.507 mmol) was added. The mixture was stirred at 50 °C for 24 h. Et_2O (2 mL) and $PhI(OAc)_2$ (0.330 g, 1.02 mmol) was added to the reaction mixture at rt. After stirring at rt for 12 h, the mixture was quenched by CH_2Cl_2 (2 mL) and 1 N HCl aq. (5 mL), and then extracted with CH_2Cl_2 (2 mL x 3). The collected organic layer was dried ($MgSO_4$). The solvent was evaporated and the residue was purified by column chromatography (hexane/ethyl acetate = 90:10, column length 11 cm, diameter 26 mm, spherical silica gel) to give the product (0.104 g, 56%). IR: (KBr) 1728 (C=O) cm^{-1}; ^1H NMR: (400 MHz, CD_2Cl_2) 8.13 (d, J = 8.2 Hz, 1H, 8-H), 7.77 (d, J = 8.2 Hz, 1H, 5-H), 7.72 (dd, J = 8.2, 7.7 Hz, 1H, 6-H), 7.60 (dd, J = 8.6 Hz, $^4J_{HF}$ = 5.4 Hz, 1H, *o*), 7.48 (dd, J = 8.2, 7.7 Hz, 1H, 7-H), 7.09 (dd, J = 8.6 Hz, $^3J_{HF}$ = 9.1 Hz, 1H, *m*); ^{13}C NMR: (100 MHz, CD_2Cl_2) 163.9 (s, d, $^1J_{CF}$ = 250 Hz, *p*), 161.5 (s, C-1), 154.1 (s, C-2), 138.3 (s, C-4), 136.1 (d, C-6), 132.6 (d, d, $^3J_{CF}$ = 8.6 Hz, *o*), 132.0 (s, d, $^4J_{CF}$ = 3.8 Hz, *i*), 131.9 (d, C-5), 129.9 (d, C-8), 129.8 (d, C-7), 120.6 (s. C-9), 115.6 (d, d, $^2J_{CF}$ = 11.4 Hz, *m*), 77.0 (s, C-3); MS: (EI, 70 eV) m/z 366

(M, 100), 338 (57), 211 (60), 183 (65), 123 (50), 95 (48); HRMS: (CI, 70 eV) Calculated ($C_{15}H_9FIO_2$) 366.9631 $[M + H]^+$ Found: 366.9630.

(**25k**) 4-bromo-3-butyl-1*H*-isochromen-1-one

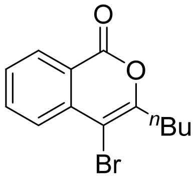

To a solution of $GaBr_3$ (0.169 g, 0.546 mmol) in toluene (1 mL), methyl 2-(hex-1-yn-1-yl)benzoate (0.110 g, 0.509 mmol) was added. The mixture was stirred at 50 °C for 24 h. Et_2O (2 mL) and $PhI(OAc)_2$ (0.322 g, 1.00 mmol) was added to the reaction mixture at rt. After stirring at rt for 12 h, the mixture was quenched by CH_2Cl_2 (2 mL) and 1 N HCl aq. (5 mL), and then extracted with CH_2Cl_2 (2 mL x 3). The collected organic layer was dried ($MgSO_4$). The solvent was evaporated and the residue was purified by column chromatography (hexane/ethyl acetate = 90:10, column length 11 cm, diameter 26 mm, spherical silica gel) to give the product (0.0852 g, 60%). The NMR data was agreement with the literature[10]. The spectral data of this compound is shown below.

(**28aa**) 4-phenyl-1*H*-isochromen-1-one

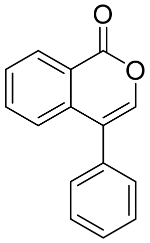

To a solution of $InBr_3$ (0.185 g, 0.522 mmol) in toluene (1 mL), methyl 2-ethynylbenzoate (0.082 g, 0.512 mmol) was added. The mixture was stirred at 50 °C for 24 h. Pd_2dba_3 (0.026 g, 0.0284 mmol), LiCl (0.044 g, 1.04 mmol), iodebenzene (0.255 g, 1.25 mmol) and NMP (2.5 mL) was added to the reaction mixture at rt. After stirring at 50 °C for 24 h, the mixture was quenched by Et_2O (5 mL) and water (10 mL), and then extracted with Et_2O (10 mL x 3). The collected organic layer was dried ($MgSO_4$). The solvent was evaporated and the residue was purified by column chromatography (hexane/ethyl acetate = 90:10, column length 11 cm, diameter 26 mm, spherical silica gel) to give the product (0.0922 g, 81%). The NMR data was agreement with the literature[11]. The spectral data of this compound is shown below.

(**28ab**) 4-(4-methoxyphenyl)-1*H*-isochromen-1-one

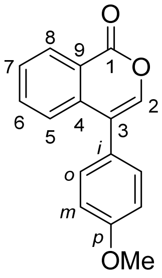

To a solution of $InBr_3$ (0.179 g, 0.505 mmol) in toluene (1 mL), methyl 2-ethynylbenzoate (0.083 g, 0.518 mmol) was added. The mixture was stirred at 50 °C for 24 h. Pd_2dba_3 (0.03 g, 0.033 mmol), LiCl (0.059 g, 1.39 mmol), 1-iodo-4-methoxybenzene (0.234 g, 1.0 mmol) and NMP (2.5 mL) was added to the reaction mixture at rt. After stirring at 50 °C for 24 h, the mixture was quenched by Et_2O (5 mL) and water (10 mL), and then extracted with Et_2O (10 mL x 3). The collected organic layer was dried ($MgSO_4$). The solvent was evaporated and the residue was purified by column chromatography (hexane/ethyl acetate = 90:10, column length 11 cm, diameter 26 mm, spherical silica gel) to give the product (0.0931 g,

71%). IR: (KBr) 1728 (C=O) cm^{-1}; ^1H NMR: (400 MHz, $CDCl_3$) 8.39 (d, J = 8.0 Hz, 1H, 8-H), 7.70 (t, J = 8.0 Hz, 1H, 6-H), 7.55 (t, J = 8.0 Hz, 1H, 7-H), 7.41 (d, J = 8.0 Hz, 1H, 5-H), 7.32 (d, J = 8.7 Hz, 2H, *o*), 7.23 (s, 1H, 2-H), 7.02 (d, J = 8.7 Hz, 2H, *m*); ^{13}C NMR: (100 MHz, $CDCl_3$) 162.1 (s, C-1), 159.7 (s, *p*), 142.0 (d, C-2), 137.1 (s, C-4), 134.6 (d, C-6), 130.9 (d, *o*), 130.0 (d, C-8), 128.4 (d, C-7), 125.1 (s, *i*), 124.6 (d, C-5), 121.3 (s, C-9), 120.2 (s, C-3), 114.2 (d, *m*), 55.3 (q, OMe); MS: (EI, 70 eV) m/z 252 (M, 100), 224 (36), 181 (31), 152 (33); HRMS: (CI, 70 eV) Calculated ($C_{16}H_{13}O_3$) 253.0865 $[M + H]^+$ Found: 253.0861.

(**28ac**) 4-(1-oxo-1*H*-isochromen-4-yl)benzonitrile

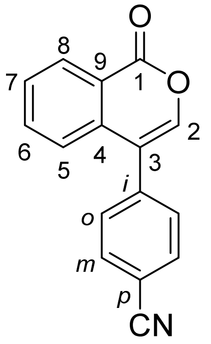

To a solution of $InBr_3$ (0.188 g, 0.530 mmol) in toluene (1 mL), methyl 2-ethynylbenzoate (0.081 g, 0.506 mmol) was added. The mixture was stirred at 50 °C for 24 h. Pd_2dba_3 (0.026 g, 0.0284 mmol), LiCl (0.0511 g, 1.21 mmol), 1-iodo-4-cyanobenzene (0.250 g, 1.09 mmol) and NMP (2.5 mL) was added to the reaction mixture at rt. After stirring at 50 °C for 24 h, the mixture was quenched by Et_2O (5 mL) and water (10 mL), and then extracted with Et_2O (10 mL x 3). The collected organic layer was dried ($MgSO_4$). The solvent was evaporated and the residue was purified by column chromatography (hexane/ethyl acetate = 90:10, column length 11 cm, diameter 26 mm, spherical silica gel) to give the product (0.090 g,

72%). IR: (KBr) 2225 (CN) cm^{-1}, 1720 (C=O) cm^{-1}; ^{1}H NMR: (400 MHz, $CDCl_3$) 8.42 (d, *J* = 7.8 Hz, 1H, 8-H), 7.81 (d, *J* = 7.3 Hz, 2H, *o*), 7.74 (t, *J* = 7.8 Hz, 1H, 6-H), 7.61 (t, *J* = 7.8 Hz, 1H, 7-H), 7.56 (d, *J* = 7.3 Hz, 2H, *m*), 7.33 (d, *J* = 7.8 Hz, 1H, 5-H), 7.30 (s, 1H, 2-H); ^{13}C NMR: (100 MHz, $CDCl_3$) 161.4 (s, C-1), 142.8 (d, C-2), 138.1 (s, C-3), 135.6 (s, C-4), 135.0 (d, C-6), 132.7 (d, *o*), 130.5 (d, *m*), 130.4 (d, C-8), 129.1 (d, C-7), 124.0 (d, C-5), 121.3 (s, C-9), 119.3 (s, *i*), 118.3 (s, *p*), 112.5 (s, CN); MS: (EI, 70 eV) m/z 247 (M, 63), 219 (100), 190 (66); HRMS: (CI, 70 eV) Calculated ($C_{16}H_{10}NO_2$) 248.0712 $[M + H]^+$ Found: 248.0710.

(**28ad**) 4-benzoyl-1*H*-isochromen-1-one

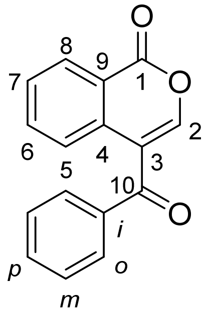

To a solution of $InBr_3$ (0.180 g, 0.508 mmol) in toluene (1 mL), methyl 2-ethynylbenzoate (0.0824 g, 0.514 mmol) was added. The mixture was stirred at 50 °C for 24 h. Pd_2dba_3 (0.027 g, 0.0295 mmol), LiCl (0.052 g, 1.23 mmol), benzoyl chloride (0.142 g, 1.01 mmol) and NMP (2.5 mL) was added to the reaction mixture at rt. After stirring at 50 °C for 24 h, the mixture was quenched by Et_2O (5 mL) and water (10 mL), and then extracted with Et_2O (10 mL x 3). The collected organic layer was dried ($MgSO_4$). The solvent was evaporated and the residue was purified by column chromatography (hexane/ethyl acetate = 90:10, column length 11 cm, diameter 26 mm, spherical silica gel) to give the product (0.1058 g, 82%). IR: (KBr) 1735 (C=O) cm^{-1}; ^{1}H NMR: (400 MHz, $CDCl_3$) 8.39 (d, *J* = 8.0 Hz, 1H, 8-H), 8.16 (d,

J = 8.2 Hz, 1H, 5-H), 7.88 (d, J = 8.5 Hz, 2H, *o*), 7.82 (t, J = 8.2 Hz, 1H, 6-H), 7.67 (s, 1H, 2-H), 7.69-7.60 (m, 2H, *p* and 7-H), 7.53 (t, J = 8.0 Hz, 2H, *m*); ^{13}C NMR: (100 MHz, $CDCl_3$) 192.4 (s, C-10), 160.5 (s, C-1), 151.7 (d, C-2), 137.6 (s, *i*), 135.3 (d, C-6), 133.9 (s, C-4), 133.5 (d), 129.9 (d, C-8), 129.8 (d, *o*), 129.3 (d), 128.7 (d, *m*), 125.2 (d, C-5), 120.7 (s, C-9), 117.7 (s, C-3); MS: (EI, 70 eV) m/z 250 (M, 97), 249 (100), 105 (78), 77 (52); HRMS: (CI, 70 eV) Calculated ($C_{16}H_{11}O_3$) 251.0708 $[M + H]^+$ Found: 251.0709.

(**28ae**) 4-(4-chlorobenzoyl)-1*H*-isochromen-1-one

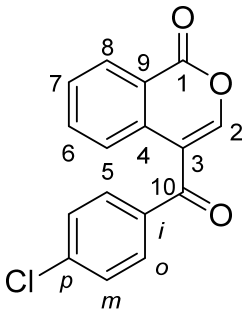

To a solution of $InBr_3$ (0.179 g, 0.505 mmol) in toluene (1 mL), methyl 2-ethynylbenzoate (0.080 g, 0.499 mmol) was added. The mixture was stirred at 50 °C for 24 h. Pd_2dba_3 (0.026 g, 0.0284 mmol), LiCl (0.049 g, 1.16 mmol), 4-chlorobenzoyl chloride (0.180 g, 1.03 mmol) and NMP (2.5 mL) was added to the reaction mixture at rt. After stirring at 50 °C for 24 h, the mixture was quenched by Et_2O (5 mL) and water (10 mL), and then extracted with Et_2O (10 mL x 3). The collected organic layer was dried ($MgSO_4$). The solvent was evaporated and the residue was purified by column chromatography (hexane/ethyl acetate = 90:10, column length 11 cm, diameter 26 mm, spherical silica gel) to give the product (0.091 g, 64%). This product was recrystallized from CH_2Cl_2 to give a single crystal and the structure was determined by X-ray crystallographic analysis (CCDC 1576344). IR: (KBr) 1763 (C=O)

cm^{-1}; ^{1}H NMR: (400 MHz, $CDCl_3$) 8.37 (d, J = 8.0 Hz, 1H, 8-H), 8.10 (d, J = 8.0 Hz, 1H, 5-H), 7.86-7.77 (m, 3H, *o* and 6-H), 7.65 (s, 1H, 2-H), 7.63 (t, J = 8.0 Hz, 1H, 7-H), 7.50 (d, J = 8.7 Hz, 2H, *m*); ^{13}C NMR: (100 MHz, $CDCl_3$) 191.2 (s, C-10), 160.3 (s, C-1), 151.5 (d, C-2), 140.1 (s), 136.0 (s), 135.4 (d, C-6), 133.7 (s, C-4), 131.2 (d, *o*), 130.0 (d, C-8), 129.5 (d, C-7), 129.1 (d, *m*), 125.2 (d, C-5), 120.8 (s, C-9), 117.6 (s, C-3); MS: (EI, 70 eV) m/z 286 (M +2, 32), 284 (M, 93), 283 (49), 249 (40), 139 (100), 111 (55); HRMS: (CI, 70 eV) Calculated ($C_{16}H_{10}ClO_3$) 285.0318 [M + H]$^+$ Found: 285.0318.

(**28af**) 4-isobutyryl-1*H*-isochromen-1-one

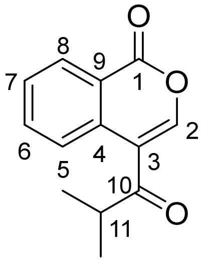

To a solution of $InBr_3$ (0.185 g, 0.522 mmol) in toluene (1 mL), methyl 2-ethynylbenzoate (0.0857 g, 0.535 mmol) was added. The mixture was stirred at 50 °C for 24 h. Pd_2dba_3 (0.030 g, 0.0328 mmol), LiCl (0.043 g, 1.01 mmol), isobutyryl chloride (0.115 g, 1.08 mmol) and HMPA (2.5 mL) was added to the reaction mixture at rt. After stirring at rt for 24 h, the mixture was quenched by Et_2O (5 mL) and water (10 mL), and then extracted with Et_2O (10 mL x 3). The collected organic layer was dried ($MgSO_4$). The solvent was evaporated and the residue was purified by column chromatography (hexane/ethyl acetate = 80:20, column length 11 cm, diameter 26 mm, spherical silica gel) to give the product (0.070 g, 61%). IR: (KBr) 1739 (C=O) cm^{-1}; ^{1}H NMR: (400 MHz, $CDCl_3$) 8.47 (d, J = 8.2 Hz, 1H, 5-H), 8.34 (dd, J = 7.8, 1.4 Hz, 1H, 8-H), 8.03 (s, 1H, 2-H), 7.81 (ddd, J = 8.2, 7.8, 1.4 Hz, 1H, 6-H),

7.60 (t, J = 7.8 Hz, 1H, 7-H), 3.29-3.17 (m, 1H, 11-H), 1.25 (d, J = 6.9 Hz, 6H, 11-Me_2); ^{13}C NMR: (100 MHz, $CDCl_3$) 160.6 (s, C-10), 150.5 (d, C-2), 135.4 (d, C-7), 133.7 (s, C-4), 129.9 (d, C-5), 129.2 (d, C-6), 125.8 (d, C-8), 120.9 (s, C-9), 117.1 (s, C-3), 37.8 (d, C-11), 19.0 (q, 11-Me_2); MS: (EI, 70 eV) m/z 216 (M, 49), 173 (100), 89 (52); HRMS: (CI, 70 eV) Calculated ($C_{13}H_{13}O_3$) 217.0865 $[M + H]^+$ Found: 217.0863.

(**28ag**) 4-benzyl-1*H*-isochromen-1-one

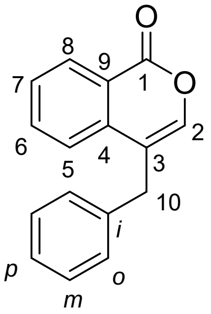

To a solution of $InBr_3$ (0.188 g, 0.530 mmol) in toluene (1 mL), methyl 2-ethynylbenzoate (0.0824 g, 0.514 mmol) was added. The mixture was stirred at 50 °C for 24 h. Pd_2dba_3 (0.0223 g, 0.0242 mmol), LiCl (0.043 g, 1.01 mmol), benzyl bromide (0.176 g, 1.03 mmol) and HMPA (2.5 mL) was added to the reaction mixture at rt. After stirring at 50 °C for 24 h, the mixture was quenched by Et_2O (5 mL) and water (10 mL), and then extracted with Et_2O (10 mL x 3). The collected organic layer was dried ($MgSO_4$). The solvent was evaporated and the residue was purified by column chromatography (hexane/ethyl acetate = 90:10, column length 11 cm, diameter 26 mm, spherical silica gel) to give the product (0.062 g, 51%). IR: (KBr) 1720 (C=O) cm^{-1}; ^1H NMR: (400 MHz, $CDCl_3$) 8.34 (d, J = 8.2 Hz, 1H, 8-H), 7.67 (dd, J = 8.2, 7.8 Hz, 1H, 6-H), 7.51 (dd, J = 8.2, 7.8 Hz, 1H, 7-H), 7.44 (d, J = 8.2 Hz, 1H, 5-H), 7.35-7.28 (m, 2H, *m*), 7.28-7.21 (m, 3H, *o* and *p*), 7.11 (s, 1H, 2-H), 3.93 (s, 2H, 10-

H_2); ^{13}C NMR: (100 MHz, $CDCl_3$) 162.4 (s, C-1), 142.9 (d, C-2), 137.7 (s, *i*), 136.7 (s, C-4), 134.6 (d, C-6), 130.1 (d, C-8), 128.8 (d), 128.4 (d), 128.3 (d, C-7), 126.8 (d, *p*), 123.4 (d, C-5), 121.6 (s, C-9), 115.7 (s, C-3), 33.6 (t, C-10); MS: (EI, 70 eV) m/z 236 (M, 100), 208 (63), 178 (40); HRMS: (CI, 70 eV) Calculated ($C_{13}H_{13}O_3$) 237.0916 $[M + H]^+$ Found: 237.0916.

(**28ah**) 4-(but-2-en-1-yl)-1*H*-isochromen-1-one

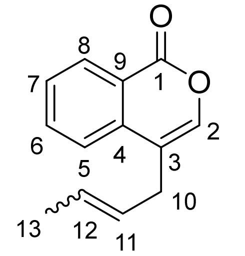

To a solution of $InBr_3$ (0.185 g, 0.522 mmol) in toluene (1 mL), methyl 2-ethynylbenzoate (0.078 g, 0.487 mmol) was added. The mixture was stirred at 50 °C for 24 h. Pd_2dba_3 (0.026 g, 0.0284 mmol), LiCl (0.057 g, 1.34 mmol), 1-bromobut-2-ene (0.149 g, 1.10 mmol) and HMPA (2.5 mL) was added to the reaction mixture at rt. After stirring at 50 °C for 24 h, the mixture was quenched by Et_2O (5 mL) and water (10 mL), and then extracted with Et_2O (10 mL x 3). The collected organic layer was dried ($MgSO_4$). The solvent was evaporated and the residue was purified by column chromatography (hexane/ethyl acetate = 90:10, column length 11 cm, diameter 26 mm, spherical silica gel) to give the product (0.0441 g, 45%, *E*/*Z* = 90:10). IR: (KBr) 1728 (C=O) cm^{-1}; ^{1}H NMR: (400 MHz, $CDCl_3$) 8.34 (d, J = 7.8 Hz, 1H, 8-H), 7.76 (t, J = 7.8 Hz, 1H, 6-H), 7.59-7.50 (m, 2H, 5-H and 7-H), 7.21 (s 1H, 2-H), 5.73-5.50 (m, 2H, 11-H and 12-H), 3.26 (d, J = 4.6 Hz, 2H, 10-H_2), 1.70 (d, J = 5.0 Hz, 3H, 13-H_3); ^{13}C NMR: (100 MHz, $CDCl_3$) 162.6 (s, C-1), 141.8 (d, C-2), 136.9 (s, C-4), 134.5 (s, C-6), 130.0 (d, C-8), 128.4 (d), 128.2 (d), 126.9 (d), 123.1 (d, C-5), 121.5 (s, C-9), 115.9 (s, C-

3), 30.3 (t, C-10), 17.9 (q, C-13); MS: (EI, 70 eV) m/z 200 (M, 100), 171 (33), 157 (39), 128 (46), 115 (30); HRMS: (CI, 70 eV) Calculated ($C_{13}H_{13}O_3$) 201.0916 $[M + H]^+$ Found: 201.0913.

(**30**) 2-iodo-6-methoxybenzoic acid

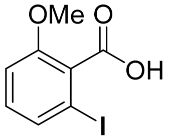

This compound was synthesized by a reported method[12] and the NMR data was agreement with the literature[12]. The spectral data of this compound is shown below.

(**31**) 2-hydroxy-6-iodobenzoic acid

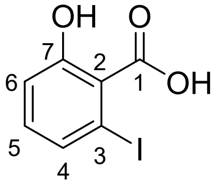

To a three necked flask, 2-iodo-6-methoxybenzoic acid (0.141 g, 0.507 mmol), CH_2Cl_2 (4 mL) and BBr_3 (1 M in CH_2Cl_2, 1.0 mL, 1.0 mmol) were added. After stirring for 20 h at rt, the reaction mixture was evaporated and the residue was diluted with CH_2Cl_2 (10 mL). The solution was washed with 0.5 N HCl aq. (10 mL) and the water layer was extracted by CH_2Cl_2 (10 mL x 3). The corrected organic layer was dried ($MgSO_4$) and the solvent was evaporated to give the product (100% NMR yield). The product was used in next step without further purification. IR: (neat) 3568 (OH) cm^{-1}, 1635 (C=O) cm^{-1}; ^1H NMR: (400 MHz, acetone-d_6) 7.62 (dd, J = 7.7, 0.97 Hz, 1H), 7.19 (t, J = 7.7 Hz, 1H, 5-H), 7.10 (dd, J = 7.7, 0.97 Hz, 1H);

^{13}C NMR: (100 MHz, acetone-d_6) 168.9 (s, C-1), 158.5 (s, C-7), 133.5 (d, C-5), 132.0 (d), 123.5 (s, C-2), 116.8 (d), 93.1 (s, C-3); HRMS: (CI, 70 eV) Calculated ($C_7H_6O_3I$) 264.9362 $[M + H]^+$ Found: 264.9364.

(**32**) methyl 2-hydroxy-6-iodobenzoate

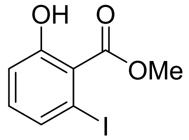

This compound was synthesized by a reported method[13] and the NMR data was agreement with the literature[14]. The spectral data of this compound is shown below.

(**33**) methyl 2-acetoxy-6-iodobenzoate

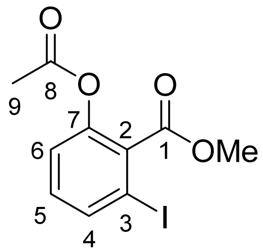

To a three necked flask, methyl 2-hydroxy-6-iodobenzoate (0.136 g, 0.491 mmol), acetone (0.7 mL) and pyridine (0.042 mL, 0.52 mmol) were added. The mixture was cooled to -5 °C and acetyl chloride (37 μL. 0.52 mmol) was added. After stirring for 14 h at rt, the reaction mixture was quenched by H_2O (2 mL). The solution was extracted by CH_2Cl_2 (2 mL x 2). The corrected organic layer was dried ($MgSO_4$) and the solvent was evaporated to give the product (97% NMR yield). The product was used in next step without further purification. IR: (KBr) 1766 (C=O) cm^{-1}, 1732 (C=O) cm^{-1}; ^1H NMR: (400 MHz, $CDCl_3$) 7.72 (dd, J =

6.8, 1.9 Hz, 1H), 7.16 (dd, J = 8.2, 1.9 Hz, 1H), 7.13 (dd, J = 8.2, 6.8 Hz, 1H, 5-H), 3.93 (s, 3H, OMe), 2.26 (s, 3H, 9-H_3); ^{13}C NMR: (100 MHz, $CDCl_3$) 168.4 (s, 8), 166.2 (s,1), 147.7 (s, C-7), 136.7 (d), 132.9 (s, C-2), 131.6 (d, C-5), 122.8 (d), 92.2 (s, C-3), 52.7 (q, OMe), 20.6 (q, C-9); MS: (EI, 70 eV) m/z 320 (M, 19), 278 (100), 246 (100); HRMS: (CI, 70 eV) Calculated ($C_{10}H_{10}O_4I$) 320.9624 $[M + H]^+$ Found: 320.9620.

(**34**) methyl 2-acetoxy-6-((trimethylsilyl)ethynyl)benzoate

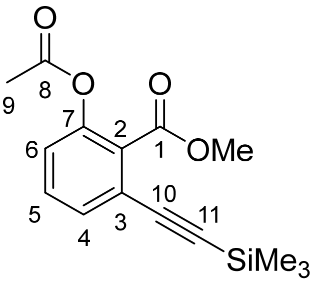

To a three necked flask, methyl 2-acetoxy-6-iodobenzoate (0.156 g, 0.487 mmol), NEt_3 (0.5 mL), $PdCl_2(PPh_3)_2$ (0.0072 g, 0.0103 mmol) and CuI (0.0096 g, 0.0956 mmol) were added. The mixture was cooled to 0 °C and trimethylsilylacetylene (0.0538 g. 0.548 mmol) was added. After stirring for 17 h at rt, the reaction mixture was filtered through a celite pad. The filtrate was evaporated and the residue was purified by column chromatography (hexane/ethyl acetate = 90:10, column length 11 cm, diameter 26 mm, spherical silica gel) to give the product (0.1581 g, 100%). IR: (neat) 1778 (C=O) cm^{-1}, 1736 (C=O) cm^{-1}; ^{1}H NMR: (400 MHz, $CDCl_3$) 7.41 (dd, J = 7.8, 1.8 Hz, 1H, 4-H), 7.38 (t, J = 7.8 Hz, 1H, 5-H), 7.11 (dd, J = 7.8, 1.8 Hz, 1H, 6-H), 3.90 (s, 3H, OMe), 2.29 (s, 3H, 9-H_3), 0.236 (s, 9H, $SiMe_3$); ^{13}C NMR: (100 MHz, $CDCl_3$) 168.9 (s, C-8), 165.5 (s, C-1), 148.2 (s, C-7), 130.7 (d), 130.6 (d), 128.9 (s, C-2), 123.3 (d, C-6), 123.2 (s, C-3), 101.3 (s, C-10), 99.5 (s, C-11), 52.3 (q,

OMe), 20.8 (q, C-9), -0.241 (q, $SiMe_3$); MS: (EI, 70 eV) m/z 290 (M, 8), 248 (79), 233 (32), 216 (100), 203 (85); HRMS: (CI, 70 eV) Calculated ($C_{15}H_{19}O_4Si$) 291.1053 $[M+H]^+$ Found: 291.1054.

(**35**) methyl 2-acetoxy-6-ethynylbenzoate

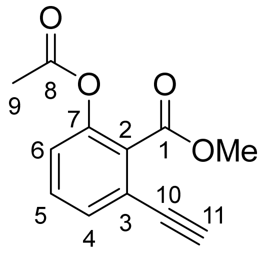

To a three necked flask, methyl 2-acetoxy-6-((trimethylsilyl)ethynyl)benzoate (0.0488 g, 0.168 mmol), DMF (1.7 mL) and 1 M KF aq. (0.28 mL, 0.28 mmol) were added. After stirring for 0.5 h at rt, the reaction mixture was extracted by $CHCl_3$ (5 mL). The organic layer was washed by 10% NH_4Cl aq. (5 mL) and then was dried ($MgSO_4$). The solvent was evaporated to give the product (0.0279 g, 76%). The product was used in next step without further purification. IR: (neat) 1774 (C=O) cm^{-1}, 1732 (C=O) cm^{-1}; ^1H NMR: (400 MHz, $CDCl_3$) 7.46 (dd, J = 8.0, 1.4 Hz, 1H, 4-H), 7.42 (t, J = 8.0 Hz, 1H, 5-H), 7.15 (dd, J = 8.0, 1.4 Hz, 1H, 6-H), 3.92 (s, 3H, OMe), 3.28 (s, 1H, 11-H), 2.29 (s, 3H, 9-H_3); ^{13}C NMR: (100 MHz, $CDCl_3$) 168.8 (s, C-8), 165.3 (s, C-1), 148.2 (s, C-7), 131.1 (d), 130.9 (d), 128.9 (s, C-2), 123.8 (d, C-6), 122.3 (s, C-3), 81.8 (s, C-11), 80.2 (d, C-11), 52.5 (q, OMe), 20.7 (q, C-9); MS: (EI, 70 eV) m/z 218 (M, 7), 176 (98), 144 (100), 116 (36); HRMS: (CI, 70 eV) Calculated ($C_{12}H_{11}O_4$) 219.0657 $[M+H]^+$ Found: 219.0654.

(**36**) 2-(8-acetoxy-1-oxo-1*H*-isochromen-4-yl)-2-oxoethyl acetate

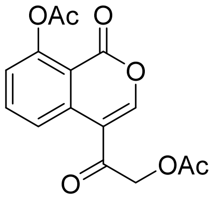

To a solution of $InBr_3$ (0.114 g, 0.322 mmol) in toluene (0.6 mL), methyl 2-acetoxy-6-ethynylbenzoate (0.069 g, 0.316 mmol) was added. The mixture was stirred at 50 °C for 24 h. Pd_2dba_3 (0.0171 g, 0.0187 mmol), LiCl (0.0284 g, 0.670 mmol), 2-chloro-2-oxoethyl acetate (0.087 g, 0.637 mmol) and HMPA (1.5 mL) was added to the reaction mixture at rt. After stirring at rt for 9 h, the mixture was quenched by Et_2O (5 mL) and water (10 mL), and then extracted with Et_2O (10 mL x 3). The collected organic layer was dried ($MgSO_4$). The solvent was evaporated and the residue was purified by column chromatography (hexane/ethyl acetate = 60:40, column length 11 cm, diameter 26 mm, spherical silica gel) to give the product (0.0420 g, 44%). The NMR data was agreement with the literature[15]. The spectral data of this compound is shown below.

Isolation of Organoindium Compounds

(**3**) zwitterion intermediate

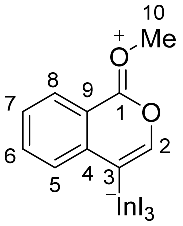

To a sealed vial, InI_3 (0.152 g, 0.307 mmol) in toluene (0.6 mL) and methyl 2-ethynylbenzoate (0.048 g, 0.30 mmol) were added. The mixture was stirred at rt for 24 h. The solvent was removed by decantation to obtain a white solid and the solid was washed by $CHCl_3$ (3 mL x 6). The residue was dried under vacuum to give the product as a white solid including a small amount of a stating ester (0.150 g, 98wt%, 73% yield). This compound was recrystallized from CH_2Cl_2 and hexane to give a single crystal. The structure was determined by X-ray crystallographic analysis (CCDC 1579824). Characterization by NMR study was also carried out and the spectra is shown below. ^{1}H NMR: (400 MHz, CD_2Cl_2) 8.68 (d, J = 8.2 Hz, 1H, 8-H), 8.55 (d, J = 8.2 Hz, 1H, 5-H), 8.35 (t, J = 8.2 Hz, 1H, 6-H), 8.10 (s, 1H, 2-H), 7.97 (t, J = 8.2 Hz, 1H, 7-H), 4.68 (s, 3H, 10-H_3); ^{13}C NMR: (100 MHz, CD_2Cl_2) 171.8 (s, C-1), 146.7 (d, C-2), 144.0 (s, C-4), 141.8 (d, C-6), 132.3 (d, C-7), 130.5 (d, C-5), 129.8 (d, C-8), 127.6 (s, C-3), 115.8 (s, C-9), 62.1 (q, C-10).

^{1}H NMR (400 MHz, in CD_2Cl_2)

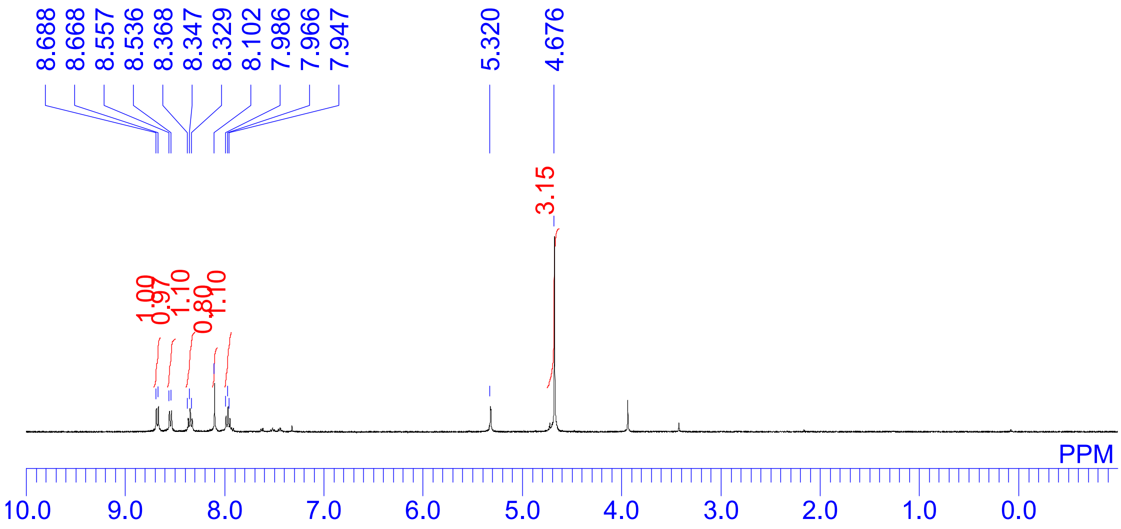

^{13}C NMR (100 MHz, in CD_2Cl_2)

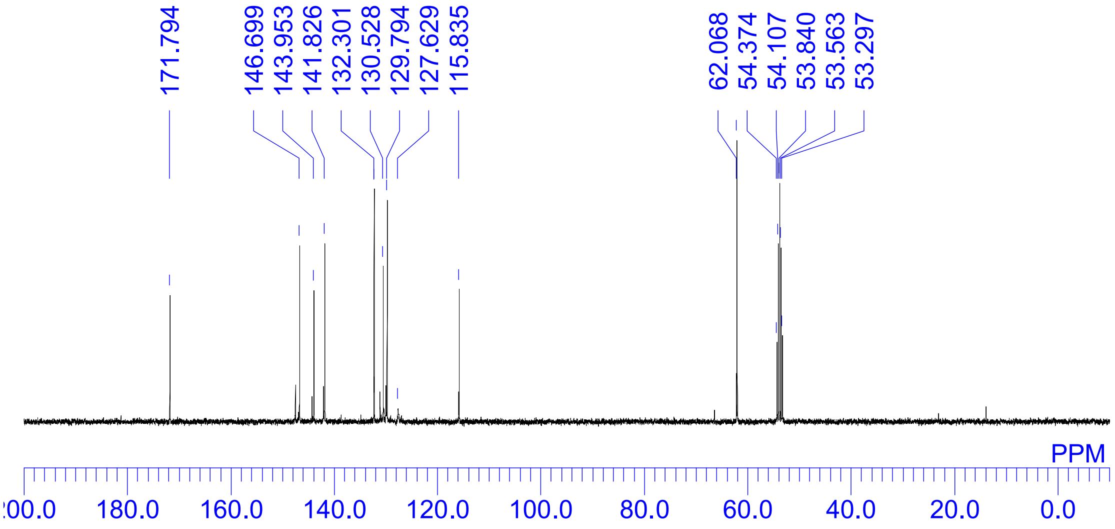

HMBC (400 MHz, CD_2Cl_2)

The signal intensity of carbon atom 3 is very weak because this carbon atom combines with indium atom. However, HMBC measurement showed the correlation of the signal at 127.6 ppm with 5-H and 2-H, and thus we assigned this signal as carbon atom 3.

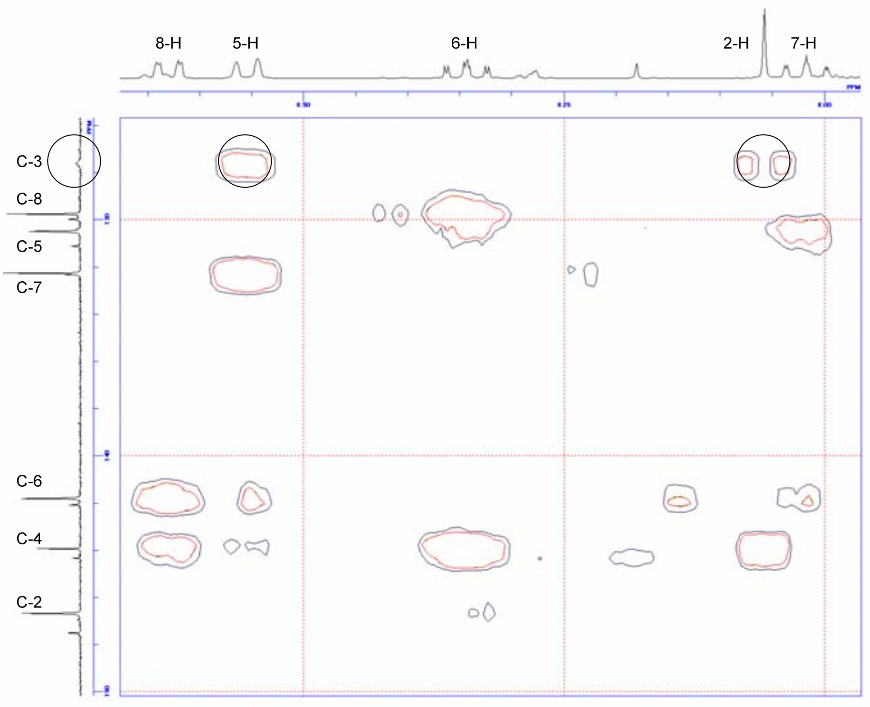

(**4a**) (1-oxo-1*H*-isochromen-4-yl)indium(III) iodide

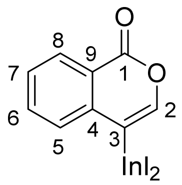

To a sealed vial, zwitterion intermediate **3** (0.190 g, 98wt%, 0.284 mmol) in toluene (0.6 mL) was added. The mixture was stirred at 50 °C for 10 h. The solvent was removed by decantation to obtain a white solid and the solid was washed by $CHCl_3$ (3 mL x 2). The residue was dried under vacuum to give the product as a white solid (0.150 g, 100%). This compound was characterization by NMR study and the spectra is shown below. ^{1}H NMR: (400 MHz, THF-d_8) 8.26 (d, *J* = 7.7 Hz, 1H, 8-H), 8.09 (d, *J* = 7.7 Hz, 1H, 5-H), 7.75 (t, *J* = 7.7 Hz, 1H, 6-H), 7.53 (t, *J* = 7.7 Hz, 1H, 7-H), 7.17 (s, 1H, 2-H); ^{13}C NMR: (100 MHz, THF-d_8) 161.9 (s, C-1), 149.8 (d, C-2), 141.3 (s, C-4), 135.2 (d, C-6), 130.4 (d, C-8), 129.1 (d, C-7), 128.7 (d, C-5), 124.3 (s, C-9), 120.2 (s, C-3).

^{1}H NMR (400 MHz, in THF-d_8)

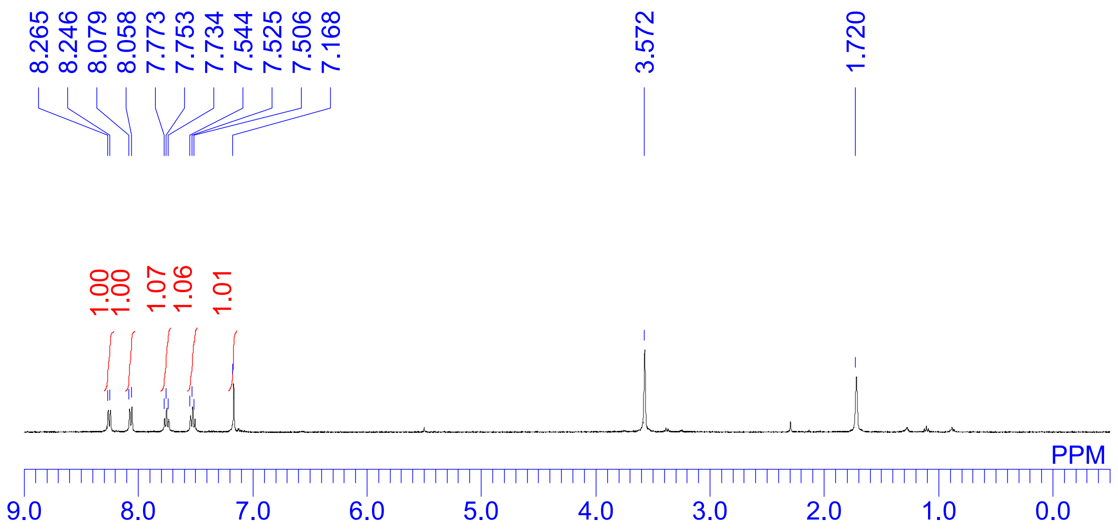

^{13}C NMR (100 MHz, in THF-d_8)

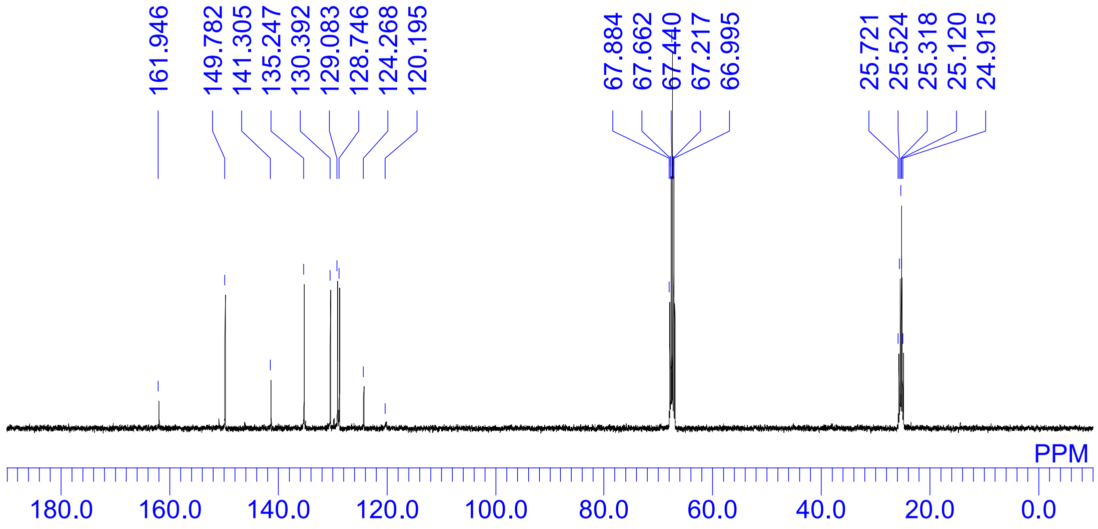

HMBC (400 MHz, in THF-d_8)

The same correlation with zwitterion **3** was observed for **4a** and we assigned the signal at 120.2 ppm as the carbon atom 3.

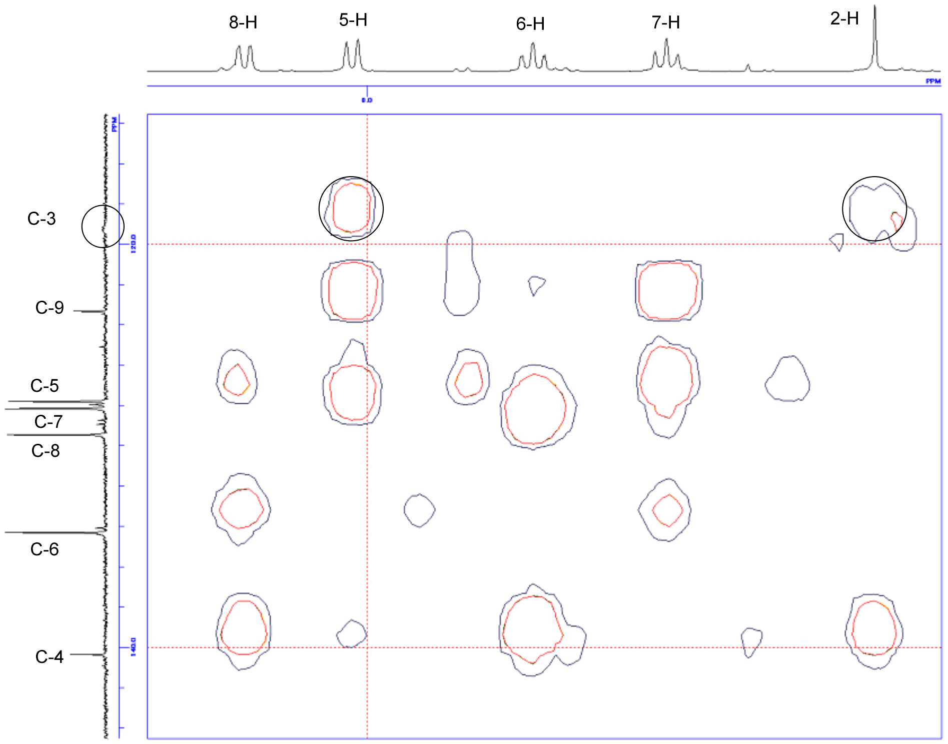

(**4b**) (7-nitro-1-oxo-1*H*-isochromen-4-yl)indium(III) iodide

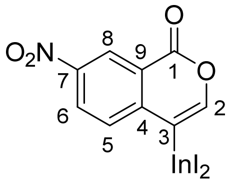

To a sealed vial, InI_3 (0.250 g, 0.505 mmol) in toluene (1.0 mL) and methyl 2-ethynyl-5-nitrobenzoate (0.089 g, 0.434 mmol) were added. The mixture was stirred at 50 °C for 12 h. The solvent was removed by decantation to obtain a white solid and the solid was washed by CH_2Cl_2 (3 mL x 2). The residue was dried under vacuum to give the product as a white solid (0.218 g, 90%). This compound was recrystallized from THF and hexane to give a single crystal. The structure was determined by X-ray crystallographic analysis (CCDC 1576342). Characterization by NMR study was also carried out and the spectra is shown below. ^{1}H NMR: (400 MHz, THF-d_8) 8.99 (s, 1H, 8-H), 8.61 (d, J = 7.2 Hz, 1H, 6-H), 8.25 (d, J = 7.2 Hz, 1H, 5-H), 7.35 (s, 1H, 2-H); ^{13}C NMR: (100 MHz, THF-d_8) 160.8 (s, C-1), 152.7 (d, C-2), 148.1 (s, C-7), 146.5 (s, C-4), 130.2 (d), 129.4 (d), 125.7 (d, C-8), 124.8 (s, C-9), 119.3 (s, C-3).

^{1}H NMR (400 MHz, in THF-d_8)

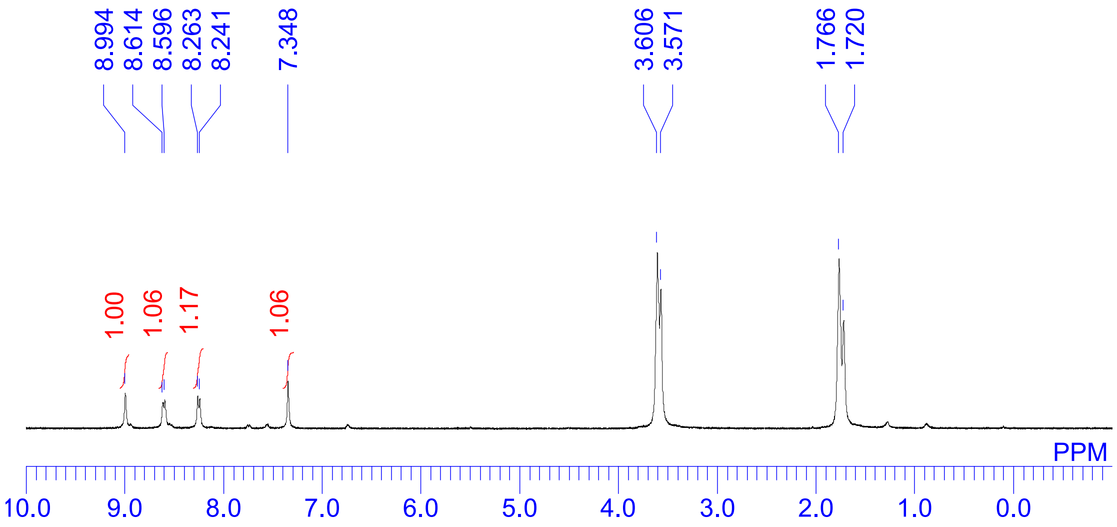

^{13}C NMR (100 MHz, in THF-d_8)

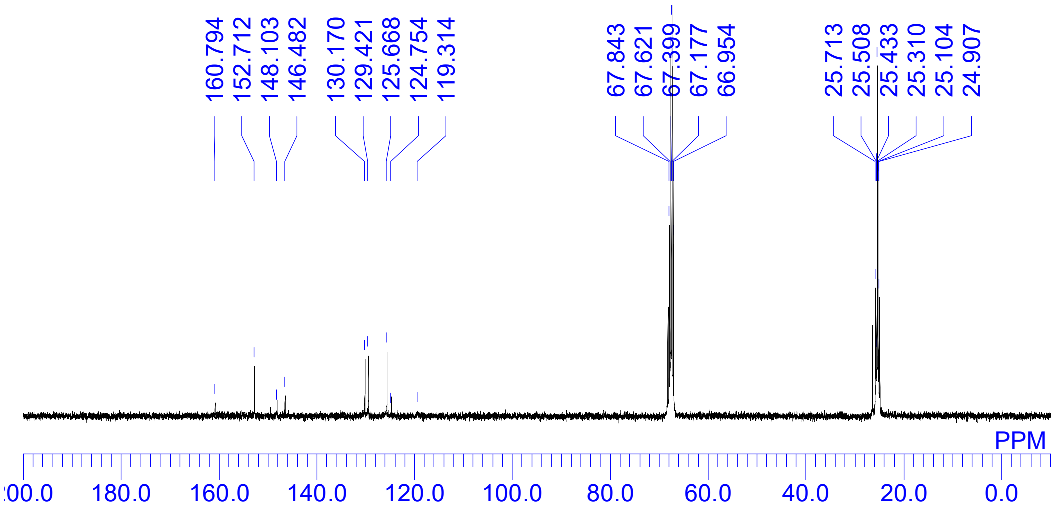

HMBC (400 MHz, in THF-d_8)

The same correlation with zwitterion **3** was observed for **4b** and we assigned the signal at 119.3 ppm as the carbon atom 3.

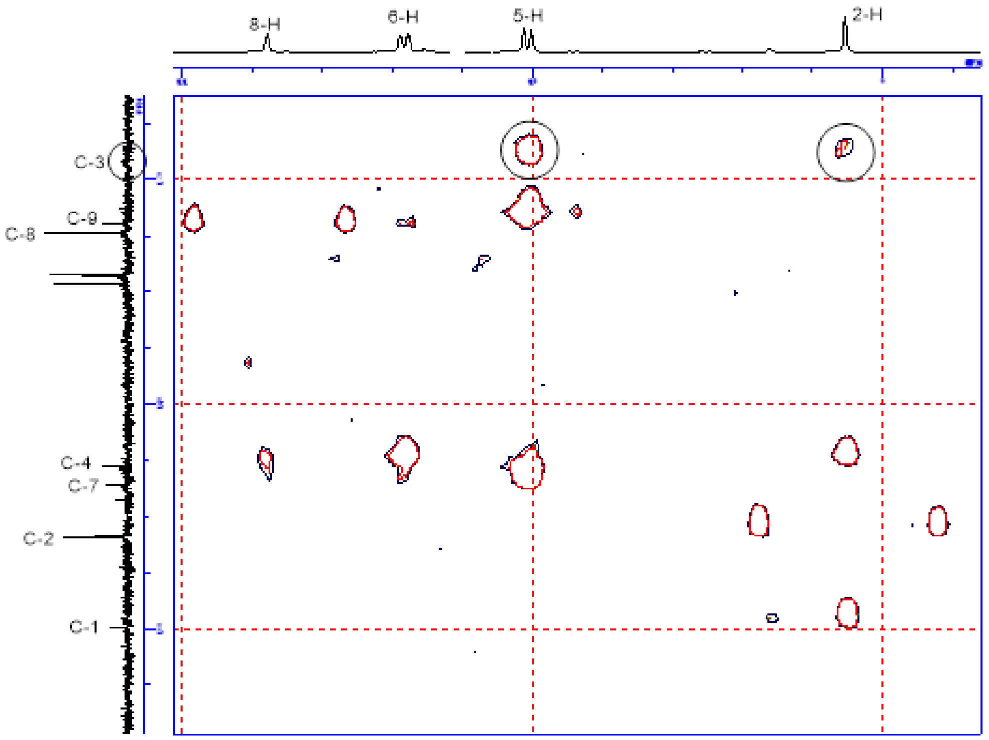

Computational Details

All calculations (Fig. 3-6) were performed with Gaussian09 rev.E.01. The wB97XD density functional and a mixed basis set of DGDZVP for In and I and 6-31+G(d,p) for other atoms were used. All molecular geometries were fully optimized and Gibbs free energies including contribution of vibrational entropy at an appropriate temperature were described in energy profiles. Solvation effect was introduced using the IEFPCM model and toluene was used as a solvent. Aromaticity of 6- and 5-membered zwitterions (**3** and **6**) was calculated by the B3LYP density functional and a mixed basis set of 6-31G (d,p) for C, H, O and DGDZVP for In, I for their optimized structures. Electrostatic potential maps were calculated on the 0.001 au isosurface of electron density for optimized structures of transition states of oxyindation and oxyboration. The potential is depicted with a color gradient from the most negative (red) to the most positive (blue) value (kcal/mol).

The energy profile of dissociation of In_2I_6 and In_2Cl_6

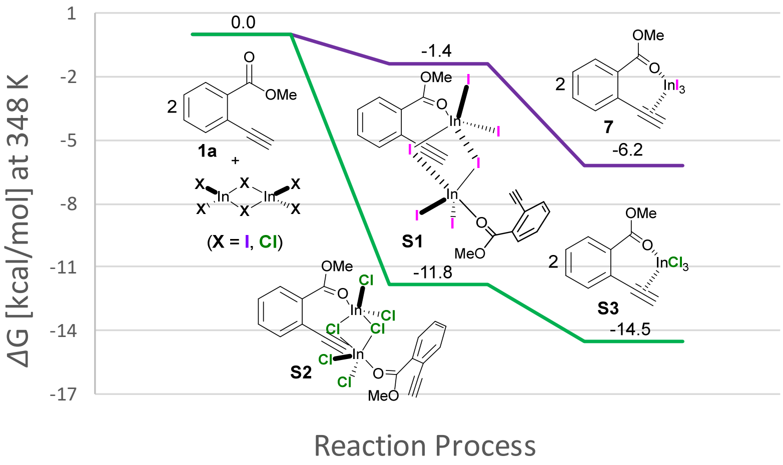

Fig. S3 The energy profile of dissociation of In_2I_6 and In_2Cl_6

Coordination of two molecules of **1a** to In_2X_6 gives **S1** or **S2**. The aggregation of In_2X_6 was dissociated to generate **7** or **S3**. The all steps are exergonic to afford the complex **7** or **S3** initiatively.

The energy profile of intramolecular elimination of MeI

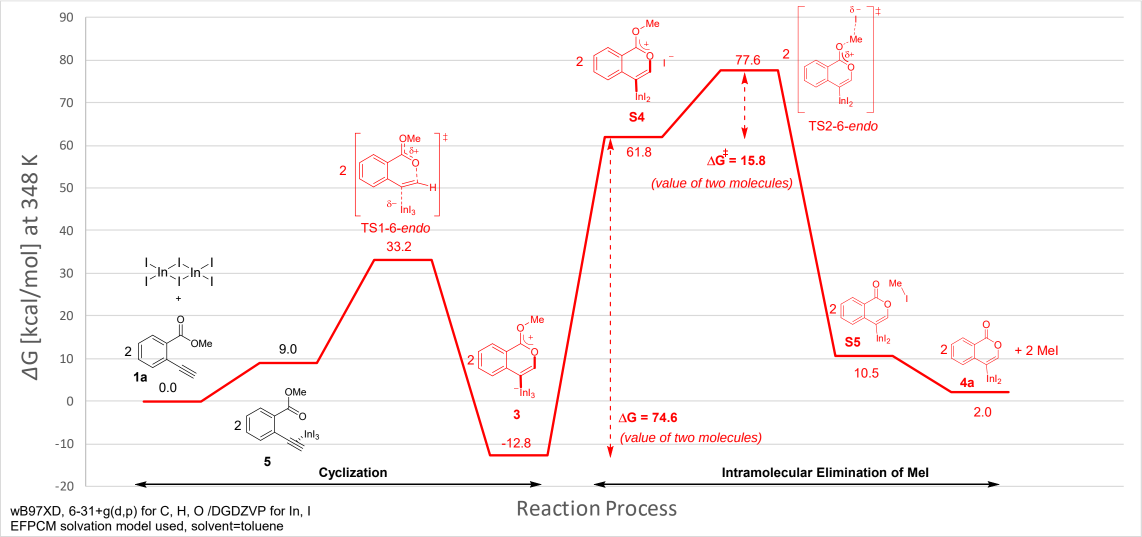

Fig. S4 The energy profile of intramolecular elimination of MeI

In this pathway, oxyindation proceeds in a concerted mechanism, and then dissociation of iodide from zwitterion **3** gives complex **S4**. Elimination of MeI takes places to give **S5**. The activation energy of dealkylation step (**S4** to **S5**) is only 7.9 kcal/mol. However, the intermediate **S4** is too unstable to be generated.

The energy profile of oxyindation using $InCl_3$

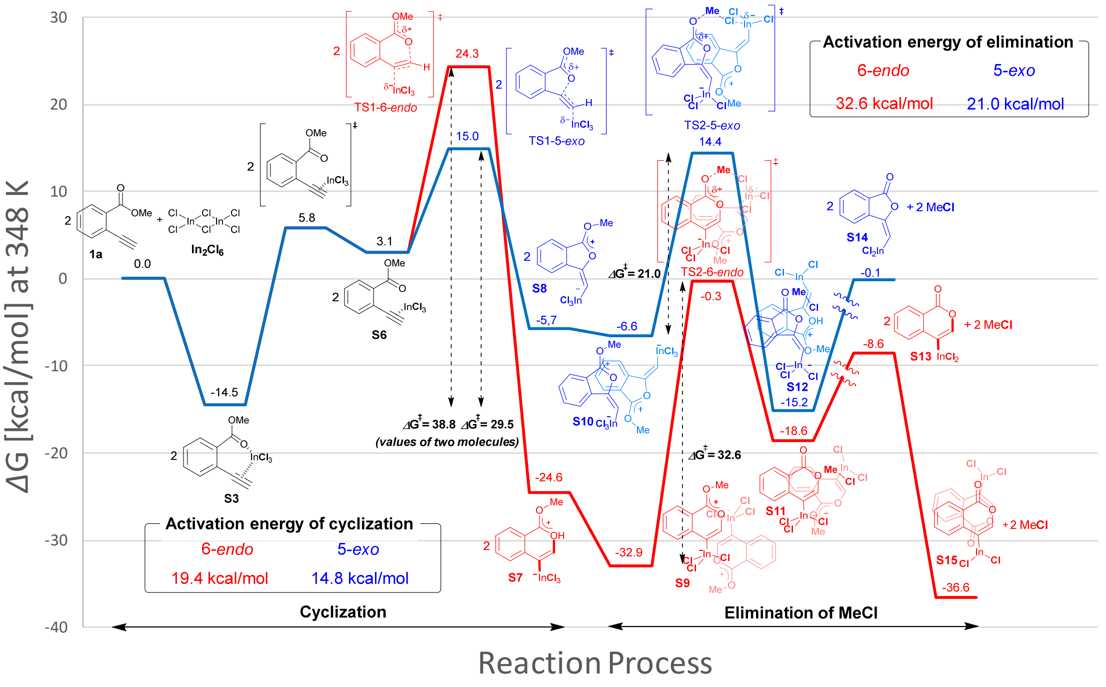

Fig. S5 The energy profile of oxyindation using $InCl_3$.

We found that oxyindation using $InCl_3$ proceeds through the same fashion with InI_3. In the case of $InCl_3$, the energy level of all structures became lower than that of the case with InI_3. The remarkable difference between the cases of $InCl_3$ and InI_3 was the activation energy of elimination step. The elimination of MeCl (**S9** to TS2-6-*endo*) showed higher activation energy (32.7 kcal/mol) than that of the elimination of MeI (**8** to TS2-6-*endo*, 28.7 kcal/mol). This inhibition of elimination step, which is a rate determining step, was rationalized by the low nucleophilicity of Cl^-, and it caused much less reactivity of $InCl_3$.

The comparison of elimination steps in oxyboration

(i) The energy profile of elimination of MeCl by chloride on the zwitterion intermediate

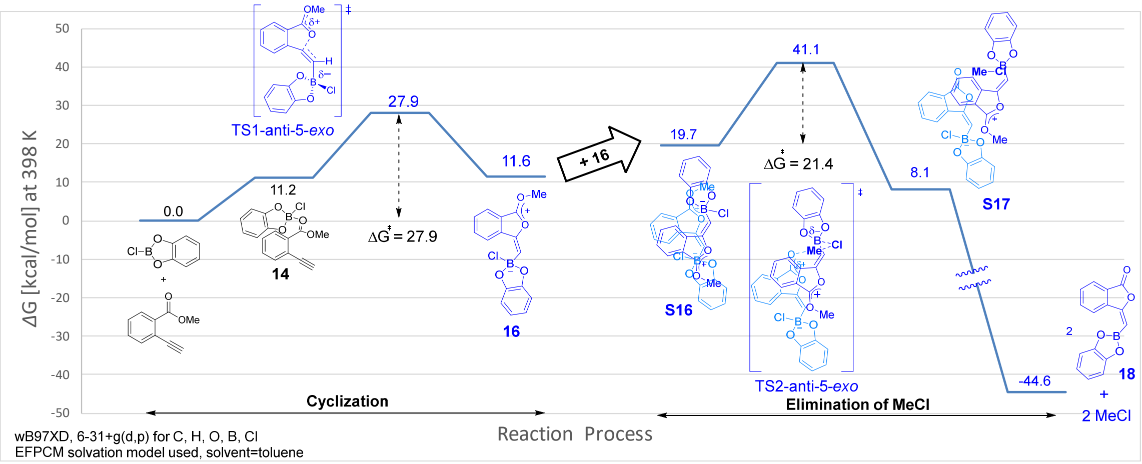

Fig. S6 The energy profile of elimination of MeCl by another zwitterion intermediate.

After 5-*exo* cyclization, two zwitterions aggregate in head to tail fashion to give the complex **S16**. The elimination step starts from **S16**. Intermolecular nucleophilic substitution of methyl group by chloride proceeds in SN2 manner to give complex **S17** and MeCl, and then further elimination of MeCl affords target product.

(ii) The energy profile of elimination of MeCl by free chloride

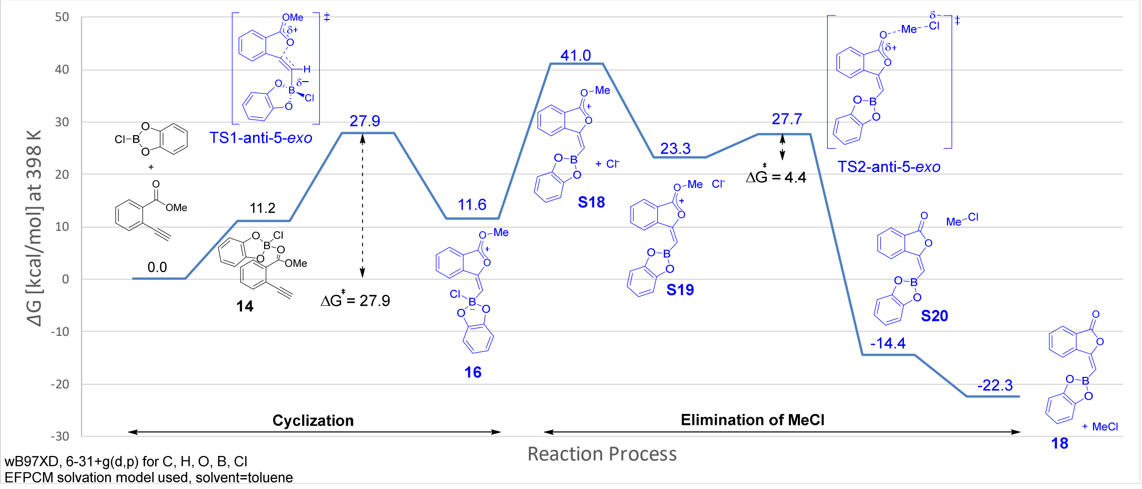

Fig. S7 The energy profile of elimination of MeCl by free chloride

After 5-*exo* cyclization, dissociation of chloride from zwitterion **16** provides free chloride **S18**. After the chloride move toward the methyl moiety (**S19**), elimination of MeCl proceeds to give **S20**.

(iii) The energy profile of elimination of MeCl by $[Cl_2Bcat]^-$ (the most probable path)

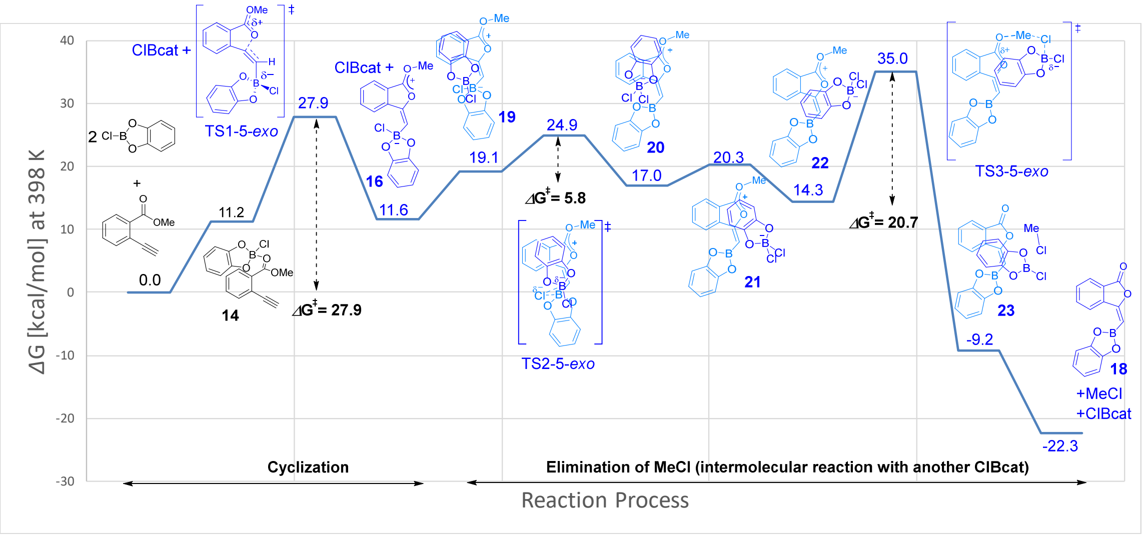

Fig. S8 The energy profile of elimination of MeCl by $[Cl_2Bcat]^-$

In the case of path i, the activation energy of elimination step (**S16** to **S17**, 21.4 kcal/mol) is higher than that of elimination in the path iii (**22** to **23**, 20.7 kcal/mol). In the path ii, the activation energy of dealkylation step (**S19** to **S20**) is only 4.4 kcal/mol. However, the intermediate **S18**, which has naked Cl-, is too unstable to be generated. Therefore, path iii is the most probable reaction process of oxyboration.

The value of electrostatic potential

The value of V_{max}, V_{min} and the difference ΔV was summarized in Table S2. The value of V_{max} was higher in transition state of oxyindation than that of oxyboration. However, the transition state of oxyboration showed much more negative value of V_{min} than that of oxyindation, and thus the value of difference ΔV was small in the case of oxyindation compared with oxyboration. As the total result, the transition state of oxyindation delocalized the increasing charge more efficiently than that of oxyboration.

Table S2 The value of $V_{\max}$, $V_{\min}$ and the difference ΔV for the electrostatic potential of transition state of cyclization step

	Oxyindation	Oxyboration
	TS1-6-*endo*	TS1-5-*exo*
$V_{\max}$ (kcal/mol)	43.0	33.2
$V_{\min}$ (kcal/mol)	-21.3	-43.8
ΔV (kcal/mol)	64.3	77.0

The energy diagram for 5-*exo* cyclic oxyindation with syn-addition

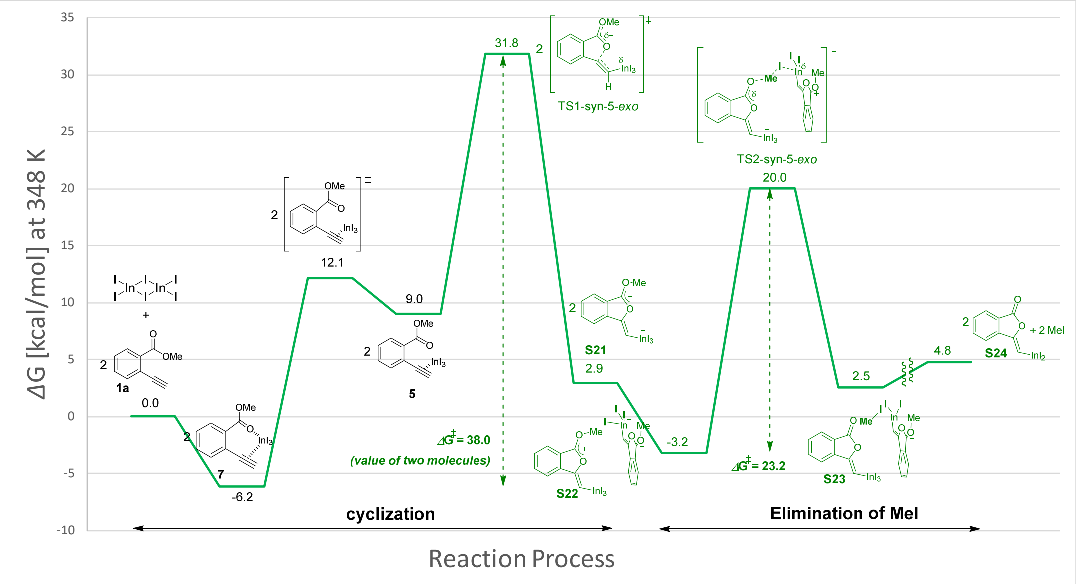

Fig. S9 The energy diagram for 5-*exo* cyclic oxyindation with syn-addition.

In this pathway, cyclization proceeds in a concerted mechanism (**5** to **S21**), and then two zwitterions aggregate in head to tail fashion to give complex **S22**. The elimination step starts from **S22** and intermolecular nucleophilic substitution of methyl group by iodide proceeds in an SN2-manner to give complex **S23** and MeI. Finally, further elimination of MeI affords product **S24**. The zwitterion intermediate **S21** was more unstable than 6-membered zwitterion **3**, and thus reversible cyclization occurs like as the case of anti 5-*exo* cyclization. Therefore, the product **S24** is not obtained.

The energy diagram for 5-*exo* cyclization of oxyboration with syn-addition

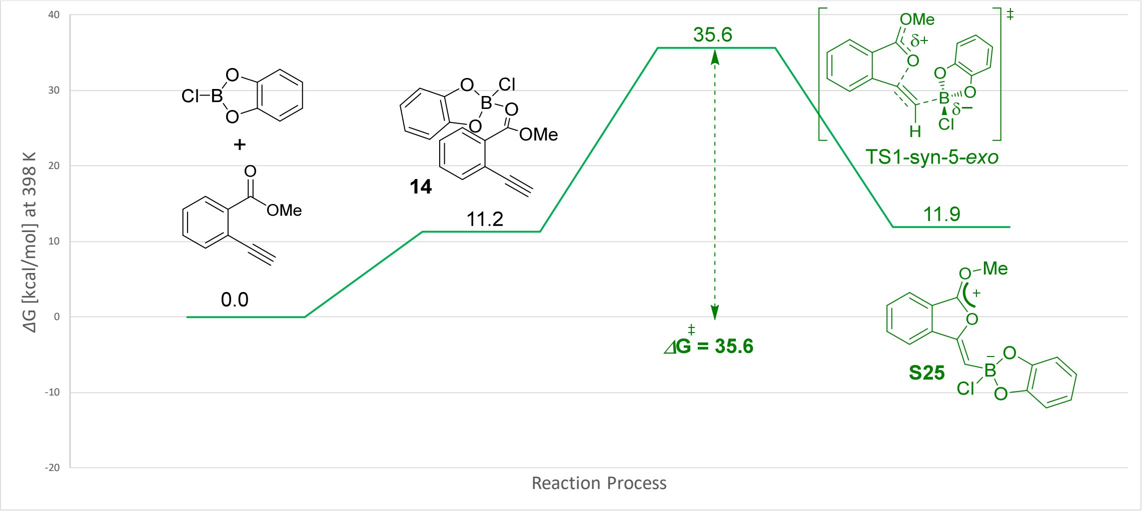

Fig. S10 The energy diagram for 5-*exo* cyclization of oxyboration with syn-addition.

In this pathway, oxyboration proceeds in a concerted mechanism (**14** to **S25**). The activation energy was higher than that of anti 5-*exo* cyclization so that this process is disfavor.

Cartesian coordinates of all optimized structures shown in Fig. 3, 5 and S3-S8 and their Gibbs free energies (in hartree)

1a

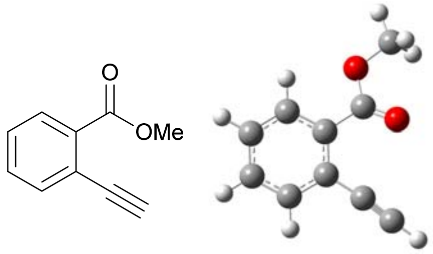

G (348K) = -536.005665

C	2.47391903	0.11894579	-0.03568024
C	1.15437767	0.59715637	-0.01099515
C	0.09477236	-0.33343485	0.03504752
C	0.38313701	-1.70195898	0.05281738
C	1.69491893	-2.15893762	0.03592021
C	2.74344613	-1.24300857	-0.00915452
H	3.28580750	0.83679575	-0.07747670
H	-0.43714206	-2.40859881	0.08573545
H	1.89667623	-3.22458981	0.05688854
H	3.77222426	-1.58770925	-0.02524849
C	0.97458019	2.01997272	-0.05760254
C	0.94223947	3.22627022	-0.11325026
H	0.86881039	4.29014099	-0.15414920
C	-1.32756345	0.12080378	0.08234048
O	-1.69393900	1.24978886	0.32518200
O	-2.18231933	-0.88270911	-0.16994753
C	-3.57210876	-0.54720963	-0.11496266
H	-4.10274841	-1.47199986	-0.33434131
H	-3.83580449	-0.17875006	0.87849913
H	-3.80806807	0.21647764	-0.85866458

In_2I_6

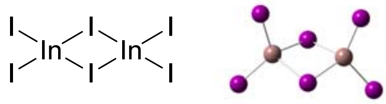

G (348K) = -53004.71672

In	1.94728359	0.03773856	0.00006511
In	-1.94730765	0.03772351	-0.00008003
I	3.68859010	2.08922179	0.00004219
I	2.74248375	-2.53422359	0.00024958
I	-0.00008931	0.41024459	2.09408712
I	0.00006873	0.40998915	-2.09414528
I	-2.74246620	-2.53423928	0.00005028
I	-3.68856482	2.08924053	-0.00027011

S1 (Fig. S3)

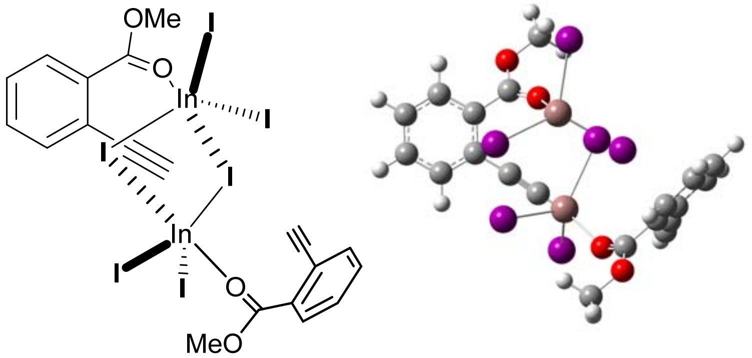

G (348K) = -54076.7302

C	4.57061435	-3.65416328	0.84294606
C	4.52752235	-2.26968717	1.04951608
C	5.35189242	-1.44392811	0.25759302
C	6.19635348	-2.00613015	-0.70421906
C	6.20948348	-3.37975626	-0.90993107
C	5.39405541	-4.20151032	-0.13364701

H	3.94112230	-4.29194633	1.45302011
H	6.82480353	-1.35703610	-1.30352810
H	6.85144655	-3.80616129	-1.67284413
H	5.39932343	-5.27543440	-0.28786902
C	3.67714428	-1.75874214	2.08171416
C	2.94600323	-1.38838511	2.96880523
H	2.29024618	-1.04018408	3.73574728
C	5.31593441	0.02619100	0.39126703
O	4.30151833	0.69148206	0.63569005
O	6.47549950	0.61027705	0.22016402
C	6.50006751	2.05442616	0.25230002
H	7.53023856	2.32130318	0.02997100
H	5.81664647	2.45367919	-0.49840304
H	6.21063047	2.40244018	1.24450309
In	-2.18904617	-0.92876807	-0.04229800
In	2.18820517	0.92883507	-0.04290800
I	-2.77847121	-2.83924922	-1.91100115
I	-1.14126409	-1.92175414	2.25539417
I	1.61124112	-1.47716311	-1.17601809
I	-1.60989812	1.47704812	-1.17478309
I	1.14161609	1.92190114	2.25536517
I	2.77894821	2.84041722	-1.91013315
C	-4.57166035	3.65346428	0.84211206
C	-4.52846234	2.26903518	1.04895308
C	-5.35280240	1.44306511	0.25720402
C	-6.19734246	2.00501115	-0.70469205
C	-6.21057845	3.37859726	-0.91065907
C	-5.39517642	4.20055432	-0.13456201
H	-3.94220330	4.29141233	1.45204911
H	-6.82578150	1.35575510	-1.30383810
H	-6.85260052	3.80482329	-1.67362113
H	-5.40052741	5.27444740	-0.28900202
C	-3.67806528	1.75834814	2.08126816

C	-2.94676223	1.38833411	2.96837123
H	-2.29142217	1.04001608	3.73561929
C	-5.31674442	-0.02701100	0.39114503
O	-4.30223533	-0.69214805	0.63572905
O	-6.47621051	-0.61126704	0.22010102
C	-6.50065548	-2.05542816	0.25223302
H	-7.53069958	-2.32241318	0.02944900
H	-5.81686946	-2.45463119	-0.49816404
H	-6.21164247	-2.40340519	1.24457209

7

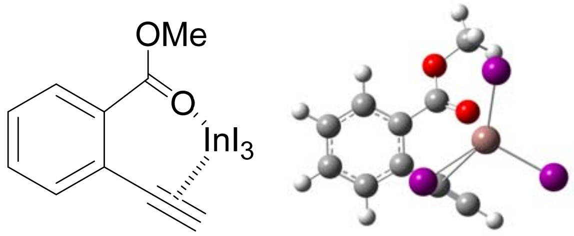

G (348K) = -27038.368944

C	-4.25056032	-1.22847959	-0.37637861
C	-3.06388930	-0.76672498	-0.95848611
C	-2.85649883	0.62512667	-1.05276007
C	-3.82295799	1.51654137	-0.57864854
C	-4.98340234	1.03957615	0.01770367
C	-5.19381656	-0.33462200	0.11704734
H	-4.41799197	-2.29749183	-0.30963702
H	-3.64846388	2.58345859	-0.66120735
H	-5.71906312	1.73689826	0.40293400
H	-6.09921839	-0.71409451	0.57892040
C	-2.13255341	-1.72426170	-1.47482097
C	-1.38504772	-2.57099781	-1.90270603
H	-0.70661318	-3.30702622	-2.27406248
C	-1.60724986	1.17133224	-1.61762254
O	-0.48354093	0.67005872	-1.46533491

O	-1.76202474	2.26718806	-2.31324358
C	-0.57001960	2.90591509	-2.82388898
H	-0.92435149	3.81470113	-3.30361925
H	0.10987596	3.13151725	-2.00129875
H	-0.08362747	2.24691385	-3.54365045
In	0.77035906	-0.11015202	0.18566271
I	-0.89250187	-0.95364667	2.15870257
I	2.36942568	-1.89539368	-1.07980395
I	1.93632826	2.30006235	0.73517951

TS (7 to 5)

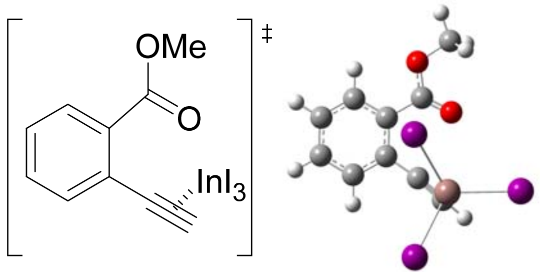

G (348K) = -27038.35439

C	-2.29061468	-2.48535789	-1.37041460
C	-2.18725127	-1.08811118	-1.39510383
C	-3.23812453	-0.29306289	-0.89262638
C	-4.37725209	-0.92070040	-0.39346971
C	-4.47855434	-2.30999756	-0.38405626
C	-3.43519925	-3.09356521	-0.86867020
H	-1.46575585	-3.08201789	-1.74451123
H	-5.18360030	-0.31363084	-0.00092617
H	-5.37326854	-2.77903938	0.01122303
H	-3.50701557	-4.17551803	-0.85239558
C	-0.97805435	-0.54506752	-1.93205711
C	0.10252952	-0.21104874	-2.38577848
H	0.94138922	0.09567271	-2.97715290
C	-3.10944353	1.19699108	-0.85933471

O	-2.16619265	1.81094926	-1.31220207
O	-4.14852301	1.77961069	-0.26225251
C	-4.08021235	3.20668794	-0.13941992
H	-4.99263896	3.49547475	0.37852209
H	-3.19965809	3.48961334	0.44054326
H	-4.03293922	3.66911077	-1.12710505
In	0.94161493	0.03169247	0.03474727
I	2.34633497	-2.30153035	-0.03231644
I	2.30786673	2.31639904	-0.48623759
I	-0.88768959	0.14706184	2.03724101

5

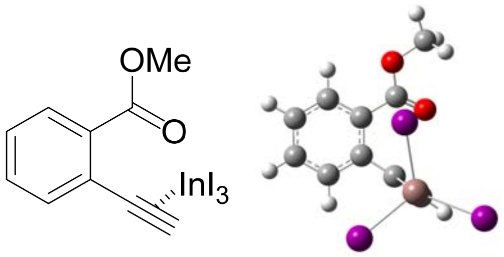

G (348K) = -27038.35683

C	2.13309493	-2.45485607	-1.18567513
C	2.17382880	-1.05993239	-1.31814009
C	3.28747846	-0.33729981	-0.84008080
C	4.33081882	-1.03025078	-0.23290377
C	4.28497432	-2.41786026	-0.10925261
C	3.18865997	-3.13090197	-0.58524661
H	1.26479651	-2.99523549	-1.54577986
H	5.18303791	-0.47711358	0.14301361
H	5.10950005	-2.94043825	0.36396519
H	3.14894555	-4.20988114	-0.48502147
C	1.03450870	-0.42743861	-1.90301242
C	-0.01456648	0.01266259	-2.34551694
H	-0.77942378	0.42285516	-2.97399745
C	3.35123668	1.14881313	-0.99944989

O	2.69536857	1.77570681	-1.80402176
O	4.22418137	1.70362505	-0.15934269
C	4.32690380	3.13231207	-0.21658374
H	5.07027374	3.39852682	0.53218087
H	4.64714800	3.44996472	-1.21071751
H	3.36146096	3.58406762	0.02036701
In	-0.97854627	0.04219422	0.01504416
I	0.80872297	1.06520472	1.78475414
I	-3.02864904	1.68892454	-0.67049617
I	-1.61016825	-2.57953313	0.36764605

TS1-6-*endo* (Fig. 3)

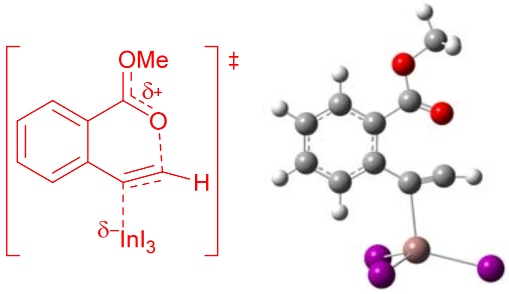

G (348K) = -27038.33755

C	2.12224405	-1.86223017	-0.01894802
C	2.42167800	-0.49915205	-0.04069002
C	3.77343908	-0.08036795	-0.04045802
C	4.77999821	-1.05169697	-0.01681302
C	4.46714125	-2.40607309	0.00461399
C	3.13619517	-2.81187919	0.00334399
H	1.08928298	-2.18901625	-0.02043502
H	5.81582630	-0.73703490	-0.01606801
H	5.26419235	-3.14149411	0.02228799
H	2.88211121	-3.86612728	0.01967899
C	1.36894687	0.50348797	-0.06387002
C	1.24127080	1.73560006	-0.09958702
H	0.89862572	2.75048412	-0.12802702

C	4.16831504	1.36133418	-0.06504902
O	3.40917793	2.32085921	-0.11116302
O	5.48613813	1.52461226	-0.03194002
C	5.96894908	2.87594639	-0.05282602
H	7.05309319	2.79166743	-0.01813502
H	5.64899005	3.37579341	-0.96869009
H	5.59563004	3.42213641	0.81513805
In	-1.01932129	0.03644082	0.00233999
I	-1.13799723	-1.32711229	2.35883817
I	-2.43067151	2.37056393	-0.02116202
I	-1.26439923	-1.44997231	-2.26850419

TS1-5-*exo* (Fig. 3)

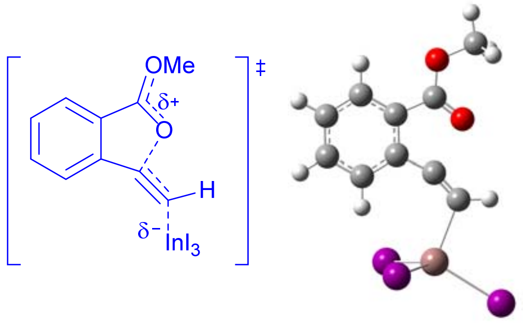

G (348K) = -27038.34428

C	-2.41870643	-1.57356555	-0.00008366
C	-2.82202661	-0.23632542	0.00018913
C	-4.18786265	0.07722921	0.00022019
C	-5.15895676	-0.91717962	-0.00001855
C	-4.75304919	-2.24835586	-0.00028990
C	-3.39395139	-2.56702698	-0.00032145
H	-1.37075013	-1.84481866	-0.00010500
H	-6.20958037	-0.64935947	0.00000736
H	-5.49420290	-3.03984415	-0.00047770
H	-3.08243934	-3.60586915	-0.00053446
C	-1.89345574	0.86247847	0.00044100

C	-0.77909895	1.44815636	0.00057305
H	-0.52248921	2.49829664	0.00084780
C	-4.41978051	1.52666449	0.00050655
O	-3.43799960	2.27221036	0.00073839
O	-5.66355364	1.93806458	0.00046397
C	-5.87995094	3.36386644	0.00061631
H	-6.96015268	3.48689752	0.00052165
H	-5.43786409	3.80448871	0.89520065
H	-5.43768195	3.80469148	-0.89377963
In	1.11171816	0.10998638	0.00002426
I	0.96967258	-1.38548756	-2.28957993
I	3.09968681	1.98105234	0.00041163
I	0.97015818	-1.38691694	2.28872520

3

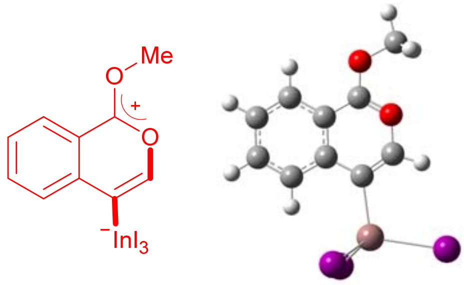

G (348K) = -27038.3742

C	-2.01040992	2.05054911	-0.00039374
C	-2.36146921	0.68700011	-0.00014834
C	-3.74548074	0.36999441	-0.00021367
C	-4.74024058	1.37372330	-0.00051309
C	-4.35527723	2.69375354	-0.00068363
C	-2.98579541	3.02708515	-0.00061529
H	-0.96423629	2.33536632	-0.00038583
H	-5.78760533	1.09469201	-0.00059911
H	-5.10276103	3.47908935	-0.00089788
H	-2.69120545	4.07127218	-0.00074162

C	-1.37679510	-0.37607285	0.00006962
C	-1.83619100	-1.63453997	0.00027754
H	-1.26673505	-2.55421284	0.00044539
C	-4.09285508	-1.00407369	0.00004227
O	-3.19669304	-1.94107193	0.00027414
O	-5.33390024	-1.37777344	0.00005189
C	-5.65750959	-2.78874438	0.00040493
H	-6.74348502	-2.81954173	0.00059191
H	-5.25412860	-3.25872109	-0.89692499
H	-5.25382950	-3.25842654	0.89775179
In	0.82901607	-0.07803533	0.00013764
I	1.47393016	1.34510215	2.27435572
I	1.95097327	-2.59674222	0.00038260
I	1.47432466	1.34395947	-2.27469967

6

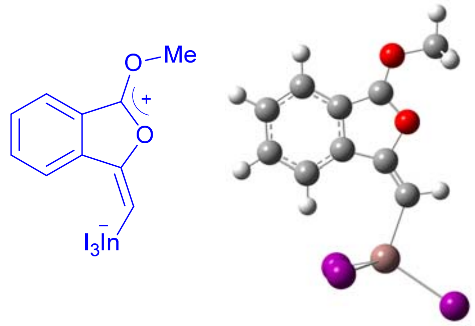

G (348K) = -27038.35907

C	-2.41997609	-1.55238129	0.00013772
C	-2.84544948	-0.22526925	-0.00067208
C	-4.22023490	0.06093668	-0.00068309
C	-5.20690165	-0.93001665	0.00016408
C	-4.77092227	-2.24355396	0.00100715
C	-3.39535697	-2.54213949	0.00096384
H	-1.37146372	-1.82015583	0.00009766
H	-6.26073154	-0.67573063	0.00015924

H	-5.49187346	-3.05326497	0.00168772
H	-3.08302068	-3.58107945	0.00160111
C	-2.11317766	1.03462864	-0.00160020
C	-0.84454554	1.41771170	-0.00182494
H	-0.68704787	2.49621272	-0.00258629
C	-4.30991499	1.48504240	-0.00169474
O	-3.14970398	2.06002795	-0.00226103
O	-5.39188007	2.17475346	-0.00206321
C	-5.30200879	3.62546381	-0.00295129
H	-6.33384500	3.96425396	-0.00286920
H	-4.77908701	3.95480311	0.89489859
H	-4.77958611	3.95373484	-0.90148418
In	0.98164728	0.16281461	-0.00026744
I	0.95743521	-1.41268739	-2.27203046
I	3.09918607	1.91801173	-0.00045292
I	0.95450214	-1.40867296	2.27435348

8

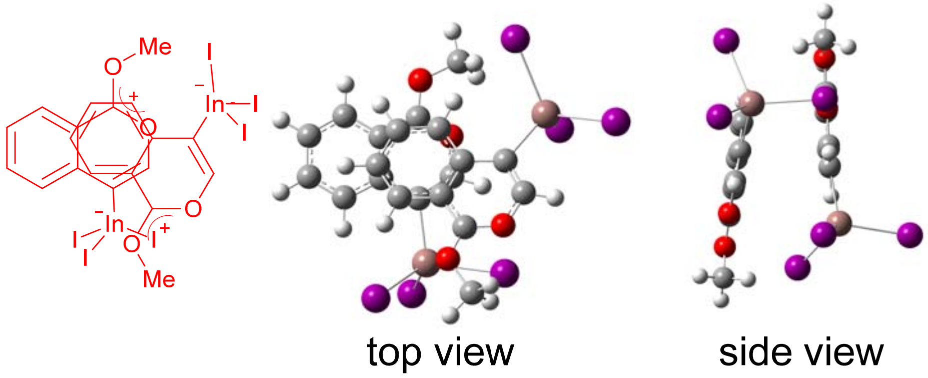

G (348K) = -54076.75721

C	1.06657670	1.35156699	2.04927467
C	0.88581297	-0.03692049	1.89546116
C	-0.25410470	-0.61205752	2.51125089
C	-1.19101247	0.16739267	3.22406366
C	-0.98012051	1.52086178	3.34864683

C	0.16272184	2.10546888	2.77137074
H	1.94577385	1.82222854	1.62308761
H	-2.06173160	-0.30694744	3.66047808
H	-1.68897953	2.13456836	3.89301049
H	0.33482761	3.17046244	2.89491284
C	1.80954149	-0.87717171	1.15943268
C	1.55184373	-2.19395606	1.14699394
H	2.12293008	-2.97836176	0.66900779
C	-0.40988222	-2.02040955	2.40535227
O	0.46375643	-2.76033379	1.79540311
O	-1.41722884	-2.61468200	2.94643213
C	-1.62736763	-4.03121410	2.72572201
H	-2.56684363	-4.24542907	3.22724537
H	-1.70594781	-4.21650368	1.65353280
H	-0.80514560	-4.59595907	3.16600270
In	3.57809770	-0.25961091	-0.03819083
I	4.86678249	1.95630925	1.01782544
I	5.27581035	-2.42808957	-0.07805552
I	2.72717865	0.31196028	-2.60894518
C	-3.46463196	2.89545253	0.96820365
C	-2.23597315	2.65563911	0.32358622
C	-1.31567656	3.73268981	0.25458977
C	-1.60292003	4.99571693	0.81712240
C	-2.81011264	5.18685633	1.44809668
C	-3.74144768	4.13106435	1.51701064
H	-4.19550635	2.09739093	1.04229030
H	-0.87445832	5.79533878	0.74509883
H	-3.04954100	6.14930517	1.88624068
H	-4.69435259	4.29212797	2.01047077
C	-1.88789164	1.37069845	-0.24624197
C	-0.68286740	1.26621285	-0.82675295
H	-0.23553829	0.40463719	-1.30667063
C	-0.08067042	3.48781101	-0.40127339

O	0.20674894	2.32967896	-0.90455096
O	0.80964258	4.41675495	-0.51891064
C	2.05326900	4.13452744	-1.21898764
H	2.57934840	5.08480358	-1.22825139
H	2.62034211	3.38077437	-0.67068595
H	1.83500826	3.78888505	-2.22927120
In	-3.24876883	-0.36803798	-0.44058983
I	-5.04214457	0.29350357	-2.42012777
I	-1.62323404	-2.48673381	-1.15807714
I	-4.65101878	-0.99365254	1.86321469

9

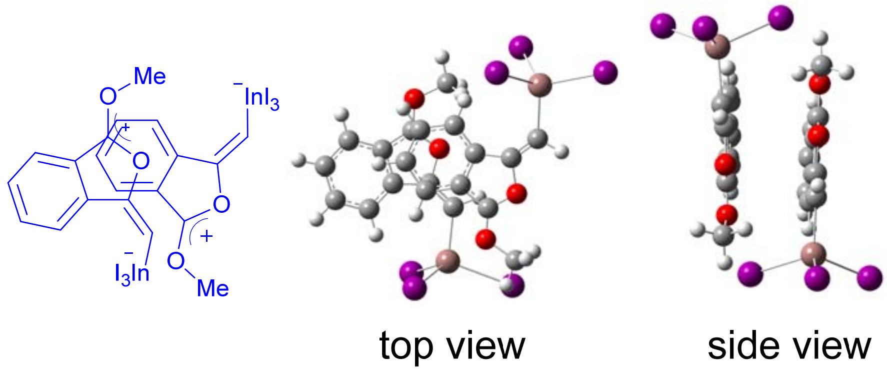

G (348K) = -54076.72079

C	-3.99374327	2.99446828	0.38898842
C	-2.80669694	2.75889257	-0.30018851
C	-1.91778203	3.81938565	-0.52925843
C	-2.16021694	5.12645454	-0.10110222
C	-3.34391547	5.34865523	0.58360256
C	-4.24231550	4.29290156	0.82149838
H	-4.70981385	2.20828193	0.59039413
H	-1.45178707	5.92382693	-0.29518742
H	-3.58425561	6.34403801	0.93968058
H	-5.16196647	4.49586543	1.36002431
C	-2.22415990	1.54463171	-0.85952955

C	-2.56237136	0.26683358	-0.95202462
H	-1.83337723	-0.36575691	-1.45974765
C	-0.80307365	3.24079569	-1.21136108
O	-0.94653213	1.96696678	-1.40912132
O	0.24566034	3.86073682	-1.59643557
C	1.31382120	3.11163674	-2.24861415
H	2.11176519	3.83473316	-2.38711193
H	1.63338078	2.29916007	-1.59745060
H	0.95193670	2.73014746	-3.20320728
In	-4.42036079	-0.71781403	-0.27301768
I	-6.57302613	0.48317942	-1.50007730
I	-4.15928489	-3.35599607	-1.01926650
I	-4.64465241	-0.45949385	2.47943330
C	1.89888637	1.57951926	1.09345194
C	1.35107284	0.29710241	1.12020732
C	0.04040345	0.11817258	1.58480718
C	-0.76134040	1.16336504	2.04817740
C	-0.20047651	2.42988880	2.03088374
C	1.11000266	2.62766440	1.55590034
H	2.90811613	1.76511887	0.74625245
H	-1.77382523	0.98124799	2.39526472
H	-0.77462879	3.27900340	2.38578919
H	1.52386842	3.63115651	1.55732513
C	1.89145930	-1.00800334	0.75742492
C	3.04569655	-1.49015939	0.31944874
H	3.06480346	-2.57063481	0.17583756
C	-0.21211071	-1.28711948	1.48513697
O	0.80410923	-1.94046453	1.01630074
O	-1.29420456	-1.87474986	1.81746267
C	-1.38533733	-3.32609951	1.74241494
H	-2.40281393	-3.54659920	2.05207164
H	-1.21513210	-3.64561469	0.71494512
H	-0.65304033	-3.75804503	2.42451010

In	4.95652301	-0.44829079	-0.08187868
I	5.69606184	1.01371961	2.14193988
I	6.78024199	-2.41032826	-0.67786047
I	4.60623967	1.25305830	-2.24708035

TS2-6-*endo* (Fig. 3)

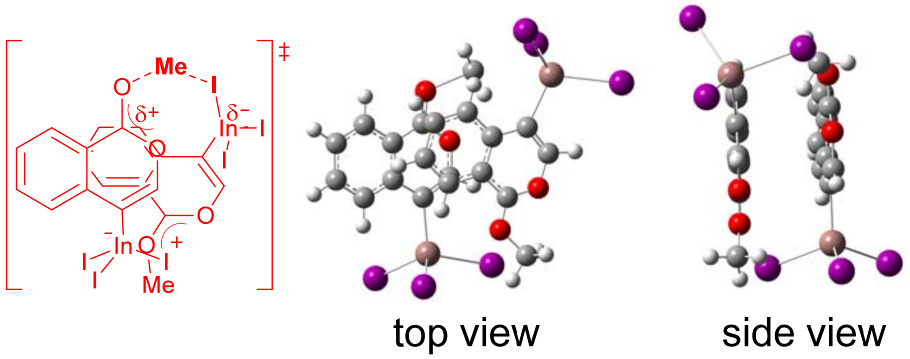

G (348K) = -54076.71146

C	1.82077253	1.78727958	1.38212622
C	1.49970341	0.42409608	1.25912012
C	0.20944705	0.02066856	1.69016197
C	-0.72179389	0.93500758	2.22487692
C	-0.36679915	2.26074927	2.33177262
C	0.90719804	2.68117003	1.91002036
H	2.80026084	2.13796971	1.07573741
H	-1.70591942	0.58887874	2.52561468
H	-1.07520813	2.98230879	2.72362274
H	1.17726399	3.72891457	1.99214104
C	2.42136549	-0.56693768	0.72901997
C	1.98662816	-1.83498111	0.66890798
H	2.52434416	-2.70747977	0.32219300
C	-0.11501216	-1.35953699	1.58504730
O	0.72289827	-2.22471196	1.09385659
O	-1.25108741	-1.79830997	1.99241153
C	-1.54560864	-3.21987778	1.98968944

H	-2.58173038	-3.27463967	2.31299985
H	-1.43563269	-3.61613491	0.98130137
H	-0.87835756	-3.71828122	2.69372355
In	4.53383458	-0.27589638	0.11047069
I	5.84486051	1.73718431	1.41547900
I	5.80265307	-2.69518458	0.15649875
I	4.41751121	0.49059959	-2.63886310
C	-3.59672647	3.04385621	0.41771617
C	-2.56736744	2.40770576	-0.29845367
C	-1.44398933	3.18336081	-0.65789715
C	-1.35047219	4.54302849	-0.31760232
C	-2.37692970	5.14001393	0.38776325
C	-3.50085325	4.38252059	0.75491265
H	-4.47712948	2.48079472	0.71009864
H	-0.46883573	5.10272952	-0.61002374
H	-2.31728323	6.18929198	0.65577486
H	-4.30795978	4.85149239	1.30840960
C	-2.61143994	0.99723270	-0.65310148
C	-1.54943745	0.50218284	-1.30000491
H	-1.41831285	-0.51413120	-1.65128975
C	-0.33569382	2.54462289	-1.33721922
O	-0.42884336	1.24616199	-1.62780772
O	0.71791581	3.12429840	-1.64582560
C	2.30513511	1.97822201	-2.14146568
H	2.92109307	2.76562158	-1.73688038
H	1.93378006	1.18807508	-1.50999817
H	2.02479873	1.99562911	-3.18342835
In	-4.29514213	-0.35461483	-0.22989048
I	-6.60329216	0.59533693	-1.39014868
I	-3.59467523	-2.82157151	-1.28835763
I	-4.67662203	-0.53594587	2.51875302

TS2-5-*exo* (Fig. 3)

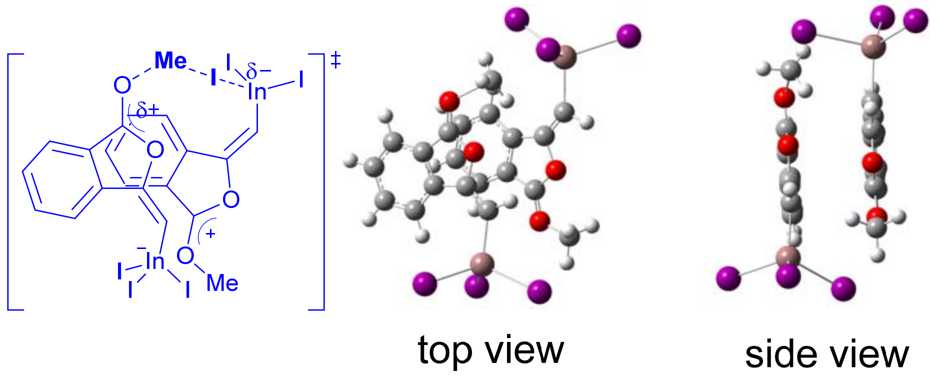

G (348K) = -54076.68577

C	3.11808247	2.94691695	-0.10570844
C	1.98623675	2.40973893	0.50206799
C	0.91686690	3.24228955	0.83307672
C	0.91644608	4.61092264	0.58326410
C	2.04698525	5.14208258	-0.02493681
C	3.13002514	4.31514886	-0.36087097
H	3.96867047	2.33870503	-0.38602255
H	0.06618496	5.22817830	0.85211881
H	2.09834660	6.20397897	-0.23972192
H	4.00412521	4.75213965	-0.83238709
C	1.62529980	1.04046418	0.87731624
C	2.23297058	-0.13917355	0.82008082
H	1.64804224	-0.97434490	1.20912783
C	-0.11765766	2.39544625	1.40502459
O	0.29974365	1.12705702	1.40913170
O	-1.23092237	2.71619413	1.82527891
C	-2.54573798	1.28215335	2.28987957
H	-3.30515170	1.94904689	1.91058102
H	-2.05054458	0.57765189	1.63843996
H	-2.26955990	1.32137079	3.33291189
In	4.31348326	-0.58069367	0.27228401
I	6.00978712	0.85516743	1.90699767

I	4.61598012	-3.30385511	0.65114651
I	4.90442087	0.05326473	-2.37483974
C	-1.69425663	1.96419103	-1.59680562
C	-1.09347528	0.70937299	-1.59691145
C	0.20345009	0.56502015	-2.11195743
C	0.94453055	1.62559137	-2.63367102
C	0.33345929	2.87018866	-2.63283122
C	-0.96484261	3.02895340	-2.12112981
H	-2.69443968	2.11862241	-1.20984560
H	1.95469433	1.47505980	-3.00016360
H	0.86639010	3.73271445	-3.01653957
H	-1.41537094	4.01611844	-2.12753334
C	-1.55406183	-0.60228169	-1.14308203
C	-2.65224168	-1.14571965	-0.63489638
H	-2.55503103	-2.21754407	-0.45293603
C	0.52438323	-0.82141132	-1.95913855
O	-0.43582474	-1.49236781	-1.39842912
O	1.60032360	-1.38713553	-2.33376639
C	1.78968631	-2.81455371	-2.11803361
H	2.79117548	-3.01135310	-2.48995717
H	1.72646190	-3.02796585	-1.05098648
H	1.03397841	-3.36102464	-2.68201894
In	-4.67682495	-0.47129168	-0.10086427
I	-5.52440283	2.07945220	-0.63186288
I	-6.43583719	-2.39675620	-0.88929913
I	-4.41537603	-0.55210514	2.74357879

10

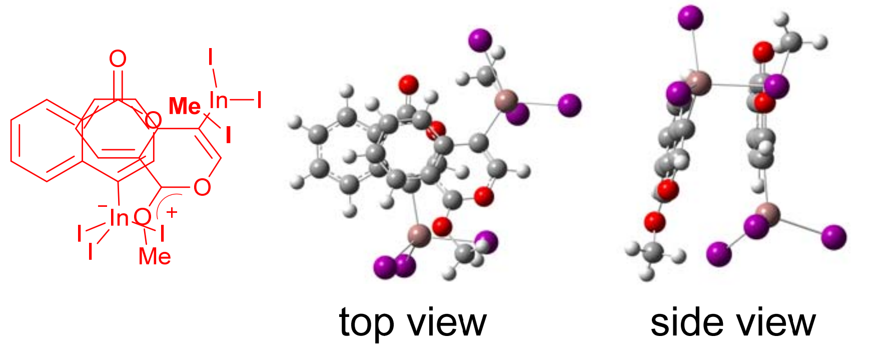

G (348K) = -54076.74228

C	1.09681822	1.59584402	2.16747000
C	0.80833403	0.22631572	2.05609560
C	-0.37678395	-0.24443996	2.67769046
C	-1.26421742	0.62762644	3.34235258
C	-0.95623601	1.96709847	3.41489793
C	0.23419193	2.44303195	2.83799932
H	1.99823208	1.99717059	1.71671743
H	-2.17780369	0.23619140	3.77399408
H	-1.63182277	2.65533596	3.90954802
H	0.47417414	3.49923402	2.90298189
C	1.65620186	-0.71177585	1.34415932
C	1.29167327	-2.00377406	1.35349574
H	1.79740179	-2.84071554	0.89102345
C	-0.63703232	-1.63872915	2.61903526
O	0.16863723	-2.46289466	2.01459990
O	-1.67030270	-2.14195820	3.19502683
C	-1.99927911	-3.54397081	3.02242904
H	-2.95056273	-3.66035353	3.53365064
H	-2.09998413	-3.75713243	1.95735232
H	-1.22356241	-4.15798883	3.48073365
In	3.53234597	-0.28613932	0.28873702
I	4.93518985	1.98222505	0.77306709
I	4.99635377	-2.58290068	0.11217790

I	2.84907705	0.03294299	-2.59969194
C	-3.18189367	3.10876019	0.68517200
C	-1.96893706	2.68290986	0.11760453
C	-0.92226813	3.62249628	0.01814617
C	-1.08131597	4.93811577	0.47674479
C	-2.28248192	5.32987177	1.04076299
C	-3.33450038	4.40776047	1.14015966
H	-4.00694997	2.40901324	0.77707106
H	-0.25073627	5.62967084	0.38261701
H	-2.41268472	6.34613752	1.39754410
H	-4.28044368	4.71334038	1.57628722
C	-1.75274112	1.32030053	-0.34992112
C	-0.54348025	1.03795240	-0.85265683
H	-0.22874631	0.07652640	-1.24235940
C	0.37219451	3.22695281	-0.53938892
O	0.49409598	1.93385117	-0.94725063
O	1.35015991	3.94075295	-0.65697935
C	3.25237071	2.15022541	-2.80580945
H	4.32439875	2.27381045	-2.67751129
H	2.67381882	2.66399904	-2.03883191
H	2.92680343	2.41004984	-3.81054912
In	-3.26982412	-0.25030047	-0.46737438
I	-5.04081290	0.27551887	-2.51344061
I	-1.85554788	-2.60256991	-1.01581171
I	-4.73330761	-0.64525751	1.86221159

11

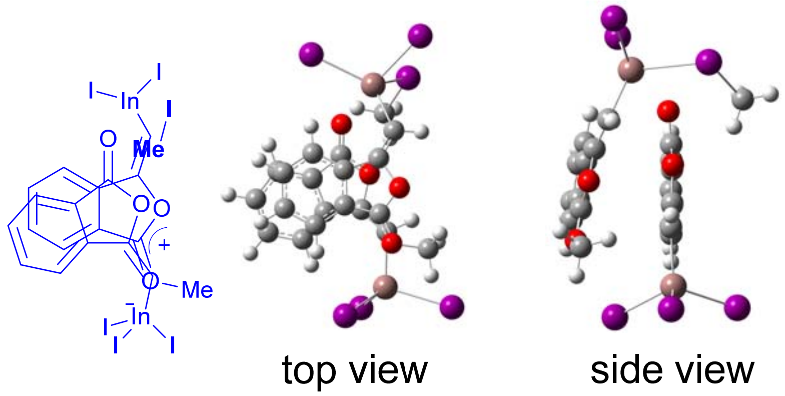

G (348K) = -54076.71927

C	2.13249773	2.43626433	1.49240019
C	1.07396530	1.54853117	1.31428857
C	-0.22475608	1.94019164	1.64254770
C	-0.52857782	3.19751719	2.15383190
C	0.53132735	4.07622999	2.34090869
C	1.84128770	3.69463838	2.01125937
H	3.15384019	2.17533942	1.24383404
H	-1.55197636	3.47126056	2.38791793
H	0.34923810	5.06679849	2.74371414
H	2.65247986	4.39892102	2.16409114
C	1.00853147	0.18156009	0.78898797
C	1.88928490	-0.67832023	0.28631249
H	1.45931792	-1.63433801	-0.01811040
C	-1.10846313	0.83684256	1.28670439
O	-0.36458814	-0.18844792	0.85008170
O	-2.33325372	0.77444381	1.31316353
C	-2.43731356	-1.53861022	3.36796023
H	-2.67245077	-0.51594512	3.64919413
H	-1.47726337	-1.61627994	2.86438375
H	-2.50135193	-2.21695612	4.21565837
In	4.07037658	-0.52562571	0.15679037
I	5.12859915	-0.18938108	2.68320920

I	4.88416283	-2.91153617	-0.98676643
I	4.92592925	1.57638717	-1.45027244
C	-1.55396439	2.69231969	-1.33367392
C	-0.88393901	1.57988941	-1.83092800
C	0.44823018	1.70132438	-2.25250180
C	1.17217397	2.88940444	-2.15639564
C	0.50317243	3.98529298	-1.63159679
C	-0.84154610	3.88498432	-1.24101368
H	-2.58865924	2.64586178	-1.01344111
H	2.21110123	2.93915019	-2.46147931
H	1.02202085	4.93090375	-1.52352502
H	-1.34152790	4.76241140	-0.84422604
C	-1.28717686	0.18701921	-1.99241644
C	-2.30211886	-0.56918729	-1.60306117
H	-2.22013713	-1.62303285	-1.86858931
C	0.81799503	0.40112513	-2.72299572
O	-0.15378354	-0.45719142	-2.62561944
O	1.95201796	0.07236141	-3.20073939
C	2.20791567	-1.30866912	-3.58226629
H	3.24967641	-1.31605636	-3.88884075
H	2.05866378	-1.95207969	-2.71493676
H	1.54345905	-1.58198965	-4.40176932
In	-4.00715024	-0.12776274	-0.30013025
I	-5.28937761	2.21536550	0.26170125
I	-5.96478186	-1.80738536	-1.38328744
I	-3.94866036	-2.17321139	1.96531351

MeI

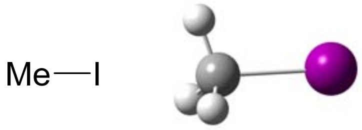

G (348K) = -6959.906859

C	0.00000000	0.00000000	-1.82938170
H	-0.00000000	1.03616638	-2.15883534
H	-0.89734640	-0.51808319	-2.15883534
H	0.89734640	-0.51808319	-2.15883534
I	-0.00000000	0.00000000	0.32929804

4a

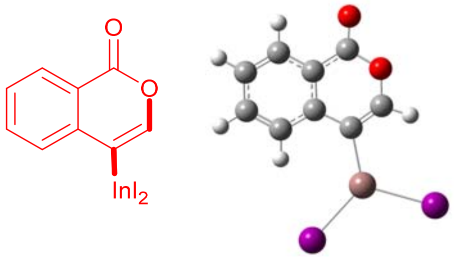

G (348K) = -20078.45556

C	-2.45495742	1.65823453	-0.00007335
C	-2.53995895	0.25715463	-0.00003172
C	-3.82139191	-0.32975113	0.00002105
C	-4.97714258	0.46221517	0.00011053
C	-4.86921045	1.84251177	0.00013876
C	-3.60150438	2.43721656	0.00001382
H	-1.48305746	2.14203223	-0.00024642
H	-5.94509710	-0.02707864	0.00016605
H	-5.76124484	2.45953347	0.00019945
H	-3.51181887	3.51888591	-0.00000195
C	-1.37387643	-0.61995068	-0.00000906
C	-1.59126020	-1.94985110	0.00006120
H	-0.82041641	-2.71330490	0.00009569
C	-3.97611624	-1.79091804	-0.00008589
O	-2.81699693	-2.53003369	0.00014943
O	-5.02216067	-2.39487405	-0.00032882
In	0.68960515	-0.03394012	0.00006397
I	2.51203733	-2.01879105	-0.00001073

I	1.67054294	2.46937765	-0.00004181

12

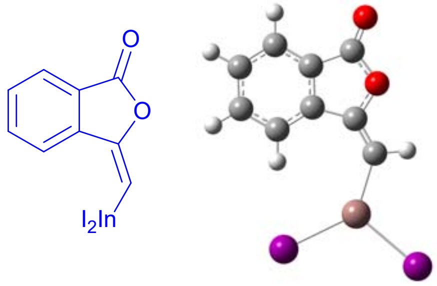

G (348K) =-20078.44805

C	2.73506422	1.21389570	-0.00011660
C	3.00126929	-0.15125096	-0.00000415
C	4.31690179	-0.60740264	0.00002897
C	5.40637022	0.25313006	-0.00004099
C	5.14013721	1.61999271	-0.00015633
C	3.81996734	2.08872697	-0.00019365
H	1.72558206	1.60644179	-0.00013457
H	6.42018804	-0.13231379	-0.00002058
H	5.95975261	2.33064717	-0.00022577
H	3.63534407	3.15810079	-0.00028891
C	2.12803859	-1.33696553	0.00003134
C	0.80518684	-1.53149084	0.00003218
H	0.50131263	-2.57755657	0.00002670
C	4.28434291	-2.08113248	0.00007897
O	2.95997440	-2.45231990	0.00005496
O	5.17081233	-2.89263035	0.00014433
In	-0.87160799	-0.21593838	0.00000583
I	-3.25325207	-1.48558888	-0.00005108
I	-1.09398098	2.46955578	0.00006626

13

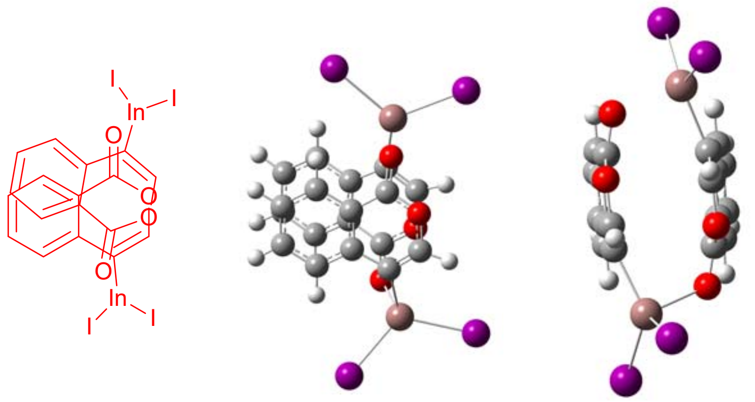

top view side view

G (348K) = -40156.95109

C	1.46469646	-2.41418174	1.22103571
C	0.89986858	-1.15026202	1.46420667
C	-0.47941804	-1.09418473	1.76713083
C	-1.26844620	-2.25705791	1.81391401
C	-0.68549251	-3.48308248	1.56826210
C	0.68484650	-3.55553499	1.27081732
H	2.52197724	-2.49471139	0.98782499
H	-2.32602130	-2.17579496	2.04310863
H	-1.28437554	-4.38646533	1.59938484
H	1.14039649	-4.52033908	1.07251197
C	1.65428944	0.08940822	1.36444237
C	1.01058237	1.21907138	1.70052072
H	1.41968058	2.22168931	1.70432466
C	-1.10577880	0.19583186	1.92298200
O	-0.32744850	1.27041299	2.02859857
O	-2.33767008	0.39039117	1.92050152
In	3.49140032	0.28913786	0.19075421
I	4.21867605	2.86491954	-0.24862017
I	5.48389024	-1.54874728	0.27794147
C	-1.89199680	-2.48470399	-1.80023906

C	-1.07254426	-1.35381940	-1.64559602
C	0.30402121	-1.49579166	-1.93907285
C	0.84487957	-2.72470054	-2.35329059
C	0.01088790	-3.81311469	-2.51095818
C	-1.35989306	-3.68598522	-2.23403613
H	-2.95192782	-2.41120361	-1.58073012
H	1.90982614	-2.79517828	-2.54638858
H	0.41241884	-4.76389973	-2.84400058
H	-2.01316552	-4.54423921	-2.35503265
C	-1.57345773	-0.06515877	-1.18955809
C	-0.70956641	0.96315396	-1.21905337
H	-0.91856062	1.99771654	-0.97369744
C	1.17674790	-0.36623592	-1.72542294
O	0.62597769	0.82950109	-1.52662330
O	2.42000945	-0.43047475	-1.67216684
In	-3.41666800	0.34981914	-0.07520287
I	-4.27018286	2.91280628	-0.27574986
I	-5.28227847	-1.56967702	0.36664916

ClBcat

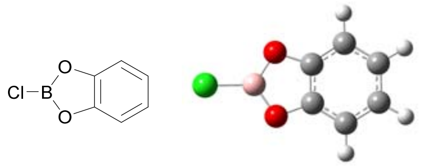

G (398K) = -866.496499

C	0.00002762	-2.93340575	0.69877474
C	0.00002762	-2.93340575	-0.69877474
C	-0.00006850	-1.74066198	-1.43116877
C	-0.00014468	-0.57357197	-0.69504361
C	-0.00014468	-0.57357197	0.69504361
C	-0.00006850	-1.74066198	1.43116877
H	-0.00001820	-3.87865829	1.23078317
H	-0.00001820	-3.87865829	-1.23078317

H	-0.00004732	-1.72824354	-2.51469694
H	-0.00004732	-1.72824354	2.51469694
O	-0.00006850	0.73020479	1.14650366
O	-0.00006850	0.73020479	-1.14650366
B	0.00002306	1.49230197	-0.00000000
Cl	0.00019638	3.23768787	-0.00000000

14

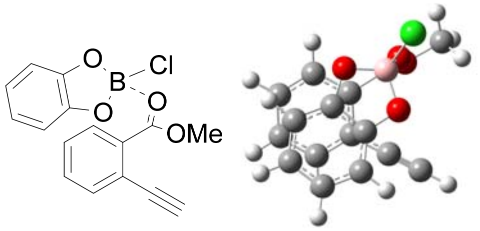

G (398K) = -1402.491756

C	1.99675522	2.16073011	0.19578563
C	0.67108386	1.82117384	0.48935552
C	-0.18477315	1.47989613	-0.56990180
C	0.26193758	1.48980643	-1.88781834
C	1.57689509	1.84730640	-2.16215797
C	2.44125638	2.17604436	-1.12004931
H	2.66985705	2.40363743	1.00992627
H	-0.40917677	1.20289907	-2.68976881
H	1.92886153	1.85356601	-3.18783256
H	3.47263812	2.43608252	-1.33257875
C	0.20292236	1.80022386	1.84320975
C	-0.20201098	1.77628431	2.98060086
H	-0.55565856	1.74262200	3.98745976
C	-1.58845324	1.08696608	-0.28031808
O	-2.02706586	-0.07843372	-0.24760180
O	-2.39394014	2.08194306	-0.07279251
C	-3.76909473	1.78394184	0.27134098
H	-4.23250307	2.75508737	0.42175549

H	-4.24154476	1.24150080	-0.54739308
H	-3.79130399	1.18759139	1.18347510
B	-1.18479986	-1.49154521	-0.19449654
Cl	-2.53982939	-2.70656580	-0.44474270
O	-0.16993124	-1.50018894	-1.20587937
O	-0.55058633	-1.52279374	1.09111548
C	2.29463807	-1.44274768	-1.05807855
C	3.35856634	-1.40454766	-0.14378128
C	3.12948740	-1.40689465	1.23060607
C	1.82685400	-1.45319125	1.75121034
C	0.78954267	-1.49258593	0.84257809
C	1.01902807	-1.48400451	-0.53528367
H	2.46039988	-1.43259270	-2.12957432
H	4.37696433	-1.37083598	-0.51771116
H	3.97170002	-1.37540723	1.91453881
H	1.63524402	-1.44942933	2.81828812

TS1-6-*endo* (Fig. 5)

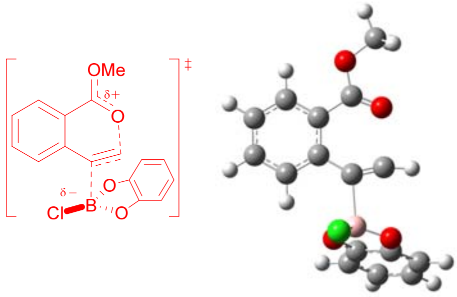

G (398K) = -1402.456404

C	1.10252217	2.03125896	-0.55888200
C	1.35696151	0.72285067	-0.13898527
C	2.63513388	0.15822018	-0.36349921
C	3.61784674	0.93117761	-0.99353774
C	3.35090301	2.23135599	-1.40235994
C	2.09030180	2.78034991	-1.18573462
H	0.12058807	2.45710528	-0.39839749

H	4.59703888	0.50299033	-1.16522961
H	4.12640703	2.81206492	-1.89015282
H	1.87221812	3.79416493	-1.50440003
C	0.32406232	-0.06130215	0.52177836
C	0.17267237	-1.21838830	0.93732116
H	-0.20428002	-2.12121799	1.37020578
C	2.97823703	-1.23938956	0.03767883
O	2.21107094	-2.07363976	0.49851729
O	4.26315209	-1.52390851	-0.16605426
C	4.68841462	-2.85018740	0.17471734
H	5.75058859	-2.87838206	-0.05939890
H	4.52244946	-3.03873113	1.23673674
H	4.14278927	-3.58609125	-0.41848346
C	-4.66996454	-1.76009093	-1.02439018
C	-4.41565189	-0.83953304	-2.03972482
C	-3.48341783	0.19498747	-1.86792096
C	-2.83368795	0.25404205	-0.65229156
C	-3.08657799	-0.66835028	0.36456029
C	-4.00335439	-1.68808526	0.20829089
H	-5.39723826	-2.54887301	-1.18679513
H	-4.94705300	-0.92135443	-2.98222843
H	-3.28055588	0.91901329	-2.64902775
H	-4.19861659	-2.39535862	1.00667243
O	-2.32098291	-0.37531174	1.45887813
O	-1.90171213	1.16203959	-0.23136838
B	-1.45596617	0.67566572	1.02999113
Cl	-0.98724316	1.89643582	2.28874337

TS1-5-*exo* (Fig. 5)

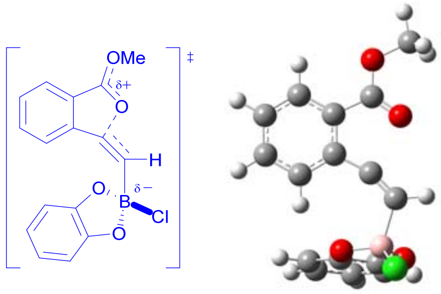

G (398K) = -1402.466799

C	-0.81167649	1.20712378	-1.55761172
C	-1.42682568	0.30629983	-0.68049633
C	-2.72904447	0.55544499	-0.22187327
C	-3.41519004	1.70081356	-0.61251247
C	-2.79658674	2.60356424	-1.47049089
C	-1.50559076	2.35157424	-1.93701746
H	0.19323055	1.01047984	-1.91034709
H	-4.42230993	1.87225834	-0.24986324
H	-3.32139111	3.49956978	-1.78278175
H	-1.02961666	3.05438730	-2.61248535
C	-0.74931138	-0.87354139	-0.23369458
C	0.14258056	-1.73835179	-0.04337023
H	0.09419069	-2.69483904	0.45734099
C	-3.26606685	-0.50077474	0.65304825
O	-2.59451640	-1.50133351	0.87840792
O	-4.47020356	-0.28493040	1.14350774
C	-5.01302611	-1.31062070	1.99482565
H	-5.99737675	-0.95007455	2.28402697
H	-5.08967088	-2.24938302	1.44436384
H	-4.37591473	-1.44319895	2.87034238
C	3.75583930	1.71810159	2.16345177
C	3.40607159	2.55256712	1.10369540
C	2.78708374	2.04260065	-0.04846119
C	2.54082768	0.68469434	-0.08125469

C	2.88983026	-0.15286361	0.98210562
C	3.50138374	0.33846568	2.11853709
H	4.23478560	2.13928687	3.04149173
H	3.61567427	3.61555003	1.16603174
H	2.51808383	2.68206800	-0.88231497
H	3.77278571	-0.31963529	2.93651353
O	2.54547079	-1.43838509	0.69745577
O	1.96509201	-0.04349123	-1.07884282
B	1.81532790	-1.37656699	-0.54648772
Cl	2.19425630	-2.71171174	-1.76043499

15

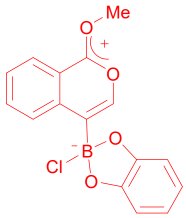

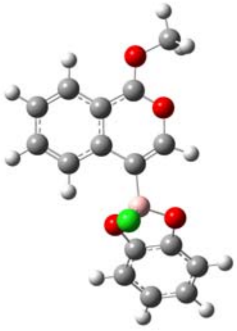

G (398K) = -1402.505073

C	1.17402518	2.13593781	-0.13814207
C	1.46226673	0.76040327	-0.03199724
C	2.78825468	0.34056491	-0.31017137
C	3.79773322	1.26037305	-0.67759142
C	3.48025717	2.59365477	-0.76841732
C	2.16466101	3.02553719	-0.49752086
H	0.16737889	2.47951656	0.06610303
H	4.80118538	0.90583776	-0.88227472
H	4.23905032	3.31574647	-1.04835369
H	1.92744565	4.08160549	-0.57357552
C	0.46181222	-0.21857333	0.33807138
C	0.84568458	-1.50292144	0.38514644

H	0.23165028	-2.35677537	0.62823806
C	3.06231737	-1.04387377	-0.20468621
O	2.14733251	-1.90391576	0.11865822
O	4.25244855	-1.51059903	-0.43359867
C	4.49577381	-2.93139439	-0.31707474
H	5.54970835	-3.04851777	-0.55314769
H	4.28654947	-3.25930705	0.70145407
H	3.87460245	-3.47217002	-1.03155581
C	-5.20373328	-1.16253784	-0.80581898
C	-5.09709187	0.04112296	-1.49722290
C	-3.94412283	0.83760930	-1.39288015
C	-2.92441156	0.38001642	-0.58139644
C	-3.03140639	-0.83188287	0.11367016
C	-4.16164310	-1.61990898	0.01863802
H	-6.10662079	-1.75809521	-0.89868422
H	-5.91738848	0.37521511	-2.12491687
H	-3.85265174	1.77892608	-1.92428976
H	-4.23724697	-2.55344594	0.56611349
O	-1.90203666	-1.06853580	0.82696218
O	-1.72248775	0.95955461	-0.33793866
B	-1.07969504	0.13425877	0.68922218
Cl	-1.05820603	1.09666915	2.37885155

16

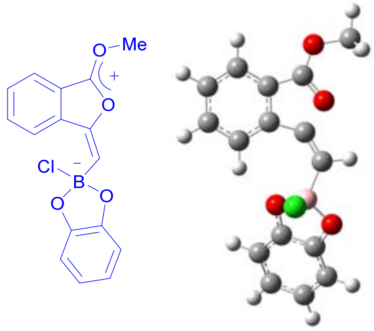

G (398K) = -1402.492722

C	-1.13368522	1.87336514	-0.29389720
C	-1.76410901	0.65384106	-0.03540186
C	-3.13911347	0.62724802	0.25610098
C	-3.93014504	1.78175682	0.30741121
C	-3.29533088	2.98195184	0.05143713
C	-1.91716249	3.01768886	-0.24596178
H	-0.07348909	1.91382976	-0.51109700
H	-4.98878391	1.72915162	0.53433263
H	-3.85870453	3.90793065	0.07617527
H	-1.45177821	3.97755552	-0.44485187
C	-1.27535306	-0.71187765	-0.02484127
C	-0.12146628	-1.32727563	-0.27140826
H	-0.14662515	-2.41506014	-0.21694576
C	-3.45663757	-0.74726705	0.44350774
O	-2.42860305	-1.52282422	0.29105894
O	-4.61285267	-1.23127770	0.73199245
C	-4.75298592	-2.66828897	0.88233173
H	-5.80335460	-2.81937462	1.11344120
H	-4.48192096	-3.15947599	-0.05215270
H	-4.11792838	-3.01003255	1.69943433
C	5.49706971	-0.26870181	1.09388021
C	5.02767831	1.00752734	1.39411201
C	3.71248908	1.38845674	1.07882048
C	2.90938600	0.44969082	0.46175315
C	3.38020121	-0.83467946	0.16028446
C	4.67191564	-1.21713422	0.46694764
H	6.51845949	-0.53801292	1.34431698
H	5.68631080	1.72338214	1.87599556
H	3.33745405	2.38068098	1.30662774
H	5.02921892	-2.21282050	0.22711662
O	2.40104673	-1.57167777	-0.41808707
O	1.61275646	0.57471794	0.07942926
B	1.29024168	-0.65249713	-0.65341311

Cl	1.15556749	-0.24968149	-2.55946672

MeCl

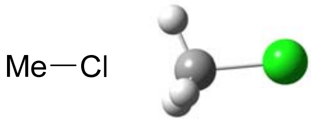

G (398K) = -500.077735

C	0.00000000	0.00000000	-1.13428000
H	0.00000000	1.03298100	-1.47752500
H	-0.89458800	-0.51649100	-1.47752500
H	0.89458800	-0.51649100	-1.47752500
Cl	0.00000000	0.00000000	0.66107400

17

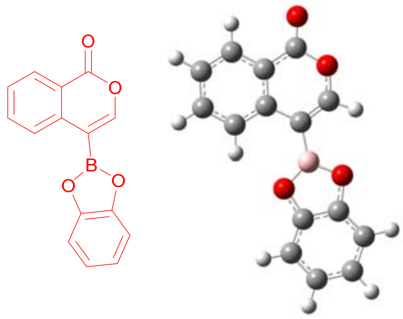

G (398K) = -902.474681

C	-1.45433157	1.82279024	-0.00022462
C	-1.71560168	0.44240116	-0.00014717
C	-3.05949441	0.01965046	-0.00008439
C	-4.10909393	0.94786264	-0.00009949
C	-3.83074435	2.30313782	-0.00020573
C	-2.49870063	2.73437181	-0.00025119
H	-0.42958872	2.17403218	-0.00027048
H	-5.12991829	0.58196747	-0.00004088
H	-4.63916430	3.02625455	-0.00022406

H	-2.27726965	3.79702721	-0.00025931
C	-0.66663831	-0.57662781	-0.00000241
C	-1.05578260	-1.86874037	-0.00005690
H	-0.37369329	-2.71040847	0.00016117
C	-3.39743912	-1.40883570	0.00022278
O	-2.33785099	-2.28951802	-0.00027729
O	-4.50887904	-1.88048767	0.00075636
C	5.35969371	-0.42078693	-0.00007684
C	5.17344591	0.96398470	0.00029655
C	3.89257387	1.52898377	0.00043464
C	2.83333389	0.64429637	0.00021006
C	3.01778885	-0.73348923	-0.00017183
C	4.27443033	-1.30507190	-0.00035416
H	6.36740526	-0.82234639	-0.00016432
H	6.03893843	1.61789276	0.00050013
H	3.73631040	2.60145469	0.00073376
H	4.40816878	-2.38056806	-0.00066586
O	1.79123932	-1.35169145	-0.00035296
O	1.48441020	0.91645786	0.00029044
B	0.85736314	-0.32139074	-0.00000765

18

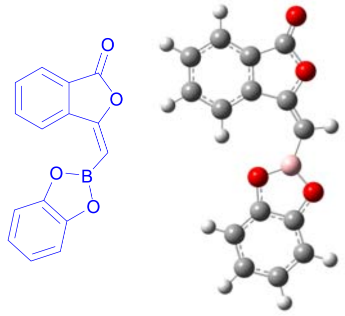

G (398K) = -902.469032

C	-1.42454198	1.52975139	-0.00022248
C	-2.02012433	0.27050276	0.00008149

C	-3.40904519	0.15418626	0.00009725
C	-4.25327869	1.25526810	-0.00026916
C	-3.65900892	2.51426331	-0.00064793
C	-2.26471025	2.64237891	-0.00061941
H	-0.34871185	1.64411171	-0.00011601
H	-5.33056785	1.13029076	-0.00025074
H	-4.27854709	3.40470530	-0.00097093
H	-1.82480423	3.63445484	-0.00090909
C	-1.47463637	-1.09821164	0.00016716
C	-0.24428825	-1.63154887	-0.00005347
H	-0.22729367	-2.71957017	-0.00027777
C	-3.74573550	-1.27973052	0.00030929
O	-2.55583916	-1.96867782	0.00040867
O	-4.80625317	-1.84516270	0.00040989
C	4.99668131	1.36757148	0.00056324
C	5.52142399	0.07226532	-0.00033772
C	4.69038913	-1.05385357	-0.00084216
C	3.33029270	-0.81376014	-0.00040696
C	2.80972089	0.47536576	0.00048503
C	3.61571151	1.59607247	0.00099941
H	5.67240868	2.21616947	0.00093496
H	6.59739548	-0.06586102	-0.00065294
H	5.08720603	-2.06236414	-0.00154169
H	3.19837339	2.59637147	0.00171331
O	1.43653249	0.40348207	0.00072399
O	2.29748616	-1.71591565	-0.00075103
B	1.13320605	-0.95424832	-0.00001652

19

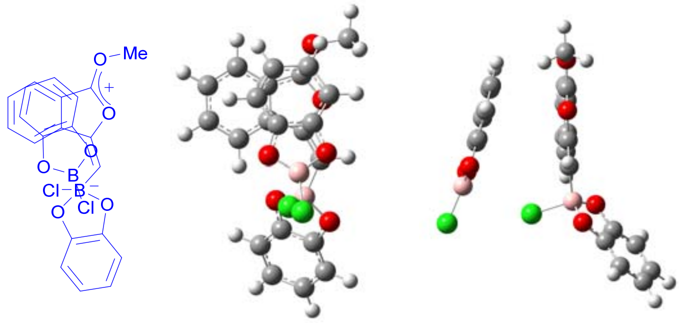

top view side view

G (398K) = -2268.977289

C	-0.15516900	-1.27029100	1.88386400
C	-0.68761100	-1.60377800	0.63650700
C	-1.87051300	-2.35605200	0.56148200
C	-2.55985800	-2.80783400	1.69221400
C	-2.02595000	-2.47090400	2.92092400
C	-0.84212300	-1.70881500	3.00652700
H	0.75808200	-0.69437200	1.96325300
H	-3.47320900	-3.38467900	1.60141500
H	-2.51977900	-2.79081700	3.83173900
H	-0.45367100	-1.45671300	3.98787700
C	-0.27056300	-1.28620900	-0.71633400
C	0.74506400	-0.64133000	-1.28308000
H	0.71198000	-0.56105400	-2.36871100
C	-2.16340900	-2.45490300	-0.82689900
O	-1.28383900	-1.85791700	-1.56870000
O	-3.17129300	-3.05618700	-1.35424800
C	-3.32731900	-3.01125900	-2.79512700
H	-4.25269100	-3.54534100	-2.99051500
H	-3.39590000	-1.97185500	-3.11858200
H	-2.48014500	-3.50759100	-3.26826000
C	6.55863200	-0.67184600	-0.49139000
C	6.24002200	-1.34869500	0.68329600

C	4.90688300	-1.47455600	1.10836100
C	3.93052800	-0.90117800	0.31756200
C	4.25015000	-0.22123600	-0.86475100
C	5.55831100	-0.09277800	-1.28980600
H	7.59677500	-0.58486100	-0.79661000
H	7.03195700	-1.78428200	1.28462400
H	4.64791100	-1.99811800	2.02259300
H	5.79642300	0.43859000	-2.20499400
O	3.12241600	0.22575400	-1.47016200
O	2.58658400	-0.90496100	0.50770900
B	2.03431700	-0.03507200	-0.53456800
Cl	1.46576700	1.64145800	0.29411600
C	-4.04756100	0.13514100	0.59985400
C	-4.00945000	0.17676000	-0.79614700
C	-3.17047000	1.06658100	-1.47920100
C	-2.40258700	1.90674500	-0.69857200
C	-2.44712900	1.87011800	0.69286900
C	-3.25332400	0.98400800	1.37875800
H	-4.70867400	-0.56899300	1.09390700
H	-4.65942700	-0.48165600	-1.36442200
H	-3.13868600	1.11680400	-2.56152800
H	-3.27760800	0.96276800	2.46183500
O	-1.63491800	2.85406600	1.19586200
O	-1.55701700	2.91087800	-1.09778000
B	-1.09946200	3.46931700	0.08045200
Cl	-0.11826100	4.90923400	0.15000500

TS2-5-*exo* (Fig. 5)

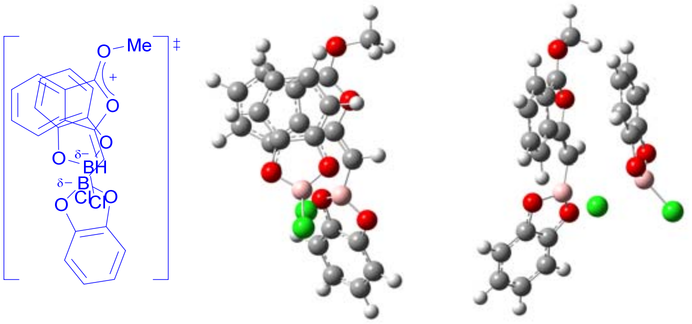

top view side view

G (398K) = -2268.967956

C	-0.34945700	-1.26387100	1.84625200
C	-0.95741400	-1.48109900	0.60974800
C	-2.26473400	-1.98810800	0.55429600
C	-3.01237900	-2.29434700	1.69453000
C	-2.40303500	-2.07158200	2.91527100
C	-1.09167300	-1.56181700	2.98137500
H	0.65780200	-0.87397500	1.91413400
H	-4.02435100	-2.67488700	1.61822100
H	-2.93788300	-2.28569300	3.83378500
H	-0.64512000	-1.39262800	3.95560000
C	-0.51176300	-1.24897000	-0.75523100
C	0.57526500	-0.76564700	-1.34685400
H	0.52644000	-0.66867700	-2.42976200
C	-2.58562600	-2.05184700	-0.83155600
O	-1.61410600	-1.64295900	-1.58936700
O	-3.68302600	-2.47759400	-1.34240400
C	-3.85131300	-2.41705100	-2.78246100
H	-4.85510300	-2.79204500	-2.95904800
H	-3.75359300	-1.38185600	-3.11136700
H	-3.10477200	-3.04961900	-3.26249300
C	6.44785000	-0.87731000	-0.41937000
C	6.09960900	-1.61020400	0.71478800

C	4.75696200	-1.80013900	1.07370400
C	3.80304600	-1.22946300	0.25500800
C	4.15112800	-0.49653500	-0.88207800
C	5.46947400	-0.30311300	-1.24420600
H	7.49545900	-0.74317100	-0.66900100
H	6.87956600	-2.03888400	1.33584600
H	4.47506500	-2.36553600	1.95511400
H	5.72959300	0.27204400	-2.12576400
O	3.02452800	-0.06846400	-1.51942100
O	2.44405200	-1.28032800	0.37293200
B	1.95069900	-0.40189400	-0.64525100
Cl	1.53733000	1.41108500	0.47172500
C	-4.16586200	0.84459900	0.66133100
C	-4.07404300	0.75003000	-0.72741600
C	-2.95652100	1.24358500	-1.41870900
C	-1.96354100	1.83495900	-0.66285600
C	-2.06926100	1.95265500	0.72565700
C	-3.15447900	1.45162400	1.41807500
H	-5.04073500	0.44950800	1.16750800
H	-4.88955500	0.30031200	-1.28563100
H	-2.87751500	1.18920800	-2.49908700
H	-3.22311400	1.54543200	2.49578200
O	-1.00682700	2.65129000	1.20815000
O	-0.81676700	2.43298000	-1.08954300
B	-0.18386400	2.89992900	0.08541300
Cl	0.73428100	4.43613900	0.01455600

20

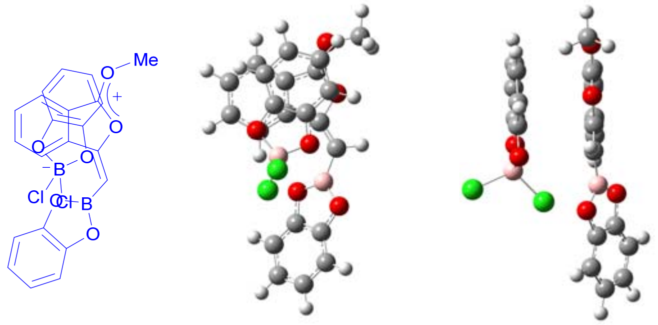

top view side view

G (398K) = -2268.980676

C	-0.37366400	-0.79069900	1.86383600
C	-1.15076900	-1.27723200	0.81797900
C	-2.53493300	-1.43957300	0.98750500
C	-3.19248200	-1.13868100	2.18018700
C	-2.41080200	-0.65919500	3.21787800
C	-1.02646300	-0.48375800	3.05282900
H	0.68932800	-0.62559400	1.75065800
H	-4.26552900	-1.25551100	2.27638600
H	-2.87148100	-0.39848600	4.16395200
H	-0.44599400	-0.08037800	3.87536100
C	-0.82831600	-1.65333800	-0.55511300
C	0.25754700	-1.70273200	-1.32156000
H	0.09259300	-1.95690800	-2.36605000
C	-3.02267000	-1.88138000	-0.27563200
O	-2.07232700	-2.01686000	-1.15820100
O	-4.23168400	-2.17609400	-0.57070400
C	-4.55096600	-2.54563200	-1.93847500
H	-5.62626700	-2.69654300	-1.93879900
H	-4.26632100	-1.72844700	-2.60169100
H	-4.02326400	-3.46301400	-2.19801800
C	6.12294600	-0.77386700	-0.35246300
C	5.82321600	-1.22153200	0.93758600

C	4.52425200	-1.59468500	1.29834700
C	3.56500300	-1.49644100	0.31106900
C	3.86271300	-1.05306400	-0.97227400
C	5.13893700	-0.67954300	-1.34197800
H	7.14090200	-0.48554800	-0.59182700
H	6.61303600	-1.27418000	1.67928100
H	4.27806100	-1.93311400	2.29802800
H	5.35725300	-0.32150800	-2.34106300
O	2.71511400	-1.05889000	-1.72909600
O	2.22157300	-1.78506700	0.38573200
B	1.72350000	-1.46673300	-0.86417700
Cl	2.03175200	1.54785600	0.64049500
C	-3.97369000	1.50970700	-0.06043300
C	-3.66263200	1.04346800	-1.33588100
C	-2.33847700	1.05726300	-1.81177900
C	-1.36151900	1.54817500	-0.96729700
C	-1.67748400	2.02447800	0.31787000
C	-2.97651400	2.01234700	0.79084400
H	-5.00528700	1.49843800	0.27818600
H	-4.45988400	0.69708800	-1.98776000
H	-2.08630700	0.71065000	-2.80858100
H	-3.20914500	2.38719100	1.78152500
O	-0.56732500	2.45759700	0.94173400
O	-0.03798000	1.65402500	-1.19383800
B	0.51026200	2.40229900	-0.05453500
Cl	0.99302100	4.14778200	-0.58986600

21

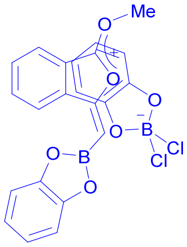

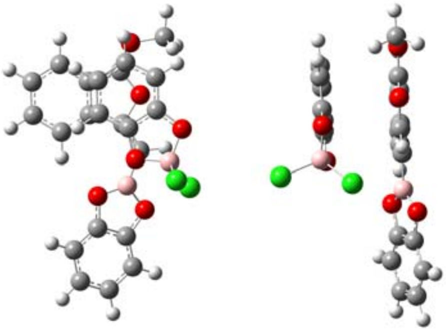

top view side view

G (398K) = -2268.975308

C	0.39114400	-2.91366100	-0.77336000
C	1.11854200	-2.11348500	0.10371600
C	2.50514500	-2.28799800	0.21793400
C	3.21617500	-3.23791700	-0.51445100
C	2.48436700	-4.03140800	-1.38280900
C	1.09395400	-3.86526500	-1.50524300
H	-0.67555800	-2.78079400	-0.89830200
H	4.29060200	-3.33838900	-0.41329200
H	2.98603100	-4.78412400	-1.98019400
H	0.55088800	-4.49639900	-2.20083100
C	0.73457800	-1.02530300	0.99795300
C	-0.38299600	-0.41944600	1.38859300
H	-0.26568900	0.36981000	2.12720400
C	2.93988600	-1.29020700	1.14473500
O	1.95306600	-0.57662200	1.59807500
O	4.13351500	-1.09604000	1.55276900
C	4.39183700	-0.00019400	2.47714400
H	5.46898300	-0.00724100	2.61349200
H	4.05099400	0.92899400	2.02025800
H	3.87213000	-0.19513600	3.41506100
C	-6.24899600	-1.15502600	0.25839200
C	-5.80005600	-1.65851000	-0.96624700

C	-4.43648300	-1.73389200	-1.26987200
C	-3.56912000	-1.28702400	-0.29362400
C	-4.01420400	-0.78461900	0.92349400
C	-5.35659000	-0.70317300	1.23621300
H	-7.31479300	-1.10660900	0.45432300
H	-6.52394800	-1.99263000	-1.70174200
H	-4.07756100	-2.11133600	-2.22024300
H	-5.69387400	-0.30192500	2.18455400
O	-2.93011400	-0.41614700	1.68205000
O	-2.19274500	-1.24550600	-0.31769700
B	-1.83028600	-0.68693000	0.89447400
Cl	-1.53771800	2.76369700	1.11002000
C	3.10479700	0.23441300	-1.82392500
C	3.86219900	1.07527800	-1.01361700
C	3.25450700	2.09434600	-0.25525300
C	1.88118200	2.22000800	-0.33565800
C	1.11571300	1.36481500	-1.14952000
C	1.70724300	0.37054000	-1.90445600
H	3.59688800	-0.53510000	-2.41081600
H	4.94170700	0.96072200	-0.98085600
H	3.83856900	2.77917400	0.35136400
H	1.10831500	-0.27707400	-2.53573900
O	-0.19112400	1.67049400	-1.04865000
O	1.08541900	3.10351700	0.29369900
B	-0.26205800	2.90967800	-0.25846300
Cl	-0.72775500	4.36236800	-1.37161300

22

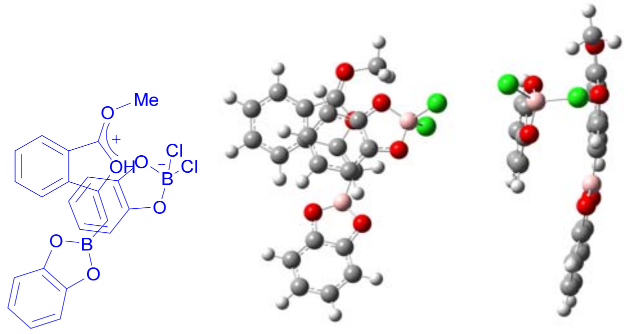

top view side view

G (398K) = -2268.984968

C	-1.09484800	3.12836000	-0.06818000
C	0.04555400	2.44397400	-0.47859700
C	1.30644400	3.04154300	-0.32999700
C	1.48986600	4.30187600	0.23741400
C	0.34864500	4.97421600	0.64657500
C	-0.91983900	4.39135300	0.48907700
H	-2.07713800	2.68567700	-0.16360600
H	2.47908300	4.73038000	0.35042000
H	0.43247200	5.95876100	1.09245100
H	-1.79363300	4.94320000	0.81953300
C	0.25607800	1.11698100	-1.05306100
C	-0.45651200	0.02368100	-1.31788800
H	0.11905200	-0.81960200	-1.69942300
C	2.24592200	2.07744100	-0.81946800
O	1.65851900	1.01632400	-1.28129600
O	3.51458900	2.17727000	-0.82033400
C	4.31227100	1.02405200	-1.25784800
H	5.33912500	1.33261200	-1.08833100
H	4.03027800	0.16220200	-0.65177700
H	4.11535000	0.84437500	-2.31401200
C	-6.03709700	-1.92502200	-0.22547700
C	-6.26805700	-0.60617400	0.17748200

C	-5.26266900	0.36465600	0.11370400
C	-4.03864500	-0.05593100	-0.36676400
C	-3.80820000	-1.36676200	-0.76871000
C	-4.79130600	-2.33511300	-0.71162500
H	-6.84161600	-2.64949500	-0.15892500
H	-7.24795000	-0.32762100	0.55008500
H	-5.43109500	1.38906300	0.42505500
H	-4.59997900	-3.35477000	-1.02452300
O	-2.50916500	-1.48517700	-1.19503400
O	-2.88225900	0.67229700	-0.53383900
B	-1.95141500	-0.23521600	-1.01261500
Cl	2.62726000	-2.23820600	-1.69578700
C	-0.96833400	-0.19052300	2.34151800
C	0.02729900	0.76327900	2.52149600
C	1.33607200	0.54163600	2.05399200
C	1.58821900	-0.64592600	1.39706500
C	0.58616200	-1.61399100	1.22361200
C	-0.69880200	-1.40861600	1.69074900
H	-1.97069000	0.00013700	2.71222000
H	-0.20215900	1.69294700	3.03294500
H	2.12250400	1.27525500	2.20471000
H	-1.46600100	-2.16312400	1.55286800
O	1.06827900	-2.67321500	0.54555500
O	2.73798200	-1.05160600	0.81385900
B	2.45358500	-2.36044900	0.20270900
Cl	3.64266000	-3.65819200	0.82567800

TS3-5-*exo* (Fig. 5)

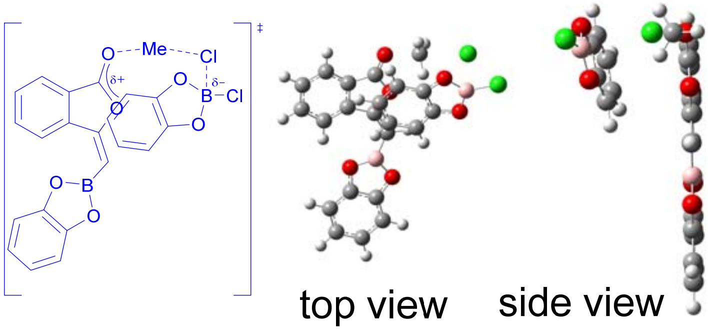

top view side view

G (398K) = -2268.951872

C	-2.63694300	2.55591400	0.30850800
C	-1.50030900	2.06423400	-0.32683300
C	-0.36742300	2.87380200	-0.45314000
C	-0.30761500	4.17362600	0.03316800
C	-1.44625800	4.66291900	0.66356900
C	-2.58833600	3.86163200	0.79501000
H	-3.52482400	1.94828800	0.42318400
H	0.58987700	4.77193400	-0.07572500
H	-1.45252800	5.67217100	1.05977100
H	-3.46201400	4.26833800	1.29343600
C	-1.17359400	0.76592600	-0.92772600
C	-1.78069200	-0.41106300	-1.09674100
H	-1.17805200	-1.16672200	-1.59634800
C	0.64646200	2.07056900	-1.11287000
O	0.14752200	0.87303900	-1.40401000
O	1.81484900	2.37033200	-1.38242900
C	2.95368100	1.05362000	-1.78538400
H	2.85103800	1.08450400	-2.85927600
H	2.46142600	0.26324200	-1.24264300
H	3.79936500	1.54585200	-1.33379600
C	-7.05869100	-2.99379500	0.17826500
C	-7.37333500	-1.75217300	0.73878700
C	-6.46011800	-0.69230600	0.72763000

C	-5.23981500	-0.94574900	0.13417600
C	-4.92572400	-2.17950100	-0.42288900
C	-5.81706400	-3.23431900	-0.41939500
H	-7.79348300	-3.79111000	0.20663800
H	-8.34698500	-1.60519100	1.19356900
H	-6.69420100	0.27371400	1.15958600
H	-5.56295500	-4.19281400	-0.85640300
O	-3.65070700	-2.13120300	-0.93294300
O	-4.16278300	-0.09941900	-0.01765100
B	-3.19939100	-0.84887700	-0.67477100
C	0.87289000	0.17451900	1.70715100
C	1.70208400	1.26801000	1.96515900
C	3.09239500	1.18253000	1.80928400
C	3.59250700	-0.03508100	1.39493400
C	2.76392000	-1.12641800	1.13399300
C	1.39282600	-1.05351300	1.27581700
H	-0.19926300	0.27379700	1.84619900
H	1.26313700	2.20330000	2.29794100
H	3.74648500	2.02418600	2.00527400
H	0.75692300	-1.90599200	1.06678900
O	3.52986100	-2.19299400	0.74789500
O	4.89475900	-0.39322800	1.17328700
B	4.82608900	-1.70394400	0.72779300
Cl	6.23078800	-2.71807000	0.50473800
Cl	4.59153300	-0.81227700	-2.16646300

23

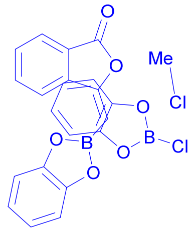

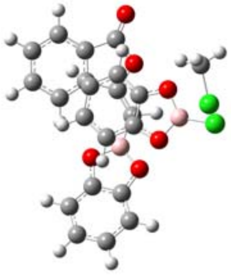

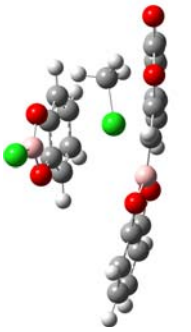

top view side view

G (398K) = -2269.02233

C	3.15728000	1.63519500	0.14054900
C	2.95217700	0.35565800	-0.36982300
C	3.91850900	-0.63148800	-0.18986700
C	5.09955400	-0.40657400	0.50438500
C	5.30042900	0.86860200	1.02444800
C	4.34038600	1.87181900	0.83747200
H	2.42088500	2.41648400	0.00782200
H	5.83005900	-1.19827000	0.63044800
H	6.20827600	1.09179800	1.57463800
H	4.52275100	2.86044000	1.24651400
C	1.83648800	-0.26089500	-1.10427100
C	0.61597700	0.13361800	-1.48601400
H	0.02660600	-0.62897500	-1.99135300
C	3.43625600	-1.86290200	-0.83640300
O	2.21166800	-1.57177400	-1.38480800
O	3.92583300	-2.95815800	-0.92533300
C	-0.43719300	-3.80400200	-1.48318900
H	-0.39419100	-4.66427000	-2.14808800
H	0.50401400	-3.25711500	-1.49480900
H	-0.69895700	-4.10413600	-0.47053500
C	-3.16853500	4.67974500	-0.44901000
C	-2.02713600	5.34971600	-0.00011500

C	-0.76369700	4.74797800	-0.04106100
C	-0.71259300	3.46468100	-0.54763100
C	-1.84767000	2.79766800	-0.99703900
C	-3.10008000	3.37873300	-0.96072200
H	-4.13127100	5.17719500	-0.39925700
H	-2.11991300	6.35762100	0.39016200
H	0.12780100	5.25826300	0.30527800
H	-3.97918300	2.84783300	-1.30686200
O	-1.49350700	1.54733100	-1.42803900
O	0.37641900	2.63983000	-0.68881900
B	-0.12571700	1.44592400	-1.19950600
C	0.32606200	0.81673900	2.16362800
C	1.07975000	-0.35611300	2.24750300
C	0.51523100	-1.60896000	1.98356000
C	-0.82186000	-1.61559300	1.64186900
C	-1.57087700	-0.44772500	1.55130200
C	-1.02831400	0.79436700	1.81238200
H	0.80173500	1.77048200	2.36527700
H	2.12949100	-0.29499900	2.51548600
H	1.08954400	-2.52598300	2.04892600
H	-1.62336200	1.69882900	1.74731400
O	-2.86589200	-0.76572700	1.20954300
O	-1.63554400	-2.69451200	1.37135400
B	-2.86997500	-2.13909300	1.10586300
Cl	-4.29768700	-3.08014000	0.75955500
Cl	-1.73104800	-2.70320500	-2.07930000

S4

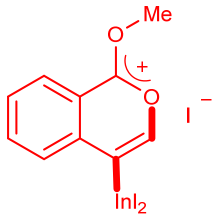

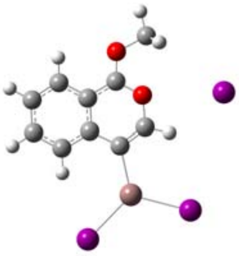

G (348K) = -27038.31476

C	-1.38555092	3.17675814	0.09110538
C	-0.13260305	2.53996905	0.05151879
C	1.01915140	3.36893057	0.03415760
C	0.92341477	4.77592240	0.05023418
C	-0.32139321	5.36258683	0.08546290
C	-1.47407763	4.55572347	0.10702033
H	-2.29205779	2.58162756	0.10791966
H	1.82666672	5.37508986	0.03504587
H	-0.41508497	6.44246000	0.09753004
H	-2.45241935	5.02399695	0.13660116
C	0.03211368	1.09775126	0.03037518
C	1.28447669	0.60538171	0.00323761
H	1.63515102	-0.42411792	-0.01197369
C	2.28442902	2.72009410	0.00126452
O	2.38572910	1.42823545	-0.00927828
O	3.38611111	3.38381512	-0.01885101
C	4.65290753	2.65396121	-0.05357448
H	5.40827229	3.43389559	-0.07291600
H	4.73523572	2.02803764	0.83445041
H	4.68763004	2.03001472	-0.94611972
In	-1.51612036	-0.42411615	0.01413479
I	4.35982857	-1.49054928	-0.04874151
I	-0.71430033	-2.96643338	0.08466928
I	-4.14204573	0.12965833	-0.09353004

TS2-6-*endo* (Fig. S3)

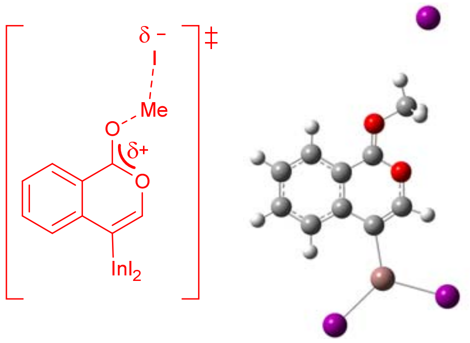

G (348K) = -27038.30217

C	-0.91000218	2.86716492	-0.06680010
C	-0.07195744	1.74311804	-0.12436318
C	1.32569873	1.96891594	-0.19261565
C	1.86722362	3.26954004	-0.19960036
C	1.01386371	4.35043488	-0.14090907
C	-0.37432323	4.14326222	-0.07516259
H	-1.98575998	2.73869673	-0.01455653
H	2.94166241	3.40406311	-0.24967847
H	1.41271327	5.35822996	-0.14477398
H	-1.04023189	4.99857764	-0.02950940
C	-0.56210555	0.37460223	-0.11687131
C	0.34189229	-0.61658867	-0.17874317
H	0.16068611	-1.68353281	-0.18296894
C	2.18070562	0.82921352	-0.25333985
O	1.69983721	-0.38603780	-0.24837488
O	3.44835248	0.95874305	-0.31445397
C	4.32677469	-0.21468117	-0.35685015
H	4.13563009	-0.76024730	-1.28051963
H	5.33968949	0.18156301	-0.33060858
H	4.13478550	-0.83161302	0.51999909
In	-2.62914399	-0.28894420	0.00034894
I	8.04921538	-0.57155130	0.17395106
I	-2.96268759	-2.94492906	-0.05732691

I	-4.75224965	1.32555573	0.19336986

S5

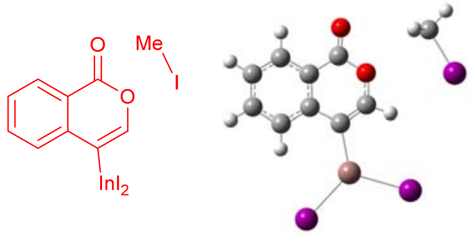

G (348K) = -27038.35565

C	-1.85679503	3.10470344	-0.00026657
C	-0.53304638	2.63755230	0.00044546
C	0.50650036	3.58996292	0.00060095
C	0.22684122	4.96296180	0.00001758
C	-1.08652787	5.40056048	-0.00070637
C	-2.12776188	4.46397229	-0.00083579
H	-2.68049397	2.39740861	-0.00038840
H	1.05405622	5.66448721	0.00014209
H	-1.30794275	6.46234918	-0.00117021
H	-3.15899676	4.80255918	-0.00139481
C	-0.17973282	1.22233477	0.00100323
C	1.12887052	0.90331114	0.00172592
H	1.53886661	-0.10155158	0.00212740
C	1.91145213	3.16236344	0.00136209
O	2.13936712	1.80893294	0.00198710
O	2.87811624	3.88871475	0.00147671
C	5.28051672	1.02623724	0.00773631
H	6.36753020	1.01478424	0.00802675
H	4.88144093	1.49447595	0.90296772
H	4.88189574	1.50409938	-0.88258399
In	-1.50977441	-0.45915154	0.00053143
I	4.61401467	-1.02974982	-0.00337551
I	-0.36622511	-2.89761111	0.00234374

I	-4.19796274	-0.39650939	-0.00176019

In_2Cl_6

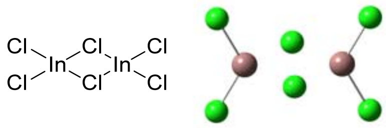

G (348K) = -14245.46456

In	1.85286730	0.00002982	0.00008381
In	-1.85292436	0.00006185	-0.00008263
Cl	2.90945685	2.08361044	-0.00000472
Cl	2.90875553	-2.08376229	0.00023384
Cl	-0.00014873	0.00011058	1.73592661
Cl	0.00000215	-0.00007022	-1.73592576
Cl	-2.90864042	-2.08330485	0.00002640
Cl	-2.90926091	2.08315213	-0.00025975

S2 (Fig. S3)

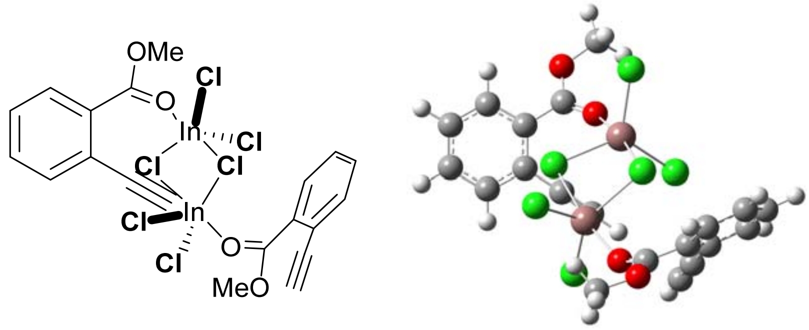

G (348K) = -15317.49469

C	-4.42120384	2.74942428	1.16706501
C	-4.18846179	1.37015445	1.09638681
C	-4.82432630	0.63005787	0.07719413
C	-5.67588315	1.26996933	-0.82889329
C	-5.88168727	2.64144876	-0.75337410
C	-5.25044927	3.37923967	0.24650007

H	-3.93612827	3.31967285	1.95118047
H	-6.15802972	0.68702133	-1.60536343
H	-6.52857349	3.13175600	-1.47249829
H	-5.40479687	4.45120597	0.31196612
C	-3.35050416	0.77156021	2.09103733
C	-2.64530404	0.33052550	2.96636316
H	-2.00885733	-0.08275342	3.71679897
C	-4.57990738	-0.81725292	-0.08564612
O	-3.51859452	-1.39038211	0.18607049
O	-5.60176013	-1.48636210	-0.56020009
C	-5.40283002	-2.89216010	-0.82541472
H	-6.33995064	-3.22970847	-1.26115598
H	-4.57194378	-3.02491077	-1.51972283
H	-5.19601307	-3.41402116	0.10963854
In	1.32731109	1.40053477	-0.22029557
In	-1.32740361	-1.40061211	-0.21998987
C	4.42154330	-2.74928729	1.16686208
C	4.18867196	-1.37004038	1.09612339
C	4.82444255	-0.62993752	0.07687518
C	5.67604656	-1.26981767	-0.82919063
C	5.88198462	-2.64127331	-0.75360527
C	5.25083114	-3.37907335	0.24631533
H	3.93653856	-3.31954120	1.95101741
H	6.15812280	-0.68686392	-1.60570004
H	6.52890624	-3.13155452	-1.47271532
H	5.40528101	-4.45102175	0.31183543
C	3.35069945	-0.77149087	2.09078792
C	2.64549626	-0.33052490	2.96614531
H	2.00899999	0.08267298	3.71658361
C	4.57987384	0.81733966	-0.08604823
O	3.51851489	1.39037156	0.18568984
O	5.60164789	1.48653244	-0.56064895
C	5.40256739	2.89229983	-0.82592417

H	6.33966298	3.22993435	-1.26165236
H	4.57168595	3.02492461	-1.52026292
H	5.19566325	3.41417590	0.10910102
Cl	1.34156789	-1.01661729	-0.71718790
Cl	1.64048336	2.73919041	-2.16547463
Cl	0.79695371	2.40643767	1.86205670
Cl	-0.79684761	-2.40640866	1.86235632
Cl	-1.64066262	-2.73941172	-2.16506215
Cl	-1.34173578	1.01648930	-0.71725945

S3

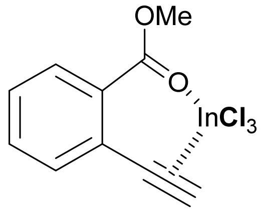

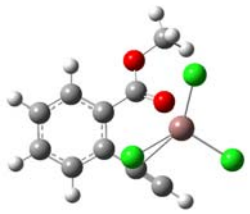

G (348K) = -7658.749536

C	3.35569991	1.66453060	-0.16146831
C	2.28434065	0.87462215	-0.59489144
C	2.26619552	-0.49189648	-0.24156186
C	3.30717117	-1.03643876	0.51739462
C	4.35233837	-0.23290475	0.95358112
C	4.37256268	1.11852146	0.61316930
H	3.37665463	2.71263831	-0.43733748
H	3.28019818	-2.08807897	0.77886650
H	5.14575321	-0.65868510	1.55765340
H	5.18628925	1.75212614	0.94982134
C	1.28388797	1.47718138	-1.42429091
C	0.47669911	2.03426887	-2.12987004
H	-0.25577978	2.51255709	-2.74251162
C	1.14697432	-1.36590901	-0.63596359
O	-0.02870890	-0.99234930	-0.78438653

O	1.46919084	-2.61644402	-0.82657626
C	0.39795175	-3.54341168	-1.11841051
H	0.87425698	-4.52009533	-1.14239963
H	-0.36010500	-3.48986543	-0.33605701
H	-0.04114630	-3.29805177	-2.08577432
In	-1.47958117	0.24701517	0.26090441
Cl	-0.30276082	1.66806268	1.74796510
Cl	-2.84710449	1.15730430	-1.44344035
Cl	-2.47915987	-1.59873901	1.39518083

TS (A to B) (Fig. S5)

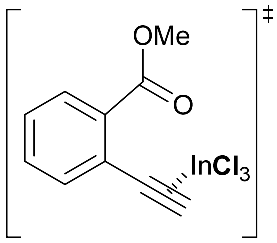

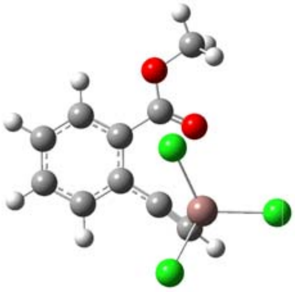

G (348K) = -7658.733342

C	1.78271610	-2.49710629	0.68719253
C	1.55218679	-1.12349619	0.84185413
C	2.47446973	-0.18708365	0.32935029
C	3.61629508	-0.65337158	-0.31589900
C	3.84903648	-2.02097190	-0.45009884
C	2.93336706	-2.94334818	0.04787405
H	1.05311933	-3.20249452	1.06954167
H	4.32284260	0.06181381	-0.71886484
H	4.74641956	-2.36380344	-0.95397875
H	3.10794267	-4.00748674	-0.06592291
C	0.35375596	-0.74567019	1.52437786
C	-0.71237036	-0.53939464	2.08223835
H	-1.50704740	-0.35882422	2.77954905

C	2.21319644	1.28055096	0.46137692
O	1.34649579	1.75504155	1.16609799
O	3.03916745	2.00755795	-0.28568444
C	2.82033558	3.42528871	-0.26353404
H	3.56741224	3.84235490	-0.93572549
H	1.81244190	3.65036935	-0.61718122
H	2.95049024	3.81186731	0.74896683
In	-1.64656383	0.00774569	-0.16200790
Cl	-3.07111107	1.70810462	0.67142296
Cl	-0.13976067	0.64129854	-1.87902212
Cl	-2.65708312	-2.10743339	-0.56179490

S6

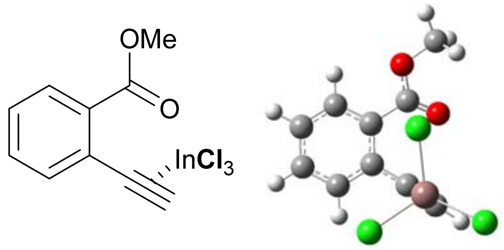

G (348K) = -7658.735502

C	1.38020653	2.46705525	-0.73328162
C	1.41362258	1.07380800	-0.88367389
C	2.46701648	0.32339775	-0.31970890
C	3.46349527	0.98879192	0.38707995
C	3.43089537	2.37622151	0.52238352
C	2.39216976	3.11570295	-0.03573567
H	0.55079483	3.02563936	-1.15234892
H	4.26919832	0.41561328	0.82980284
H	4.21998262	2.87813659	1.07216633
H	2.36218073	4.19356875	0.07759331
C	0.32458221	0.46472366	-1.57885314
C	-0.68997298	0.03198531	-2.10577229

H	-1.40527139	-0.36468121	-2.79970896
C	2.51987014	-1.16091572	-0.50244060
O	1.96103847	-1.75217527	-1.40195127
O	3.26435465	-1.75108869	0.42912589
C	3.34533165	-3.18107487	0.35450676
H	3.97993900	-3.47762693	1.18710725
H	3.78545460	-3.48630167	-0.59675530
H	2.34849457	-3.61435963	0.45586539
In	-1.68461525	-0.08953774	0.16250332
Cl	-3.57565667	-1.31198153	-0.58334586
Cl	-2.06851008	2.18548760	0.72386572
Cl	-0.21766110	-1.23554737	1.63119743

TS1-6-*endo* (Fig. S5)

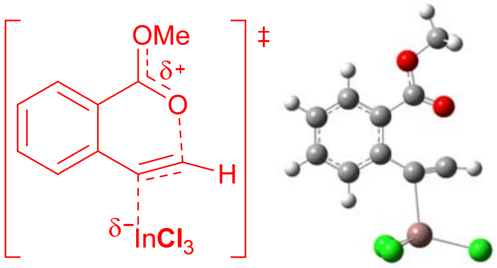

G (348K) = -7658.718612

C	-0.92495499	2.02704384	0.00041377
C	-1.36864287	0.70373557	0.00021106
C	-2.75584369	0.42565722	0.00018631
C	-3.65375789	1.49773679	0.00036990
C	-3.19982173	2.81227511	0.00057610
C	-1.83414042	3.07780222	0.00059667
H	0.13714165	2.24422194	0.00043447
H	-4.71709398	1.29471413	0.00035262
H	-3.91560661	3.62718343	0.00071927
H	-1.47171790	4.09989287	0.00075519

C	-0.42792087	-0.40236522	0.00002534
C	-0.37548495	-1.63799112	-0.00012817
H	-0.11712262	-2.67872895	-0.00026330
C	-3.29766724	-0.96987334	-0.00002627
O	-2.64121934	-2.00175030	-0.00009986
O	-4.62625182	-0.99182724	-0.00012419
C	-5.24787694	-2.28536910	-0.00030213
H	-6.31767567	-2.08745210	-0.00035795
H	-4.95740761	-2.84222452	-0.89281490
H	-4.95753427	-2.84240711	0.89213798
In	1.96086490	-0.14562968	-0.00009845
Cl	2.23996477	1.11029339	1.99969161
Cl	2.23969280	1.11078405	-1.99961727
Cl	2.98452336	-2.29299727	-0.00042036

TS1-5-*exo* (Fig. S5)

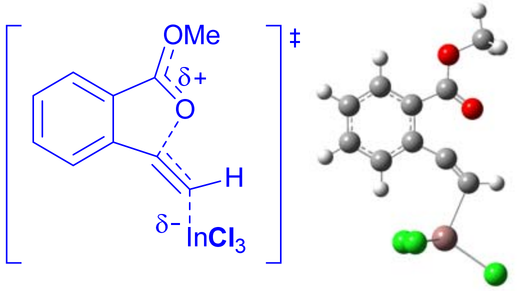

G (348K) = -7658.725983

C	1.15633731	1.84416178	0.00006494
C	1.71320143	0.56262777	0.00003919
C	3.10653102	0.40858590	0.00004124
C	3.95237218	1.51131752	0.00006729
C	3.39458633	2.78645977	0.00009147
C	2.00823265	2.94504760	0.00009045
H	0.08371865	1.99285545	0.00006488
H	5.02700097	1.36798933	0.00006867

H	4.03893542	3.65854036	0.00011163
H	1.57750209	3.94026427	0.00010975
C	0.90804963	-0.62864689	0.00001021
C	-0.11536332	-1.35545910	-0.00001199
H	-0.27601457	-2.42403830	-0.00002662
C	3.52119787	-1.00223970	0.00001446
O	2.65021948	-1.87131271	-0.00000577
O	4.81086383	-1.24091871	0.00001332
C	5.21793673	-2.62389290	-0.00001399
H	6.30495742	-2.59993399	-0.00001505
H	4.83957396	-3.12087103	0.89430375
H	4.83957130	-3.12083656	-0.89434973
In	-2.08669722	-0.17598934	-0.00002537
Cl	-3.69973214	-1.92704805	-0.00010305
Cl	-2.06345067	1.17356165	-1.97292183
Cl	-2.06353714	1.17346222	1.97293992

S7

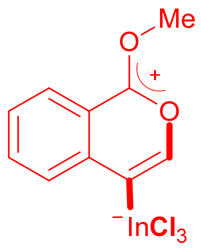

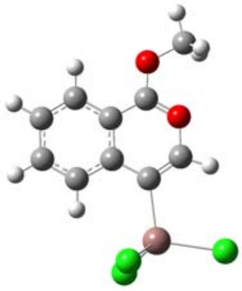

G (348K) = -7658.757528

C	-0.89599214	2.12295051	0.00015954
C	-1.32912546	0.78362241	0.00012752
C	-2.72869412	0.54745282	0.00007340
C	-3.66221898	1.60833819	0.00004591
C	-3.19881450	2.90310970	0.00007395
C	-1.81190392	3.15545464	0.00013072

H	0.16635620	2.34338222	0.00024542
H	-4.72443828	1.39260675	0.00001307
H	-3.89828156	3.73150169	0.00005213
H	-1.45698352	4.18069134	0.00015350
C	-0.40917300	-0.33404019	0.00012153
C	-0.93678117	-1.56545993	0.00005554
H	-0.41883598	-2.51486608	0.00004721
C	-3.15348584	-0.80514225	0.00001284
O	-2.31210261	-1.79255313	0.00001625
O	-4.41329512	-1.10717020	-0.00003832
C	-4.81893784	-2.49714442	-0.00002797
H	-5.90486942	-2.46408762	0.00037805
H	-4.44388384	-2.98944379	-0.89764548
H	-4.44321160	-2.98966875	0.89718183
In	1.79224301	-0.15906344	-0.00002712
Cl	2.47859509	1.05799400	1.96335507
Cl	2.61846975	-2.42175895	0.00057775
Cl	2.47812245	1.05711382	-1.96414213

S8

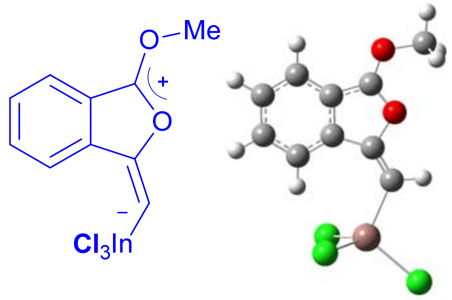

G (348K) = -7658.74245

C	1.16884871	1.84787943	-0.00004203
C	1.75353389	0.58318450	-0.00004700
C	3.15257366	0.46473218	-0.00002505
C	4.01189300	1.56788470	0.00000961

C	3.41939456	2.81866466	0.00001664
C	2.01781652	2.94827672	-0.00000839
H	0.09545449	1.98903697	-0.00006400
H	5.08872118	1.44347259	0.00002715
H	4.03669740	3.70986743	0.00004066
H	1.58261600	3.94197266	-0.00000526
C	1.17948936	-0.75769589	-0.00008388
C	-0.03488282	-1.28678481	-0.00012237
H	-0.07318376	-2.37539491	-0.00016174
C	3.41312548	-0.93845388	-0.00004566
O	2.33005278	-1.64955262	-0.00008362
O	4.56930267	-1.49324384	-0.00002604
C	4.65517966	-2.94455211	-0.00003781
H	5.72046252	-3.15595038	0.00000259
H	4.17593488	-3.33352770	0.89831190
H	4.17600732	-3.33350792	-0.89843486
In	-1.94975664	-0.20057408	-0.00001158
Cl	-3.70869717	-1.83542799	-0.00027962
Cl	-2.05384298	1.21929182	1.95219715
Cl	-2.05400751	1.22000204	-1.95167965

S9

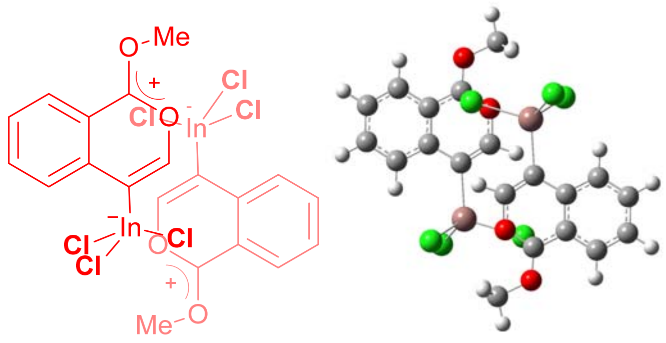

G (348K) = -15317.52832

C	4.15500790	-0.29021984	-0.93345028
C	3.18967826	-0.89882974	-0.11039763

C	3.24244390	-2.30733574	0.02582610
C	4.20876123	-3.08464054	-0.64495995
C	5.13638717	-2.45386485	-1.44259413
C	5.10723259	-1.05315275	-1.58032472
H	4.14990223	0.78631189	-1.06871935
H	4.20896865	-4.16197912	-0.52715722
H	5.88811339	-3.03353484	-1.96634753
H	5.84204465	-0.56339514	-2.21069737
C	2.16075827	-0.14905269	0.58340674
C	1.31165478	-0.83677779	1.36048086
H	0.48714907	-0.46171093	1.95253671
C	2.26542054	-2.91281504	0.86087510
O	1.39084021	-2.21282429	1.51362127
O	2.23349222	-4.19231884	1.02258821
C	1.16593894	-4.78753843	1.80487467
H	1.33273512	-5.85761749	1.72061386
H	1.24469395	-4.45594523	2.84053903
H	0.20305029	-4.49759397	1.38351012
In	1.97729778	2.03127313	0.46556716
C	-4.15499749	0.29022100	0.93346044
C	-3.18966929	0.89883142	0.11040610
C	-3.24244022	2.30733718	-0.02582086
C	-4.20875753	3.08464089	0.64496687
C	-5.13637988	2.45386466	1.44260456
C	-5.10722568	1.05315225	1.58033158
H	-4.14988838	-0.78630998	1.06873424
H	-4.20896659	4.16197933	0.52716297
H	-5.88810477	3.03353382	1.96636076
H	-5.84203332	0.56339377	2.21070877
C	-2.16074796	0.14905584	-0.58339850
C	-1.31164386	0.83678337	-1.36047034
H	-0.48713881	0.46171988	-1.95252927
C	-2.26541541	2.91281851	-0.86086555

O	-1.39082795	2.21283038	-1.51360367
O	-2.23349100	4.19232297	-1.02258235
C	-1.16590731	4.78754615	-1.80482605
H	-1.33280454	5.85762061	-1.72070544
H	-0.20304495	4.49773181	-1.38331217
H	-1.24451009	4.45582815	-2.84046130
In	-1.97730210	-2.03127319	-0.46557599
Cl	3.83646182	3.13639189	1.51232166
Cl	1.92917616	2.70886133	-1.86170794
Cl	-0.08746071	2.72662634	1.51794686
Cl	0.08747973	-2.72664201	-1.51789433
Cl	-1.92924715	-2.70889736	1.86169116
Cl	-3.83644841	-3.13635517	-1.51240107

S10

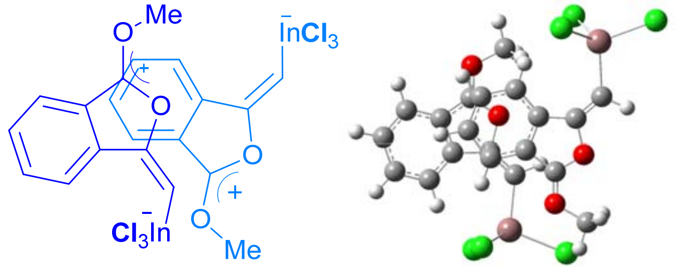

G (348K) = -15317.48645

C	-3.81803081	2.40504510	0.47685036
C	-2.61209211	2.29530575	-0.20997677
C	-1.74811116	3.39750942	-0.27636199
C	-2.02964916	4.62597010	0.32448343
C	-3.22984540	4.72307439	1.01037709
C	-4.10641157	3.62555774	1.07940748
H	-4.52156162	1.58494750	0.54955264
H	-1.33916755	5.45898329	0.25602398
H	-3.50119690	5.65311668	1.49689623
H	-5.04075616	3.73221077	1.62019519

C	-1.98475044	1.18123429	-0.91394370
C	-2.27734665	-0.08759625	-1.15547261
H	-1.53498436	-0.63457166	-1.73680805
C	-0.60750493	2.94403842	-1.01024025
O	-0.71862725	1.70708822	-1.38835208
O	0.43360134	3.63063552	-1.28004356
C	1.53452055	3.00032952	-2.00358274
H	2.30722838	3.76149590	-2.04620258
H	1.87935433	2.12985194	-1.44724733
H	1.19226467	2.72250111	-3.00010983
In	-4.05885851	-1.18003755	-0.48648087
C	1.89621031	1.09527817	1.26327712
C	1.41643896	-0.20027368	1.07386143
C	0.10707841	-0.51575451	1.46292149
C	-0.76515620	0.40611995	2.04571605
C	-0.27522311	1.68832697	2.23412355
C	1.03619178	2.02080804	1.84615988
H	2.90759634	1.38042948	0.99876330
H	-1.77666455	0.12382193	2.32151560
H	-0.90861973	2.44643612	2.68237818
H	1.39448507	3.03143967	2.01488547
C	2.03470151	-1.40499274	0.52838362
C	3.21732309	-1.74252579	0.03569373
H	3.31262645	-2.78218125	-0.27564344
C	-0.06465766	-1.90016968	1.13995354
O	1.00038644	-2.42156208	0.61395265
O	-1.12219547	-2.58682513	1.32922678
C	-1.12946355	-4.00932214	1.00980386
H	-2.13297623	-4.33597516	1.26388318
H	-0.93976055	-4.13747991	-0.05503315
H	-0.37373386	-4.50753510	1.61672868
In	4.99332312	-0.46954119	-0.19933284
Cl	-4.21211606	-1.08187503	1.93105052

Cl	-6.05328326	-0.16818208	-1.36870705
Cl	-3.79102618	-3.45834729	-1.21137809
Cl	4.49491811	1.25942395	-1.82866038
Cl	6.80785076	-1.83669801	-0.95965833
Cl	5.51697715	0.65505472	1.87136603

TS2-6-*endo* (Fig. S5)

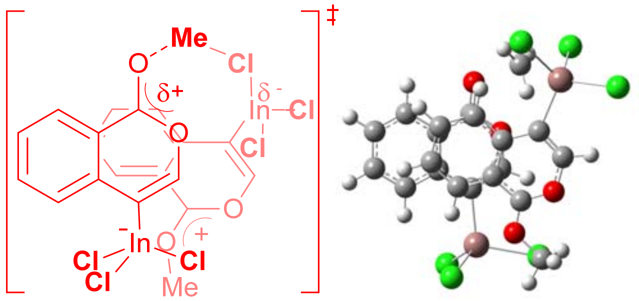

G (348K) = -15317.47634

C	1.84344214	1.34439910	1.55510812
C	1.53060812	0.02106200	1.20045509
C	0.23624202	-0.45432303	1.53208912
C	-0.71314705	0.35488303	2.18978317
C	-0.36819003	1.64624113	2.52161319
C	0.91096607	2.13527716	2.20312417
H	2.83030722	1.74115713	1.33949310
H	-1.70057713	-0.04113500	2.40810118
H	-1.08852408	2.28999318	3.01374123
H	1.17254809	3.15467724	2.46729719
C	2.46717319	-0.86737107	0.53278004
C	2.05094415	-2.11379216	0.26185502
H	2.60527620	-2.91367422	-0.21053502
C	-0.07365701	-1.80144914	1.19545809
O	0.78407906	-2.57406820	0.59263405
O	-1.21553109	-2.30158517	1.49408211
C	-1.51716712	-3.69933628	1.23492210

H	-2.55803820	-3.80055629	1.52927212
H	-1.39705010	-3.90766830	0.17301301
H	-0.86229207	-4.31893633	1.84828214
In	4.54746335	-0.43208403	-0.02603000
C	-3.39588826	2.56419019	0.52857604
C	-2.37017618	2.01217715	-0.25844002
C	-1.24620510	2.81903222	-0.53476004
C	-1.14561209	4.12970432	-0.03995500
C	-2.16772416	4.64516535	0.73336406
C	-3.29459225	3.85584429	1.01473508
H	-4.27730533	1.97255515	0.75732106
H	-0.26344202	4.71679336	-0.27033202
H	-2.10275616	5.65655744	1.11965908
H	-4.09838231	4.26240933	1.61993612
C	-2.41841619	0.64981205	-0.76393006
C	-1.36700510	0.22615602	-1.47620611
H	-1.24492610	-0.74768706	-1.93506615
C	-0.14574601	2.25869317	-1.29251210
O	-0.24983802	1.00091207	-1.73278213
O	0.90838407	2.86249422	-1.53986912
C	2.55182920	1.75747514	-2.00681415
H	3.08193524	2.45176419	-1.37397810
H	2.05115816	0.89563707	-1.59749212
H	2.43274919	1.98161115	-3.05600923
In	-4.06463631	-0.74516405	-0.42029903
Cl	-6.17521147	0.07322601	-1.22422510
Cl	-3.43046326	-2.79398221	-1.54815912
Cl	-4.30871533	-1.17541609	1.95753015
Cl	4.41215534	0.51268704	-2.35934218
Cl	5.59367742	1.33725510	1.18573809
Cl	5.77550146	-2.45455919	-0.27404702

TS2-5-*exo* (Fig. S5)

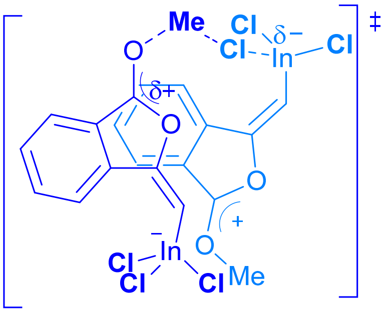

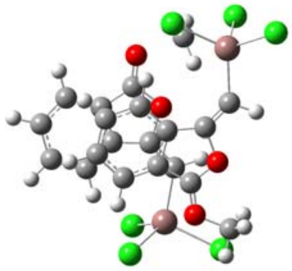

G (348K) = -15317.45291

C	-3.05623982	2.54299908	0.27172029
C	-1.89919968	2.16446149	-0.40393125
C	-0.86007935	3.08023453	-0.56995073
C	-0.91119969	4.37993914	-0.07642738
C	-2.06493592	4.75236850	0.60266272
C	-3.12081059	3.84264266	0.76663737
H	-3.88830930	1.86693056	0.42621928
H	-0.08287953	5.06548347	-0.21830096
H	-2.15622458	5.75519984	1.00563031
H	-4.01412254	4.15787871	1.29569567
C	-1.47887324	0.89367974	-1.00169260
C	-2.03436858	-0.30562035	-1.12784451
H	-1.42328766	-1.05019790	-1.63964079
C	0.21074400	2.38541797	-1.26855640
O	-0.16150078	1.12152465	-1.50109871
O	1.31546034	2.80829240	-1.60855162
C	2.76128913	1.49423401	-2.11567545
H	3.36439943	2.02932426	-1.39758569
H	2.12655807	0.67352808	-1.81871734
H	2.74014813	1.82931802	-3.14192372
In	-4.05781034	-0.88807027	-0.55806342
C	1.60803273	1.23629185	1.74318101
C	1.05999081	-0.01529320	1.48150771

C	-0.25139088	-0.29577770	1.89449462
C	-1.06273580	0.62764775	2.55310765
C	-0.50768460	1.87413344	2.80306388
C	0.80619690	2.16542978	2.40440225
H	2.62260045	1.49031128	1.45924740
H	-2.08317320	0.37962025	2.82606344
H	-1.09719889	2.63641388	3.29973963
H	1.21360200	3.14908207	2.61387941
C	1.59441956	-1.20840117	0.82204329
C	2.72701984	-1.60228234	0.25475397
H	2.69817298	-2.62620573	-0.11933285
C	-0.50141893	-1.64576219	1.48823026
O	0.51599373	-2.17313253	0.87380341
O	-1.56545712	-2.31041078	1.69482567
C	-1.65799419	-3.69666961	1.25046156
H	-2.66765500	-3.99558456	1.51437672
H	-1.51639627	-3.73542130	0.17095837
H	-0.90721667	-4.28364474	1.77898705
In	4.65733584	-0.64914000	-0.08663925
Cl	4.47582237	0.02535448	-2.50965634
Cl	6.45975727	-2.18972558	0.05638076
Cl	5.12894346	1.47512035	0.91979298
Cl	-4.28237804	-3.23470961	-1.08770232
Cl	-4.50346955	-0.60425316	1.81650019
Cl	-5.70845830	0.43368624	-1.71118731

S11

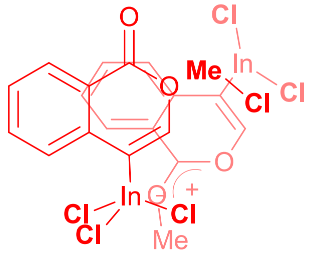

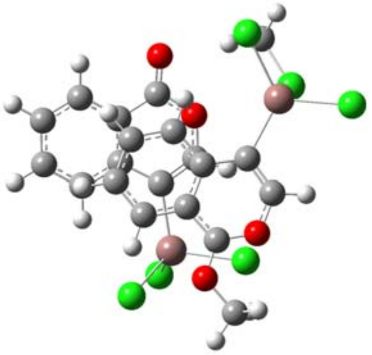

G (348K) = -15317.50559

C	0.99210860	0.89617067	2.08719974
C	0.73141145	-0.42317477	1.68679073
C	-0.49019247	-1.00941199	2.10287812
C	-1.44286050	-0.28483529	2.84774829
C	-1.16161088	1.01474825	3.20685936
C	0.06363676	1.59527715	2.83716707
H	1.92286051	1.37751104	1.80221663
H	-2.38419992	-0.75132484	3.11219251
H	-1.88667541	1.59225146	3.76844321
H	0.27920586	2.61819987	3.12769471
C	1.64390903	-1.20299655	0.87367253
C	1.31590367	-2.47774484	0.60981289
H	1.87659147	-3.20363485	0.03669721
C	-0.71791338	-2.36812058	1.75194892
O	0.15877626	-3.06105922	1.08156207
O	-1.78738762	-2.97243540	2.12269726
C	-2.07874189	-4.31373075	1.65003447
H	-3.06275129	-4.53078942	2.05500268
H	-2.09488424	-4.30475042	0.55979991
H	-1.32896985	-5.00481828	2.03577351
In	3.54699129	-0.53566050	0.07718680
C	-3.01092808	2.68321420	0.55102741

C	-1.77243417	2.33376214	-0.01320234
C	-0.71254349	3.25630918	0.09631372
C	-0.88482424	4.48554574	0.74877845
C	-2.11262172	4.80419115	1.30160921
C	-3.17670145	3.89629047	1.19803071
H	-3.84443452	1.98923143	0.49007521
H	-0.04383663	5.16787849	0.81220771
H	-2.25369122	5.75336036	1.80770023
H	-4.14178457	4.14513899	1.62818980
C	-1.54349558	1.06046104	-0.68059502
C	-0.30992888	0.83105542	-1.15151313
H	0.01428047	-0.06579873	-1.66720412
C	0.60969256	2.92436803	-0.43725589
O	0.74433292	1.70438231	-1.03194310
O	1.60073876	3.62625489	-0.38225685
C	3.65910614	2.01193864	-2.50712645
H	4.71895546	2.07960604	-2.27622821
H	3.04015372	2.52473109	-1.77164313
H	3.44088646	2.33495272	-3.52208379
In	-3.02922316	-0.49119441	-1.01195825
Cl	-4.55474787	-0.09232906	-2.82630467
Cl	-4.36195247	-1.03101973	0.94351996
Cl	-1.70554047	-2.48552761	-1.51467928
Cl	3.21876529	0.24404406	-2.46586381
Cl	4.92768763	-2.39250168	-0.46966937
Cl	4.65461990	1.40069291	0.85790418

S12

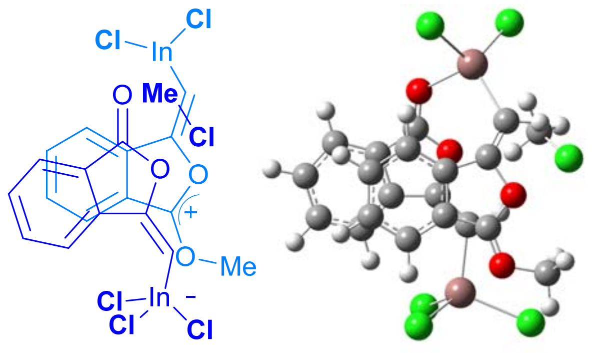

G (348K) = -15317.50007

C	-1.69599864	2.66153012	-1.32205635
C	-0.64329433	1.75071812	-1.28977524
C	0.66796956	2.19266263	-1.48845783
C	0.99108880	3.52785953	-1.71409787
C	-0.06246235	4.43155003	-1.74965992
C	-1.38422820	3.99771580	-1.55652048
H	-2.72701503	2.36221153	-1.17517777
H	2.02048769	3.83856964	-1.85526037
H	0.13233574	5.48309711	-1.93000545
H	-2.18888227	4.72476933	-1.59044367
C	-0.59699916	0.30988282	-1.03381487
C	-1.48153840	-0.62269247	-0.70217794
H	-1.06391877	-1.62081422	-0.55873254
C	1.52479616	1.03672065	-1.31914208
O	0.78483049	-0.04545823	-1.12619785
O	2.76438729	0.98083914	-1.27858895
C	1.05448923	-3.16280397	-2.27106903
H	2.05522413	-2.73644152	-2.22447808
H	0.32210231	-2.40645280	-2.54746400
H	1.02442029	-4.00069481	-2.96433239
In	-3.63718123	-0.40929765	-0.44784761
C	1.76627094	1.96076031	1.72715571

C	1.06889171	0.77698974	1.94275713
C	-0.27660744	0.82167235	2.33614629
C	-0.98887843	2.01178421	2.47531291
C	-0.29476401	3.18626911	2.21825470
C	1.06330893	3.15616737	1.86697062
H	2.82090298	1.97284587	1.47174862
H	-2.03771253	2.00962606	2.74720037
H	-0.80461036	4.13936519	2.29963437
H	1.58441782	4.09219734	1.69568993
C	1.45730564	-0.62580562	1.81868856
C	2.46249765	-1.28700133	1.26368769
H	2.38378400	-2.37216720	1.27944592
C	-0.66057006	-0.54632531	2.52673997
O	0.31833893	-1.37271561	2.29917258
O	-1.80996560	-0.96434333	2.87861760
C	-2.04678025	-2.39719452	3.02664762
H	-3.10860427	-2.47340606	3.23806983
H	-1.80303841	-2.90053100	2.09164331
H	-1.43881376	-2.76607434	3.85245593
In	3.95859822	-0.42599367	-0.05564528
Cl	0.63112037	-3.78448355	-0.63620012
Cl	4.82331781	-1.93790198	-1.66870033
Cl	5.49998067	1.17644378	0.79202933
Cl	-4.69170849	0.27040753	-2.50307899
Cl	-4.22074271	1.26894845	1.20909680
Cl	-4.44951031	-2.53965885	0.34353527

S13

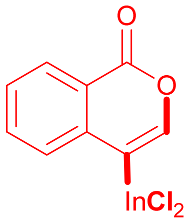

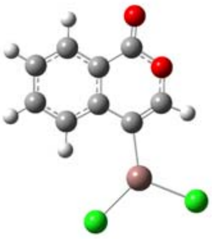

G (348K) = -7158.707214

C	1.37156245	1.80299643	-0.00002879
C	1.52520264	0.40797039	0.00000229
C	2.83161518	-0.11945635	0.00000290
C	3.94834222	0.72617791	-0.00002810
C	3.77445663	2.09987438	-0.00005727
C	2.48037336	2.63487317	-0.00005677
H	0.37735441	2.24153676	-0.00004588
H	4.93853360	0.28376948	-0.00002403
H	4.63630265	2.75829217	-0.00007998
H	2.34044736	3.71106184	-0.00008228
C	0.40450995	-0.52413493	0.00005001
C	0.67647377	-1.84391224	0.00005952
H	-0.06084711	-2.63934374	0.00011535
C	3.05226413	-1.57242119	0.00007114
O	1.92624683	-2.36527178	0.00005061
O	4.12341362	-2.12869807	-0.00005147
In	-1.65929877	0.00295279	-0.00000907
Cl	-2.58587059	2.16933256	0.00001211
Cl	-3.27955510	-1.71168844	0.00001604

S14

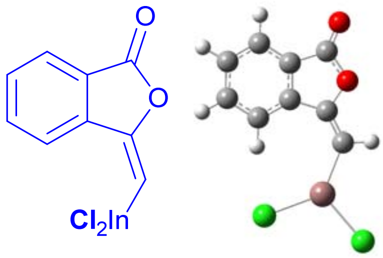

G (348K) = -7158.700453

C	-1.55465471	1.52471474	0.00009974
C	-1.92767239	0.18430619	0.00002535
C	-3.27496407	-0.16783838	-0.00001359
C	-4.29407862	0.77448030	0.00001655
C	-3.92184254	2.11642581	0.00008689
C	-2.56944900	2.48075432	0.00012791
H	-0.51871140	1.84320951	0.00013082
H	-5.33467400	0.46877979	-0.00001581
H	-4.68349893	2.88877804	0.00011020
H	-2.30235682	3.53242839	0.00018211
C	-1.15265549	-1.06917238	-0.00002921
C	0.15170077	-1.36478830	-0.00003036
H	0.39007039	-2.42645371	-0.00009075
C	-3.35907113	-1.63930379	-0.00008319
O	-2.06547785	-2.11421973	-0.00009216
O	-4.30454225	-2.37963190	-0.00014294
In	1.86271557	-0.11790321	0.00004961
Cl	2.10416085	2.23321272	0.00017444
Cl	3.98715632	-1.15180936	-0.00029604

MeCl

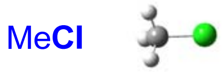

G (348K) = -500.037608

C	0.00000000	-0.00000000	-1.13424456
H	0.00000000	1.03304922	-1.47802325
H	-0.89464687	-0.51652461	-1.47802325
H	0.89464687	-0.51652461	-1.47802325
Cl	-0.00000000	0.00000000	0.66114924

S15

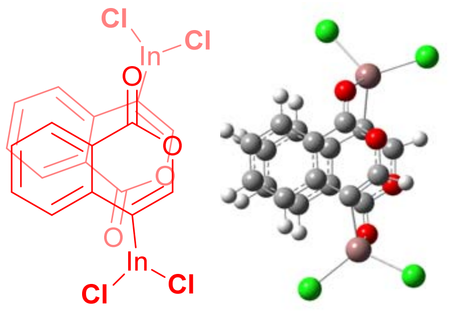

G (348K) = -14317.45892

C	-1.44876816	1.75249217	-1.66277731
C	-0.90626876	0.45665643	-1.61999903
C	0.47099315	0.30780615	-1.90007492
C	1.28137120	1.41670434	-2.20280584
C	0.72020507	2.67674186	-2.23630542
C	-0.64767838	2.83949196	-1.96352316
H	-2.50477980	1.90233331	-1.45638578
H	2.33700407	1.26966677	-2.40753695
H	1.33501254	3.53948412	-2.46710262
H	-1.08400450	3.83276681	-1.98489600
C	-1.68264421	-0.71491054	-1.24821970
C	-1.06589931	-1.90531739	-1.32475713
H	-1.49619680	-2.87521620	-1.10998947
C	1.06955923	-0.99658064	-1.75859156
O	0.27032488	-2.05257212	-1.62846412
O	2.29770602	-1.21160136	-1.69816312

In	-3.50112182	-0.57871515	-0.06125065
C	1.90258878	2.45204313	1.26114399
C	1.06734914	1.32791956	1.37497550
C	-0.30467444	1.55051584	1.63800422
C	-0.82664168	2.84863454	1.76833880
C	0.02281682	3.93218049	1.67171679
C	1.38875607	3.72728283	1.41789771
H	2.95897527	2.31660843	1.05152969
H	-1.88854805	2.97656813	1.94764764
H	-0.36353824	4.93904505	1.78556676
H	2.05254113	4.58194560	1.33583653
C	1.54757433	-0.03675116	1.21801372
C	0.67185124	-1.02314929	1.47249300
H	0.86351913	-2.08911279	1.45862553
C	-1.19220598	0.41424562	1.67768285
O	-0.66006492	-0.80452708	1.74329054
O	-2.43529930	0.48232403	1.60755345
In	3.37100151	-0.65989132	0.19684723
Cl	4.91173711	0.97098617	-0.59397616
Cl	4.31728569	-2.73974456	0.81879786
Cl	-5.18088045	1.04594811	-0.48232777
Cl	-4.28491455	-2.63997770	0.81688624

S16

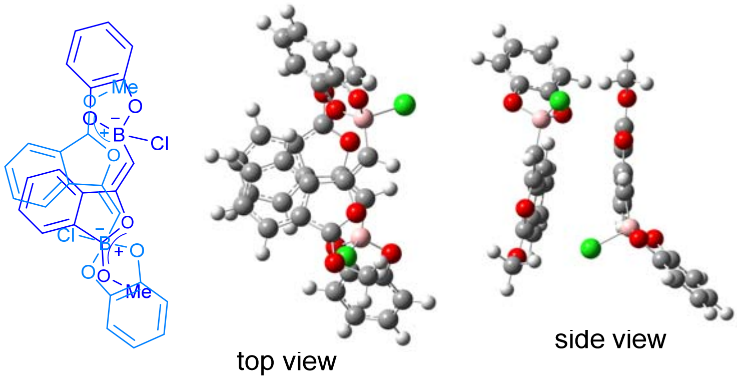

G (398K) = -2804.990925

C	-1.20695352	0.64900599	2.25426888
C	-0.50521835	1.30519407	1.24173143
C	0.69312414	1.96781294	1.54015204
C	1.23413358	2.01850129	2.82662078
C	0.52905776	1.37024701	3.82561763
C	-0.67200093	0.69597426	3.53449797
H	-2.13681524	0.13486525	2.04641594
H	2.16130512	2.54301077	3.02852024
H	0.90266611	1.37897878	4.84369876
H	-1.19902940	0.19498012	4.34007193
C	-0.76122870	1.46610663	-0.18074508
C	-1.72982177	1.11998057	-1.02315904
H	-1.56637308	1.39320991	-2.06479323
C	1.13731971	2.52710622	0.30005244
O	0.34627122	2.23457742	-0.68340478
O	2.14624393	3.29664343	0.15108122
C	2.50396532	3.75007309	-1.18505530
H	3.42643030	4.30595627	-1.04448385
H	2.65168804	2.87973582	-1.82542976
H	1.70841854	4.39214695	-1.56391883
C	-7.60740858	1.41809082	-0.94105761
C	-7.41787047	1.72171566	0.40516152
C	-6.14760493	1.62952269	0.99746318
C	-5.09993621	1.22559747	0.19319900
C	-5.28977184	0.92124798	-1.16056211
C	-6.53529008	1.00989519	-1.75140076
H	-8.59955316	1.49622398	-1.37457760
H	-8.26372696	2.03359564	1.00987320
H	-5.98879413	1.86486858	2.04450577
H	-6.67221940	0.77288149	-2.80103520
O	-4.10861026	0.57798901	-1.73387702
O	-3.79144121	1.08119950	0.52617051

B	-3.15381723	0.48369920	-0.64272066
Cl	-2.88435591	-1.44855939	-0.22624289
C	1.84803275	-1.39448271	1.50801834
C	1.02641953	-2.02599765	0.57372429
C	0.07719619	-2.95957069	1.01543312
C	-0.11052308	-3.28394534	2.36019086
C	0.70976657	-2.64960088	3.27632412
C	1.67706018	-1.72405411	2.84580206
H	2.58967623	-0.67110841	1.20144176
H	-0.86403943	-4.00155525	2.66357838
H	0.61046701	-2.86717324	4.33395480
H	2.31063202	-1.24461469	3.58472564
C	0.94544769	-1.96047042	-0.88142403
C	1.55706099	-1.33522464	-1.88350862
H	1.19834253	-1.62512157	-2.86988049
C	-0.55826059	-3.44852798	-0.16813412
O	-0.07471402	-2.91265077	-1.24256377
O	-1.45959625	-4.35495600	-0.22299902
C	-2.05877278	-4.66632090	-1.51040889
H	-2.79120801	-5.43765830	-1.29171332
H	-2.53622752	-3.76487496	-1.89569786
H	-1.28823207	-5.03269034	-2.18871578
C	6.82502981	0.24947371	0.24036165
C	6.09024487	1.16650324	0.98620697
C	4.71850618	1.35764854	0.74662151
C	4.13657838	0.60514790	-0.25502160
C	4.87370151	-0.32187734	-1.00244605
C	6.22285145	-0.51458676	-0.77260212
H	7.88404980	0.12114628	0.44079861
H	6.57980309	1.74629757	1.76222487
H	4.13895791	2.07417315	1.31841389
H	6.78870147	-1.23174498	-1.35711674
O	4.07836125	-0.95191139	-1.89529074

O	2.83173659	0.58764812	-0.64371161
B	2.77612073	-0.28358326	-1.83226521
Cl	2.55897611	0.84470420	-3.40579858

TS2-anti-5-*exo* (Fig. S7)

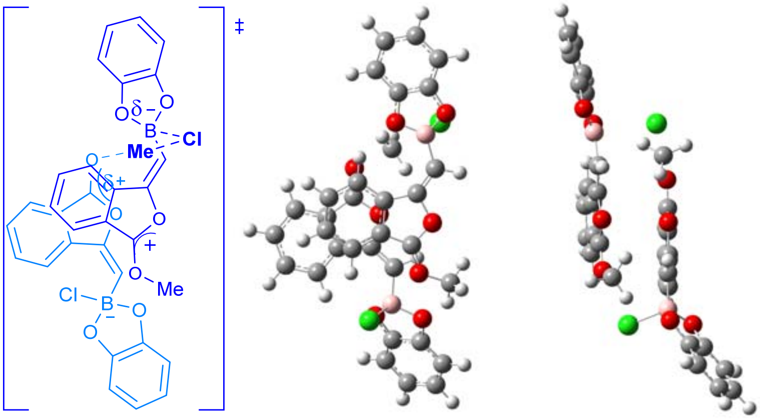

G (398K) = -2804.956804

C	-3.02405961	2.89899316	0.02383301
C	-1.93729143	2.18985937	-0.48579525
C	-0.75808708	2.85867720	-0.82489369
C	-0.59861387	4.23314298	-0.66559736
C	-1.67709795	4.93558433	-0.14623711
C	-2.86971204	4.27162978	0.18919338
H	-3.95032920	2.39760567	0.27713556
H	0.32863402	4.72506511	-0.93805633
H	-1.60544733	6.00835141	-0.00324399
H	-3.69847083	4.84879081	0.58631223
C	-1.73374886	0.77002501	-0.74476479
C	-2.45077674	-0.33445853	-0.56803875
H	-1.95745299	-1.25527832	-0.88168005
C	0.16991349	1.85184899	-1.30581891
O	-0.40726440	0.65569479	-1.26114082
O	1.33622802	2.00145654	-1.69936536

C	2.37116965	0.50800338	-2.12153631
H	3.15885867	0.82394875	-1.45618063
H	1.65502330	-0.22830328	-1.79892991
H	2.43159440	0.75965410	-3.16821498
C	-7.90986666	-2.46630461	-1.11556932
C	-8.22200308	-1.15552156	-1.46705838
C	-7.27302978	-0.12677644	-1.34829133
C	-6.02228649	-0.46614962	-0.87040477
C	-5.70791827	-1.78527158	-0.51835629
C	-6.63490134	-2.80269090	-0.63122719
H	-8.66170556	-3.24300531	-1.21444257
H	-9.21509494	-0.92048400	-1.83704367
H	-7.50668033	0.89731806	-1.61910429
H	-6.38248311	-3.82105931	-0.35511493
O	-4.41686148	-1.86507364	-0.10425614
O	-4.94639569	0.34085806	-0.68731473
B	-3.94868326	-0.48097569	-0.00991827
Cl	-3.96592253	0.02186608	1.91225538
C	2.24602303	1.11745055	1.52700670
C	1.55789691	-0.09043062	1.43608508
C	0.22834040	-0.16063253	1.87918544
C	-0.47003236	0.92957299	2.39753590
C	0.22417609	2.12695401	2.47846121
C	1.56007528	2.20934854	2.05388324
H	3.26983162	1.20601933	1.18830052
H	-1.51492429	0.83665042	2.67658132
H	-0.27330444	3.01268786	2.85702288
H	2.07428908	3.16228232	2.12421785
C	1.91863819	-1.40576418	0.91127590
C	2.98625089	-1.99823091	0.37943799
H	2.85701765	-3.04182888	0.10292639
C	-0.20021457	-1.49865711	1.61026469
O	0.73976778	-2.21171788	1.05409315

O	-1.34982199	-1.97270349	1.85754449
C	-1.76191407	-3.29624090	1.40883043
H	-1.60199010	-3.98588099	2.23633020
H	-2.81856359	-3.18042694	1.17224155
H	-1.18625926	-3.58918614	0.53207771
C	8.78902942	-0.53239535	-0.27314096
C	8.31773093	0.78072259	-0.19961796
C	6.95391415	1.05864829	-0.04964169
C	6.11055591	-0.03108188	0.02016758
C	6.57769167	-1.33933355	-0.05378176
C	7.91996151	-1.62715448	-0.20059481
H	9.85235977	-0.70991977	-0.39418776
H	9.02102051	1.60415445	-0.26408588
H	6.57705571	2.07370195	0.00291048
H	8.27480068	-2.64916925	-0.26332611
O	5.52101319	-2.20693083	0.04849725
O	4.74338688	-0.04994455	0.17435561
B	4.39768208	-1.39770795	0.10308876
Cl	3.77697711	-1.41078258	-2.58498299

S17

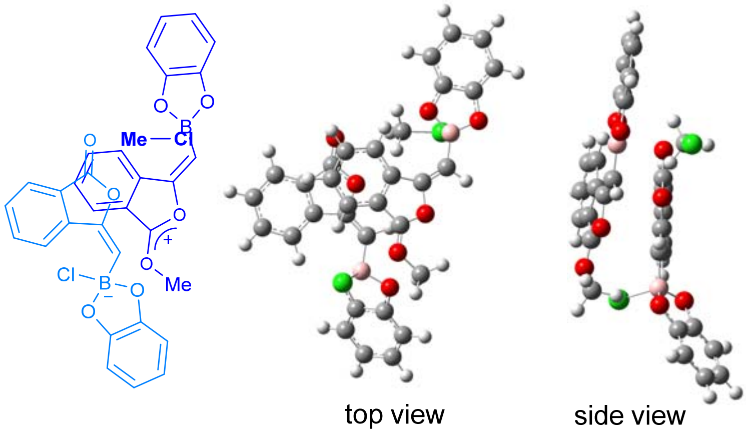

G (398K) = -2805.009453

C	-3.36133972	2.87930960	-0.41154726

C	-2.05380963	2.43806171	-0.61034845
C	-1.03058877	3.35560936	-0.84418890
C	-1.24985381	4.72820173	-0.87838982
C	-2.55116902	5.17070394	-0.66749878
C	-3.58791587	4.25271575	-0.44025953
H	-4.17010241	2.17865613	-0.24489890
H	-0.43389189	5.41805646	-1.06668918
H	-2.77179332	6.23292370	-0.68574140
H	-4.59657360	4.62298495	-0.28649201
C	-1.45795420	1.10016732	-0.62693640
C	-1.93442598	-0.12676389	-0.42438111
H	-1.19475751	-0.92427997	-0.52136470
C	0.21578427	2.60083186	-1.03072032
O	-0.08563701	1.27841741	-0.89193725
O	1.34435703	2.97608619	-1.25508653
C	2.72825601	0.31427765	-2.33610298
H	3.54587087	0.94767346	-2.00034868
H	1.96952504	0.22501451	-1.56304075
H	2.28787309	0.70165095	-3.25206625
C	-6.58897553	-3.59451006	-1.51127226
C	-7.11619303	-2.44724814	-2.09828277
C	-6.45712949	-1.21087247	-1.99609480
C	-5.26902072	-1.17687929	-1.29181808
C	-4.73847271	-2.33224331	-0.70198642
C	-5.37933196	-3.55176208	-0.79756121
H	-7.11813373	-4.53791282	-1.60320350
H	-8.05332889	-2.50488433	-2.64324415
H	-6.86005589	-0.31193265	-2.45033212
H	-4.95935683	-4.44115752	-0.33917379
O	-3.55925293	-2.04566847	-0.09383938
O	-4.45156858	-0.11810088	-1.07734149
B	-3.43776712	-0.58173891	-0.13315463
Cl	-3.98219309	0.09501128	1.67281931

C	2.11753527	1.35770195	1.59617961
C	1.37479472	0.19457582	1.76051125
C	0.09291170	0.26197777	2.32529289
C	-0.51553600	1.45494624	2.70451654
C	0.23007828	2.61263889	2.52728588
C	1.52659267	2.55544409	1.99450382
H	3.10090912	1.34145534	1.14674977
H	-1.53228313	1.46765487	3.08075791
H	-0.19835641	3.57355869	2.78857376
H	2.07822902	3.47888627	1.85622828
C	1.62855757	-1.20578981	1.41829969
C	2.62381181	-1.93649551	0.91685492
H	2.41730452	-3.00005400	0.82093108
C	-0.40309686	-1.08016250	2.33009232
O	0.44453493	-1.91634642	1.78483632
O	-1.50001229	-1.47044711	2.81995062
C	-1.98726836	-2.83237649	2.62013446
H	-2.75789659	-2.95180528	3.37548451
H	-2.42064492	-2.87339714	1.61870240
H	-1.16957177	-3.53808749	2.75998072
C	8.28257848	-1.05190935	-0.91458935
C	7.94927465	0.30272619	-0.82250116
C	6.68153308	0.71628442	-0.39950198
C	5.78891356	-0.28721807	-0.08194497
C	6.11737541	-1.63453167	-0.17551539
C	7.36491316	-2.05659722	-0.59007186
H	9.27524139	-1.33282468	-1.24965154
H	8.68780453	1.05167951	-1.08716083
H	6.41034307	1.76322141	-0.32810696
H	7.61182800	-3.10909300	-0.66420655
O	5.03087471	-2.39021486	0.19348391
O	4.48675883	-0.17307643	0.35147868
B	4.03627502	-1.47474470	0.47861354

Cl	3.39006762	-1.32710840	-2.67836557

S18 (Cl anion)

G (398K) = -460.339692

Cl	0.00000000	0.00000000	0.00000000

S18 (Cation moiety)

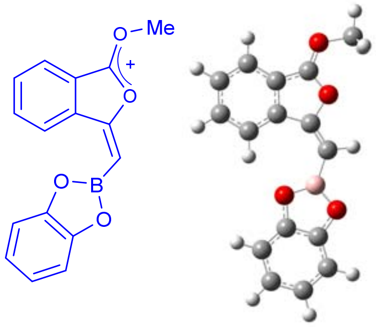

G (398K) = -942.106167

C	-0.94627900	1.85558000	-0.00004000
C	-1.64820800	0.65500100	-0.00005400
C	-3.05357000	0.66839700	-0.00007600
C	-3.80440200	1.84372000	-0.00007700
C	-3.09528500	3.03585000	-0.00006600
C	-1.69181500	3.03370300	-0.00004800
H	0.13553900	1.87537200	-0.00001300
H	-4.88791800	1.82190100	-0.00008500
H	-3.62788000	3.97961400	-0.00006500
H	-1.16701100	3.98292600	-0.00003300
C	-1.22133500	-0.74356300	-0.00001800
C	-0.08160700	-1.43304200	0.00000800
H	-0.19345200	-2.51503700	0.00003400
C	-3.45025800	-0.70417900	-0.00001800
O	-2.42797300	-1.51378600	0.00000700
O	-4.64158000	-1.15209900	0.00003600
C	-4.87747400	-2.59323900	0.00029300

H	-5.95851300	-2.68919400	0.00073200
H	-4.44138200	-3.02424900	-0.90015900
H	-4.44065300	-3.02406900	0.90047900
C	5.82336300	-0.29887600	-0.00010400
C	5.42381400	1.04174800	0.00011100
C	4.07316000	1.40354200	0.00021000
C	3.16657000	0.36241400	0.00008400
C	3.56225700	-0.96910900	-0.00012400
C	4.89209700	-1.34148800	-0.00022800
H	6.88128800	-0.53706900	-0.00017400
H	6.17821000	1.82073600	0.00020600
H	3.75457300	2.43923100	0.00037900
H	5.19099100	-2.38296700	-0.00039100
O	2.44224000	-1.77120600	-0.00018700
O	1.78604400	0.41852900	0.00015000
B	1.37604200	-0.89949000	-0.00001700

S19

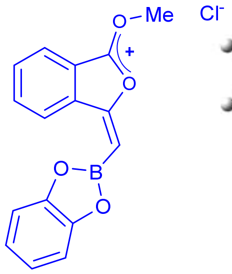

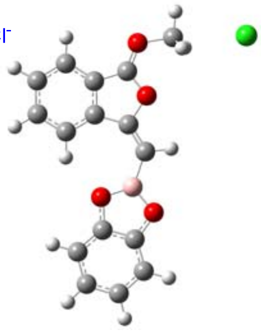

G (398K) = -1402.473992

C	0.32244300	2.47021300	-0.01169600
C	-0.70682000	1.53418700	0.00232400
C	-2.04111600	1.96636700	0.00665100
C	-2.40613400	3.30996400	-0.00231500
C	-1.37329100	4.23760100	-0.01611000
C	-0.03518500	3.81739100	-0.02066300

H	1.36006300	2.16408300	-0.01579400
H	-3.44744800	3.61070300	0.00106500
H	-1.60053200	5.29744400	-0.02359300
H	0.74943700	4.56649500	-0.03173300
C	-0.72162700	0.06979200	0.01408500
C	0.16234700	-0.92754500	0.01792300
H	-0.26940000	-1.92621400	0.02847900
C	-2.83953000	0.77152100	0.02009300
O	-2.09551500	-0.30328800	0.02442000
O	-4.09933300	0.70036300	0.02704400
C	-4.75469200	-0.64178000	0.03499800
H	-5.81476100	-0.41829400	0.02988700
H	-4.44888500	-1.18252500	-0.85574200
H	-4.45317800	-1.16567300	0.93750100
C	6.13894000	-1.59757200	0.00058000
C	6.15549200	-0.19932200	-0.01999600
C	4.97218900	0.54641400	-0.02546100
C	3.79697400	-0.17776800	-0.00945100
C	3.77929100	-1.56691000	0.01084700
C	4.93939500	-2.31617300	0.01654100
H	7.07820600	-2.13981800	0.00421700
H	7.10698200	0.32108200	-0.03207400
H	4.97462700	1.62999400	-0.04137000
H	4.91497400	-3.39931200	0.03242300
O	2.47425100	-2.00005500	0.02299600
O	2.49763900	0.28553300	-0.01013300
B	1.70930900	-0.85164400	0.00991400
Cl	-5.72029600	-3.54932000	-0.04162000

TS2-anti-5-*exo* (Fig. S7)

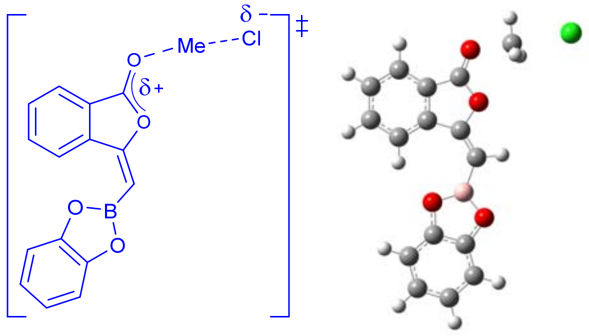

G (398K) = -1402.467076

C	0.34254000	2.43252900	-0.00002800
C	-0.68281700	1.49245300	-0.00001500
C	-2.01532100	1.91711700	-0.00003600
C	-2.38428600	3.25629000	-0.00007000
C	-1.35675000	4.19116400	-0.00008600
C	-0.01843500	3.77832200	-0.00006500
H	1.38193000	2.13167500	-0.00000500
H	-3.42807600	3.54997400	-0.00008500
H	-1.59025600	5.25022000	-0.00011400
H	0.76399500	4.53034900	-0.00007600
C	-0.69617800	0.02565700	0.00001200
C	0.20444100	-0.95852400	0.00002200
H	-0.21378400	-1.96307200	0.00002300
C	-2.83278900	0.71944600	-0.00001100
O	-2.05651500	-0.35668600	0.00001300
O	-4.06793800	0.63468900	-0.00000800
C	-4.81224800	-0.94849800	0.00001900
H	-4.41006400	-1.33074300	-0.92522300
H	-4.41015400	-1.33066000	0.92533500
H	-5.82507400	-0.58121400	-0.00004900
C	6.18313900	-1.57538500	-0.00009800
C	6.18807100	-0.17844900	0.00011800
C	4.99891200	0.55653100	0.00023300

C	3.82961400	-0.17630500	0.00012100
C	3.82290400	-1.56562900	-0.00009300
C	4.98995400	-2.30344700	-0.00020900
H	7.12702600	-2.11039700	-0.00018200
H	7.13525900	0.35068200	0.00019900
H	4.99115700	1.64056500	0.00040100
H	4.97455800	-3.38719900	-0.00037500
O	2.52368400	-2.01010000	-0.00015700
O	2.52888900	0.27619200	0.00019200
B	1.74704300	-0.86715400	0.00001800
Cl	-5.95418700	-3.22040000	0.00005100

S20

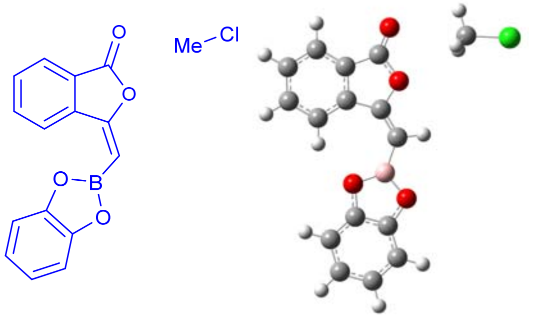

G (398K) = -1402.534084

C	0.43249800	2.59103700	-0.02802300
C	-0.67141500	1.74239100	0.00906300
C	-1.96069100	2.27279800	0.01743600
C	-2.21014400	3.63751300	-0.00960600
C	-1.10731600	4.48629600	-0.04647400
C	0.19179300	3.96405500	-0.05537900
H	1.44207000	2.20216600	-0.03546300
H	-3.22582300	4.01763300	-0.00208300
H	-1.25259800	5.56101000	-0.06858300
H	1.03557100	4.64587400	-0.08442700
C	-0.80875500	0.27619200	0.04452500

C	0.04081400	-0.76067000	0.05500800
H	-0.44068300	-1.73610700	0.08517600
C	-2.91283200	1.15129700	0.05796800
O	-2.16940600	-0.00420600	0.07246400
O	-4.11524700	1.12904100	0.07733900
C	-4.90092500	-1.95281800	-0.01002300
H	-5.77807100	-1.31557200	0.07200300
H	-4.35191300	-1.73061400	-0.92192400
H	-4.25605600	-1.82865000	0.85653600
C	5.94903000	-1.87199800	-0.00789600
C	6.07162400	-0.48030400	-0.04662900
C	4.94648300	0.35228800	-0.05101800
C	3.71883100	-0.27796800	-0.01509900
C	3.59543900	-1.66222200	0.02338500
C	4.69669400	-2.49559000	0.02823100
H	6.84403600	-2.48496100	-0.00570300
H	7.05958100	-0.03320400	-0.07402600
H	5.03045800	1.43248600	-0.08095900
H	4.59068800	-3.57373200	0.05839400
O	2.26502200	-1.99455200	0.05288000
O	2.46324800	0.28343400	-0.01026400
B	1.57625800	-0.78685100	0.03184300
Cl	-5.44436700	-3.66856400	-0.07590100

TS1-syn-5-*exo* (Fig. S9)

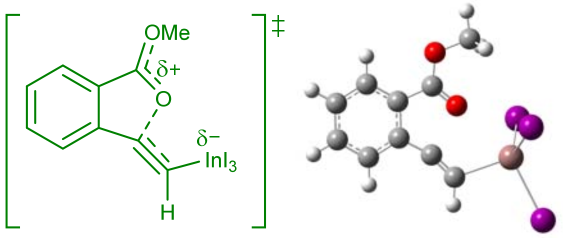

G (348K) = -27038.33866

C	3.91732991	2.87370376	-0.00017797

C	3.25832449	1.64141889	-0.00009858
C	3.99847419	0.45343001	-0.00002582
C	5.38792463	0.46946259	-0.00003018
C	6.04307443	1.69779235	-0.00010952
C	5.30992793	2.88644751	-0.00018273
H	3.35379176	3.79980733	-0.00023469
H	5.94097809	-0.46314960	0.00002725
H	7.12683414	1.73183615	-0.00011460
H	5.82925084	3.83874140	-0.00024479
C	1.82167850	1.58456426	-0.00009170
C	0.60554333	1.89142122	-0.00010925
H	0.10138081	2.84930605	-0.00016711
C	3.12808056	-0.73082729	0.00004936
O	1.91372878	-0.53581715	0.00004019
O	3.68567774	-1.91172446	0.00012095
C	2.77926352	-3.04112584	0.00019314
H	3.42105469	-3.91835974	0.00024338
H	2.15377104	-3.00702759	0.89339172
H	2.15376305	-3.00713557	-0.89300403
In	-0.92855635	0.13883926	-0.00000707
I	-3.24003626	1.64249796	-0.00010560
I	-0.70898061	-1.26860186	-2.33979596
I	-0.70899340	-1.26832127	2.33995174

S21

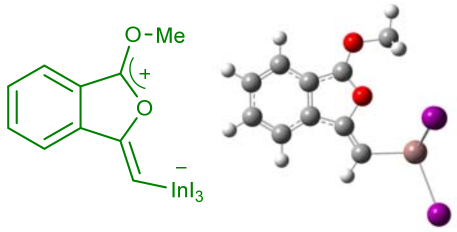

G (348K) = -27038.3617

C	-3.95568157	-2.80658188	0.03846075

C	-3.41295570	-1.52373669	0.02497952
C	-4.25943772	-0.40404643	0.01155898
C	-5.65293866	-0.50920188	0.01092456
C	-6.18293497	-1.78888845	0.02445693
C	-5.34135894	-2.91754818	0.03801567
H	-3.32292622	-3.68668631	0.04866827
H	-6.28512016	0.37130055	0.00026551
H	-7.25815689	-1.92756464	0.02457246
H	-5.79097396	-3.90503529	0.04832533
C	-2.03355105	-1.05527778	0.02043996
C	-0.81218419	-1.56968442	0.02573896
H	-0.74920560	-2.65569924	0.03808733
C	-3.38219997	0.72910757	-0.00034754
O	-2.13670875	0.38161182	0.00434594
O	-3.71283609	1.96393878	-0.01400954
C	-2.64258743	2.96072165	-0.02416143
H	-3.16050920	3.91482006	-0.03806536
H	-2.03731165	2.84213487	0.87528968
H	-2.03220952	2.81872457	-0.91675116
In	0.96604532	-0.24646972	0.00369994
I	0.80416658	1.39186871	2.23702487
I	3.24068307	-1.78363636	0.02454193
I	0.78819812	1.31349285	-2.28429846

S22

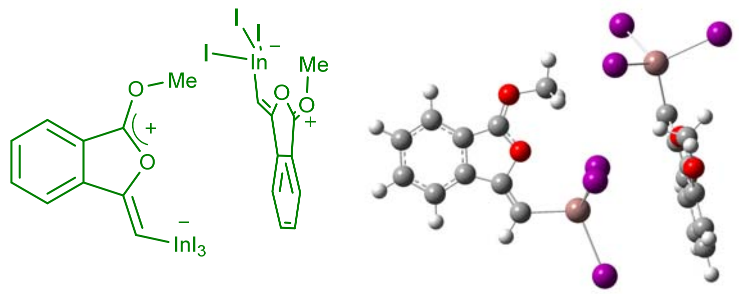

G (348K) = -54076.73322

C	-5.85118799	4.05576673	0.87501079
C	-4.55017859	3.61083471	0.65498599
C	-3.48329958	4.52093054	0.68127150
C	-3.65836268	5.88542369	0.92263180
C	-4.95625809	6.31932072	1.14106217
C	-6.03284654	5.41327820	1.11620596
H	-6.69226395	3.37187250	0.85904464
H	-2.81639576	6.56806001	0.93750170
H	-5.14875355	7.36875308	1.33337560
H	-7.03603749	5.78818224	1.29074644
C	-4.01216364	2.28472678	0.37822364
C	-4.47068541	1.05057375	0.22749366
H	-5.54670236	0.92663395	0.32870652
C	-2.31078081	3.73669213	0.41878356
O	-2.59235033	2.48323450	0.24936172
O	-1.10939671	4.15437631	0.34461648
C	-0.04750799	3.18576798	0.05650950
H	0.86992161	3.76501212	0.09146658
H	-0.05575485	2.40879786	0.82171575
H	-0.21595340	2.76370309	-0.93473707
In	-3.08893429	-0.62026761	-0.19980868
I	-1.38148818	-0.75869240	1.98287874
I	-4.51158287	-2.95291547	-0.51260807
I	-1.71537294	0.06532555	-2.50895135
C	-0.29841842	-3.52533206	-1.75180045
C	0.46544457	-3.15969270	-0.64711708
C	0.39684478	-3.91112580	0.53490415
C	-0.42763569	-5.03026395	0.67310036
C	-1.18854778	-5.38304181	-0.42849583
C	-1.12094116	-4.63796853	-1.62034217
H	-0.26760773	-2.95579147	-2.67290382
H	-0.47399705	-5.58589021	1.60255305

H	-1.85651688	-6.23499321	-0.37288838
H	-1.74195160	-4.93492584	-2.45872340
C	1.39267118	-2.05849665	-0.43322484
C	1.83916038	-1.01702321	-1.11895743
H	1.44547264	-0.91940342	-2.12830493
C	1.28413485	-3.25545117	1.44505146
O	1.86830936	-2.22896634	0.91303926
O	1.53151538	-3.59118251	2.65487746
C	2.44198000	-2.75467440	3.42707818
H	2.45402933	-3.20403109	4.41556529
H	3.42919474	-2.77911388	2.96504016
H	2.05635303	-1.73466415	3.45247564
In	3.30421653	0.41770237	-0.32550525
I	5.74637544	-0.87559814	-0.21990999
I	3.32841452	2.58524691	-2.04195679
I	2.70337071	1.31007374	2.23640294

TS2-syn-5-*exo* (Fig. S9)

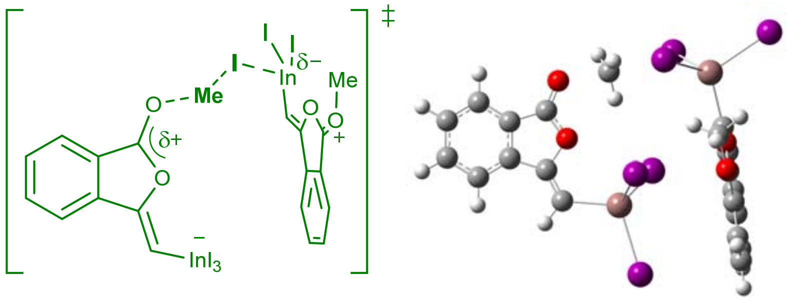

G (348K) = -54076.69618

C	-5.69158416	4.00505223	0.97861226
C	-4.37900853	3.64957118	0.67935253
C	-3.36247352	4.60292474	0.72780636
C	-3.59751092	5.93243908	1.06736552
C	-4.90752176	6.28642569	1.36682971
C	-5.93700254	5.33170717	1.32099008

H	-6.49559967	3.27772027	0.94741567
H	-2.78913162	6.65476137	1.09680112
H	-5.14171652	7.30988502	1.63892003
H	-6.95041386	5.63848731	1.55932787
C	-3.77815937	2.37113027	0.29943139
C	-4.23515845	1.13706928	0.13301050
H	-5.30359484	1.00326816	0.28792236
C	-2.12828694	3.91933302	0.35002919
O	-2.39326430	2.63459738	0.11650710
O	-0.99597798	4.38719486	0.22865566
C	0.49397843	3.39282100	-0.68712876
H	0.62671780	4.29725846	-1.25865470
H	1.00392248	3.27380746	0.25870983
H	-0.14070219	2.59821734	-1.06022274
In	-2.90567231	-0.54263303	-0.28498546
I	-1.37866116	-0.83794207	2.02757291
I	-4.33649912	-2.86167526	-0.73904191
I	-1.30154609	0.00688189	-2.48529443
C	-0.20059743	-3.84843635	-1.60443865
C	0.48831824	-3.43911183	-0.46734553
C	0.29609284	-4.10308914	0.75211668
C	-0.57555994	-5.18515876	0.89355986
C	-1.25319603	-5.59088681	-0.24398901
C	-1.06719129	-4.92734166	-1.47063665
H	-0.08562791	-3.33433057	-2.55154838
H	-0.72207201	-5.67107722	1.85118510
H	-1.95275623	-6.41710262	-0.18911978
H	-1.63255756	-5.25827754	-2.33517658
C	1.41757804	-2.34195184	-0.24314746
C	1.91476275	-1.35073832	-0.96612364
H	1.60794142	-1.34136130	-2.00937356
C	1.10878534	-3.39648478	1.69289989
O	1.76891419	-2.42162377	1.14586125

O	1.23157010	-3.64780984	2.94076032
C	2.04500588	-2.75131185	3.75067432
H	1.92548864	-3.11604587	4.76648641
H	3.08303110	-2.81655868	3.42430060
H	1.66835836	-1.73307981	3.64266076
In	3.17145525	0.26321350	-0.20315064
I	5.83369079	-0.35064307	-0.43839684
I	2.60074399	2.39412431	-1.99999475
I	2.57241857	1.26327953	2.28450499

S23

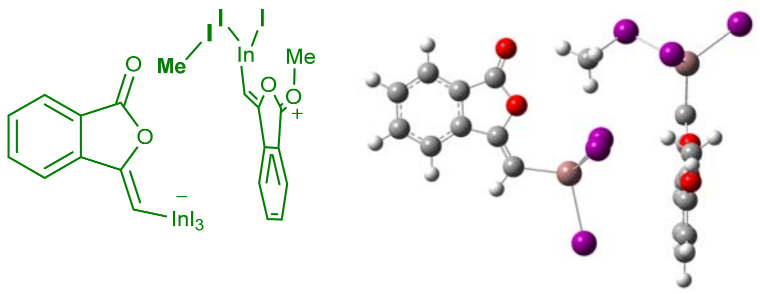

G (348K) = -54076.72404

C	6.69036034	-3.06488530	0.57704726
C	5.30508876	-3.08193509	0.43862053
C	4.60690742	-4.28488719	0.45965093
C	5.24289079	-5.51125293	0.61893529
C	6.62715403	-5.49785194	0.75901821
C	7.33763211	-4.28739687	0.73763239
H	7.24911526	-2.13502537	0.56084477
H	4.67621666	-6.43615390	0.63224018
H	7.16667971	-6.43046954	0.88615193
H	8.41724128	-4.30645978	0.84855439
C	4.32829468	-2.00298614	0.25361415
C	4.45565067	-0.68388883	0.15274206
H	5.47252314	-0.30299082	0.22439001
C	3.17825361	-3.97023854	0.29010722

O	3.06707924	-2.61507967	0.17758111
O	2.21314645	-4.69781798	0.24710334
C	-0.28245880	-2.60201766	0.34113897
H	0.33180703	-3.49650368	0.25134941
H	-0.72233834	-2.48413243	1.32892908
H	0.25060595	-1.71391757	0.01268755
In	2.80878387	0.68857229	-0.16537187
I	1.08179818	0.72006578	2.03750747
I	3.81340676	3.25445129	-0.46370945
I	1.32518231	0.00769114	-2.42525556
C	-0.22139306	3.66653563	-2.07041427
C	-0.77843809	3.31410535	-0.84638249
C	-0.55145195	4.10631458	0.28708452
C	0.21999333	5.26947499	0.25427177
C	0.75986231	5.62186543	-0.97176529
C	0.54394468	4.82722574	-2.11136472
H	-0.36541960	3.05751548	-2.95484105
H	0.39293587	5.85859504	1.14726458
H	1.37610160	6.50979781	-1.05354008
H	1.00139163	5.12274764	-3.04947242
C	-1.63197595	2.20017224	-0.45347767
C	-2.13271174	1.13563755	-1.06201848
H	-1.87331306	1.04587070	-2.11461179
C	-1.26645437	3.45997628	1.34358644
O	-1.90876623	2.40466957	0.93709729
O	-1.33329796	3.83946781	2.56059606
C	-2.06481811	3.01690207	3.51389709
H	-1.94270894	3.52675152	4.46481761
H	-3.11243850	2.96911295	3.21696040
H	-1.62153338	2.02079036	3.53264262
In	-3.36121895	-0.41343033	-0.15959412
I	-5.77882146	-0.71831628	-1.37645378
I	-1.93178814	-2.84929880	-1.04128533

I	-3.38017051	-0.74520857	2.54350608

S24

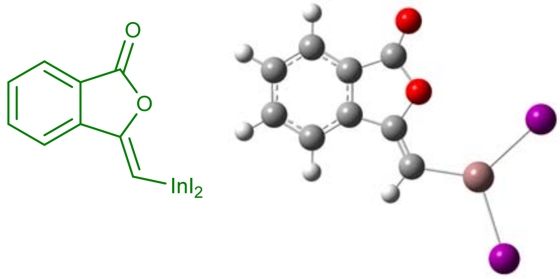

G (348K) = -20078.45334

C	-4.08448001	-2.22745360	0.00057293
C	-3.53355462	-0.95041488	0.00015746
C	-4.34702257	0.17756282	-0.00023656
C	-5.73342176	0.09024369	-0.00022478
C	-6.29041399	-1.18633260	0.00019577
C	-5.47456867	-2.32704888	0.00058968
H	-3.46181889	-3.11583687	0.00087992
H	-6.34997977	0.98254611	-0.00053120
H	-7.36888268	-1.30350936	0.00021796
H	-5.93742366	-3.30858205	0.00091221
C	-2.13995289	-0.48410490	0.00001590
C	-0.98345158	-1.14942259	0.00027773
H	-1.03790095	-2.23442694	0.00063491
C	-3.46896451	1.36428466	-0.00063676
O	-2.16922693	0.90169191	-0.00047174
O	-3.72285813	2.53791684	-0.00103886
In	0.92816435	-0.20009362	0.00006262
I	3.08415281	-1.83140355	-0.00023248
I	1.48467014	2.42430249	0.00028219

TS1-syn-5-*exo* (Fig. S10)

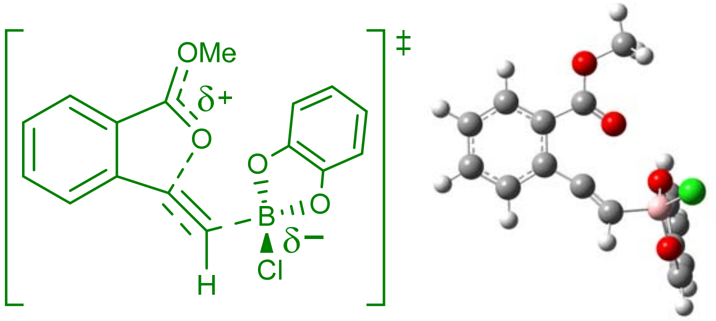

G (398K) = -1402.454406

C	-2.59667587	-2.43927731	0.74288866
C	-2.19307824	-1.12588472	0.46897965
C	-3.03892063	-0.27197022	-0.25373127
C	-4.28926334	-0.70317532	-0.67748554
C	-4.69982730	-1.99983598	-0.37767721
C	-3.85643425	-2.85885611	0.32742903
H	-1.93943020	-3.11096258	1.28333168
H	-4.92449027	-0.03279540	-1.24513946
H	-5.67253833	-2.34741722	-0.70749393
H	-4.17625336	-3.87181998	0.54621941
C	-0.94055790	-0.66483074	0.96974976
C	0.17112241	-0.53820930	1.54609328
H	0.46033041	-1.10679731	2.42650628
C	-2.44178848	1.04964318	-0.53257264
O	-1.28309856	1.26301348	-0.21310235
O	-3.23319899	1.92459558	-1.12250133
C	-2.65454913	3.21416437	-1.40359031
H	-3.44467133	3.78092459	-1.89052507
H	-2.34635171	3.69334080	-0.47349007
H	-1.79285021	3.09878222	-2.06251177
C	4.31158816	-0.80960373	-2.27032031
C	4.99043646	-1.38297928	-1.19681141
C	4.51575760	-1.24953475	0.11780600

C	3.35264045	-0.52693606	0.29834214
C	2.67146113	0.04970510	-0.77853701
C	3.12931491	-0.07776537	-2.07555813
H	4.70212251	-0.92722686	-3.27620663
H	5.90353436	-1.94174732	-1.37604919
H	5.03816316	-1.68827600	0.96094366
H	2.59575388	0.37638423	-2.90340970
O	1.56730769	0.70558209	-0.33816139
O	2.69809287	-0.25831717	1.45786481
B	1.51189246	0.49113792	1.08458382
Cl	1.26217875	2.03264746	2.09365175

S25

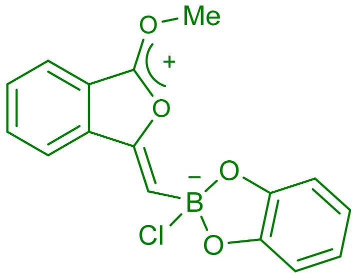

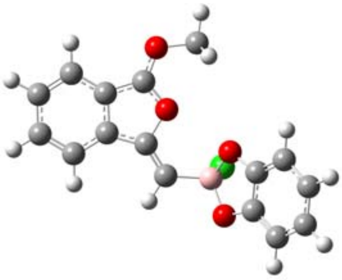

G (398K) = -1402.492169

C	3.32568000	0.33625100	-0.32239200
C	2.63770600	-0.89133600	-0.31306900
C	3.33228400	-2.07546400	-0.58202200
C	4.68829500	-1.98010900	-0.84990100
C	5.36951300	-0.74310400	-0.85713700
C	4.69727500	0.43419900	-0.59206800
C	2.33368600	1.31135800	-0.00152100
C	1.25335800	-0.62844000	0.00699400
H	2.82664800	-3.03453400	-0.57941000
H	5.24830400	-2.88574700	-1.06067500
H	6.43237800	-0.72315300	-1.07122200
H	5.20265900	1.39352200	-0.59061600

O	1.16928200	0.78671400	0.17272700
O	2.49520900	2.58387000	0.12488400
C	0.14620100	-1.34032500	0.22576600
H	0.25110200	-2.41985500	0.12736800
Cl	-1.09719500	-0.48657000	2.56537600
C	-2.88407800	0.42889000	-0.42634800
C	-3.34769100	-0.87782500	-0.22167400
C	-3.68305400	1.39314600	-1.00917700
C	-4.62347800	-1.25575200	-0.59233500
C	-4.98262300	1.01747200	-1.38924200
H	-3.31594100	2.40311600	-1.16060400
C	-5.44305400	-0.28043300	-1.18445200
H	-4.97265500	-2.26888400	-0.42426200
H	-5.63713600	1.75441100	-1.84470800
H	-6.45336800	-0.54479300	-1.48147300
B	-1.28638800	-0.72628900	0.65645300
O	-1.60437600	0.54851600	0.00678600
O	-2.37865000	-1.63706600	0.34336700
C	1.32398500	3.36883200	0.49006700
H	0.56720900	3.27380800	-0.28922500
H	0.92664900	3.00711000	1.43923800
H	1.69265800	4.38769200	0.57090400

X-ray Crystallographic Analysis of Isocoumarin Derivatives

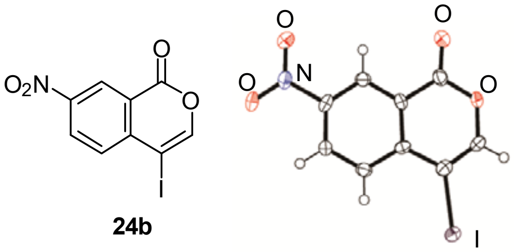

24b

Fig. S11 The X-ray crystallographic structure of halogenation product **24b** with the thermal ellipsoids shown at 50% probability (CCDC 1576343).

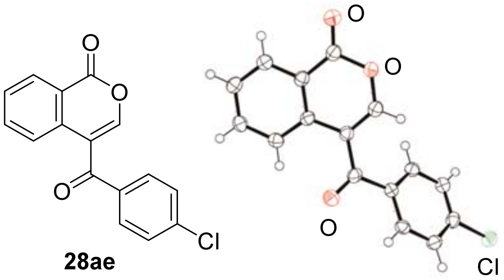

28ae

Fig. S12 The X-ray crystallographic structure of cross coupling product **28ae** with the thermal ellipsoids shown at 50% probability (CCDC 1576344).

Optimization of Reaction Conditions for Oxymetalation of Internal Alkyne

Table S3 The effect of Lewis acids on the oxymetalation of internal alkyne **1i**.[a]

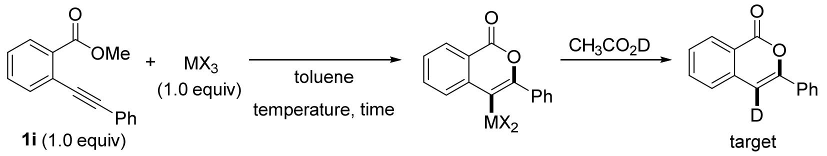

entry	MX_3	temperature	time (h)	NMR yield of target (%)
1	InI_3	50 °C	46	19
2	$InBr_3$	80 °C	25	17
**3**	**GaI_3**	**50 °C**	**23**	**73**
4	$GaBr_3$	50 °C	23	60

[a]Reaction conditions: **1i** (0.5 mmol), MX_3 (0.5 mmol), Toluene (1 mL).

The internal alkyne **1i** was subjected to the oxyindation/protonation process to afford a target product in low yield (entries 1 and 2), but the cases using gallium salts gave high yields (entries 3 and 4).

References

[1] L. C. Wilkins, B. A. R. Günther, M. Walther, J. R. Lawson, T. Wirth and R. L. Melen, *Angew. Chem. Int. Ed.* 2016, **55**, 11292-11295.

[2] Y.-X. Hu, L.-F. Wang, W.-W. Zhang, Y.-Z. Li, X.-M. Ren and J. Bai, *Inorg. Chem. Commun.* 2012, **17**, 173-176.

[3] H. Cho, Y. Iwama, K. Okano and H. Tokuyama, *Chem. Pharm. Bull.* 2014, **62**, 354-363.

[4] K. W. Kuntz, R. Chesworth, K. W. Duncan, H. Keihack, N. Warholic, C. Klaus, W. Zheng, M. Seki, S. Shirotori and S. Kawano, PCT Int. Appl. (2012), WO 2012142504 A1

[5] V. G. Landge, G. Jaiswal and E. Balaraman, *Org. Lett.* 2016, **18**, 812-815.

[6] R. Mancuso, C. C. Pomelli, F. Malafronte, A. Maner, N. Marino, C. Chiappe and B. Gabriele, *Org. Biomol. Chem.* 2017, **15**, 4831-4841.

[7] T. Katsumata, M. Shiotsuki, F. Sanda, X. Sauvage, L. Delaude and T. Masuda, *Macromol. Chem. Phys.* 2009, **210**, 1891-1902.

[8] H. Kusama, H. Funami, J. Takaya, N. Iwasawa, *Org. Lett.* 2004, **6**, 605-608.

[9] F. M. Hauser and V. M. Baghdanov, *J. Org. Chem.* 1988, **53**, 4676-4681.

[10] M. Peuchmaur, V. Lisowski, C. Gandreuil, L. T. Maillard, J. Martinez, J.-F. Hermandez, *J. Org. Chem.* 2009, **74**, 4158-4165.

[11] M. Lessi, T. Masini, L. Nucara, F. Bellina and R. Rossi, *Adv. Synth. Catal.* 2011, **353**, 501-507.

[12] T.-H. Nguyen, A.-S. Castanet and J. Mortier, *Org. Lett.* 2006, **8**, 765-768.

[13] J. L. Seidel, W. W. Epstein, D. W. Davidson, *J. Chem. Ecol.* 1990, **16**, 1791-1816.

[14] Y. C. Fan, O. Kwon, *Org. Lett.* 2012, **14**, 3264-3267.

[15] T. Kovács, I. Sonnenbichler, J. Sonnenbichler, *Liebigs Ann./Recueil* 1997, 773-777.

NMR Spectra

(**1a**) Methyl 2-ethynylbenzoate

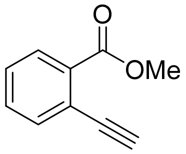

^{1}H NMR (400 MHz, in CD_2Cl_2)

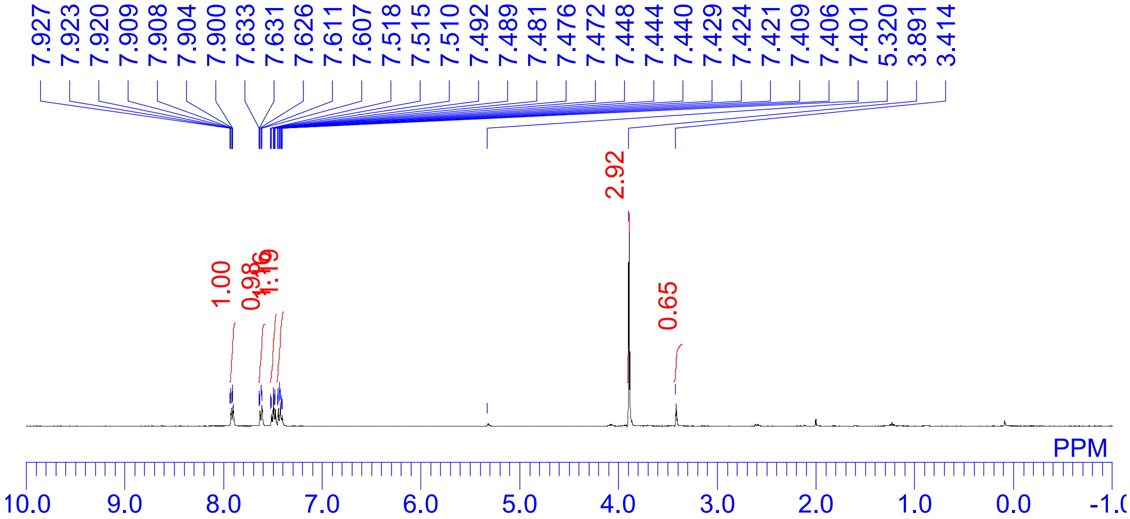

^{13}C NMR (100 MHz, in CD_2Cl_2)

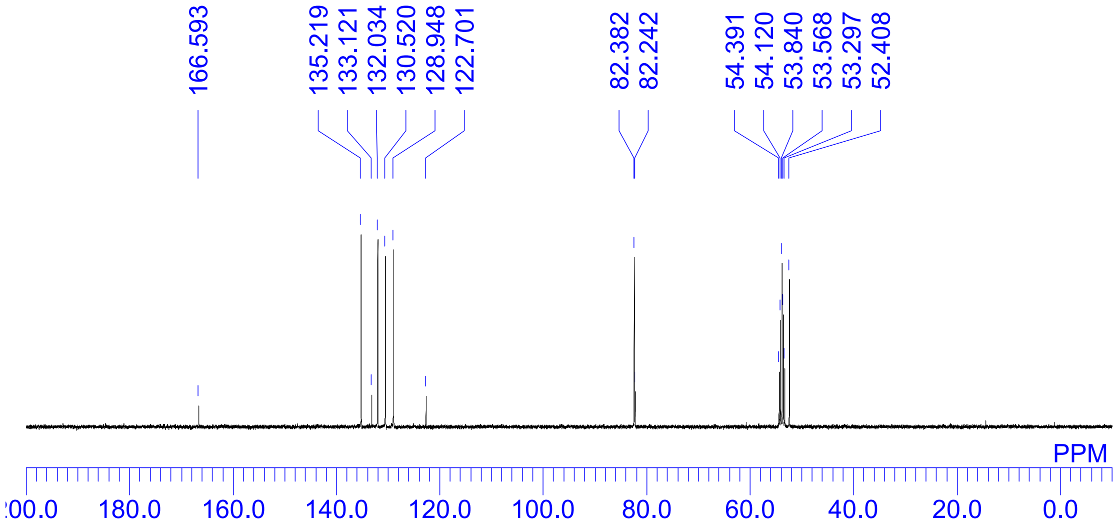

(**1b**) methyl 2-ethynyl-5-nitrobenzoate

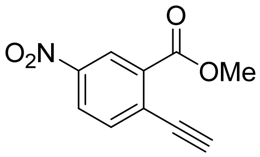

^{1}H NMR (400 MHz, in $CDCl_3$)

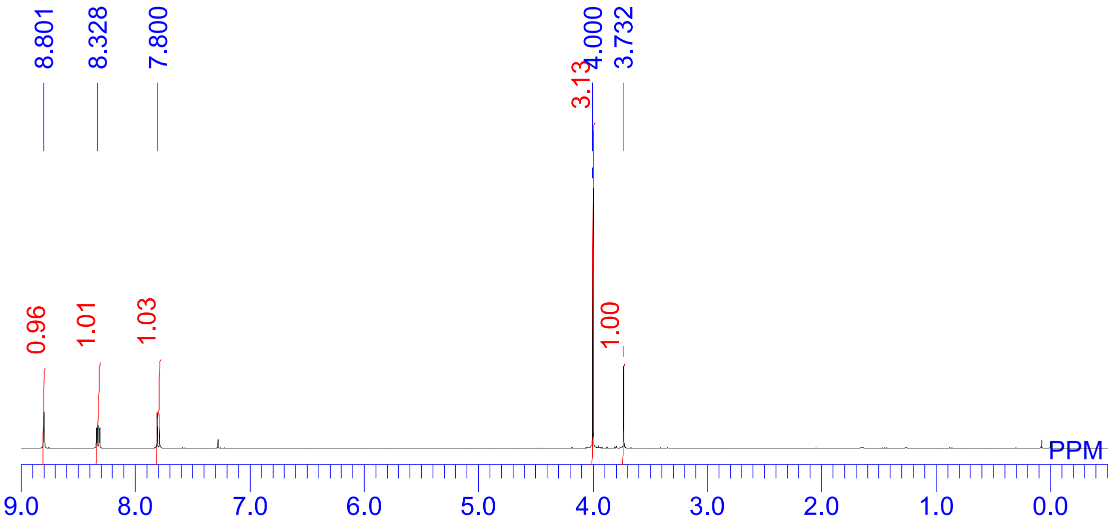

^{13}C NMR (100 MHz, in $CDCl_3$)

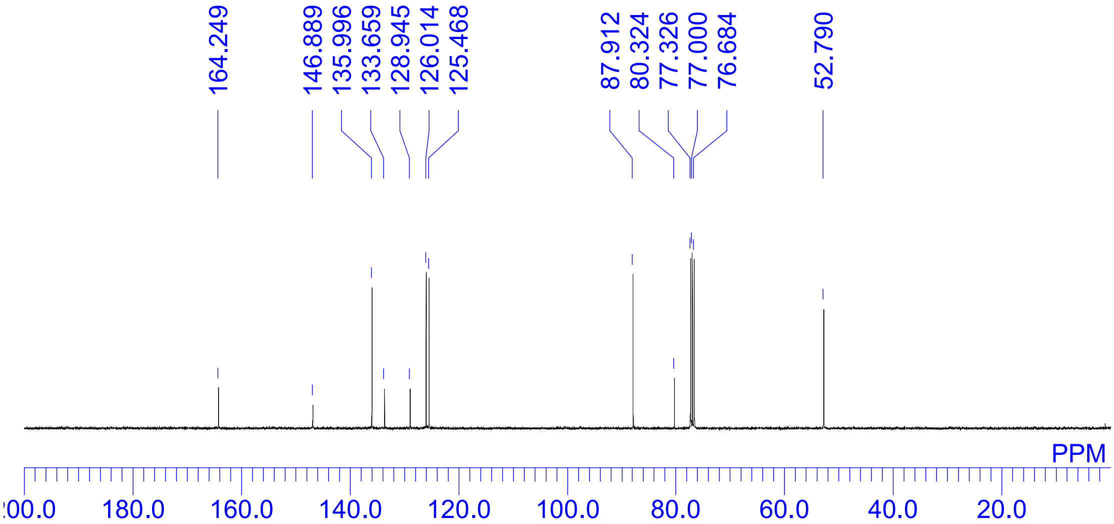

(**1c**) dimethyl 2-ethynylterephthalate

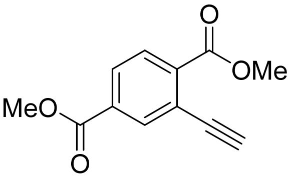

^{1}H NMR (400 MHz, in $CDCl_3$)

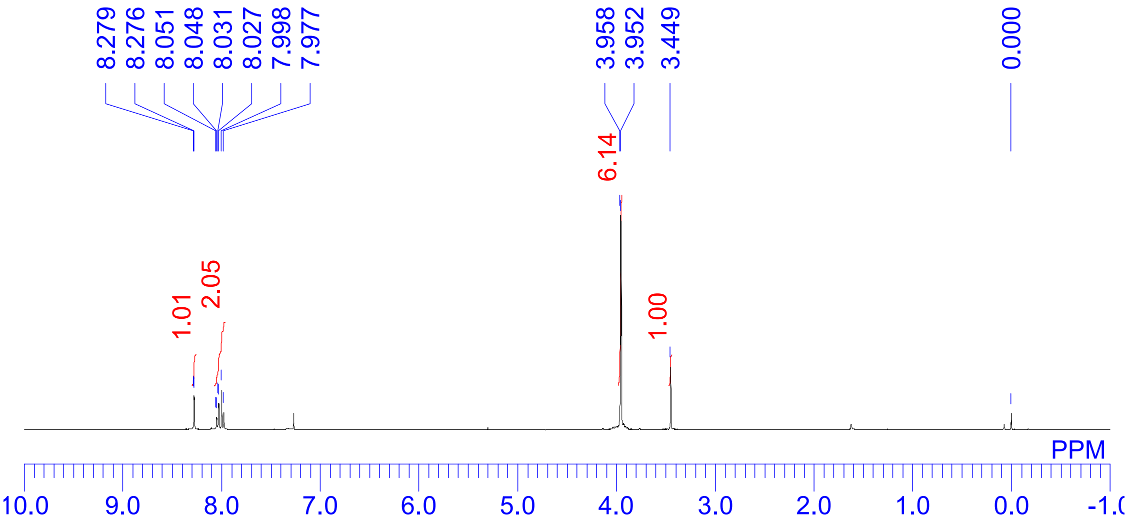

^{13}C NMR (100 MHz, in $CDCl_3$)

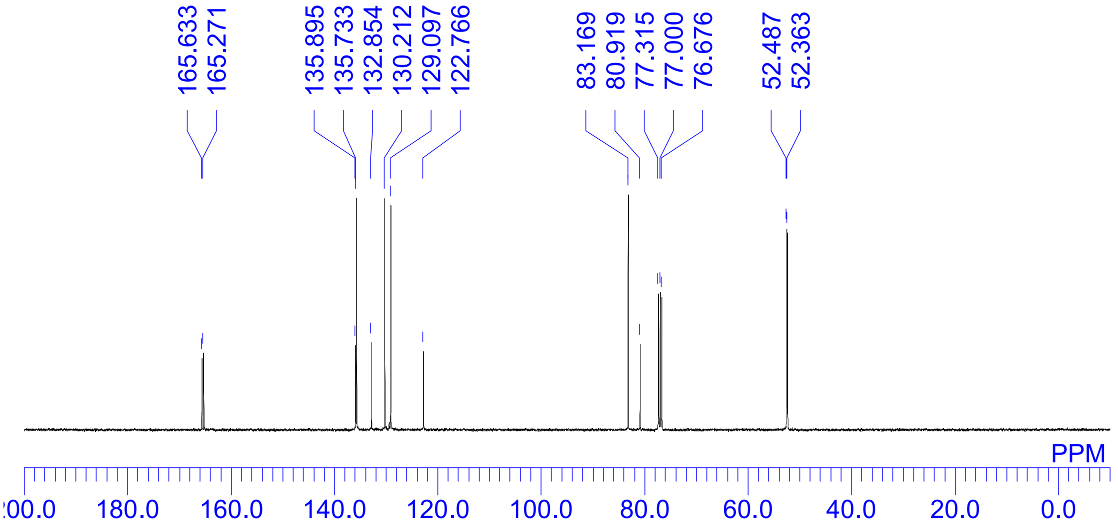

(**1d**) methyl 2-ethynyl-5-methylbenzoate

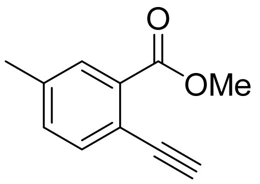

^{1}H NMR (400 MHz, in $CDCl_3$)

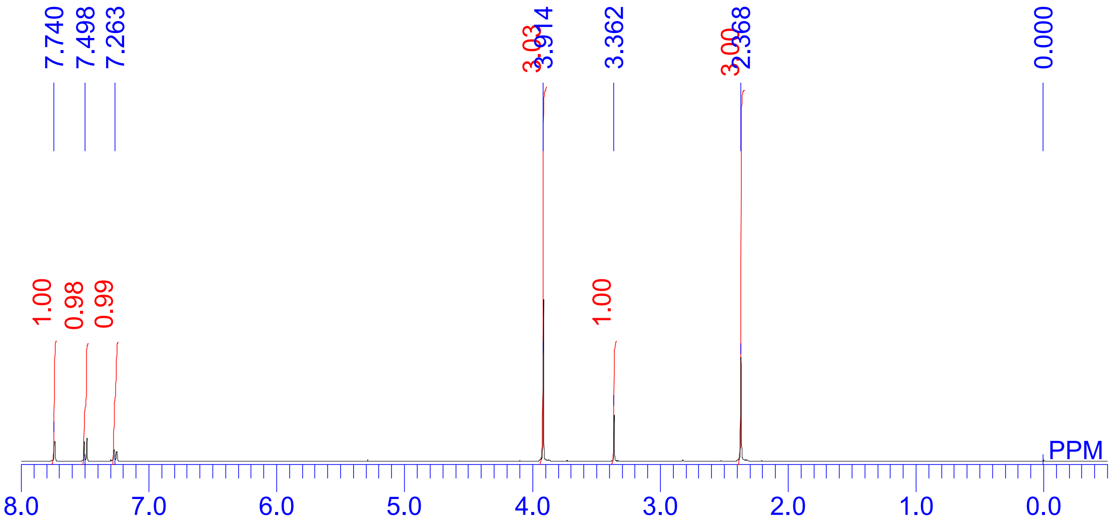

^{13}C NMR (100 MHz, in $CDCl_3$)

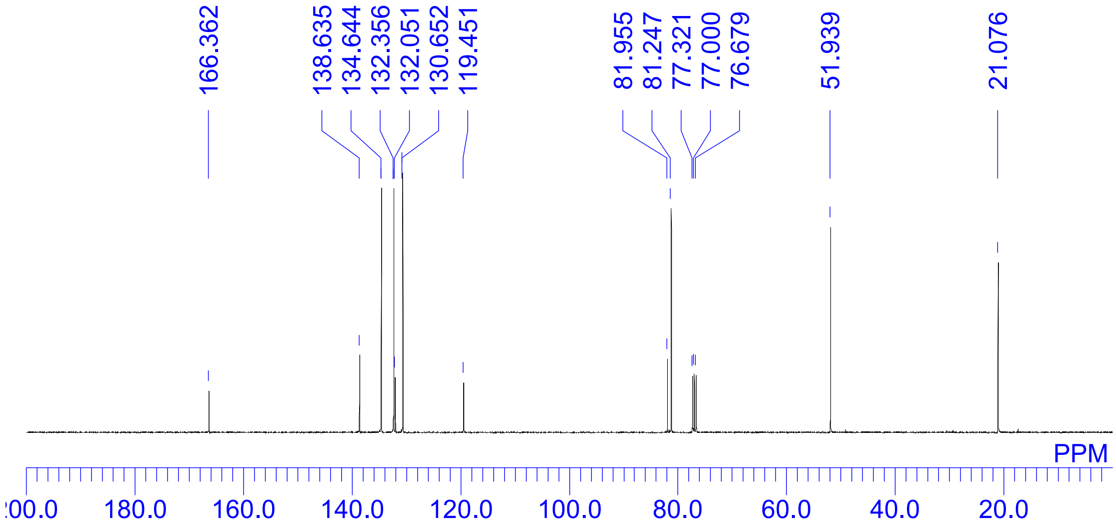

(**1e** precursor) 4'-(*tert*-butyl)-4-((trimethylsilyl)ethynyl)-[1,1'-biphenyl]-3-carboxylate

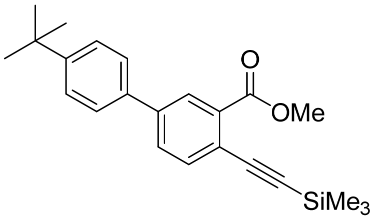

^{1}H NMR (400 MHz, in $CDCl_3$)

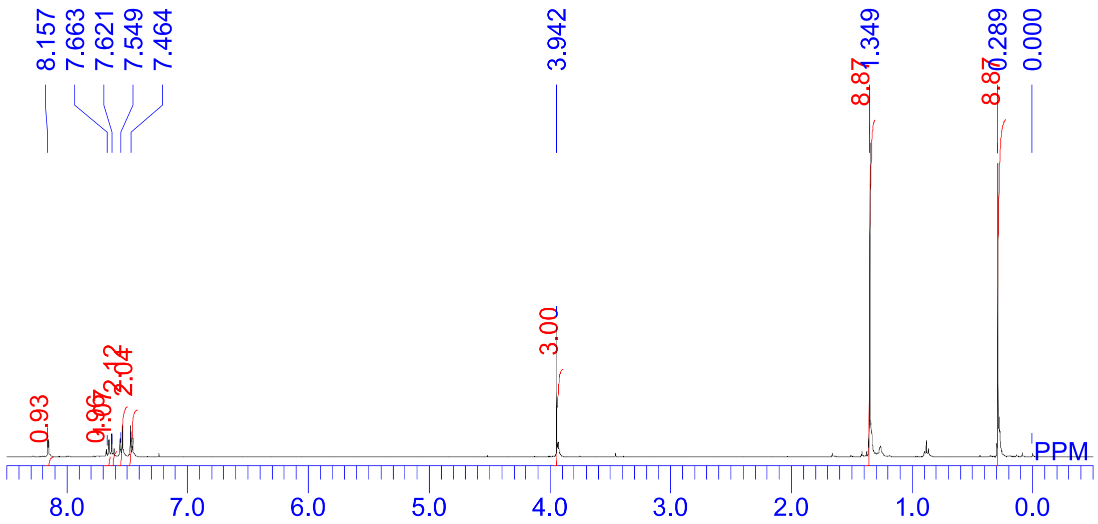

^{13}C NMR (100 MHz, in $CDCl_3$)

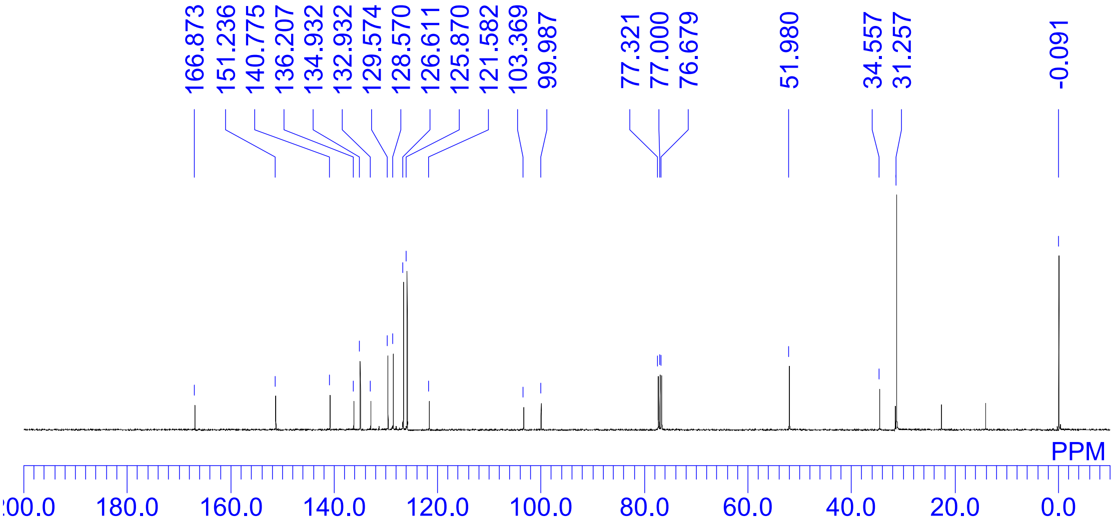

(**1e**) methyl 4'-(*tert*-butyl)-4-ethynyl-[1,1'-biphenyl]-3-carboxylate

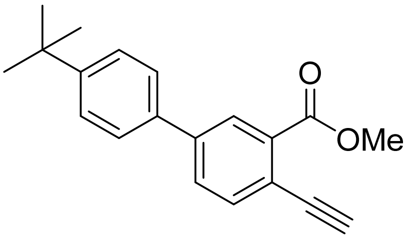

^{1}H NMR (400 MHz, in $CDCl_3$)

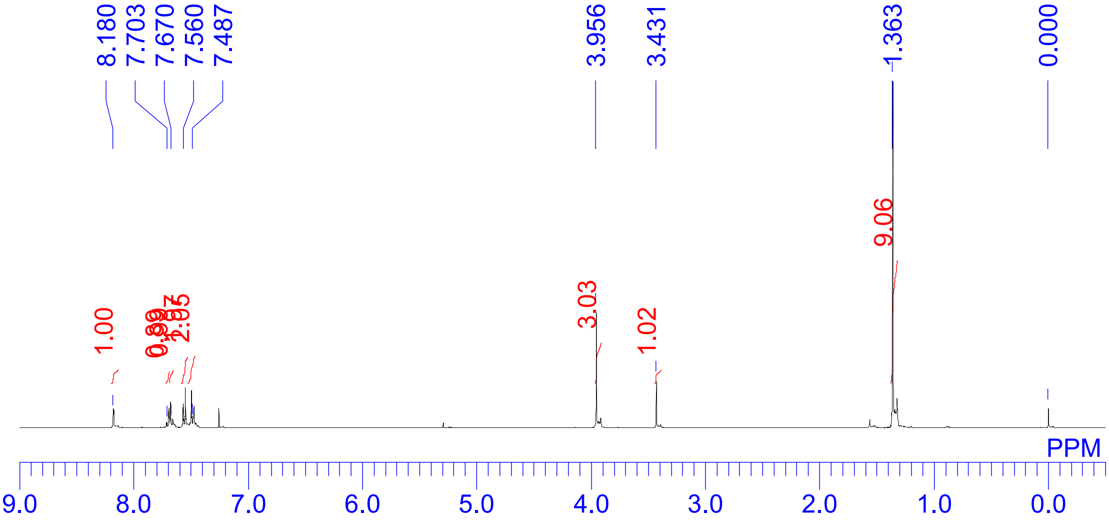

^{13}C NMR (100 MHz, in $CDCl_3$)

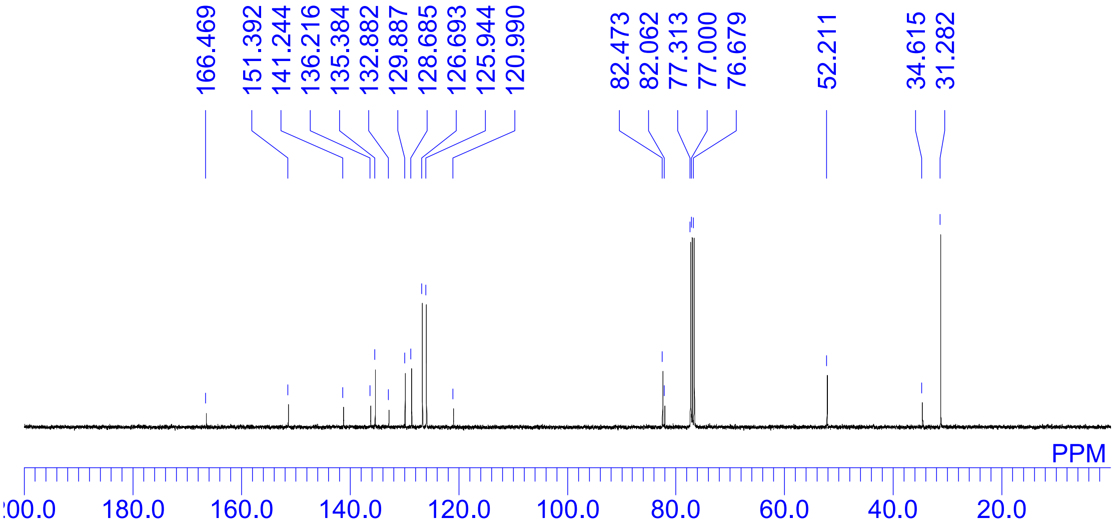

(**1f**) methyl 5-bromo-2-ethynylbenzoate

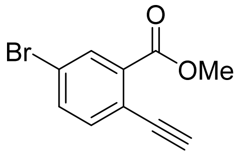

^{1}H NMR (400 MHz, in $CDCl_3$)

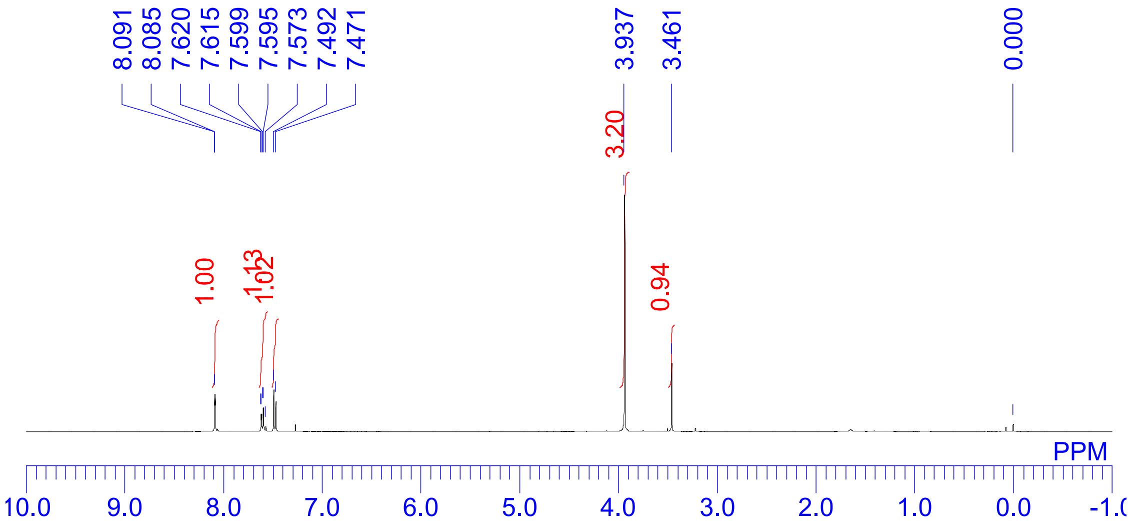

^{13}C NMR (100 MHz, in $CDCl_3$)

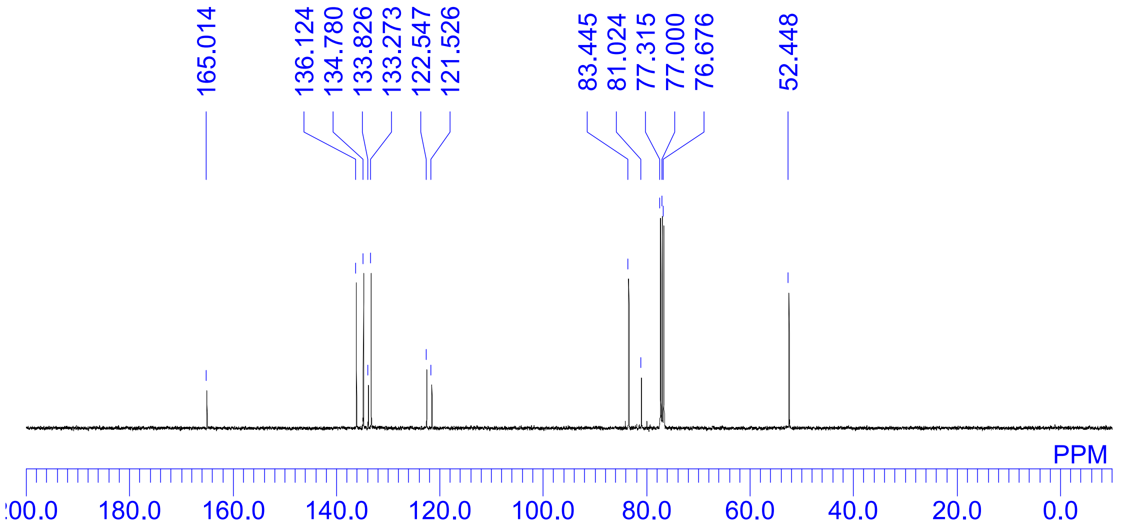

(**1g**) methyl 5-chloro-2-ethynylbenzoate

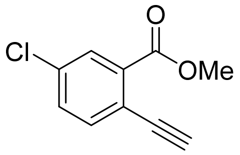

^{1}H NMR (400 MHz, in $CDCl_3$)

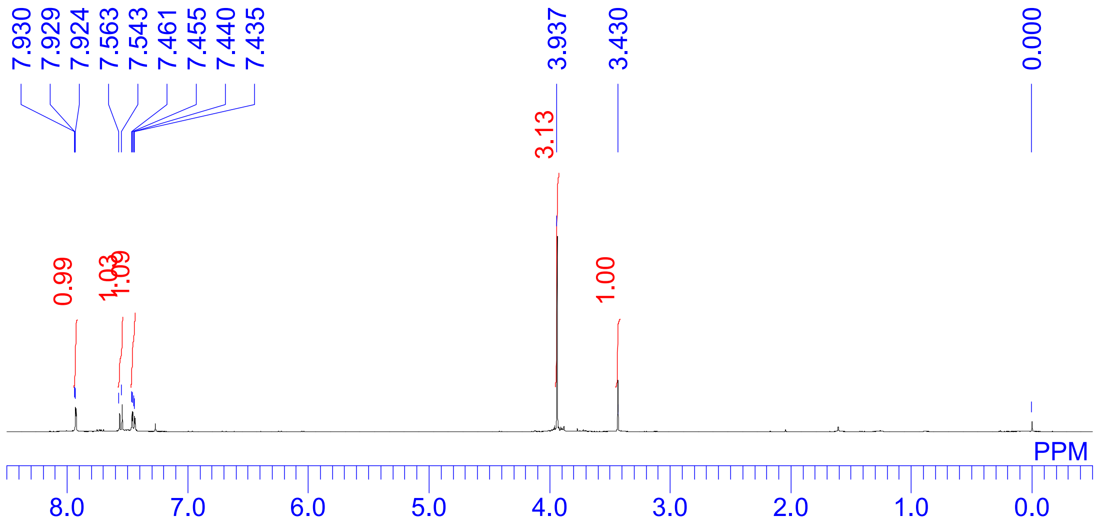

^{13}C NMR (100 MHz, in $CDCl_3$)

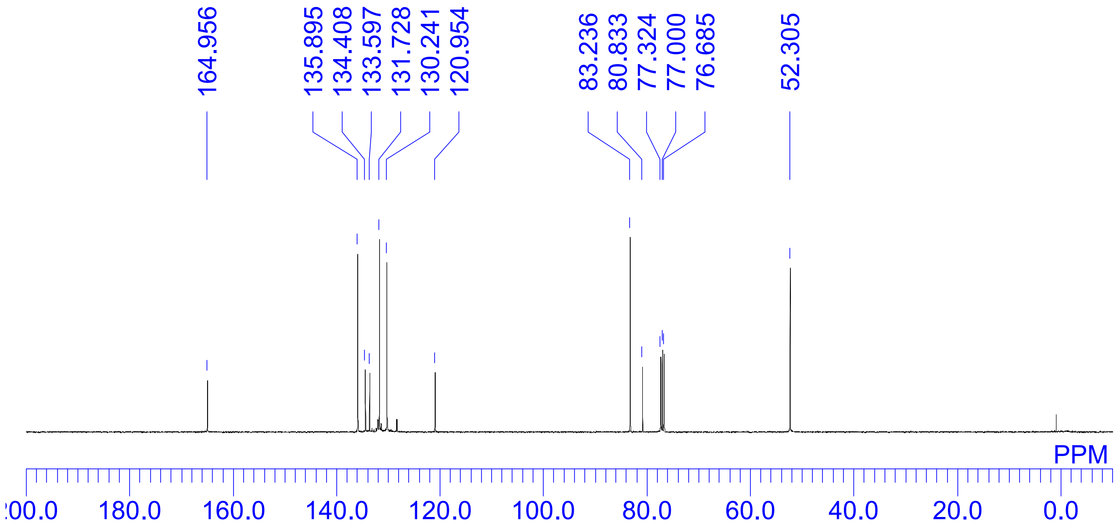

(**1h**) methyl 2-ethynyl-5-fluorobenzoate

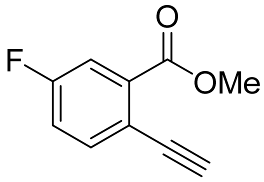

^{1}H NMR (400 MHz, in $CDCl_3$)

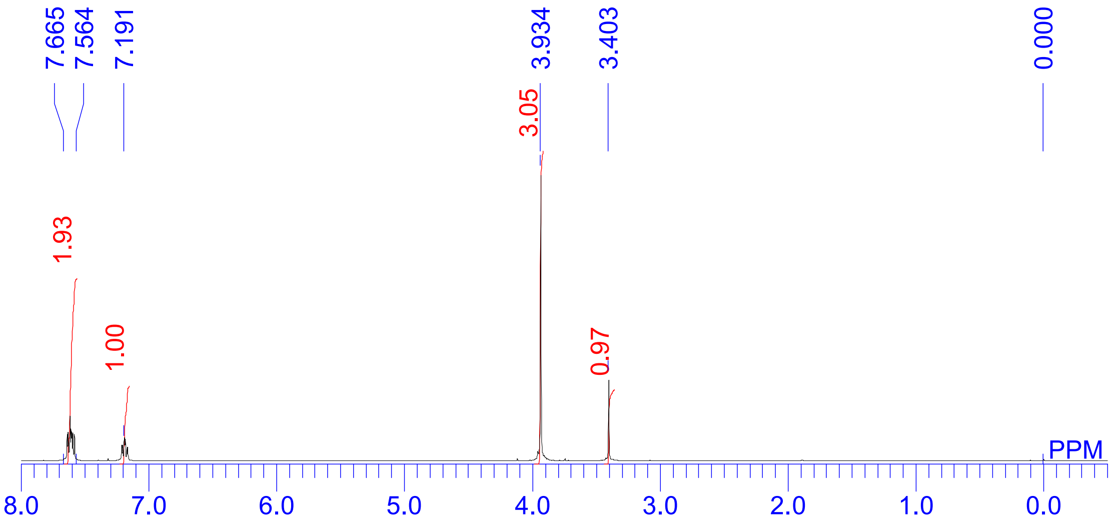

^{13}C NMR (100 MHz, in $CDCl_3$)

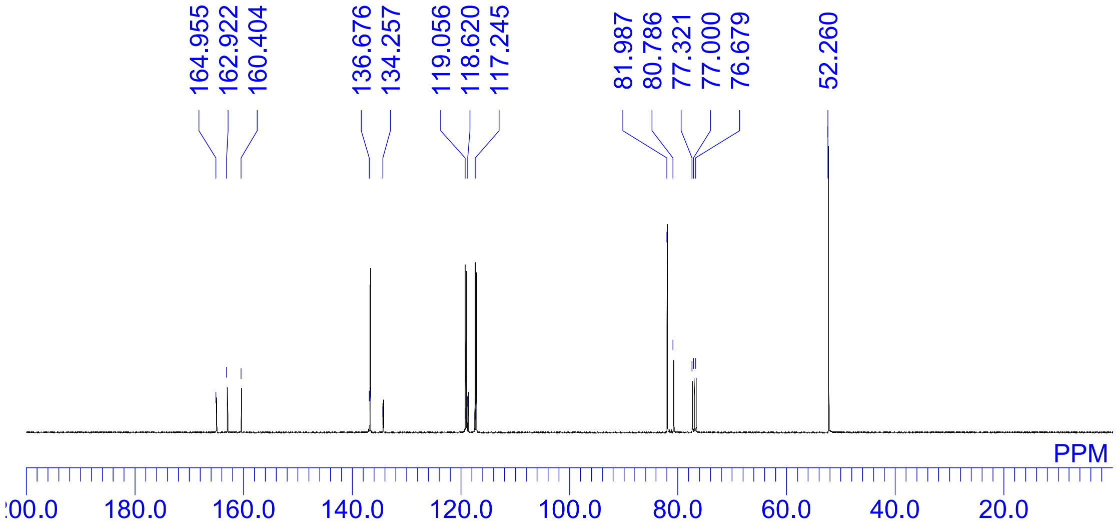

(**1i**) methyl 2-(phenylethynyl)benzoate

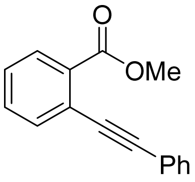

^{1}H NMR (400 MHz, in $CDCl_3$)

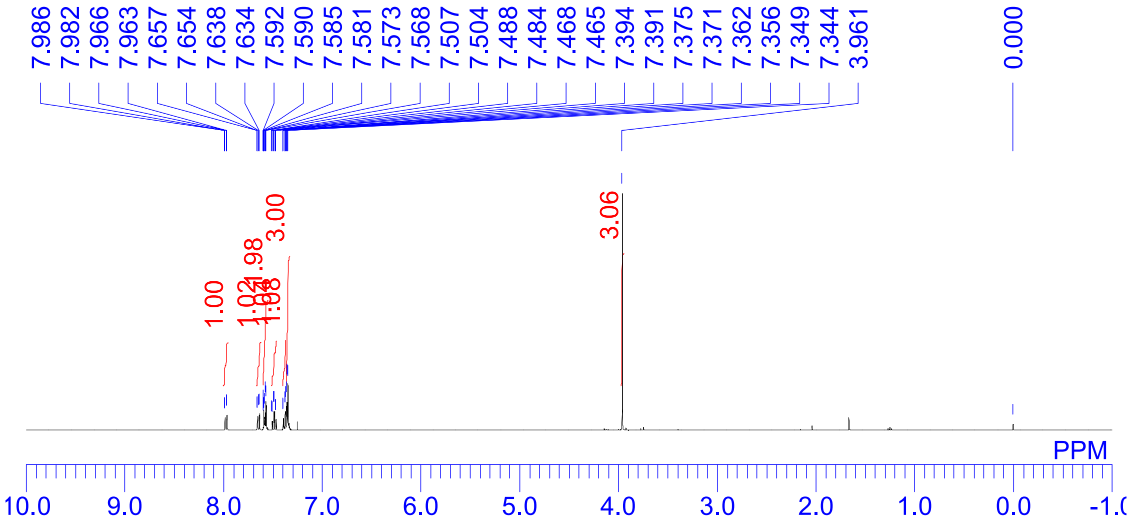

^{13}C NMR (100 MHz, in $CDCl_3$)

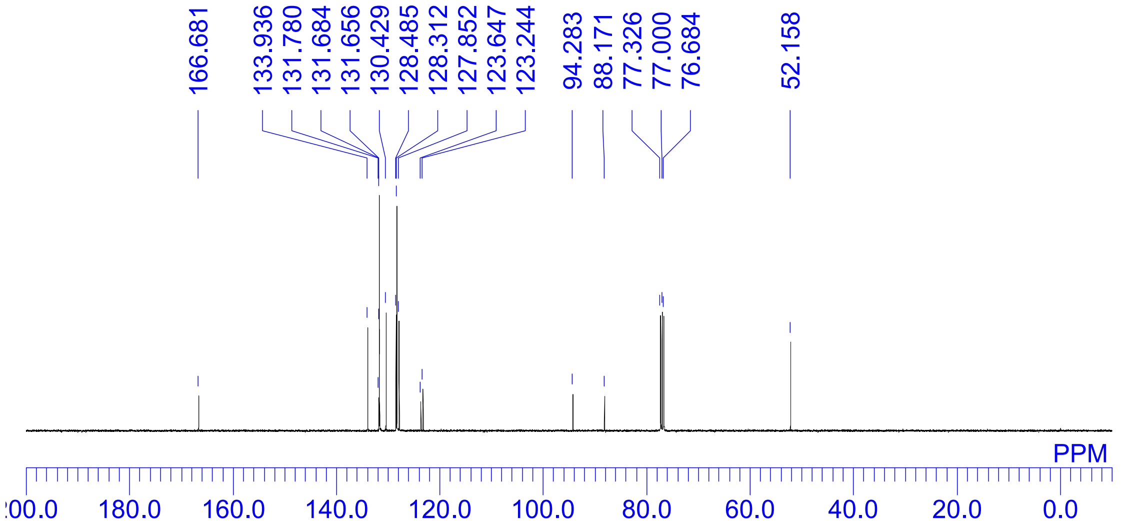

(**1j**) methyl 2-((4-fluorophenyl)ethynyl)benzoate

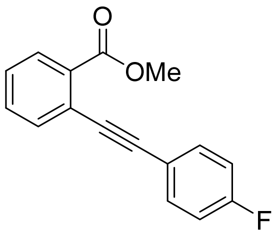

^{1}H NMR (400 MHz, in $CDCl_3$)

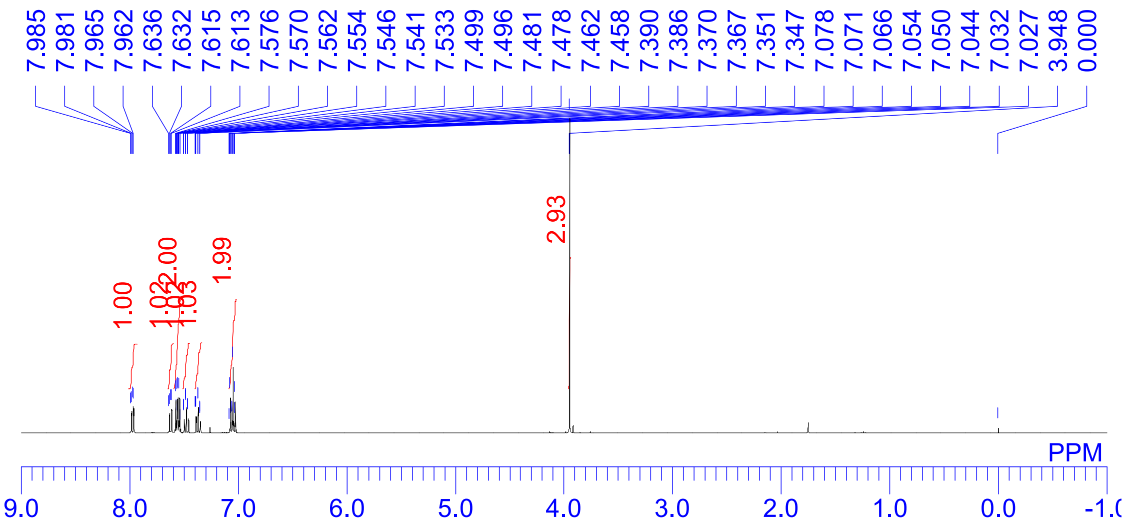

^{13}C NMR (100 MHz, in $CDCl_3$)

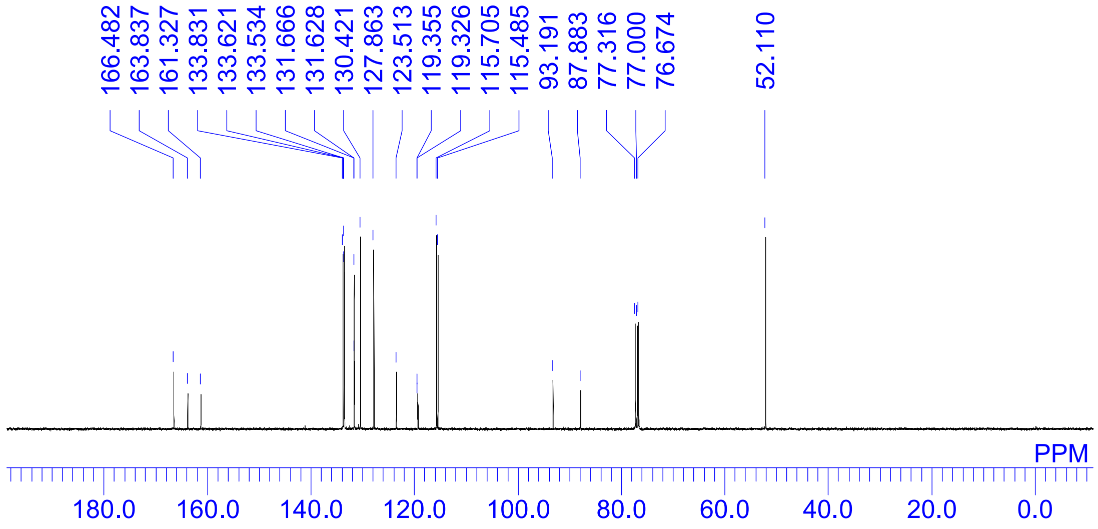

(**1k**) methyl 2-(hex-1-yn-1-yl)benzoate

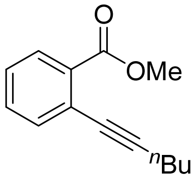

^{1}H NMR (400 MHz, in $CDCl_3$)

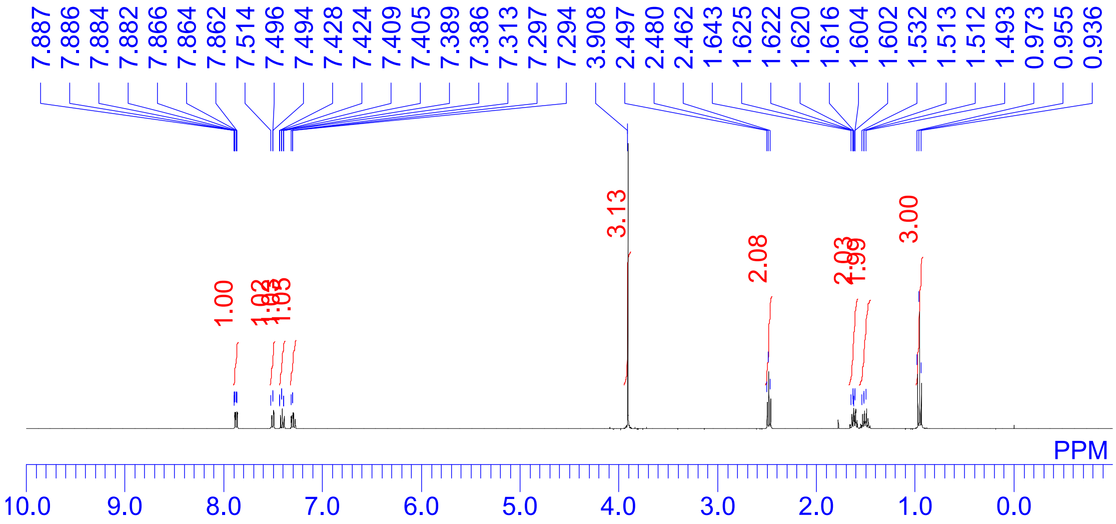

^{13}C NMR (100 MHz, in $CDCl_3$)

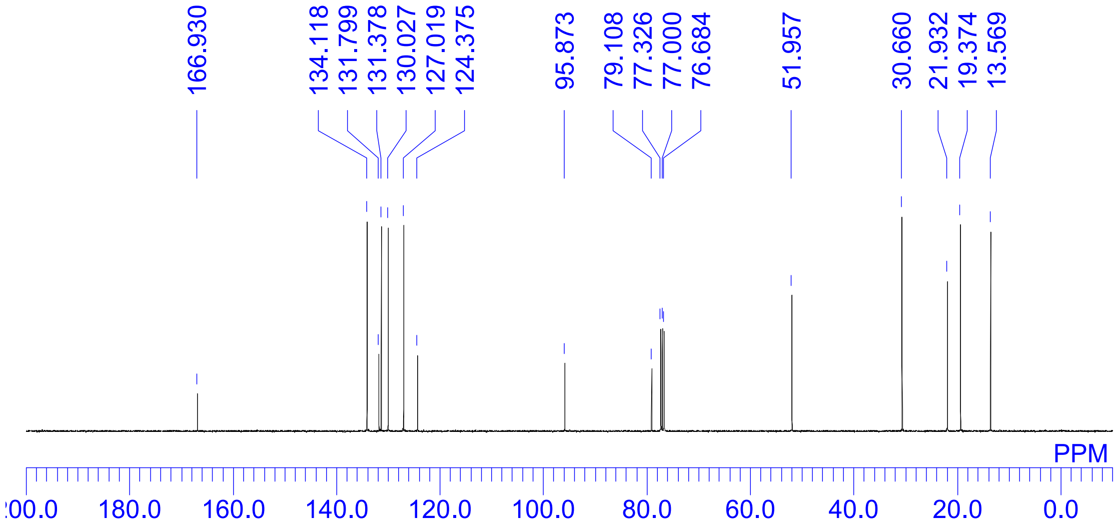

(**1l**) Ethyl 2-ethynylbenzoate

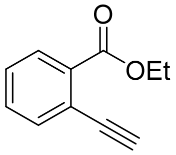

^{1}H NMR (400 MHz, in $CDCl_3$)

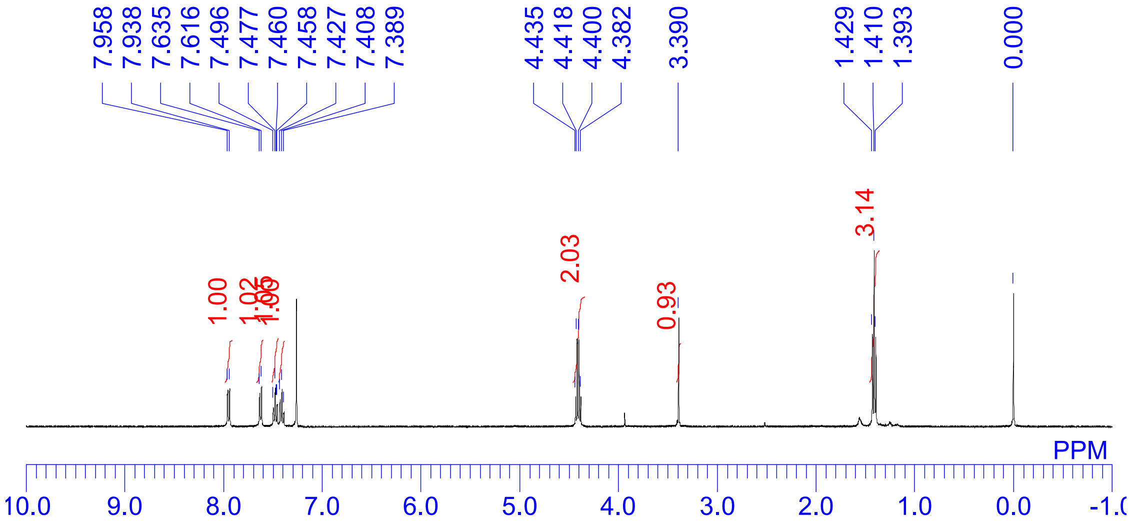

^{13}C NMR (100 MHz, in $CDCl_3$)

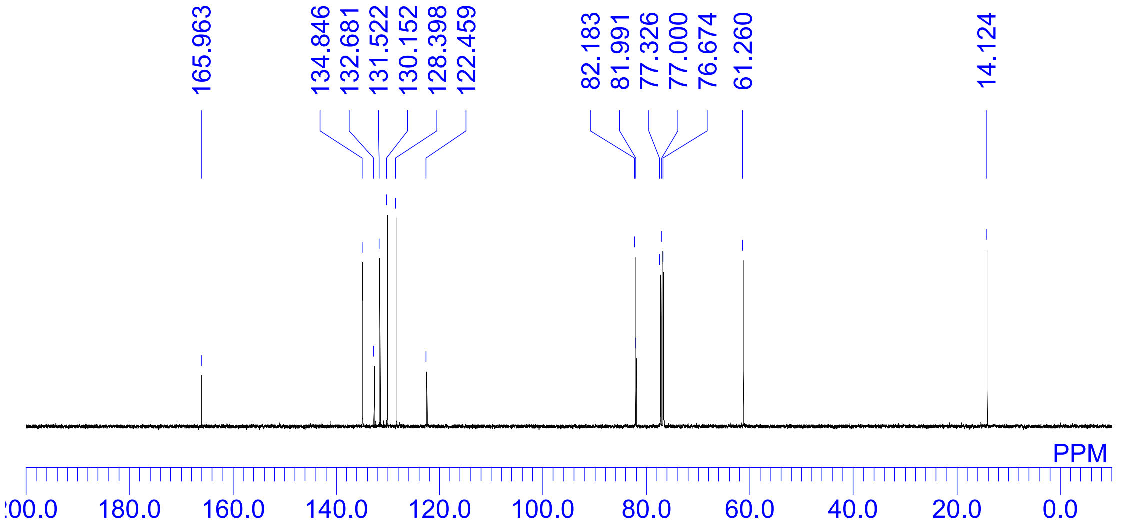

(**1m**) Isopropyl 2-ethynylbenzoate

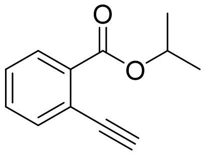

^{1}H NMR (400 MHz, in $CDCl_3$)

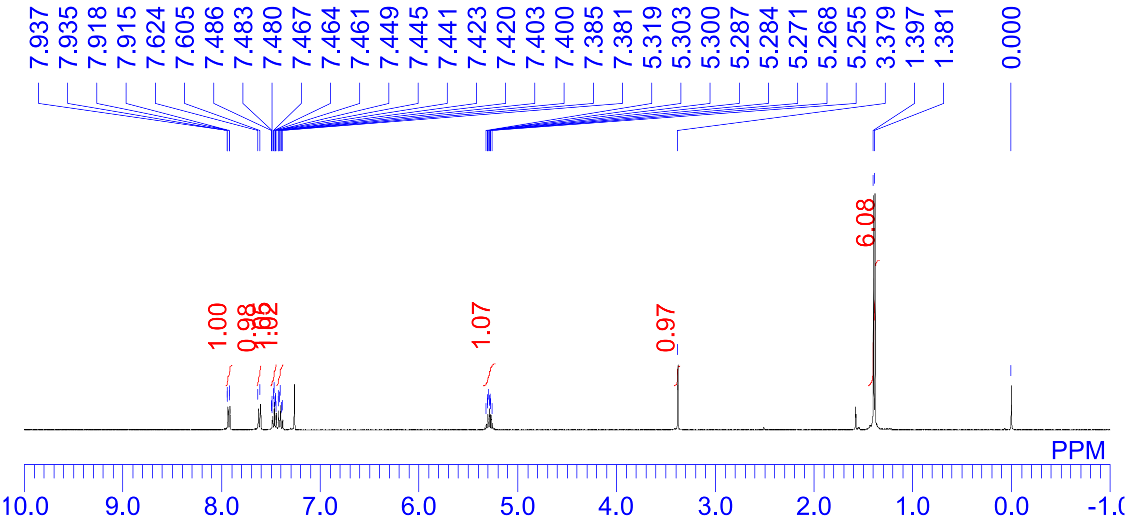

^{13}C NMR (100 MHz, in $CDCl_3$)

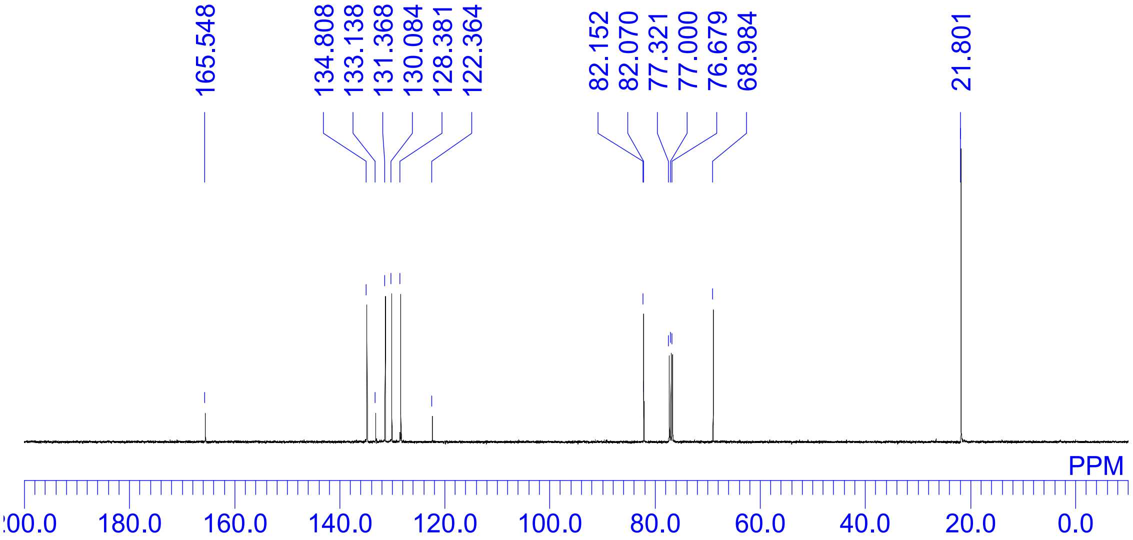

(**2**) Isocoumarin (Scheme 7)

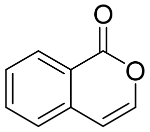

^{1}H NMR (400 MHz, in $CDCl_3$)

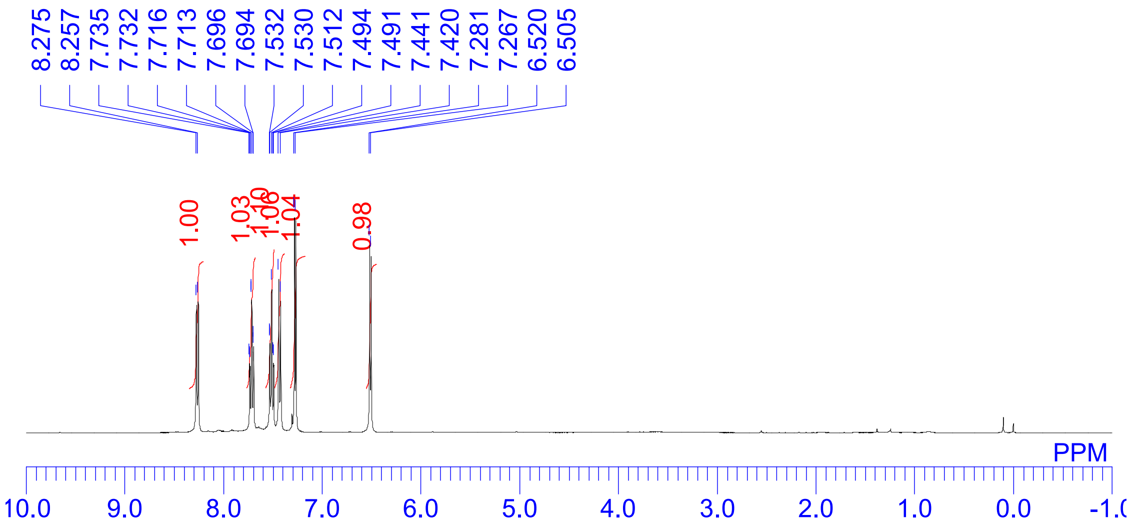

^{13}C NMR (100 MHz, in $CDCl_3$)

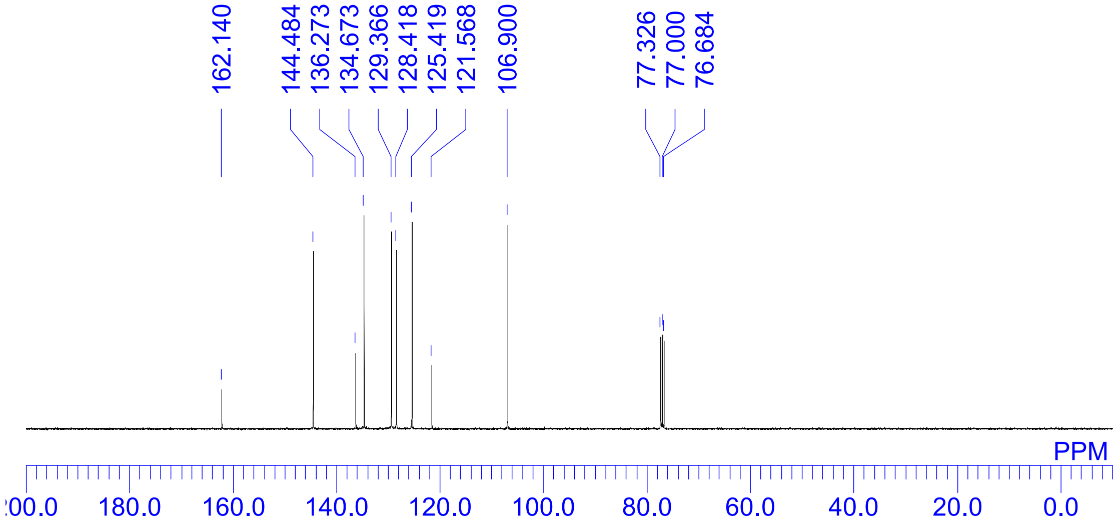

(**24a**) 4-iodo-1*H*-isochromen-1-one

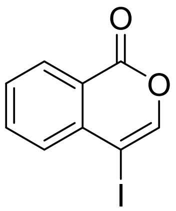

^{1}H NMR (400 MHz, in $CDCl_3$)

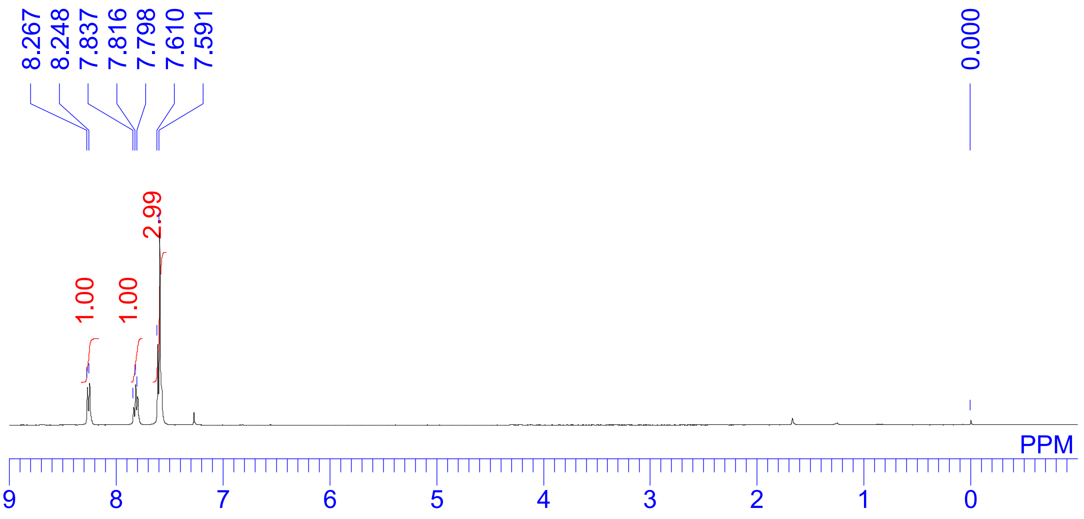

^{13}C NMR (100 MHz, in $CDCl_3$)

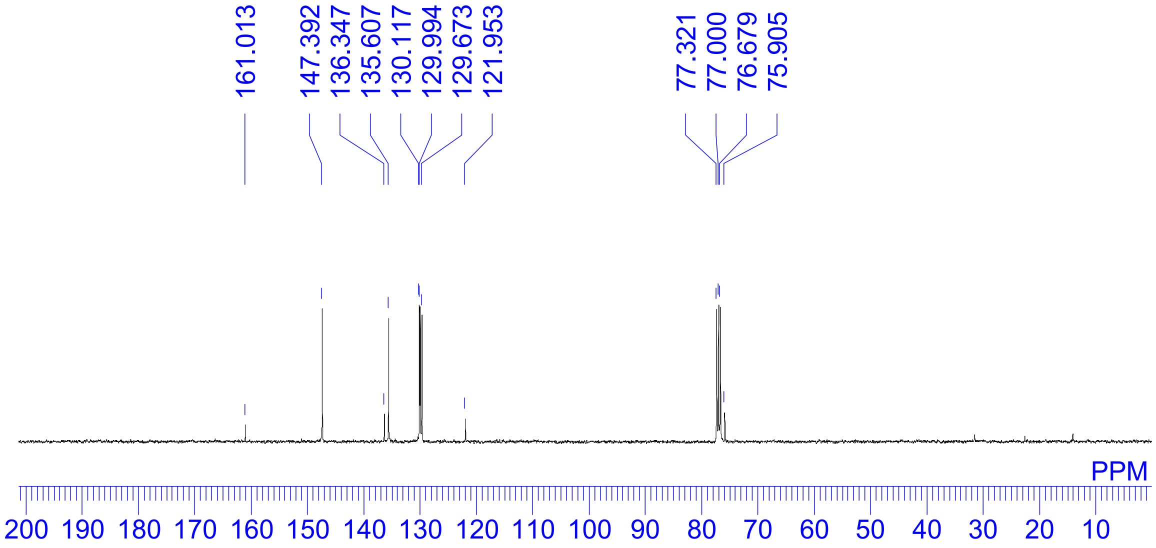

(**25a**) 4-bromo-1*H*-isochromen-1-one

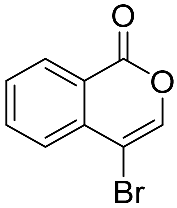

^{1}H NMR (400 MHz, in $CDCl_3$)

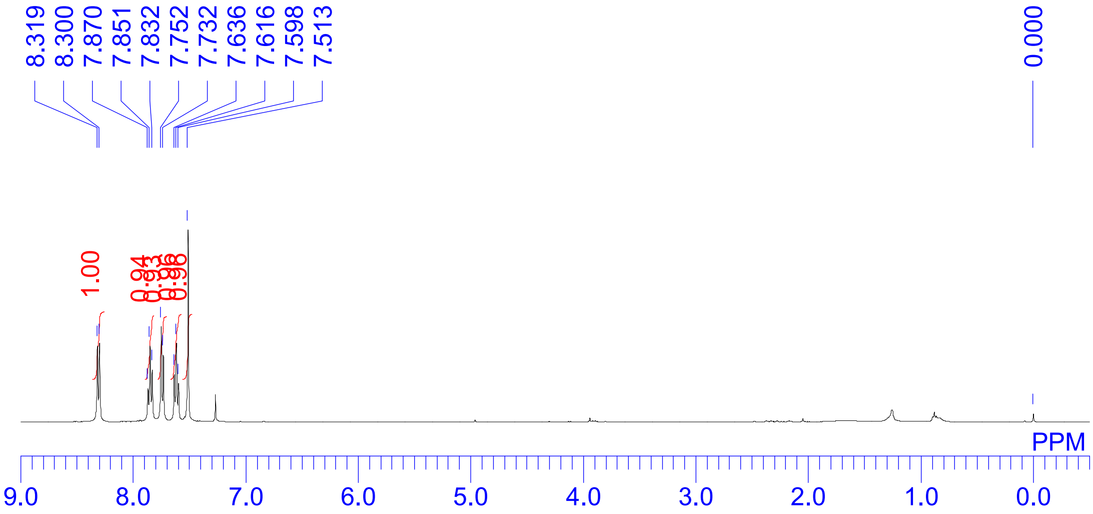

^{13}C NMR (100 MHz, in $CDCl_3$)

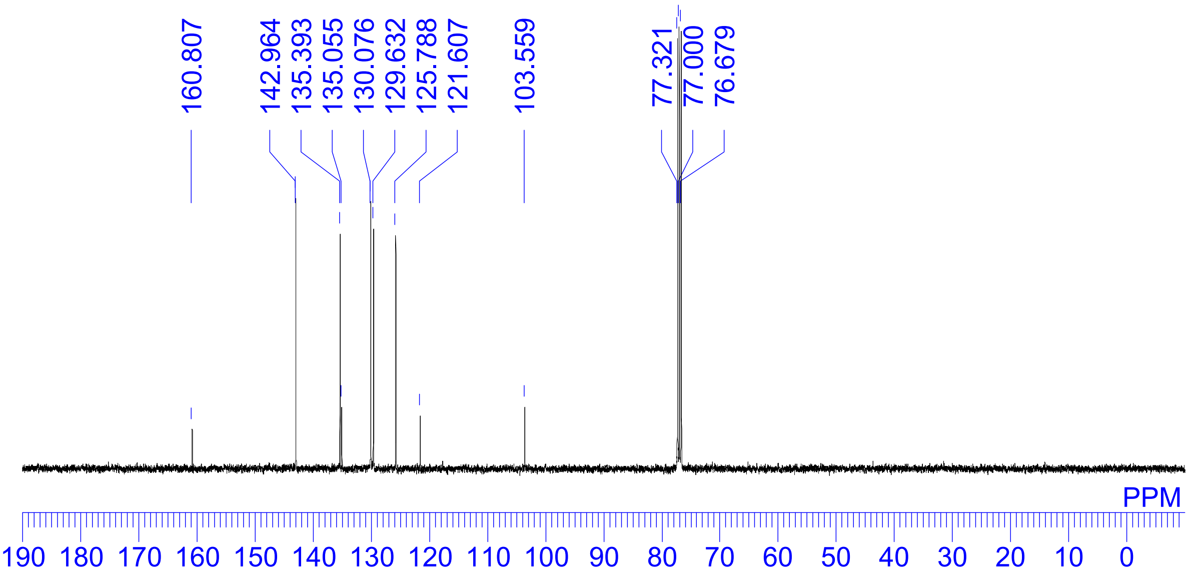

(**24b**) 4-iodo-7-nitro-1*H*-isochromen-1-one

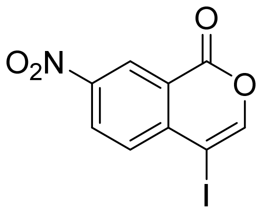

^{1}H NMR (400 MHz, in $CDCl_3$)

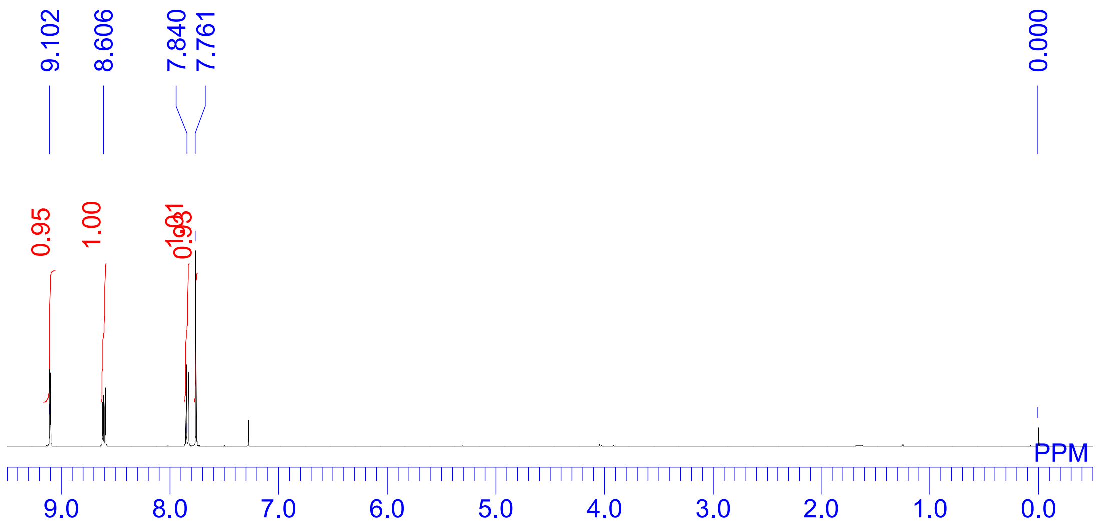

^{13}C NMR (100 MHz, in $CDCl_3$)

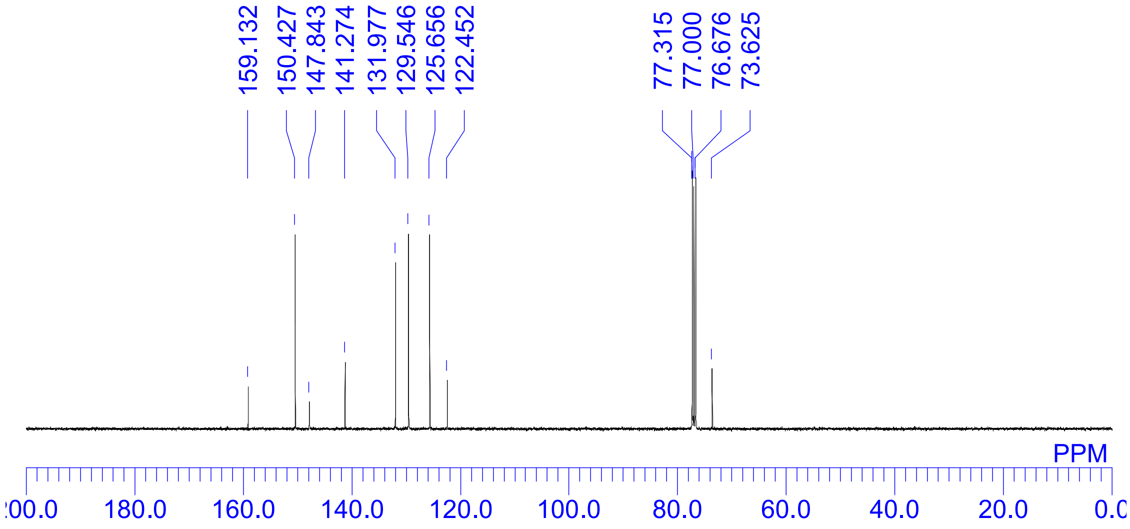

(**24c**) methyl 4-iodo-1-oxo-1*H*-isochromene-6-carboxylate

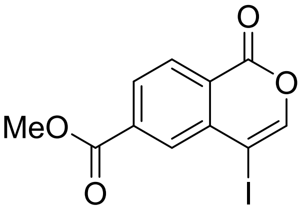

^{1}H NMR (400 MHz, in $CDCl_3$)

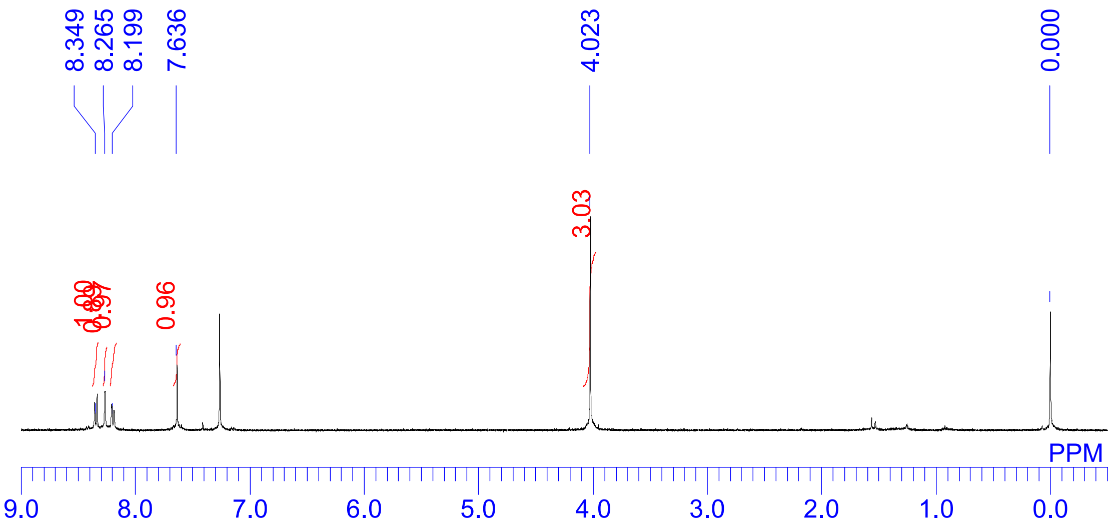

^{13}C NMR (100 MHz, in $CDCl_3$)

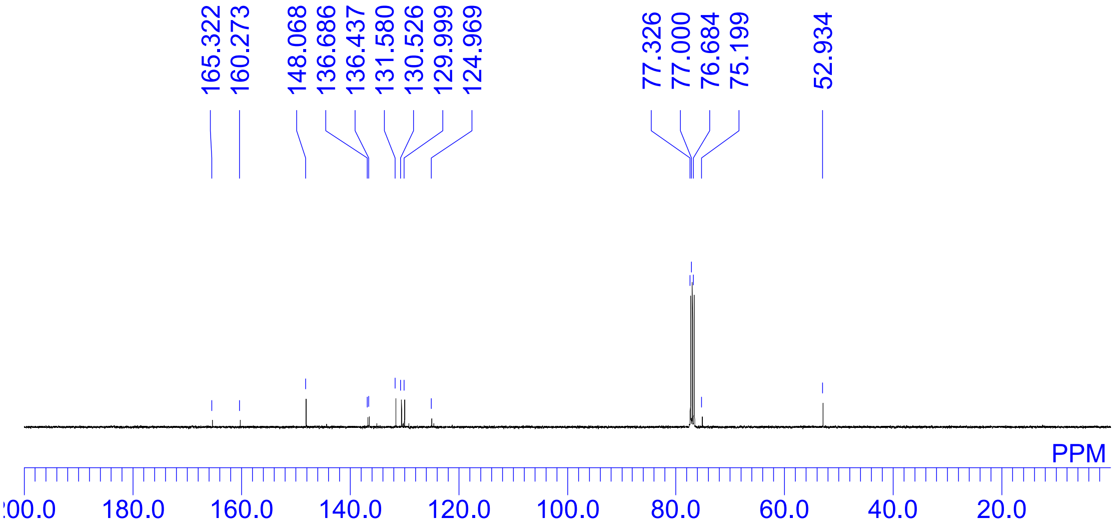

(**24d**) 4-iodo-7-methyl-1*H*-isochromen-1-one

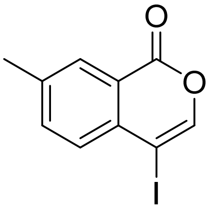

^{1}H NMR (400 MHz, in $CDCl_3$)

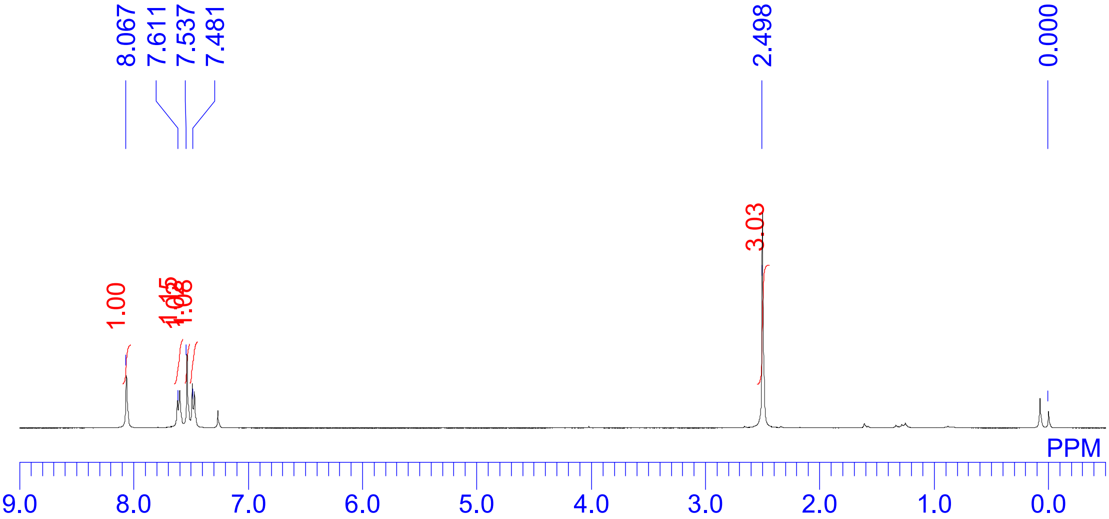

^{13}C NMR (100 MHz, in $CDCl_3$)

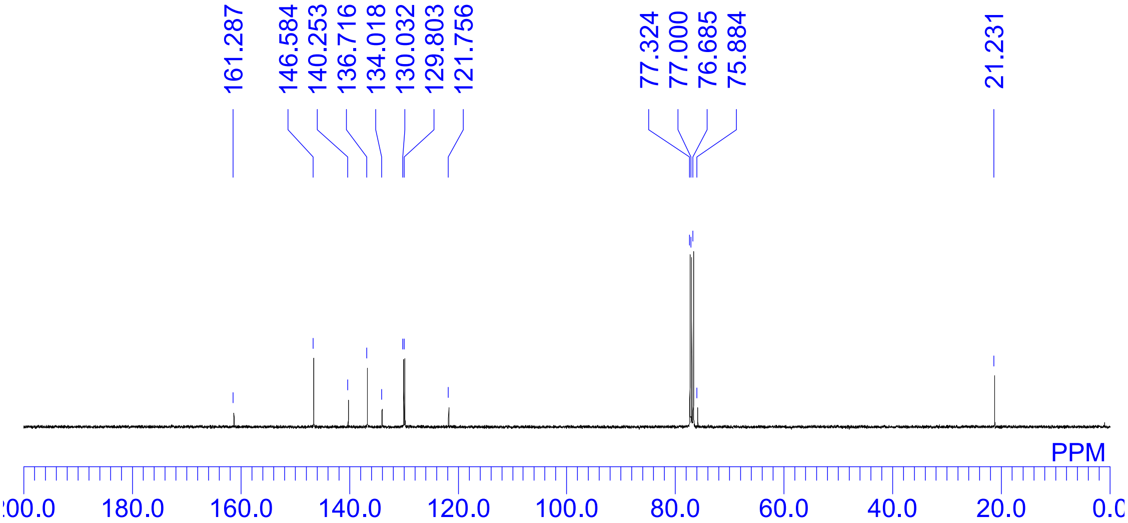

(**24e**) 7-(4-(*tert*-butyl)phenyl)-4-iodo-1*H*-isochromen-1-one

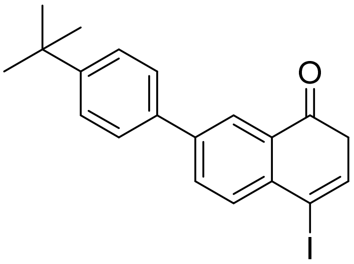

^{1}H NMR (400 MHz, in $CDCl_3$)

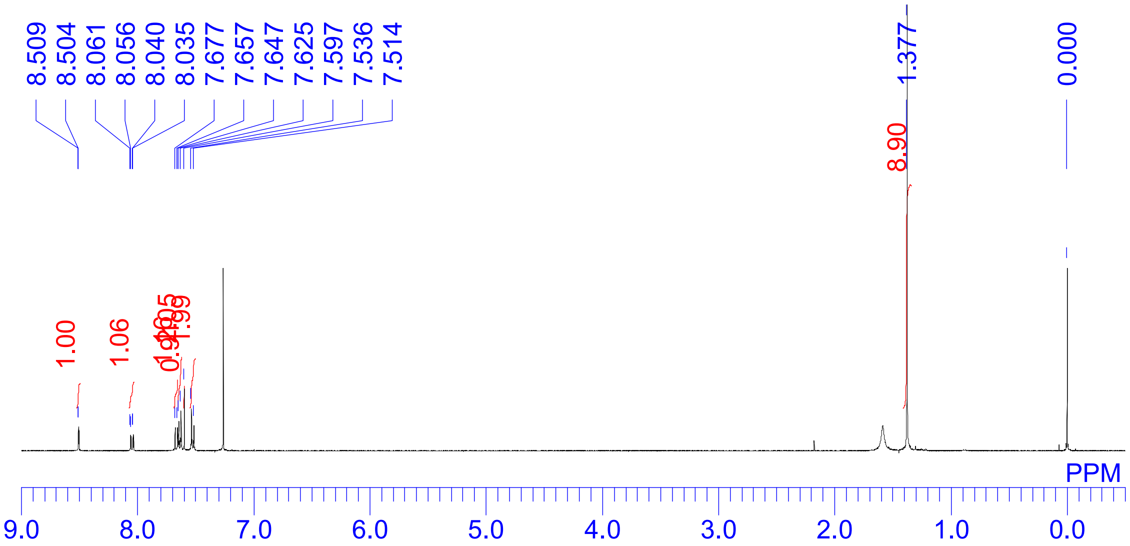

^{13}C NMR (100 MHz, in $CDCl_3$)

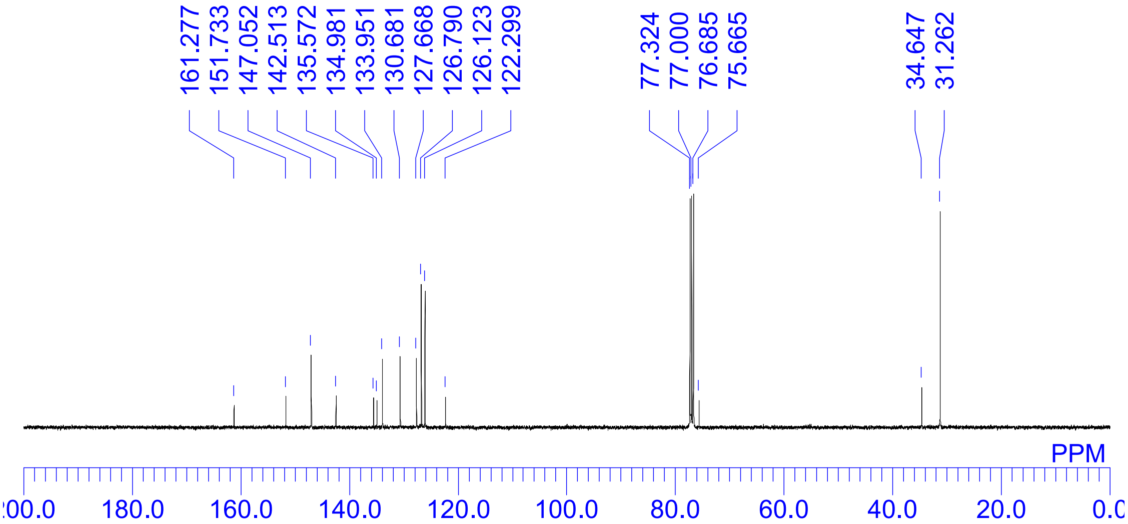

(**25f**) 4,7-dibromo-1*H*-isochromen-1-one

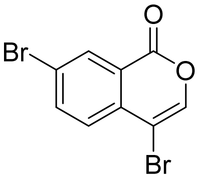

^{1}H NMR (400 MHz, in $CDCl_3$)

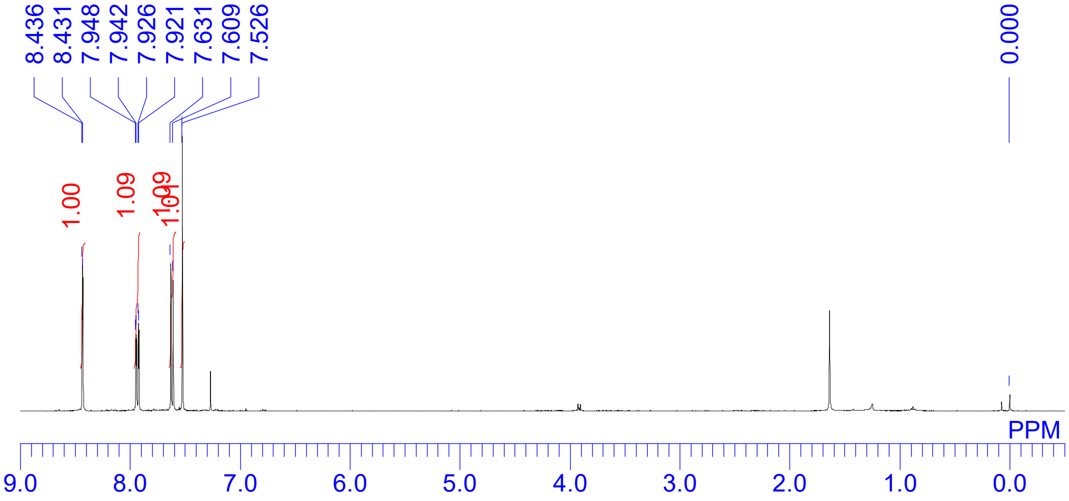

^{13}C NMR (100 MHz, in $CDCl_3$)

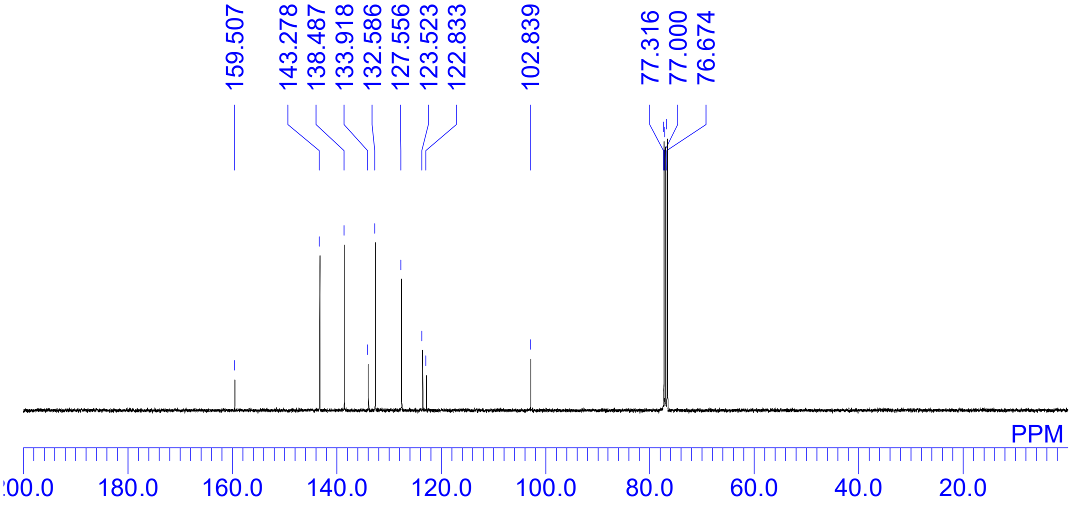

(**24g**) 7-chloro-4-iodo-1*H*-isochromen-1-one

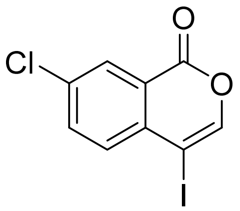

^{1}H NMR (400 MHz, in $CDCl_3$)

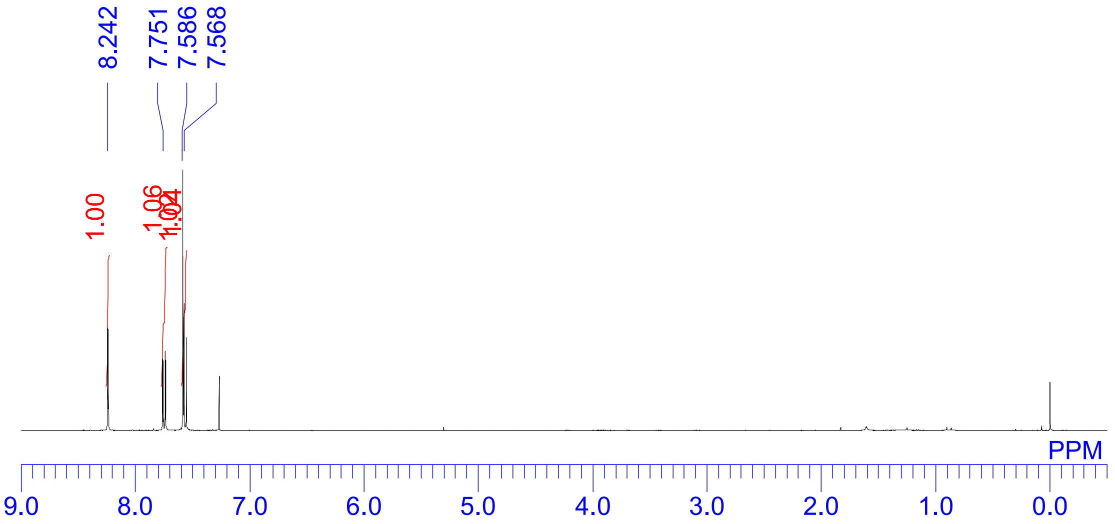

^{13}C NMR (100 MHz, in $CDCl_3$)

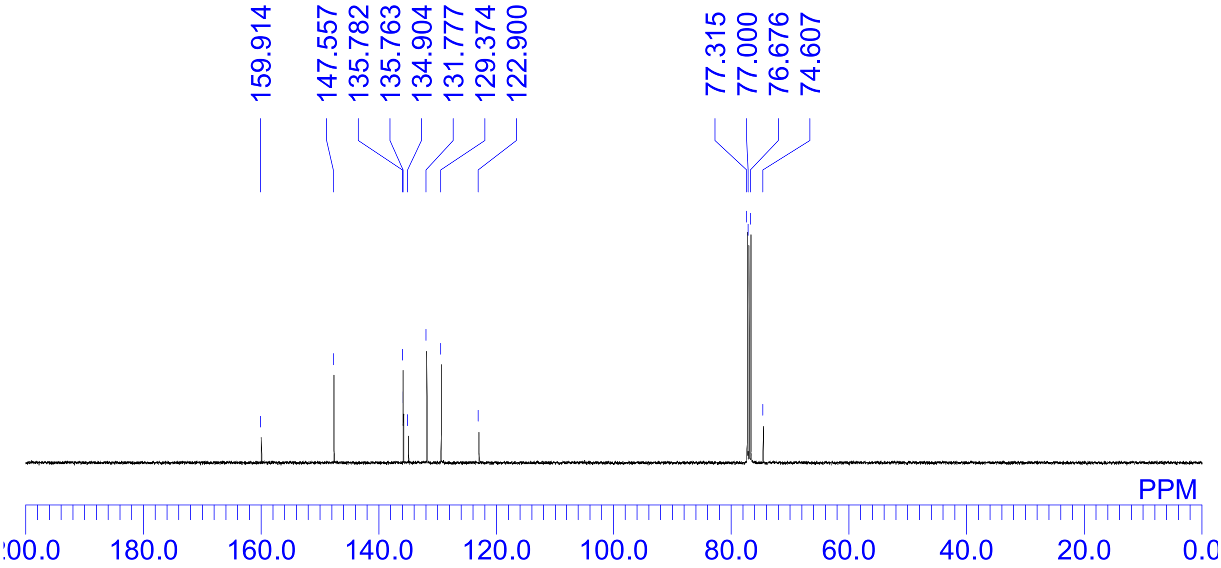

(**24h**) 7-fluoro-4-iodo-1*H*-isochromen-1-one

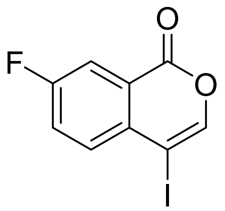

^{1}H NMR (400 MHz, in $CDCl_3$)

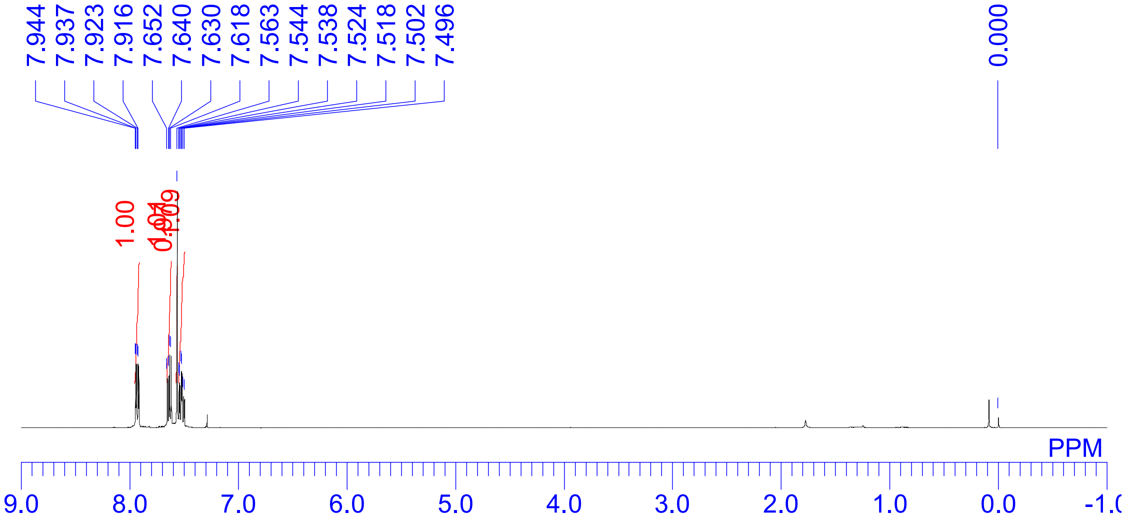

^{13}C NMR (100 MHz, in $CDCl_3$)

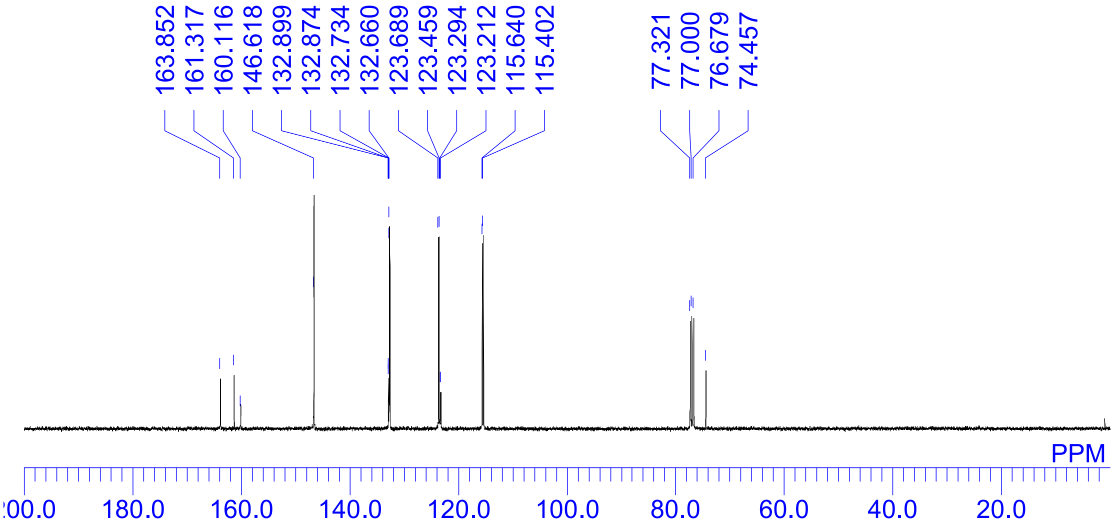

(**24i)** 4-iodo-3-phenyl-1*H*-isochromen-1-one

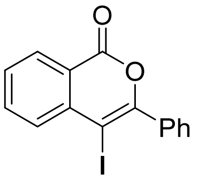

^{1}H NMR (400 MHz, in $CDCl_3$)

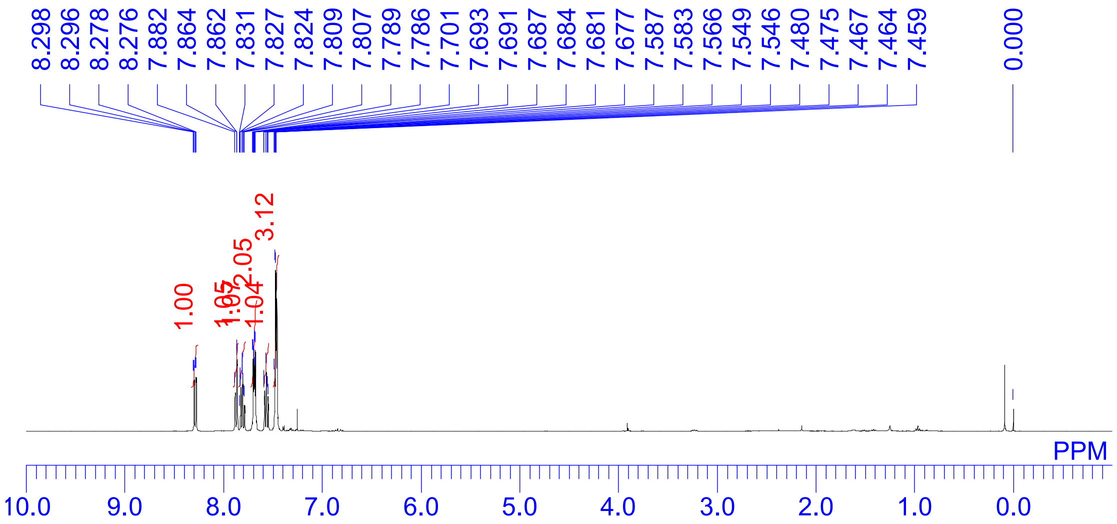

^{13}C NMR (100 MHz, in $CDCl_3$)

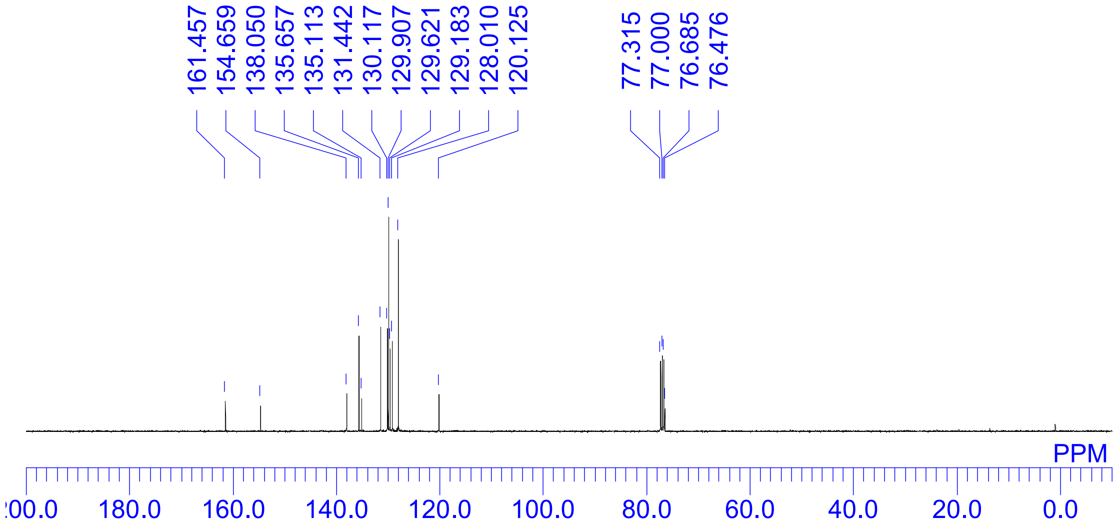

(**24j**) 3-(4-fluorophenyl)-4-iodo-1*H*-isochromen-1-one

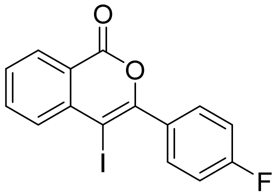

^{1}H NMR (400 MHz, in CD_2Cl_2)

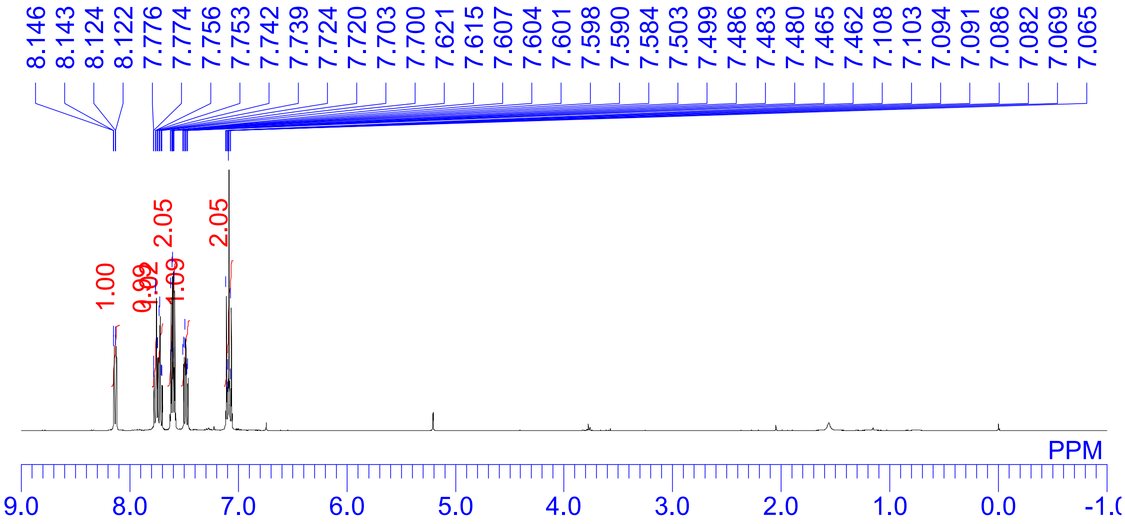

^{13}C NMR (100 MHz, in CD_2Cl_2)

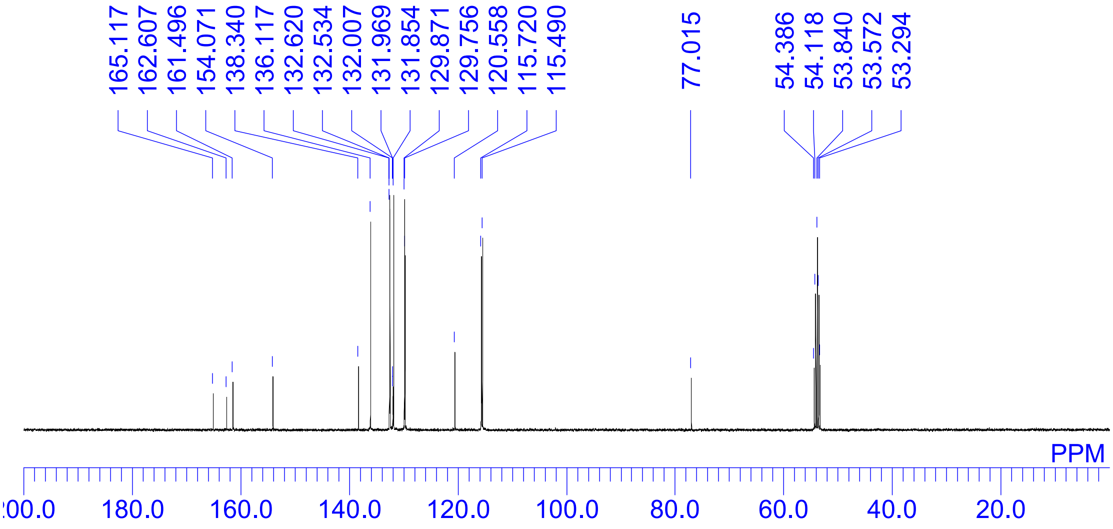

(**25k**) 4-bromo-3-butyl-1*H*-isochromen-1-one

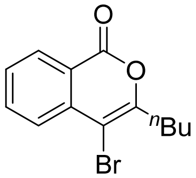

^{1}H NMR (400 MHz, in CD_2Cl_2)

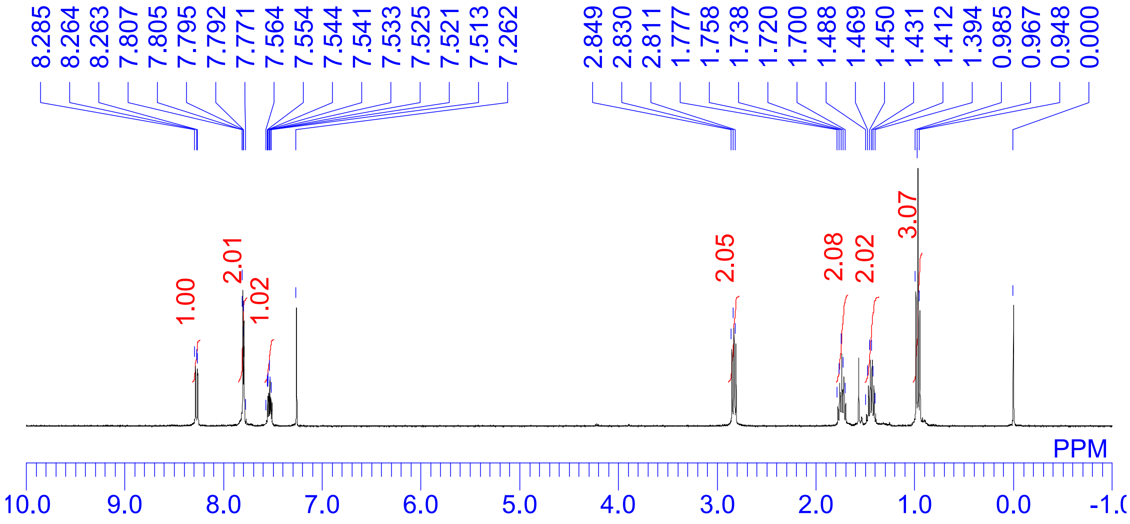

^{13}C NMR (100 MHz, in CD_2Cl_2)

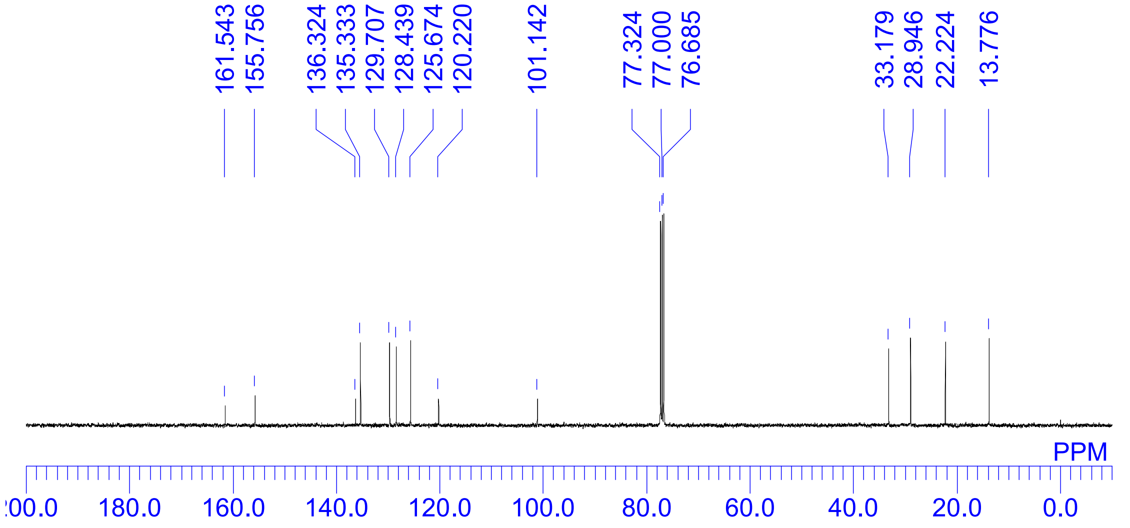

(**28aa**) 4-phenyl-1*H*-isochromen-1-one

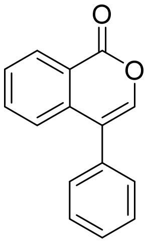

^{1}H NMR (400 MHz, in CD_2Cl_2)

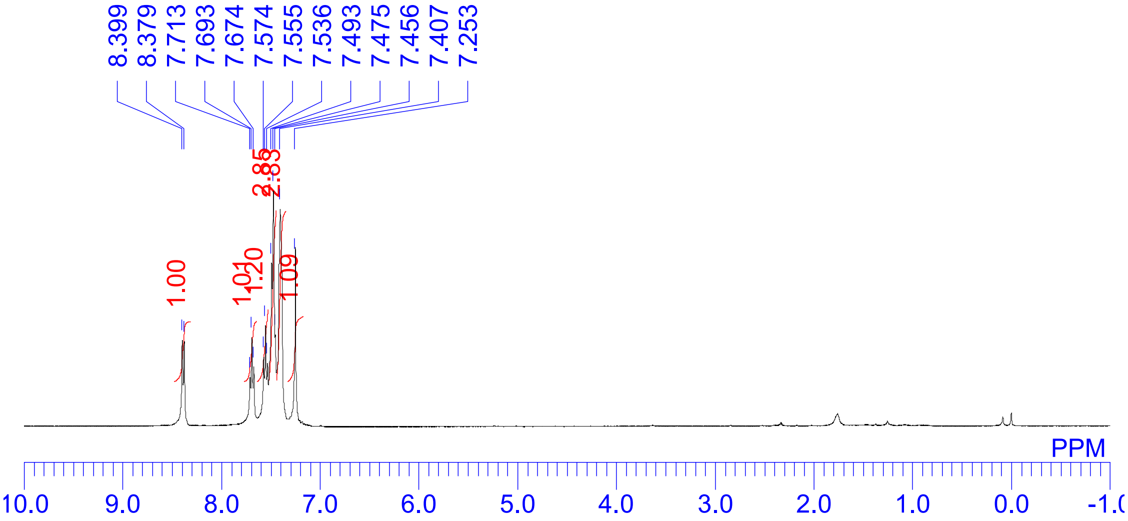

^{13}C NMR (100 MHz, in CD_2Cl_2)

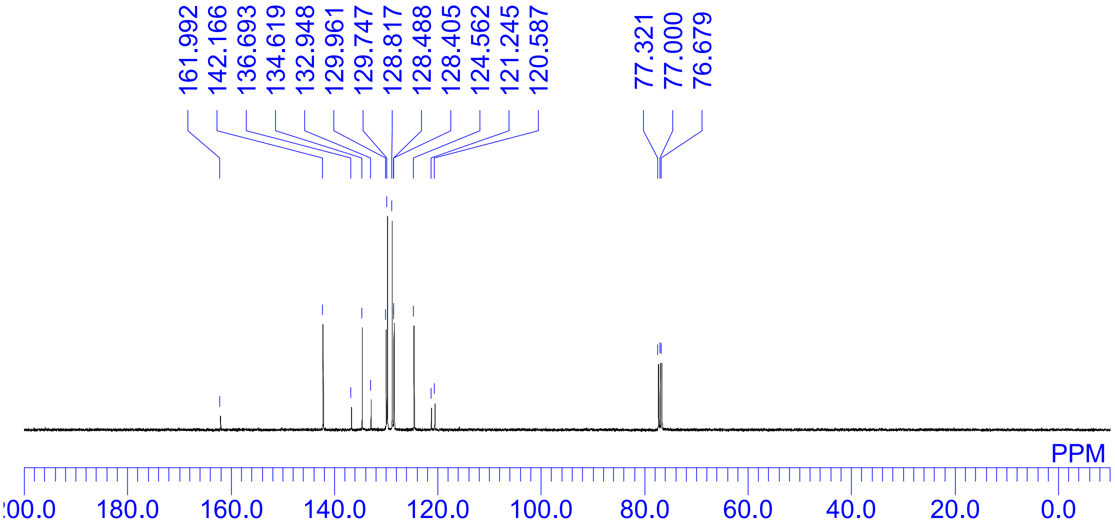

(**28ab**) 4-(4-methoxyphenyl)-1*H*-isochromen-1-one

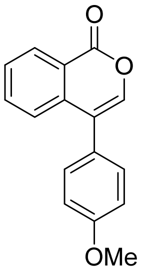

^{1}H NMR (400 MHz, in $CDCl_3$)

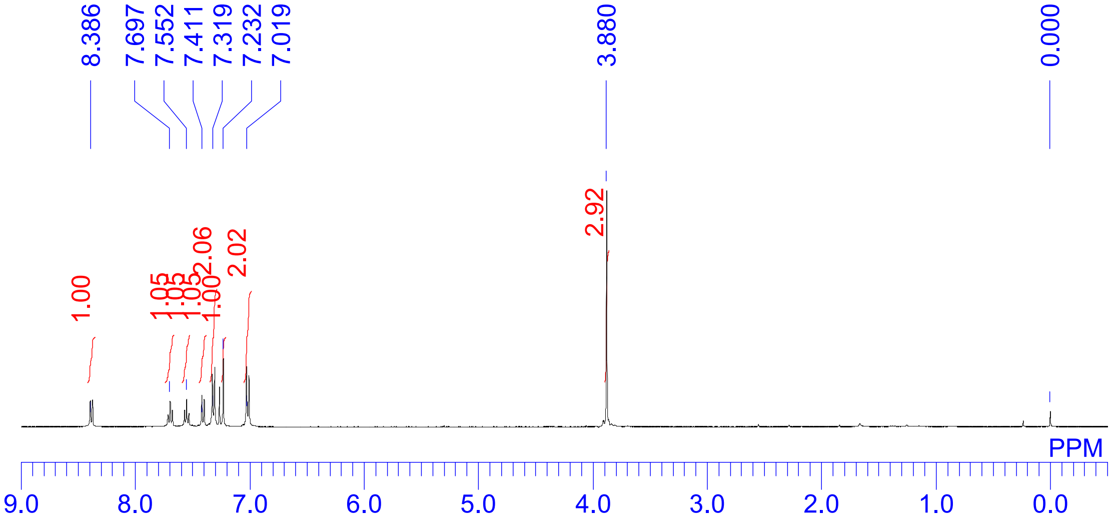

^{13}C NMR (100 MHz, in $CDCl_3$)

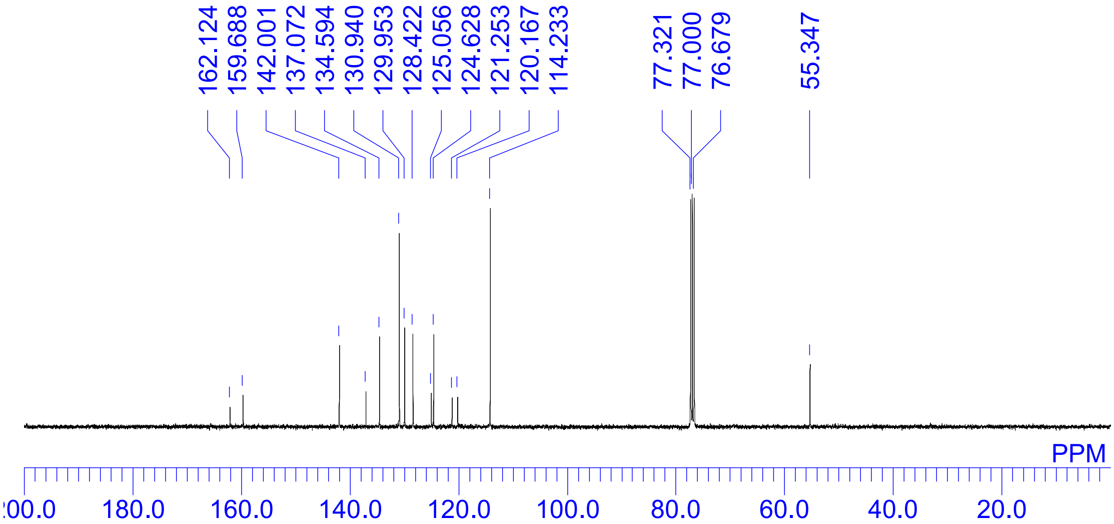

(**28ac**) 4-(1-oxo-1*H*-isochromen-4-yl)benzonitrile

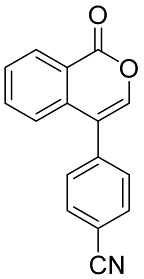

^{1}H NMR (400 MHz, in $CDCl_3$)

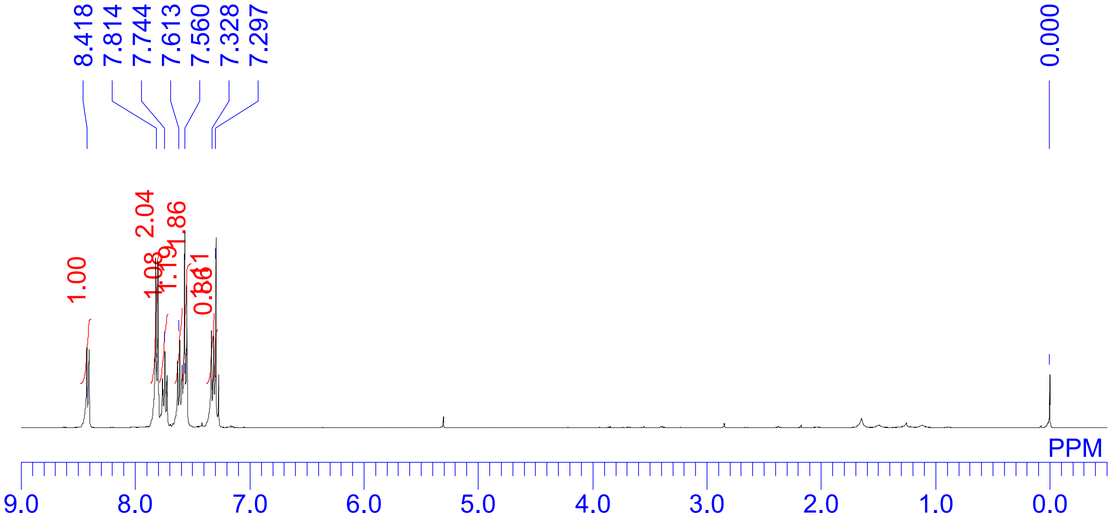

^{13}C NMR (100 MHz, in $CDCl_3$)

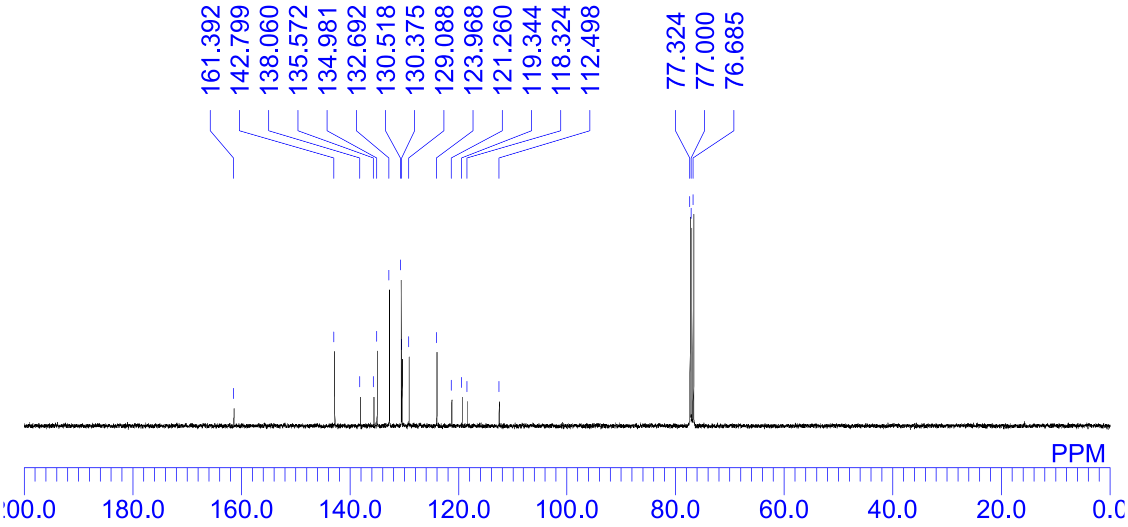

(**28ad**) 4-benzoyl-1*H*-isochromen-1-one

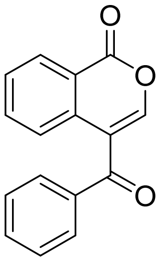

^{1}H NMR (400 MHz, in $CDCl_3$)

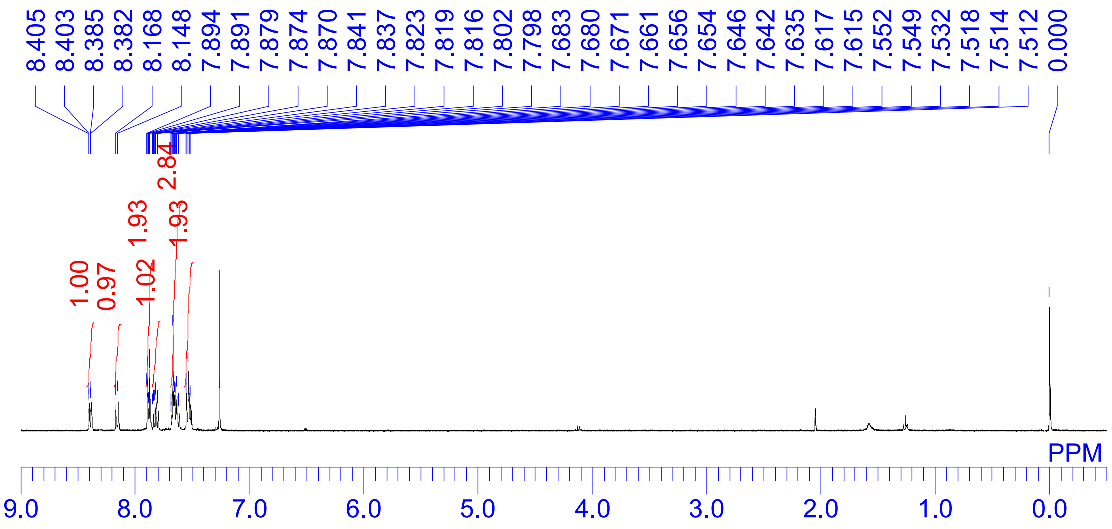

^{13}C NMR (100 MHz, in $CDCl_3$)

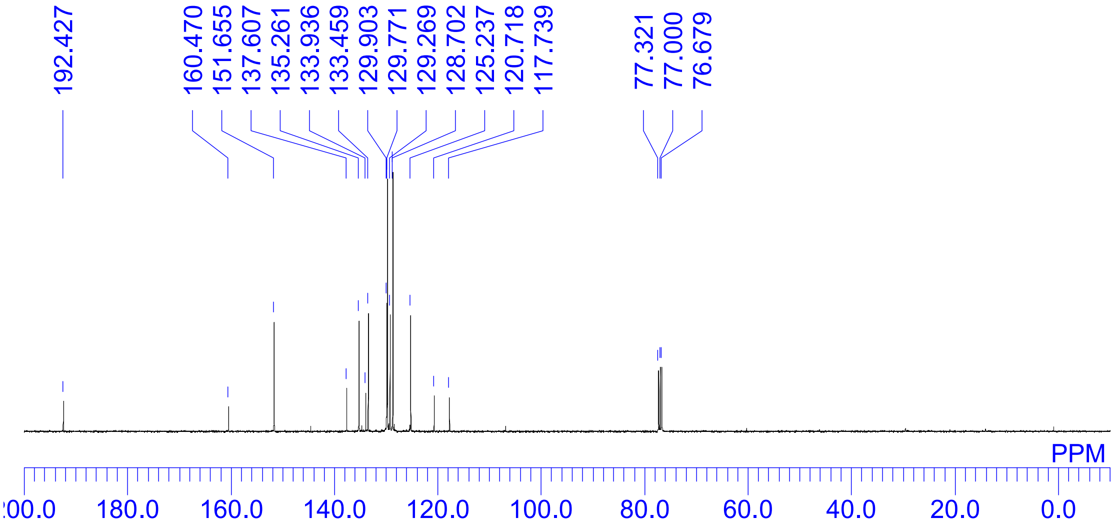

(**28ae**) 4-(4-chlorobenzoyl)-1*H*-isochromen-1-one

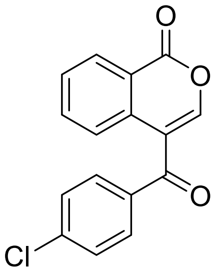

^{1}H NMR (400 MHz, in $CDCl_3$)

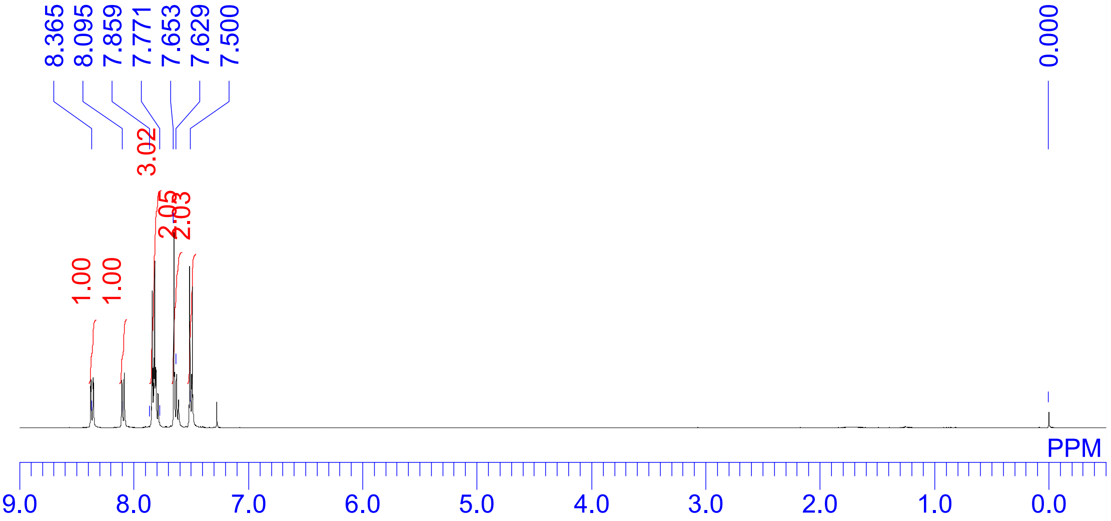

^{13}C NMR (100 MHz, in $CDCl_3$)

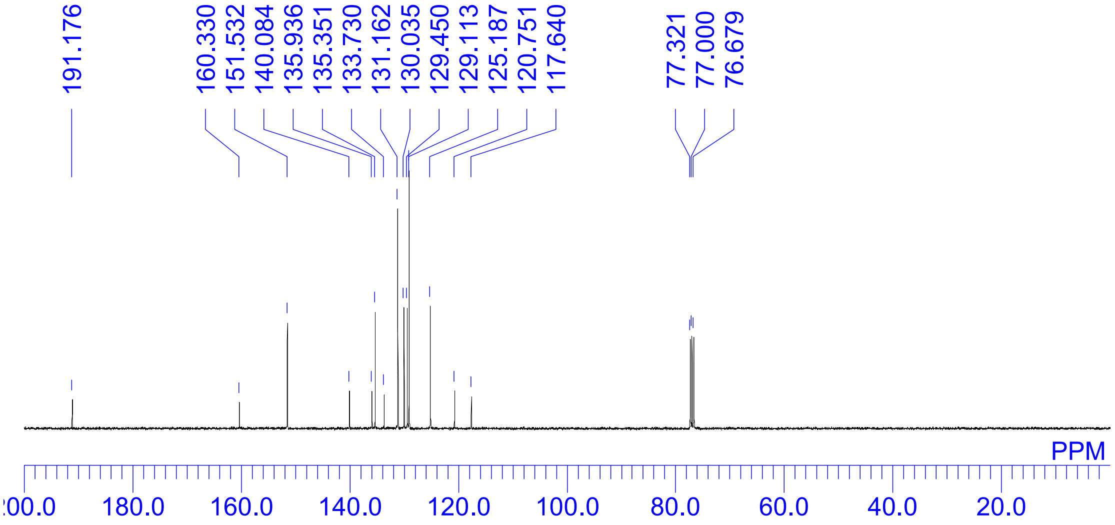

(**28af**) 4-isobutyryl-1*H*-isochromen-1-one

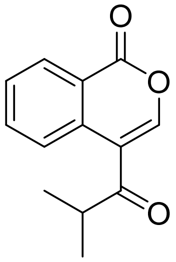

^{1}H NMR (400 MHz, in $CDCl_3$)

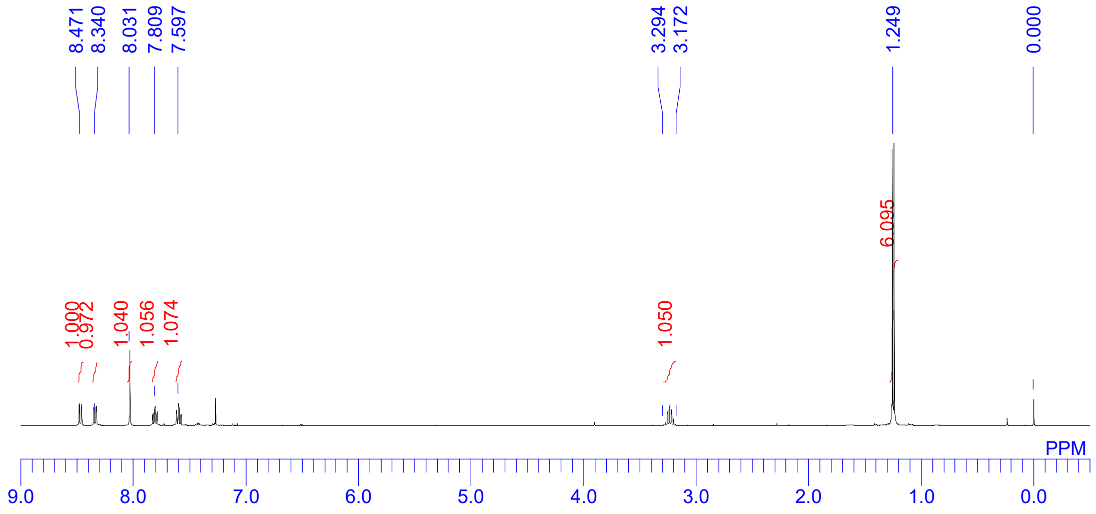

^{13}C NMR (100 MHz, in $CDCl_3$)

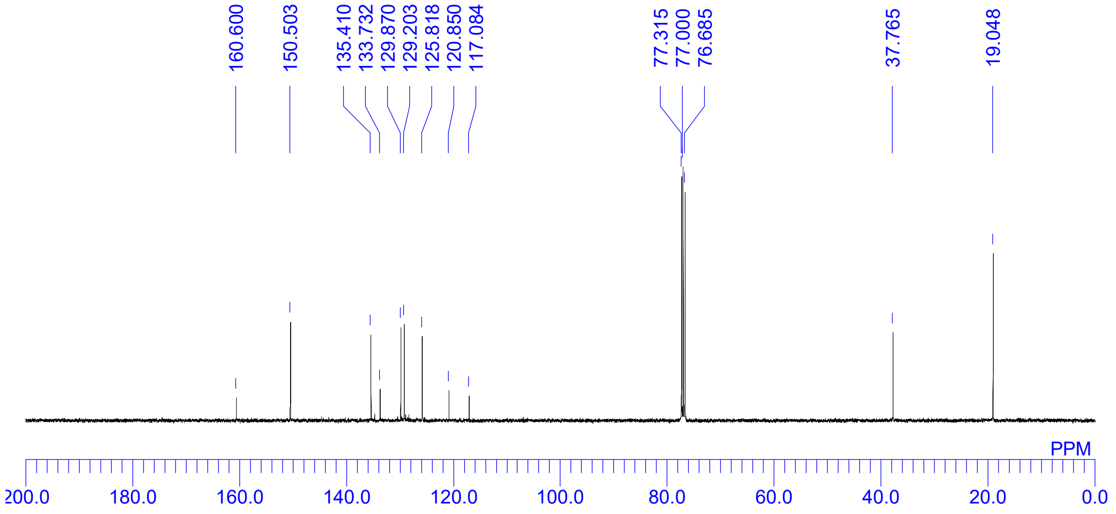

(**28ag**) 4-benzyl-1*H*-isochromen-1-one

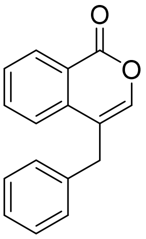

^{1}H NMR (400 MHz, in $CDCl_3$)

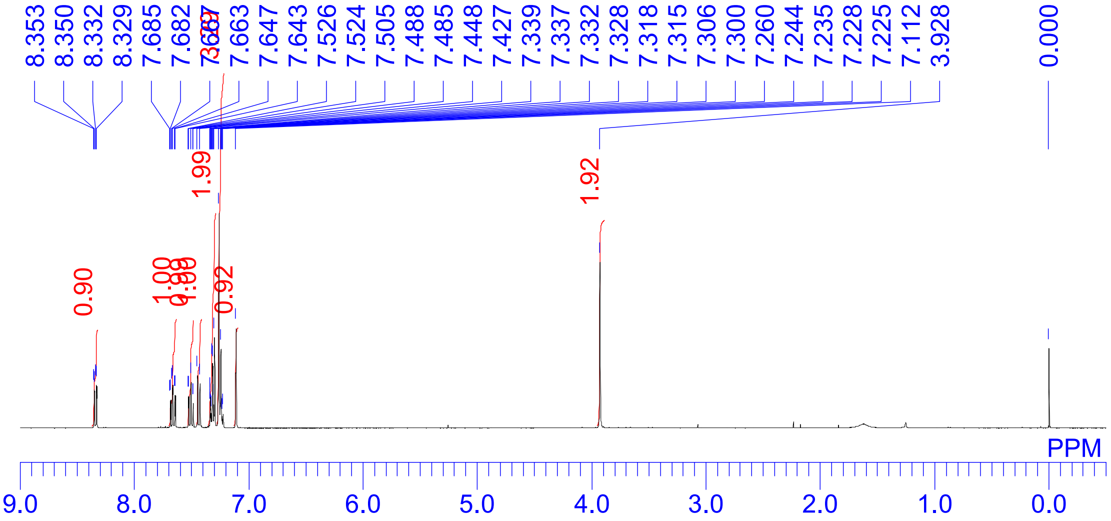

^{13}C NMR (100 MHz, in $CDCl_3$)

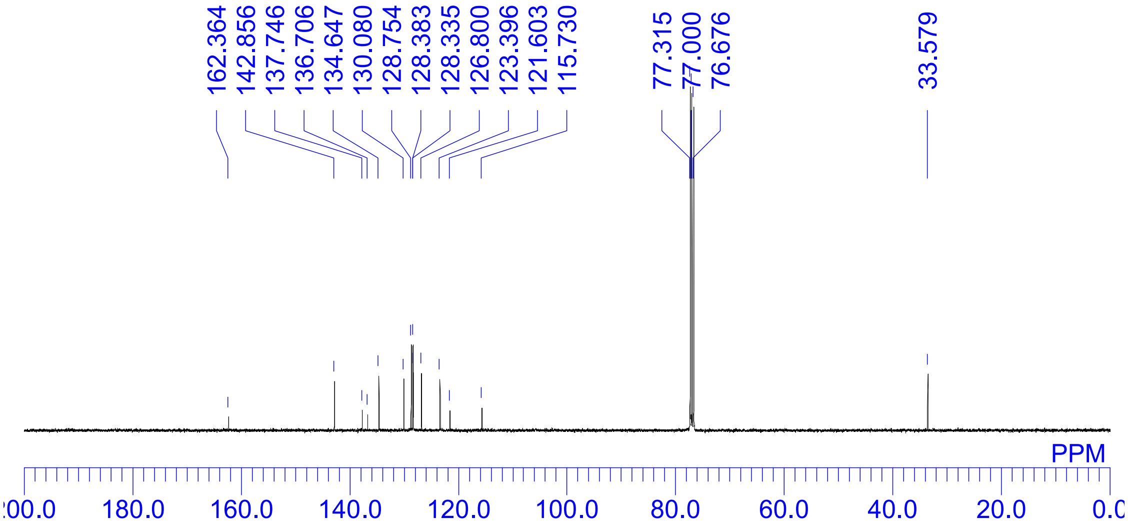

(**28ah**) 4-(but-2-en-1-yl)-1*H*-isochromen-1-one

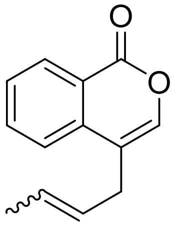

^{1}H NMR (400 MHz, in $CDCl_3$)

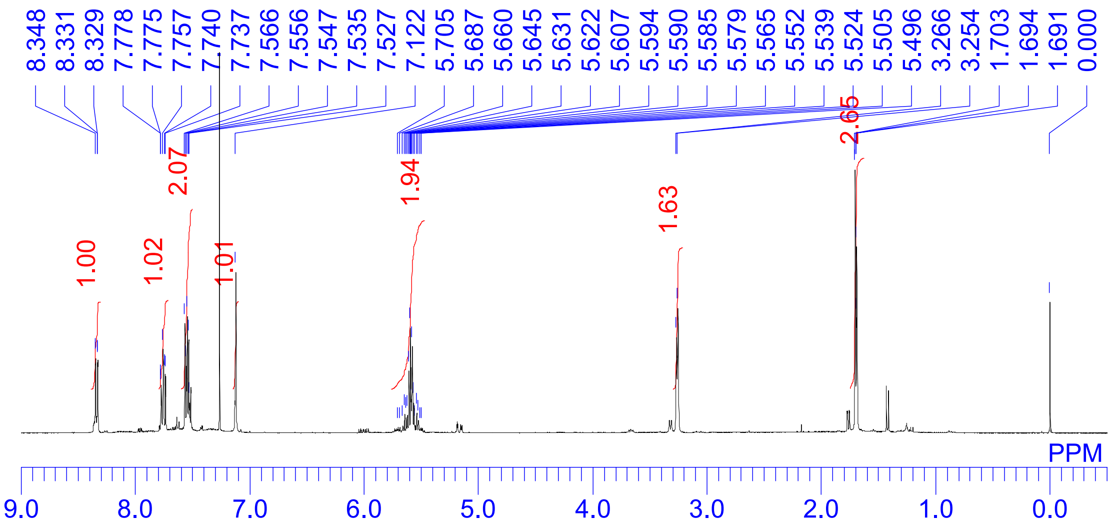

^{13}C NMR (100 MHz, in $CDCl_3$)

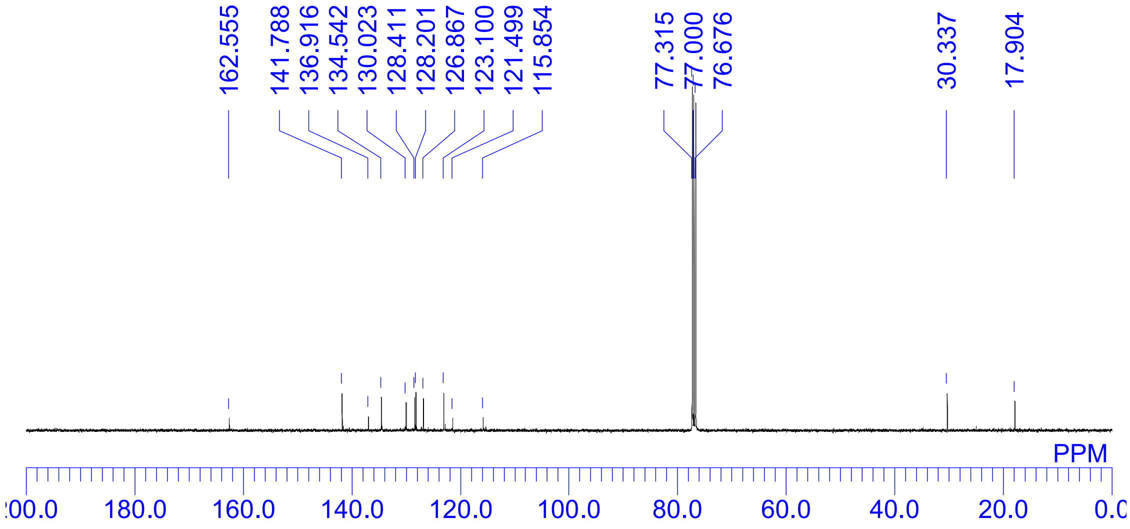

(**30**) 2-iodo-6-methoxybenzoic acid

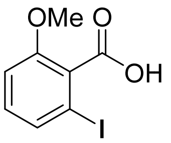

^{1}H NMR (400 MHz, in $CDCl_3$)

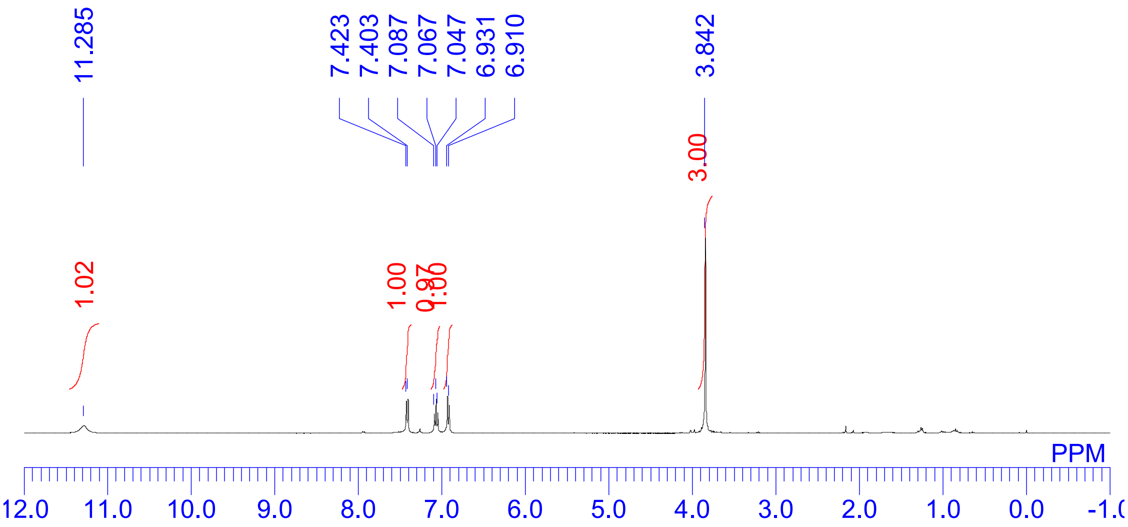

^{13}C NMR (100 MHz, in $CDCl_3$)

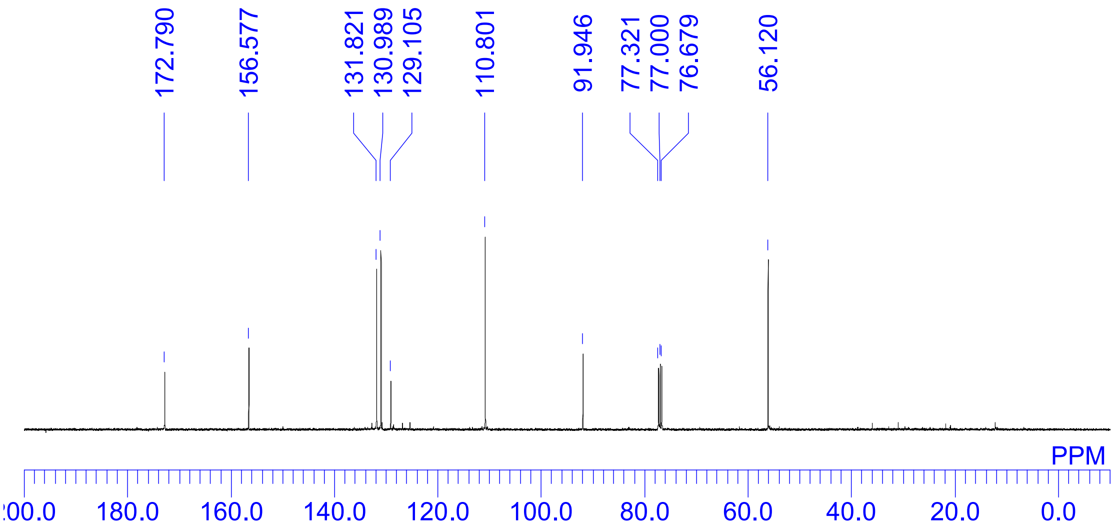

(**31**) 2-hydroxy-6-iodobenzoic acid

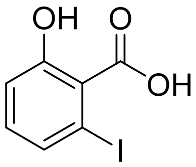

^{1}H NMR (400 MHz, in acetone-d_6)

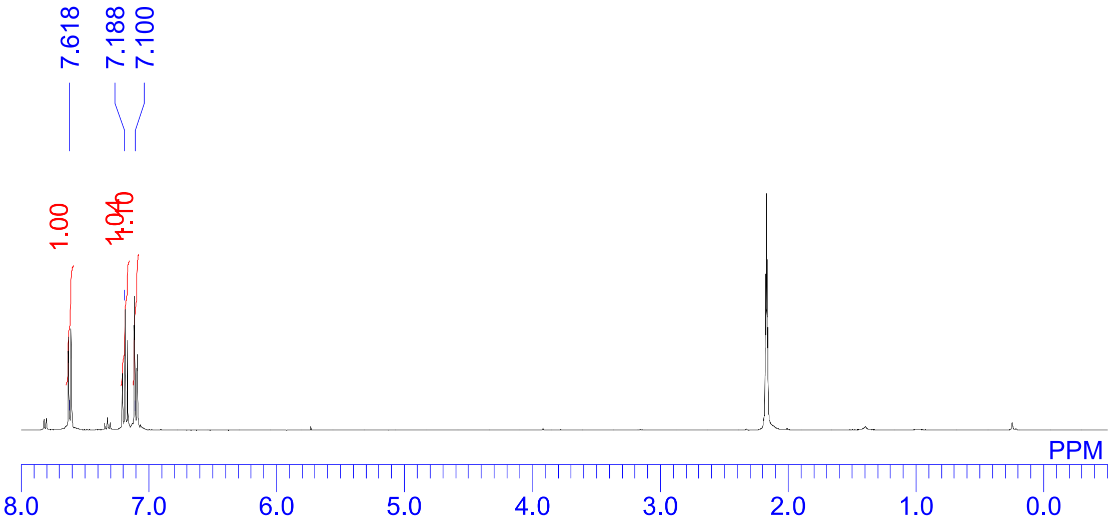

^{13}C NMR (100 MHz, in acetone-d_6)

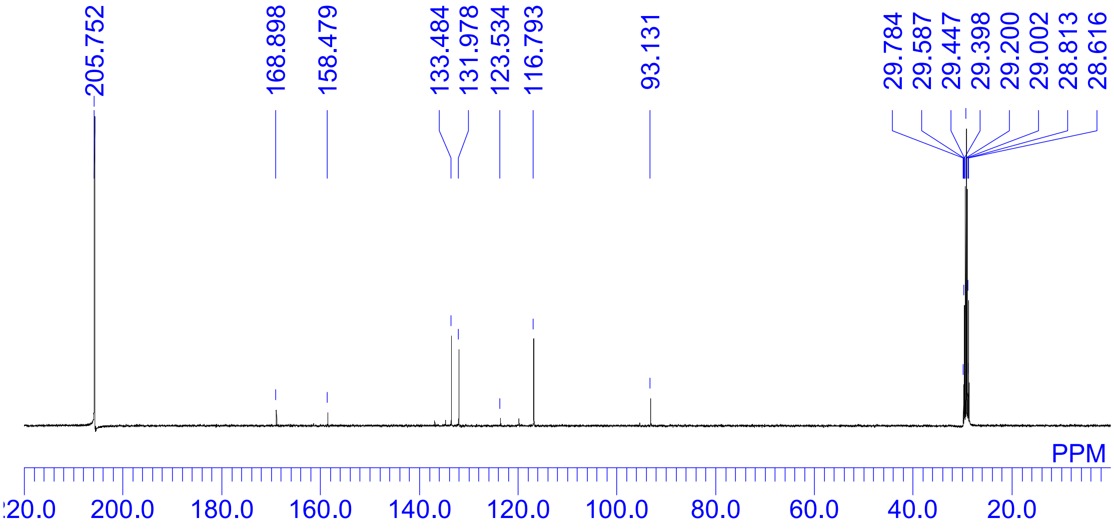

(**32**) methyl 2-hydroxy-6-iodobenzoate

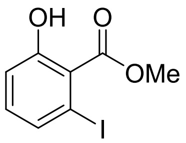

^{1}H NMR (400 MHz, in $CDCl_3$)

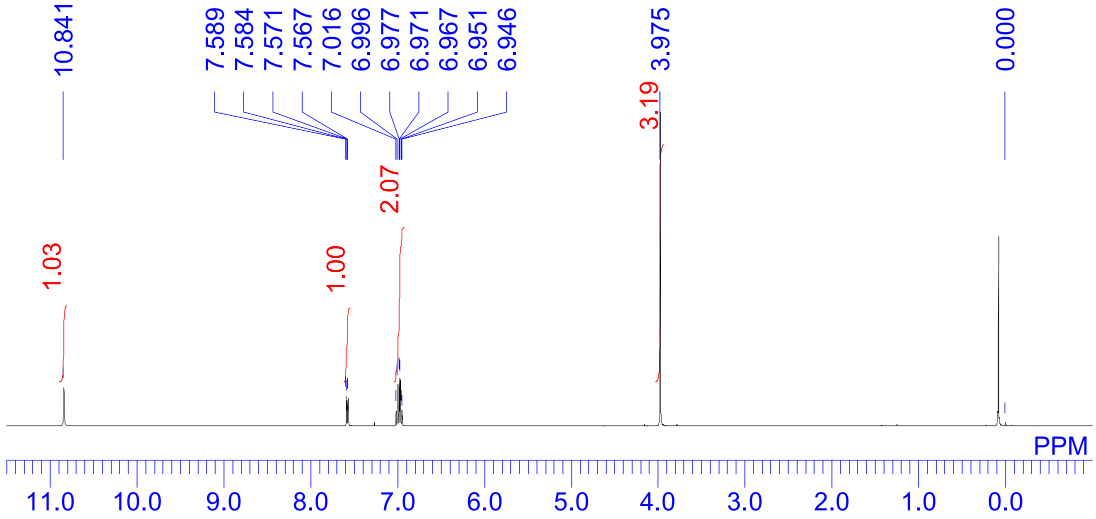

^{13}C NMR (100 MHz, in $CDCl_3$)

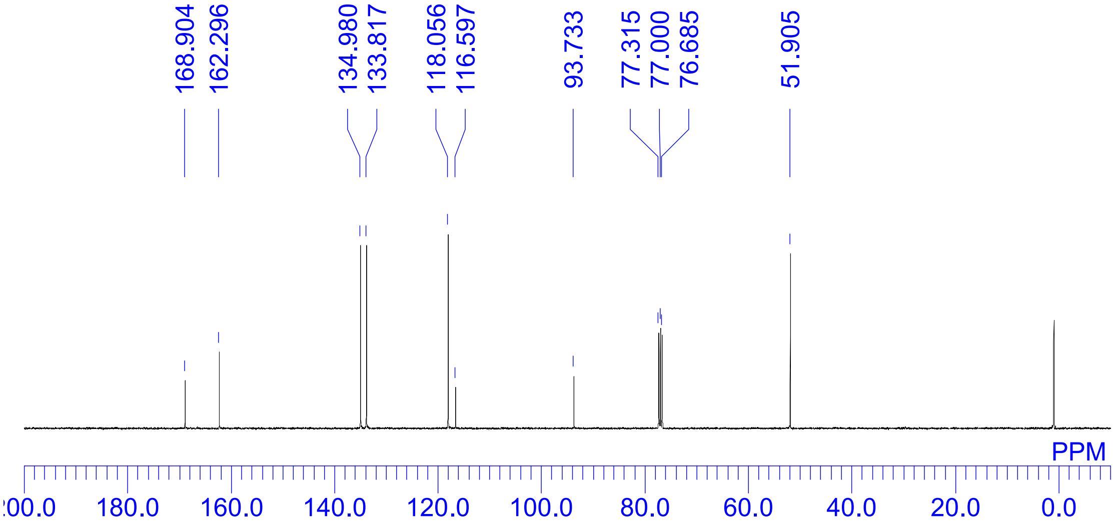

(**33**) methyl 2-acetoxy-6-iodobenzoate

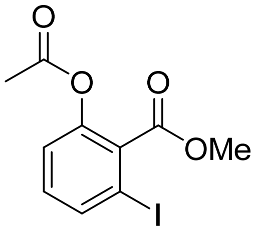

^{1}H NMR (400 MHz, in $CDCl_3$)

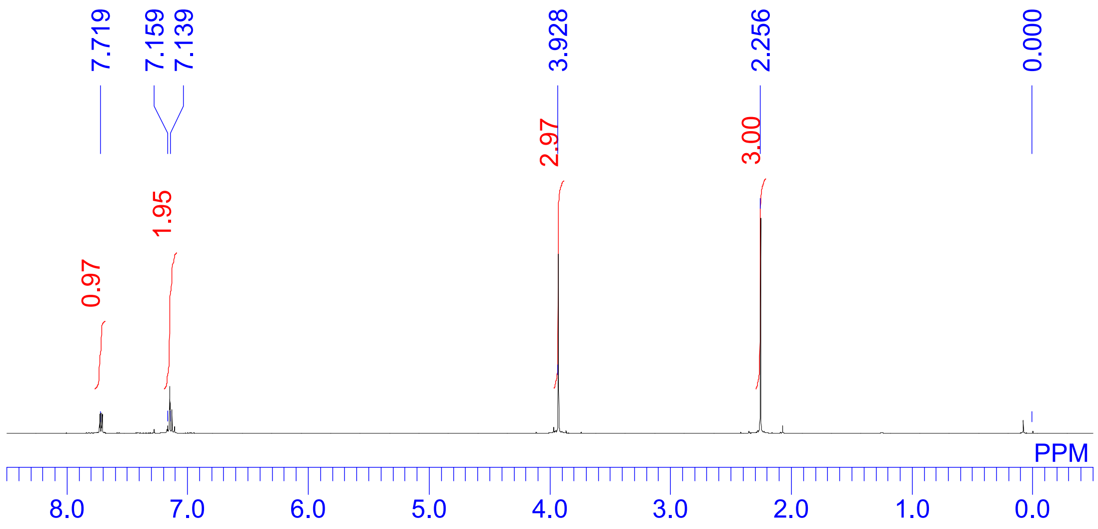

^{13}C NMR (100 MHz, in $CDCl_3$)

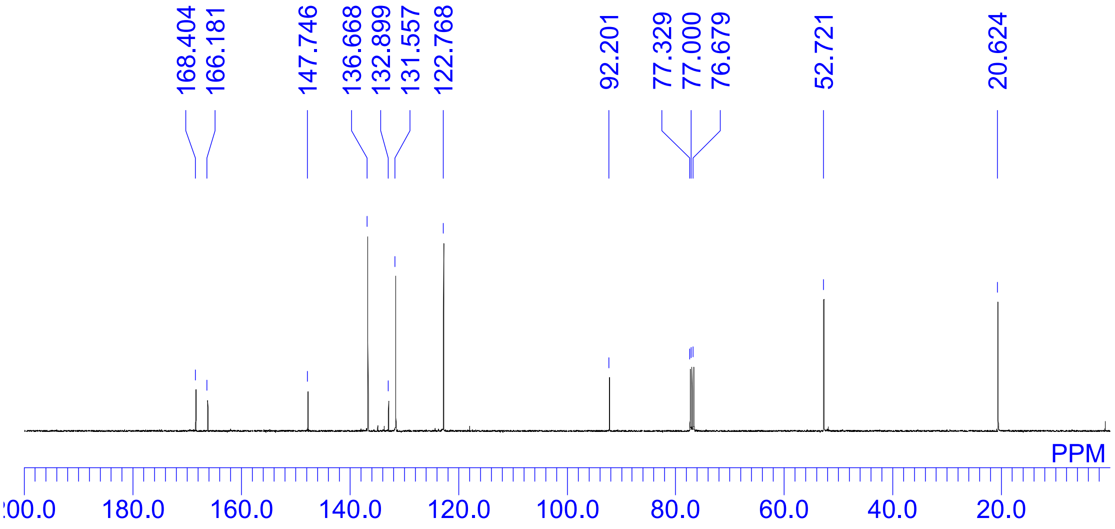

(**34**) methyl 2-acetoxy-6-((trimethylsilyl)ethynyl)benzoate

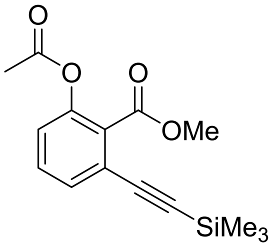

^{1}H NMR (400 MHz, in $CDCl_3$)

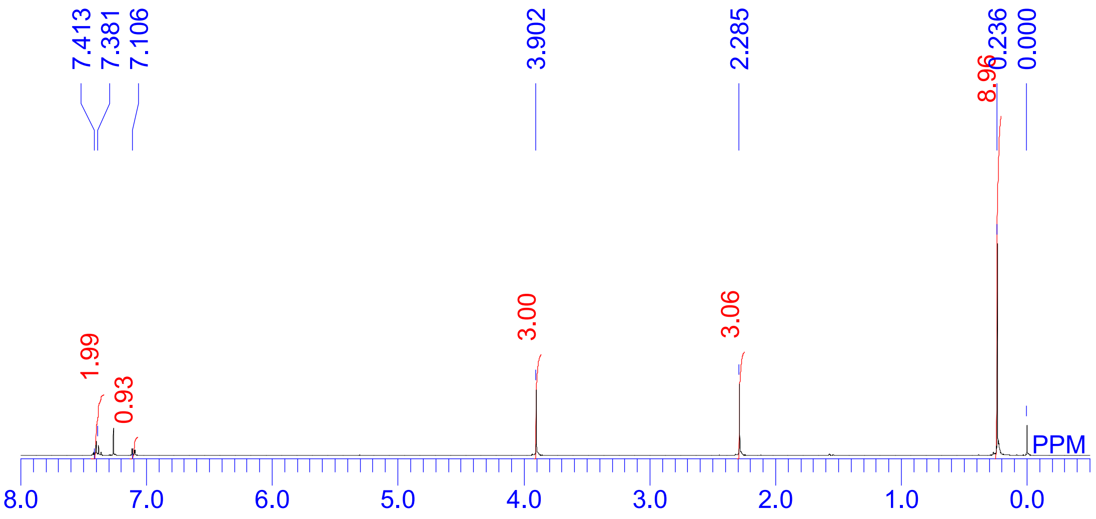

^{13}C NMR (100 MHz, in $CDCl_3$)

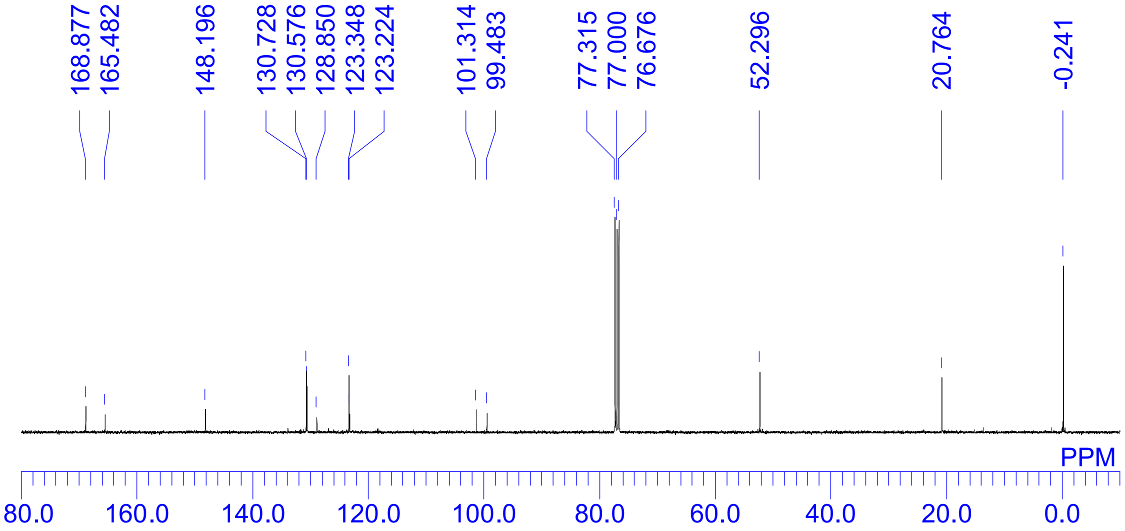

(**35**) methyl 2-acetoxy-6-ethynylbenzoate

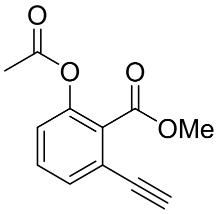

^{1}H NMR (400 MHz, in $CDCl_3$)

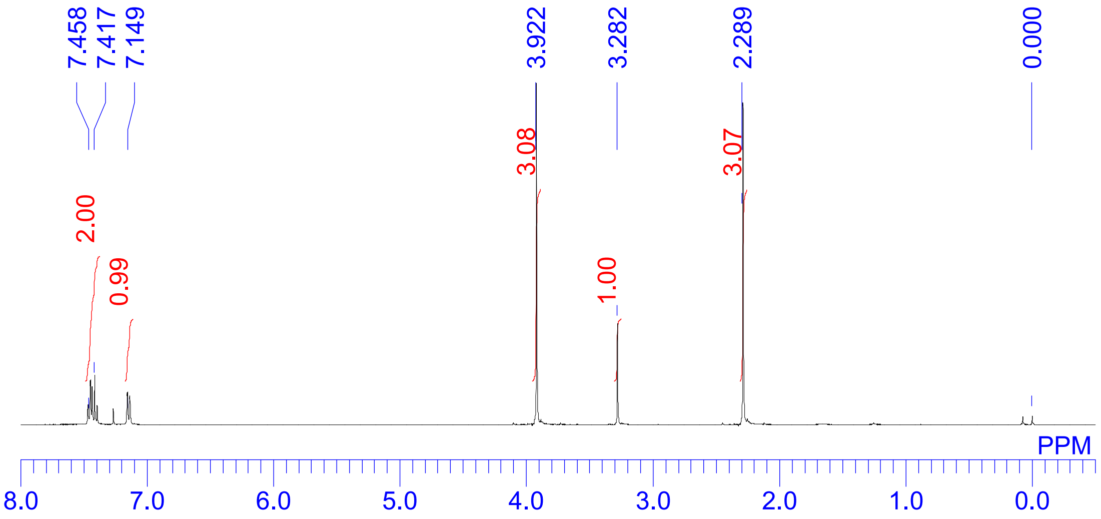

^{13}C NMR (100 MHz, in $CDCl_3$)

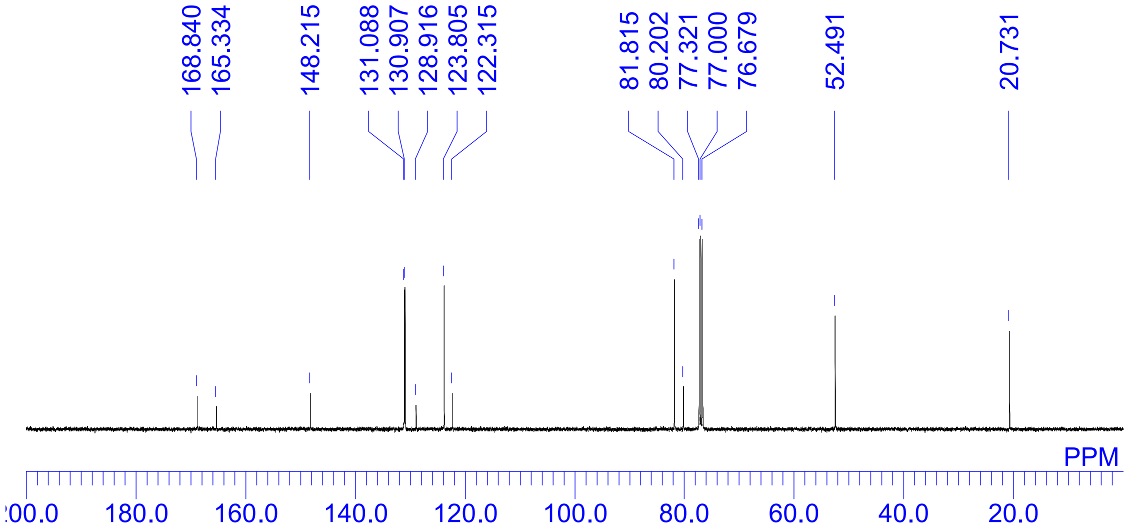

(**36**) 2-(8-acetoxy-1-oxo-1*H*-isochromen-4-yl)-2-oxoethyl acetate

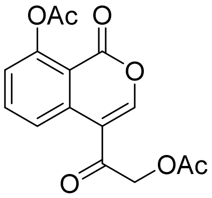

^{1}H NMR (400 MHz, in $CDCl_3$)

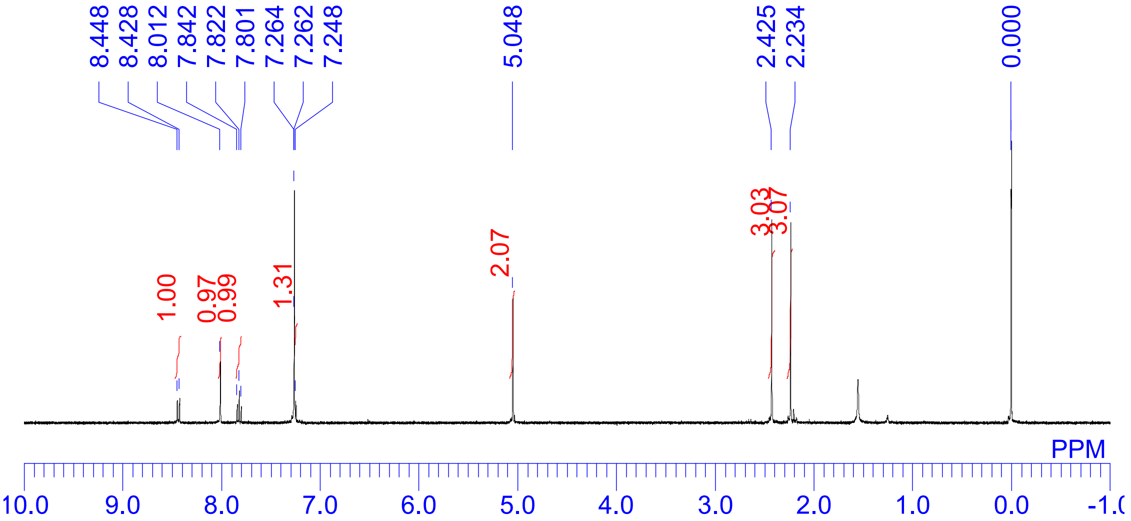

^{13}C NMR (100 MHz, in $CDCl_3$)

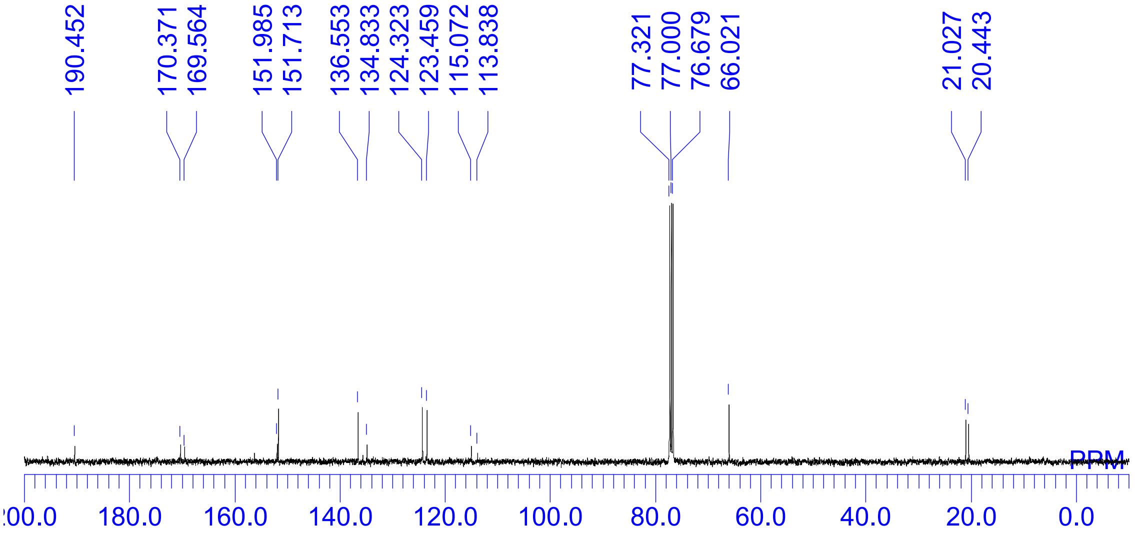

Supplement: Supplementary file 1 [file SC-009-C8SC01537F-s001.pdf]
